# Advancing drug development for atrial fibrillation by prioritising findings from human genetic association studies

**DOI:** 10.1016/j.ebiom.2024.105194

**Published:** 2024-06-27

**Authors:** Kishore Kukendrarajah, Aliki-Eleni Farmaki, Pier D. Lambiase, Richard Schilling, Chris Finan, Amand Floriaan Schmidt, Rui Providencia

**Affiliations:** a Institute of Health Informatics, University College London, 222 Euston Road, NW1 2DA, United Kingdom; b Barts Heart Centre, St Bartholomew's Hospital, West Smithfield, EC1A 7BE, United Kingdom; c Institute of Cardiovascular Science, University College London, Gower Street, WC1E 6HX, United Kingdom; d UCL British Heart Foundation Research Accelerator, United Kingdom; e Division Heart and Lungs, Department of Cardiology, University Medical Center Utrecht, Utrecht University, the Netherlands; f Department of Cardiology, Amsterdam Cardiovascular Sciences, Amsterdam University Medical Centres, University of Amsterdam, the Netherlands

**Keywords:** Atrial fibrillation, Drug development, GWAS, Systematic review, Bioinformatics

## Abstract

**Background:**

Drug development for atrial fibrillation (AF) has failed to yield new approved compounds. We sought to identify and prioritise potential druggable targets with support from human genetics, by integrating the available evidence with bioinformatics sources relevant for AF drug development.

**Methods:**

Genetic hits for AF and related traits were identified through structured search of MEDLINE. Genes derived from each paper were cross-referenced with the OpenTargets platform for drug interactions. Confirmation/validation was demonstrated through structured searches and review of evidence on MEDLINE and ClinialTrials.gov for each drug and its association with AF.

**Findings:**

613 unique drugs were identified, with 21 already included in AF Guidelines. Cardiovascular drugs from classes not currently used for AF (e.g. ranolazine and carperitide) and anti-inflammatory drugs (e.g. dexamethasone and mehylprednisolone) had evidence of potential benefit. Further targets were considered druggable but remain open for drug development.

**Interpretation:**

Our systematic approach, combining evidence from different bioinformatics platforms, identified drug repurposing opportunities and druggable targets for AF.

**Funding:**

KK is supported by 10.13039/100015652Barts Charity grant G-002089 and is mentored on the AFGen 2023-24 Fellowship funded by the AFGen NIH/10.13039/100000050NHLBI grant R01HL092577. RP is supported by the UCL BHF Research Accelerator AA/18/6/34223 and 10.13039/501100000272NIHR grant NIHR129463. AFS is supported by the 10.13039/501100000274BHF grants PG/18/5033837, PG/22/10989 and UCL BHF Accelerator AA/18/6/34223 as well as the 10.13039/100014013UK Research and Innovation (UKRI) under the UK government’s Horizon Europe funding guarantee EP/Z000211/1 and by the UKRI-NIHR grant MR/V033867/1 for the Multimorbidity Mechanism and Therapeutics Research Collaboration. AF is supported by UCL BHF Accelerator AA/18/6/34223. CF is supported by UCL BHF Accelerator AA/18/6/34223.

**Research in context**

**Evidence before this study**

There is an abundance of data on the genetic associations of atrial fibrillation (AF) and its associated traits (including PR interval and LA indices). Some of these data also include information on associations between coding variations (commonly single nucleotide polymorphisms or SNPs) and changes in mRNA expression of genes. Online bioinformatics libraries contain information on target-drug interactions, mechanisms and indications of these drugs as well as information on target tractability predictions for drug development. In addition, the online publication library MEDLINE and clinical trials registry ClinicalTrials.gov contain clinical evidence linking some of these drugs to outcomes for AF. To the best of our knowledge, an evidence synthesis framework to connect these different layers of data and explore the links between genes identified from these studies and potential therapeutic compounds which may have an effect on AF outcomes, is lacking. We sought to provide a systematic approach using these resources along with a target-prioritisation strategy to synthesise the currently available evidence on (i) drugs that may be explored for repurposing opportunities or (ii) targets that could be explored for drug development.

**Added value of this study**

Our findings can be grouped into four categories: (i) Existing drugs that are already used for other cardiovascular indications that could be explored further for repurposing for management of AF. As these drugs are already used clinically, they are likely to have an adequate safety profile in the management of AF. Initial clinical trial validation could come from examining their effect on AF outcomes when concurrently treating for their existing indication if clinical equipoise exists with other alternatives; (ii) Drugs which have clear evidence of increasing AF risk. This suggests that the associated targets may have a role in underlying AF disease pathology and could provide further avenues for drugs developed following pre-clinical studies; (iii) Targets with available drug repurposing options that may be of interest for treating AF, but for whom no supporting clinical evidence was found; (iv) High-priority targets based on target tractability data which do not have associated drugs but could be options for further drug development.

**Implications of all the available evidence**

This methodology demonstrates a reproducible method for highlighting potential targets for drug development or drugs which may be suitable options for repurposing after further validation. Additional support for this approach comes from the fact that 21 of the drugs were present in guidelines for AF management. Application of a similar workflow to other disease entities with known large-scale genetic association data can be used to accelerate drug development and identify drug repurposing opportunities.

## Introduction

Atrial fibrillation (AF) is the most common sustained arrhythmia.^1^ Hospitalization for AF is on the rise, and it has now surpassed myocardial infarction and heart failure.^2^ As a disease of ageing, the burden of AF management on healthcare systems across the globe is set to increase. Better therapeutic options to treat and prevent AF could be a way to halt or stymie this epidemic,^3^ yet no new drugs have been approved in the last 10 years.^4^

With the advent of low-cost sequencing platforms^5^^,^^6^ and the maturation of statistical tools to generate inferences from genomic data,^7^ vast knowledge has been accumulated on the genetic associations of a multitude of common diseases, with hundreds of genetic markers identified for AF and its associated traits^8^, ^9^, ^10^ Unfortunately, this flourishing of knowledge in AF genetics has not translated to the development or identification of novel drug options for the condition.

Progress in bioinformatics has led to availability of: (i) large open-source libraries of bioactive molecules with drug-like properties^11^ including protein sequencing and functional information,^12^ resulting in a better understanding of druggable proteins. Finan et al. have estimated 4479 (22%) of the 20,300 protein-coding genes to be drugged or druggable^13^ (ii) information on associations of AF with drugs used for the treatment of cardiovascular and non-cardiovascular conditions in humans in open-source medical literature^14^ and clinical trial libraries.^15^

**Fig. 1:**
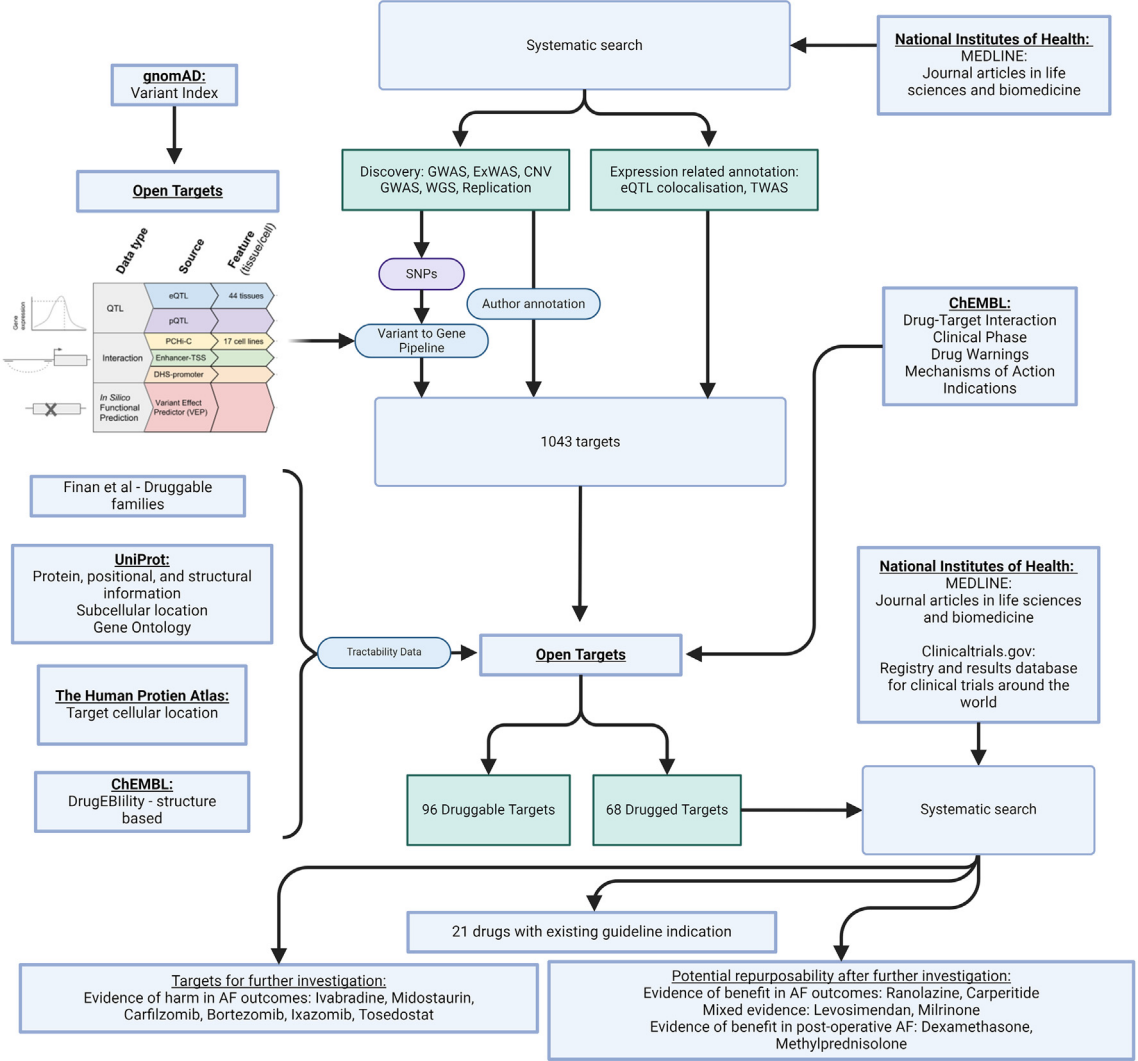
Workflow diagram detailing sources of evidence including bioinformatics libraries, clinical trial registries and journal databases. Created with BioRender.com. Partly adapted from–https://genetics-docs.opentargets.org/our-approach/data-pipeline.^16^

A systematic approach with cross-linking of these data sources and generation of evidence synthesis for the obtained outputs may lead to: (a) identification of targets for drug development for whom no active compounds are currently available, (b) drug repurposing options (i.e. drugs that may be of benefit outside of their originally approved indication), some of which with available clinical trial data support and potentially ready for phase-4 randomized controlled trials, and (c) disease-causing drugs highlighting important disease-associated pathways. Fig. 1 outlines our workflow using these sources. This approach could be applied not only to AF, but also to any disease for whom extensive genetic or protein-association data is available, thereby accelerating global drug-development. It is the wealth of information on AF that makes it an ideal subject to use as a case study in developing a systematic workflow.

To the best of our knowledge, a systematic evidence synthesis process, combining the data sources mentioned above for addressing this matter, has not yet been developed. We aimed to assess the feasibility of such an approach and whether this would identify treatment options for AF.

## Methods

### Selection of genetic association study data

Our approach is designed to cast a wide net over the available data on genetic association to AF. We believe this will allow us to identify as many genes that may have a function in AF disease pathogenesis as possible. Therefore, we included genome wide association studies (GWAS), as well as studies that employed whole genome sequencing (WGS) or copy number variant (CNV) association analyses that identified variants associated with either AF (AF or atrial flutter, early onset AF and lone AF) or AF related traits (Left atrial size, volume and phasic function as well as PR segment features). These AF related traits are related to the disease pathology and therefore including such studies may identify further genes related to the disease process or replicate those found in AF association analyses. Papers that were supplementary to original GWAS publications, in that they included expression Quantitative Trait Loci (eQTL) analyses, additional exome wide (ExWAS) or rare variant analyses (RVAS), were also included.

Studies looking at variants colocalising with eQTLs provide further support of functional significance of those genes and ExWAS and RVAS studies identify genes with large effect sizes on AF which may suggest particular importance in disease pathology. Any ancestry was eligible for inclusion as well as cross-ancestry studies to maximise generalisability of the findings. We did not include studies using novel methods for annotation of variants as they are not routinely replicated and therefore, we cannot ensure the validity of their approaches. Specific & selected patient populations (e.g. post-cardiac surgery or undergoing catheter ablation) were also excluded as the pathology underlying these phenotypes is markedly different.

#### Search Strategy

We searched the MEDLINE database on the 12th January 2024. The search was (“atrial fibrillation” OR “atrial” OR “atrium” OR ″P wave”) AND (“GWAS” OR “Genome-wide association study” OR “WGS” OR “Whole Genome Study” OR “ExWAS” OR “RVAS” OR “Exome-wide association study” OR “Rare variant association study”).

Authors RP and KK independently screened the results. The PRISMA 2020 flow diagram was created using the R package PRISMA2020.^17^

### Data extraction

Each study provided annotation of variants identified by the closest gene as well as previously identified genes at that locus. However, as there remains uncertainty on using proximity measures to annotate the likely affected gene, we used the OpenTargets variant to gene pipeline instead. This approach integrates information on QTL experiments, chromatin interaction experiments, in silico functional prediction and distance from transcriptional start site to provide an annotation score.^16^ For variants that were not present in the OpenTargets database we used the original authors annotation. We believe this approach maximises the reproducibility of this method. If multiple ancestries were analysed, data was extracted from supplementary material. Targets identified from eQTL colocalization analyses, Transcriptome Wide Association Studies (TWAS), ExWAS or RVAS were also extracted from the supplementary material. Extraction was based on tissue specificity to left or right atrium (LA or RA) and left ventricle (LV) from eQTL colocalization analyses as these were the data most routinely provided. Whilst other tissues such as immune system cells, liver, and other vascular tissues may also be relevant, we wanted to focus on targeting mechanisms driving pathophysiology intrinsic to cardiac tissue. Further studies may explore targets with changes in non-cardiac tissue expression only.

### Cross-referencing with bioinformatics database

The OpenTargets platform is an open-source resource that integrates over 20 different public data sources with available data including genetic associations, drug compounds and targets, diseases and phenotypes.^18^ We used this resource to extract relevant information from each target, including core target and drug-related data, as well as drug mechanism of action and clinical indication.

As some of the gene names provided in papers did not match the approved Human Genome Organisation Gene Nomenclature Committee (HGNC) label, unmatched targets were cross-referenced with synonyms provided in the core targets database. Ensembl IDs were obtained for each target. If names were still missing from the OpenTargets data download, a web search was employed to identify if and where the target was reported and Ensembl ID was extracted. Once data on reported targets, official HGNC symbol and Ensembl ID were available, the core target database was used to extract information on the gene biotype and the approved name of the gene (indicating its function).

OpenTargets provides information on the tractability of these targets. This includes information on druggability as detailed in Finan et al.^13^ as well as the DrugEBIlity score based on protein conformational structure provided by ChemBL for small molecule drugs.^19^ For potential targeting by antibody drugs (biologics) information on whether there was high confidence that the protein is either in the plasma membrane, extracellular matrix or secreted was available from UnitProt^12^ and the Gene Ontology (GO) database,^20^ as well as information on whether there was high confidence that the protein was in the plasma membrane from the Human Protein Atlas (HPA).^21^ A score of one was given for each gene in the following categories: (i) found to be in a druggable family (from Finan et al.); (ii) had a high or medium quality pocket from the DrugEBIlity score (≥0.7 and 0–0.7 respectively); (iii) had high confidence not to have a subcellular location from UnitProt, GO or HPA (one each). We prioritized druggability if the total score was >1 for either small molecule or antibody domains.

We also prioritised drug target genes according to reports from multiple study types. A score of one in each domain was given if the variant annotated to the gene was reported in: (i) an AF, PR indices or LA indices genetic association study (one per trait); (ii) if the gene was reported in an eQTL colocalization study or TWAS for LA, RA or LV tissues (one per tissue); (iii) an RVAS analysis. Priority was given to targets with a total score of >1.

Ensembl IDs were used to cross reference with the core drug data, extracting lists of drugs with known interactions with the targets identified. Data on drug approval, maximum clinical trial phase, black box warning, if a drug had been withdrawn, drug synonyms, and drug type were also obtained. Information on drug indication and mechanism of action was extracted for each drug from the respective databases. For drug mechanisms, information on specific action, action type, target name and target molecule type were also extracted.

Approved indications were presented by biomedical ontology nomenclature so the ‘Rols’ package from the Bioconductor library for R was used to decode.^22^ Evidence for indications from this database was given through a combination of clinical trial numbers, World Health Organisation (WHO) Anatomic Therapeutic Classification (ATC) and United States (US) Food and Drug Administration (FDA) labelling either from the FDA website or the Daily Med website. Indications were then manually coded into categories based on speciality with secondary indications based on intended function relevant to speciality. However, those recorded from the DailyMed website were excluded as coding was unreliable.

Drugs that had different salt formulations or those that were conjugated to a compound for pharmacokinetic or bioavailability purposes, were considered duplicates. However, those that were conjugated to other active compounds such as in the case of some chemotherapy drugs, were considered unique.

#### Data Visualisations

Data on drug mechanisms and indications were extracted from the relevant databases from OpenTargets using Ensembl IDs. Drug mechanism data was used to provide information on drug classes and action types through a circular dendrogram. For drug indications a chord diagram was constructed after creating a square adjacency matrix for overlapping indications.

### Literature review for drugs

Each of the identified drugs was run through the MEDLINE data base and Clinicaltrials.gov on the 15th of February 2024 for existing clinical data in humans of AF-related effects providing support to the findings. The structured search strategy used for MEDLINE was: “[drug name]” AND “atrial fibrillation”. On Clinicaltrials.gov “atrial fibrillation” was entered in the “Condition or disease” field and the drug name in the “Intervention/treatment” field. Example searches are provided under Supplementary Figure S1 and Supplementary Table S1.

Systematic reviews, randomized clinical trials (RCTs), and controlled studies in humans comparing the drug vs placebo or an active control were considered eligible for the evidence synthesis tables. Data on the effect of the drug on incident AF (either protective effect or side effect) was extracted. For each of the identified drugs we searched European Society of Cardiology,^23^ American Heart Association & American College of Cardiology^24^ for an existing guideline indication for the treatment of AF. If the drug was not available/approved in America or Europe, we used the local AF guideline.

### Role of funders

Funders for KK, AFS and RP did not have any role in the study design, data collection, analyses interpretation or writing of report.

## Results

### Structured search for genetic variants

The search produced 588 records of which 516 were excluded following review of title and abstract (Supplementary Figure S2). Details of the excluded studies and the reasons for exclusion are provided in Supplementary Table S2. 37 studies were selected from the main search for data extraction^8^, ^9^, ^10^^,^^25^, ^26^, ^27^, ^28^, ^29^, ^30^, ^31^, ^32^, ^33^, ^34^, ^35^, ^36^, ^37^, ^38^, ^39^, ^40^, ^41^, ^42^, ^43^, ^44^, ^45^, ^46^, ^47^, ^48^, ^49^, ^50^, ^51^, ^52^, ^53^, ^54^, ^55^, ^56^, ^57^, ^58^ and an additional 7 were found from references of meta-analyses or registries.^59^, ^60^, ^61^, ^62^, ^63^, ^64^, ^65^ Study characteristics for the discovery analyses and supporting studies are provided in Supplementary Tables S3 and S4.

Thirty of these studies were GWAS for SNPs of which 13 were associations studies with AF phenotypes, 3 for LA indices and 14 for PR indices. One of these studies did not report any significant variants.^51^ There was only one study that was a GWAS for CNV looking at participants with severe AF phenotypes. Four were ExWAS studies of which 2 were exploring AF associations and 2 for PR indices. All WGS studies of which there were 5 were exploring AF associations. Two of these also reported on indels.

Eight studies provided additional analyses looking at the influence of variants on expression levels either by TWAS or eQTL analyses. One additional study provided an eQTL colocalization analysis based on previously identified GWAS variants. RVAS was performed additionally by 5 studies. There were 4 additional replication studies of which one did not report the association data so could not be used.

The two oldest studies reported significance levels below the contemporary accepted genome wide significance threshold of 5.0 × 10^−8^, however the targets identified from these were replicated in other studies.

### Targets with potential repurposable drugs or drug development opportunities

From reviewing the selected studies, we identified 474 annotated genes from GWAS analysis, 22 from WGS analysis, 170 from ExWAS analysis, one from a CNV GWAS and 11 from replication studies (Supplementary Table S5). In addition, we discovered 564 from TWAS analyses or eQTL co-localisation (Supplementary Table S6) and 17 from RVAS analyses (Supplementary Table S7). The list of unique annotated genes identified that associated with AF or AF traits amounted to a total of 1043, and the frequency of reports in each analysis with prioritisation score is provided in Supplementary Table S8.

Of this list, we identified 68 which had interactions with drugs either in development or that already been approved for sale (Table 1). We found 31 targets which had both a small molecule druggability score and a gene prioritisation score of more than 1 and 83 targets which had an antibody and gene prioritisation score of more than 1 (Supplementary Tables S9 and S10). Interestingly 12 targets which were associated with drugs did not appear in the list of druggable genes (*FDPS, TNNT3, AOPEP, NDUFAF3, MET, RPS2, RAF1, WT1, RPL32, MYH7, MYH6, POLR2A*).

**Table 1:** Targets with associated drugs, details of subcellular location, target class and evidence from expression level analyses.

Target	Approved name	Location	Target class	TWAS	eQTL
ACVR2B	activin A receptor type 2B	Cell membrane	Enzyme	–	–
ADRA1A	adrenoceptor alpha 1A	Nucleus membrane, Cell membrane, Membrane	Membrane receptor	–	–
ADRB1	adrenoceptor beta 1	Cell membrane, Early endosome	Membrane receptor	–	–
AOPEP	aminopeptidase O (putative)	Nucleus, [Isoform 3]: Cytoplasm	Enzyme	RA	RA, LV
BLK	BLK proto-oncogene, Src family tyrosine kinase	Cell membrane	Enzyme	–	–
CA4	carbonic anhydrase 4	Cell membrane	Enzyme	–	–
CACNA1G	calcium voltage-gated channel subunit alpha1 G	Cell membrane, Cytoplasm	Ion channel	–	–
CDC7	cell division cycle 7	Nucleus	Enzyme, Other cytosolic protein	–	LV
CHRM2	cholinergic receptor muscarinic 2	Cell membrane, Postsynaptic cell membrane	Membrane receptor	LV	LV
ENPEP	glutamyl aminopeptidase	Cell membrane	Enzyme	–	–
EPAS1	endothelial PAS domain protein 1	Nucleus, Nucleus speckle	Transcription factor	–	–
EPHA3	EPH receptor A3	[Isoform 1]: Cell membrane, [Isoform 2]: Secreted	Enzyme	–	–
ERBB2	erb-b2 receptor tyrosine kinase 2	Cell membrane, Cell projection, [Isoform 1]: Cell membrane, Early endosome, Cytoplasm, Nucleus, [Isoform 2]: Cytoplasm, [Isoform 3]: Cytoplasm	Enzyme	RA, LV	–
ERBB4	erb-b2 receptor tyrosine kinase 4	Cell membrane, [ERBB4 intracellular domain]: Nucleus, Mitochondrion	Enzyme	–	–
ESR2	estrogen receptor 2	Nucleus	Transcription factor	RA	–
FDFT1	farnesyl-diphosphate farnesyltransferase 1	Endoplasmic reticulum membrane	Enzyme	LV	–
FGFR1	fibroblast growth factor receptor 1	Cell membrane, Nucleus, Cytoplasm, Cytoplasmic vesicle	Enzyme	–	–
FGFR2	fibroblast growth factor receptor 2	Cell membrane, Golgi apparatus, Cytoplasmic vesicle, [Isoform 1]: Cell membrane, [Isoform 3]: Cell membrane, [Isoform 8]: Secreted, [Isoform 13]: Secreted	Enzyme, Secreted protein	–	–
HBEGF	heparin binding EGF like growth factor	[Heparin-binding EGF-like growth factor]: Secreted, [Proheparin-binding EGF-like growth factor]: Cell membrane	Secreted protein	–	–
HCN4	hyperpolarization activated cyclic nucleotide gated potassium channel 4	Cell membrane	Ion channel	–	–
IGF1R	insulin like growth factor 1 receptor	Cell membrane	Enzyme	LV	LV
IL6R	interleukin 6 receptor	[Isoform 1]: Cell membrane, [Isoform 2]: Secreted, [Soluble interleukin-6 receptor subunit alpha]: Secreted	Membrane receptor, Secreted protein	RA	–
IMPDH1	inosine monophosphate dehydrogenase 1	Cytoplasm, Nucleus	Enzyme	–	–
ITGA2B	integrin subunit alpha 2b	Membrane	Membrane receptor	–	–
ITGB1	integrin subunit beta 1	Cell membrane, Cell projection, Melanosome, Cleavage furrow, Cell junction, Cell surface, [Isoform 5]: Cell membrane	Membrane receptor	RA	–
KCND3	potassium voltage-gated channel subfamily D member 3	Cell membrane, Cell projection	Ion channel	RA, LV	–
KCNH2	potassium voltage-gated channel subfamily H member 2	Cell membrane	Ion channel, Membrane receptor	–	–
KCNJ2	potassium inwardly rectifying channel subfamily J member 2	Membrane	Ion channel	–	–
KCNJ5	potassium inwardly rectifying channel subfamily J member 5	Membrane	Ion channel	RA, LV	RA, LA, LV
LAMB2	laminin subunit beta 2	Secreted	Structural protein	–	–
MAPT	microtubule associated protein tau	Cytoplasm, Cell membrane, Cell projection	Other cytosolic protein	RA, LV	RA, LV
MC4R	melanocortin 4 receptor	Cell membrane	Membrane receptor	–	–
MET	MET proto-oncogene, receptor tyrosine kinase	Membrane, [Isoform 3]: Secreted	Enzyme	–	–
MTNR1A	melatonin receptor 1A	Cell membrane	Membrane receptor	–	–
MYH6	myosin heavy chain 6	Cytoplasm	Unclassified protein	–	–
MYH7	myosin heavy chain 7	Cytoplasm	Unclassified protein	–	–
MYL4	myosin light chain 4	Cell membrane	Unclassified protein	–	–
NDUFAF3	NADH:ubiquinone oxidoreductase complex assembly factor 3	Nucleus, Mitochondrion inner membrane	Enzyme	–	RA
NDUFB10	NADH:ubiquinone oxidoreductase subunit B10	Mitochondrion inner membrane	Enzyme	RA, LV	–
NPR3	natriuretic peptide receptor 3	Cell membrane	Membrane receptor, Enzyme	–	RA
NR3C1	nuclear receptor subfamily 3 group C member 1	[Isoform Alpha]: Cytoplasm, Nucleus, Mitochondrion, Cytoplasm, [Isoform Beta]: Nucleus, [Isoform Alpha-B]: Nucleus	Transcription factor	LV	–
PDE3A	phosphodiesterase 3A	Membrane, Cytoplasm	Enzyme	–	–
PDE4B	phosphodiesterase 4B	[Isoform PDE4B5]: Cytoplasm, Cell membrane	Enzyme	–	–
POLR2A	RNA polymerase II subunit A	Nucleus, Cytoplasm, Chromosome	Enzyme	–	–
PRKCA	protein kinase C alpha	Cytoplasm, Cell membrane, Mitochondrion membrane	Enzyme	–	RA, LV
PSMB7	proteasome 20S subunit beta 7	Cytoplasm, Nucleus	Enzyme, Unclassified protein	RA, LV	–
PSMD3	proteasome 26S subunit, non-ATPase 3	Nuceloplasm	Enzyme	LV	–
PTK2	protein tyrosine kinase 2	Cell junction, Cell membrane, Cytoplasm, Nucleus	Enzyme	–	–
RAF1	Raf-1 proto-oncogene, serine/threonine kinase	Cytoplasm, Cell membrane, Mitochondrion, Nucleus	Enzyme	–	–
RPL32	ribosomal protein L32	Cytoplasm	Unclassified protein	–	–
RPS2	ribosomal protein S2	Cytoplasm, Nucleus	Unclassified protein	–	–
RPSA	ribosomal protein SA	Cell membrane, Cytoplasm, Nucleus	Unclassified protein	LV	RA
SCN10A	sodium voltage-gated channel alpha subunit 10	Cell membrane	Ion channel	RA, LV	LA
SCN5A	sodium voltage-gated channel alpha subunit 5	Cell membrane, Cytoplasm	Auxiliary transport protein, Ion channel	RA	LA
SIRT1	sirtuin 1	Nucleus, Cytoplasm, [SirtT1 75 kDa fragment]: Cytoplasm, Mitochondrion	Epigenetic regulator	RA, LV	–
SLAMF7	SLAM family member 7	Membrane	Membrane receptor	–	–
SLC6A4	solute carrier family 6 member 4	Cell membrane, Endomembrane system, Synapse, Cell junction	Transporter	–	–
SMAD7	SMAD family member 7	Nucleus, Cytoplasm	Unclassified protein	–	–
SRD5A3	steroid 5 alpha-reductase 3	Endoplasmic reticulum membrane	Enzyme	–	–
SYK	spleen associated tyrosine kinase	Cell membrane, Cytoplasm	Enzyme	–	–
THRB	thyroid hormone receptor beta	Nucleus	Transcription factor	RA, LV	RA, LV
TNFSF12	TNF superfamily member 12	Cell membrane, [Tumor necrosis factor ligand superfamily member 12, secreted form]: Secreted, [Isoform TWE-PRIL]: Cell membrane	Secreted protein	–	–
TNFSF13	TNF superfamily member 13	Secreted	Secreted protein	–	–
TNNI3	troponin I3, cardiac type	Cytoplasm	Unclassified protein	–	–
TNNT3	troponin T3, fast skeletal type	Sarcoplasmic Reticulum	Unclassified protein	LV	–
TUBB3	tubulin beta 3 class III	Cytoplasm, Cell projection	Structural protein	–	–
WT1	WT1 transcription factor	Nucleus, Cytoplasm, [Isoform 1]: Nucleus speckle, [Isoform 4]: Nucleus	Unclassified protein	–	–
XPO1	exportin 1	Cytoplasm, Nucleus	Unclassified protein	–	–

Legend: TWAS, Transcriptome-Wide Association Study; eQTL, expression Quantitative Trait Loci colocalization; RA, Right Atrium; LA, Left Atrium; LV, Left Ventricle.

### Characterization of targets

In total there were 613 unique drug compounds associated with the 68 targets after duplicates with different salts or conjugates were removed. Supplementary Table S11 outlines which drugs were associated with each target and other cardiovascular traits or outcomes found to have genomic association with each one. Of these targets, 37 were associated with AF only 21 with PR indices only and only one with LA indices. No drugged targets were associated with all three association types although 8 had associations with both AF and PR indices and only 2 with both AF and LA indices. Supplementary Table S12 details the amount of times each drugged gene was reported in a discovery analysis (based on the variant to gene annotation), expression level analysis (eQTL or TWAS) for each tissue and RVAS analysis. The targets are ordered by our prioritisation score.

Of the druggable targets 31 (*ADRB1, ENPEP, EPHA3, FGFR1, HBEGF, HCN4, IL6R, KCNH2, KCNJ5, MAPT, MC4R, MET, MYH6, MYL4, NDUFAF3, NDUFB10, NPR3, PDE3A, PDE4B, PRKCA, PSMB7, PTK2, SCN5A, SRD5A3, THRB, TNSFSF12, TNFSF13, TNNT3, TUBB3, WT1* and *XPO1*) were also associated with blood pressure and hypertension traits (Supplementary Table S11).

**Fig. 2:**
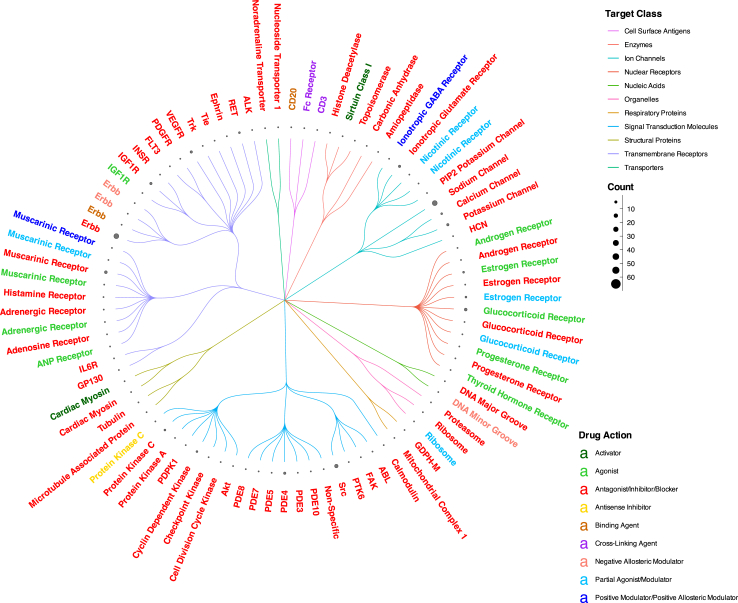
Dendrogram describing drug actions, targets and target classes for drugs which have a high prioritisation score.

**Fig. 3:**
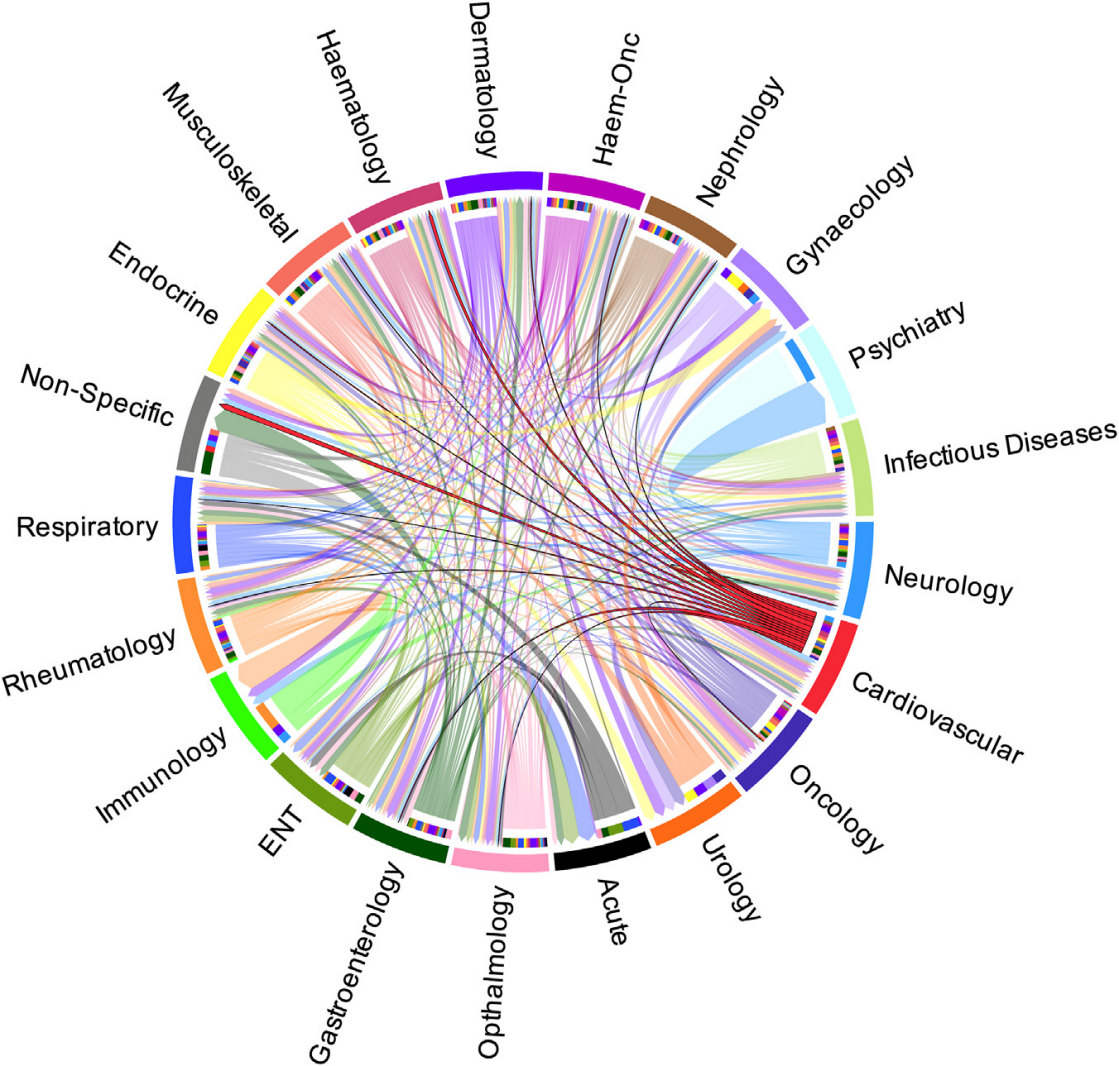
Chord diagram describing the cross-over of indications for drugs with a high prioritisation score. Each sector represents proportion of indications within the specialty.

Fig. 2 details the action types of the drugs with a high prioritisation score as well as the target proteins they act on. Of these 288 drugs, 194 (67%) are inhibitors and they include two classes of drugs currently used in the management of AF; beta blockers and ion channel blockers. The chord diagram (Fig. 3) demonstrates the high degree of crossover between indications for these drugs as divided into broad clinical categories.

### Genetic evidence supports existing drug classes used for AF management

After 1226 structured searches on MEDLINE and Clinicaltrials.gov, among the 613 identified drugs there were 70 drugs with study evidence of AF outcome. Details on their effects, maximum level of evidence, drug warnings and priority are given on Table 2. Of these there were 40 cardiovascular drugs with clinical data on their AF-related effects in humans (Supplementary Table S13). Controlled evidence on AF outcomes was available for 7 drugs from systematic reviews and 17 from RCTs. Details on effect estimates, patient populations, sample sizes and quality of evidence are presented in Supplementary Tables S14, S15 and S16. 21 drugs were included in guidelines on AF management from Europe, America or Japan.^23^^,^^24^^,^^66^ These included the beta-blockers bisoprolol, atenolol, carvedilol, esmolol, landiolol, metoprolol, nadolol, nebivolol, propranolol and sotalol. There was also systematic review evidence for 2 other drugs in this class, acebutolol and timolol from a Cochrane systematic review looking at prophylaxis for AF or SVT post cardiac surgery. Both drugs demonstrated a reduced relative risk of incident AF when compared with placebo (or no intervention).^67^

**Table 2:** Drugs with clinical evidence, associated targets and serious adverse effects.

Drugs	Symbol	Maximum Level Of Evidence	Overall Effect	Black Box Warning	Withdrawn
Amiodarone	KCNH2	Guideline	Existing treatment	Pulmonary Toxicity, Hepatotoxicity, Cardiotoxicity	No
Atenolol	ADRB1	Guideline	Existing treatment	Cardiotoxicity	No
Bepridil	CACNA1G	Guideline	Existing treatment	··	No
Bisoprolol	ADRB1	Guideline	Existing treatment	··	No
Carvedilol	ADRA1A, ADRB1	Guideline	Existing treatment	··	No
**Disopyramide**	**SCN10A, SCN5A**	**Guideline**	**Existing treatment**	**Cardiotoxicity**	**No**
Dofetilide	KCNH2	Guideline	Existing treatment	Cardiotoxicity	No
**Dronedarone**	**HCN4, KCNJ2, SCN10A, SCN5A**	**Guideline**	**Existing treatment**	**Mortaility Risk**	**No**
Esmolol	ADRB1	Guideline	Existing treatment	··	No
**Flecainide**	**SCN5A**	**Guideline**	**Existing treatment**	**Cardiotoxicity**	**No**
Ibutilide	KCNH2	Guideline	Existing treatment	Cardiotoxicity	No
Landiolol	ADRB1	Guideline	Existing treatment	··	No
Metoprolol	ADRB1	Guideline	Existing treatment	··	No
Nadolol	ADRB1	Guideline	Existing treatment	Cardiotoxicity	No
Nebivolol	ADRB1	Guideline	Existing treatment	··	No
**Procainamide**	**SCN10A, SCN5A**	**Guideline**	**Existing treatment**	**Musculoskeletatl Toxicity, Haematological Toxicity, Cardiotoxicity**	**No**
**Propafenone**	**ADRB1, SCN10A, SCN5A**	**Guideline**	**Existing treatment**	**Mortaility Risk**	**No**
Propranolol	ADRB1	Guideline	Existing treatment	Cardiotoxicity	No
**Quinidine**	**SCN10A, SCN5A**	**Guideline**	**Existing treatment**	**Cardiotoxicity**	**No**
**Sotalol**	**ADRB1, KCNH2**	**Guideline**	**Existing treatment**	**Cardiotoxicity**	**No**
**Vernakalant**	**KCND3, KCNH2, KCNJ5, SCN5A**	**Guideline**	**Existing treatment**	**··**	**No**
Colchicine	TUBB3	Systematic Review	Beneficial	··	No
**Methylprednisolone**	**NR3C1**	**Systematic Review**	**Beneficial**	**··**	**No**
Acebutolol	ADRB1	Systematic Review	Beneficial	··	No
**Dexamethasone**	**NR3C1**	**Systematic Review**	**Beneficial**	**··**	**No**
**Hydrocortisone**	**NR3C1**	**Systematic Review**	**Neutral**	**··**	**No**
**Ivabradine**	**HCN4**	**Systematic Review**	**Harmful**	**··**	**No**
**Liothyronine**	**THRB**	**Systematic review**	**Neutral**	**Against use in weight loss**	**No**
**Mavacamtam**	**MYH6, MYH7, MYL4**	**Systematic Review**	**Neutral**	**··**	**No**
**Roflumilast**	**PDE4B**	**Systematic Review**	**Beneficial**	**··**	**No**
Timolol	ADRB1	Systematic Review	Beneficial	··	No
Prednisolone	NR3C1	RCT	Neutral	··	No
**AZD1305**	**KCNH2, SCN5A**	**RCT**	**Beneficial**	**··**	**No**
**AZD7009**	**KCNH2, SCN5A**	**RCT**	**Beneficial**	**··**	**No**
**Beclomethasone Dipropionate**	**NR3C1**	**RCT**	**Beneficial**	**··**	**No**
Betaxolol	ADRB1	RCT	Neutral (Compared with other drug in same class)	··	No
Bucindolol	ADRA1A, ADRB1	RCT	Beneficial (Compared with other drug in same class)	··	No
**Carperitide**	**NPR3**	**RCT**	**Beneficial**		**No**
Cilostazol	PDE3A	RCT	Harmful	Contraindicated In Heart Failure	No
**Conjugated Estrogens**	**ESR2**	**RCT**	**Neutral**	**Endometrial cancer risk, Cariovascular risk, Dementia risk**	**No**
Dobutamine	ADRB1	RCT	Neutral (Compared with other drug in same class)	··	No
Doxazosin	ADRA1A	RCT	Harmful	··	No
Duloxetine	SLC6A4	RCT	Neutral	Psychiatric Toxicity	No
**Erlosamide**	**SCN10A, SCN5A**	**RCT**	**Neutral**		**No**
Isoproterenol	ADRB1	RCT	Neutral (Compared with other drug in same class)	··	No
Levosimendan	PDE3A, TNNI3	RCT	Mixed	··	No
**Lidocaine**	**SCN10A, SCN5A**	**RCT**	**Beneficial**	**For strict dosing under the age of 3 otherwise mortaility risk**	**No**
**Meprednisone**	**NR3C1**	**RCT**	**Neutral**	**··**	**No**
Milrinone	PDE3A	RCT	Beneficial	··	No
Nomifensine	SLC6A4	RCT	Neutral	··	Yes
**Omecamtiv Mecarbil**	**MYH6, MYH7, MYL4**	**RCT**	**Neutral**	**··**	**No**
**Prednisone**	**NR3C1**	**RCT**	**Neutral**	**··**	**No**
**Ranolazine**	**SCN5A**	**RCT**	**Beneficial**	**··**	**No**
**Tedisamil**	**KCND3, KCNH2**	**RCT**	**Beneficial**	**··**	**No**
**Theophylline**	**PDE3A, PDE4B**	**RCT**	**Neutral**	**··**	**No**
**Tosedostat**	**AOPEP (C9orf3), ENPEP**	**RCT**	**Harmful**	**··**	**No**
Vinorelbine	TUBB3	RCT	Harmful	Haematological Toxicity, Infectious Disease	No
**Bortezomib**	**PSMB7, PSMD3**	**Observational**	**Potential Harm**	**··**	**No**
Abiraterone	SRD5A3	Observational	Potential Harm	··	No
**Carfilzomib**	**PSMB7, PSMD3**	**Observational**	**Potential Harm**	**··**	**No**
**Cocaine**	**SCN10A, SCN5A**	**Observational**	**Potential Harm**	**Misuse**	**No**
Docetaxel	TUBB3	Observational	Potential Harm	Gastrointestinal Toxicity, Haematological Toxicity, Respiratory Toxicity, Dermatological Toxicity, Vascular Toxicity, Immune System Toxicity, Hepatotoxicity	No
Elotuzumab	SLAMF7	Observational	Potential Harm	··	No
**Ixazomib**	**PSMB7, PSMD3**	**Observational**	**Potential Harm**	**··**	**No**
**Levothyroxine**	**THRB**	**Observational**	**Potential Harm**	**Against use in weight loss**	**No**
**Metformin**	**NDUFAF3, NDUFB10**	**Observational**	**Mixed**	**··**	**No**
Methamphetamine	SLC6A4	Observational	Potential Harm	··	Yes
**Midostaurin**	**PRKCA**	**Observational**	**Potential Harm**	**··**	**No**
**Raloxifene**	**ESR2**	**Observational**	**Neutral**	**Respiratory Toxicity, Vascular Toxicity, Neurotoxicity**	**No**
Regorafenib	FGFR1, FGFR2, RAF1	Observational	Potential Harm	Hepatotoxicity	No
Sorafenib	RAF1	Observational	Potential Harm	··	No
**Trastuzumab**	**ERBB2**	**Observational**	**Neutral**	**Teratogenicity, Cardiotoxicity, Respiratory Toxicity**	**No**

Legend: Bold text indicates high prioritisation score

Bepridil, a calcium channel blocker was recommended in the Japanese guidelines for management of AF. Other calcium channel blockers such as verapamil and diltiazem are also recommended in American and European guidelines however, they are not associated with any of the targets identified.

The sodium channel blockers disopyramide, propafenone, encainide, flecainide and quinidine are another class of drugs in the therapeutic armamentarium recommended by guidelines. Ranolazine which is purported to act as a sodium channel blocker also has RCT evidence of benefit for reducing AF burden in patients with paroxysmal AF when used in conjunction with dronedarone compared to placebo (59% reduction, P = 0.008).^68^ There is also evidence of reduced clinical AF events post non-ST-elevation myocardial infarction (hazard ratio: 0.71, 0.55–0.92, p = 0.01).^69^

Drugs classed as potassium channel blockers were amiodarone, vernakalant, ibutilide, dronedarone, and dofetilide. Vernakalalant, amiodarone and dronedarone, also act as sodium channel blockers and there is RCT evidence for the discontinued drugs AZD1305 and AZD7009, which act on both channels, being superior to placebo in converting AF to sinus rhythm. Another discontinued potassium channel blocker, tedisamil, was found to be more effective than placebo for converting AF to sinus rhythm^70^, ^71^, ^72^ (Supplementary Tables S14 and S15).

It is worth noting that our prioritisation score placed the targets that these potassium and sodium channel blockers act on (*SCN5A, SCN10A, KCNJ5, KCND3*) in the top 7.

### Carperitide suggests protective effect of atrial natriuretic peptides

Carperitide is a recombinant atrial natriuretic peptide that is used in Japan for the treatment of heart failure. In two randomised trials of patients receiving cardiopulmonary bypass surgery, peri-operative carperitide infusion compared with placebo significantly reduced the incidence of post-operative AF.^73^^,^^74^ The target *NPR3* also has a prioritisation score of >1.

### Phosphodiesterase inhibitors show mixed effects on AF outcomes

The inotropic drugs levosimendan and milrinone act on phosphodiesterase 3. They have RCT evidence that demonstrates a protective effect on post-cardiac surgery AF risk vs. dobutamine (5% vs 18% for milrinone, P < 0.04, 5% vs 20% for levosimendan P = 0.044).^75^^,^^76^ This effect is also seen with levosimendan vs placebo (12% vs 5%, P < 0.05).^77^ However another trial of levosimendan vs dobutamine in patients with heart failure requiring inotropic support demonstrated an increased risk of incident AF (9.1% vs 6.1%, P = 0.02).^78^ In addition, another drug acting as an inhibitor of phosphodiesterase 3, cilostazol is associated with increased incidence of AF when used in conjunction with aspirin for patients with non-cardioembolic stroke compared with aspirin alone (5% vs 2%, P = 0.007). The reasons for this mixed evidence are unclear, however it could be attributable to the different patient populations the trials are in. Whilst *PDE3A* did not have a prioritisation score >1, another gene coding for phosphodiesterase 4 (*PDE4B*) did.

### Increased AF risk with doxazosin and ivabradine

A systematic review of 13 RCTs where patients were treated with ivabradine noted a significantly higher AF risk which was more pronounced if LVEF>40%.^79^ This drug acts on the potassium and sodium channel encoded by *HCN4*. Doxazosin an alpha adrenergic blocker compared with chlorthalidone, a thiazide-like diuretic had an increased risk of new onset AF from an RCT of patients being treated for hypertension.^80^ Whilst *HCN4* has a prioritisation score of >1 the target for doxazosin (*ADRA1A*) does not. Although these drugs do not constitute potential targets for repurposing, it is possible that an agent with the opposite action may provide a protective effect on incident AF especially in the case of *HCN4*.

### Some non-cardiovascular drugs may be of benefit in specific clinical scenarios

The literature search returned 128 non-cardiovascular drugs that had entries on MEDLINE or Clinicaltrials.gov (Supplementary Table S17). None were included in current AF guidelines. Ten drugs had maximum level evidence from systematic reviews on potential AF-related effects, 9 from RCTs and 15 from observational data. Details on effect estimates, patient populations, sample sizes and quality of evidence are presented in Supplementary Tables S14, S15 and S16 as before.

Evidence supporting the use of the glucocorticoids; dexamethasone, methylprednisolone come from a systematic review of RCTs for patients undergoing cardiac surgery. There was a reduced risk of new onset AF for both drugs from 4 to 8 trials respectively, however evidence from 2 RCTs for hydrocortisone did not demonstrate a significant difference.^81^ The target *NRC31* also had a prioritisation score of >1, however it is important to note that this review did not include GWAS of post cardiac surgery patients, although there may be some overlap with the underlying disease process.

Two drugs used for patients with symptomatic chronic obstructive pulmonary disease (COPD) have evidence suggesting a protective effect on the development of AF. An RCT comparing a combination inhaler containing beclomethasone, formoterol and glycopyrronium was compared with another combination inhaler without inhaled corticosteroid (indacterol and glycopyrronium). There was a significant reduction in the rate of incident AF.^82^ Roflumilast is phosphodiesterase 4 inhibitor used in the treatment of COPD. It is encoded by the gene *PDE4B* which also has a prioritisation score >1. A systematic review of 14 RCTs from the COPD Safety pool dataset demonstrate a reduced incidence rate of AF when comparing roflumilast with placebo.^83^

### Mixed evidence for agents acting on tubulin

A systematic review of 6 RCTs for the use of peri-operative colchicine for patients undergoing cardiac surgery demonstrated a reduced incidence of AF compared with placebo. The same review analysed 2 RCTs looking at peri-procedural colchicine vs placebo to prevent AF recurrence after catheter ablation. It demonstrated a reduced risk of recurrence over a 12-month period.^84^ Whilst not in the same class of drugs, vinorelbine is a chemotherapeutic agent that acts by the inhibition of tubulin which is encoded by the same target *TUBB3*. An RCT comparing vinorelbine combined with gemcitabine vs gemcitabine alone in patients with stage III or IV non-small cell lung cancer demonstrated a higher rate of incident AF.^85^

### A new chemotherapeutic agent increases AF risk

Tosedostat is an aminopeptidase inhibitor used for the treatment of acute myeloid leukaemia (AML). In a phase II trial vs standard chemotherapy in AML or high risk myelodysplastic syndrome it was associated with significantly more AF events in the first cycle although the difference was not apparent in the second cycle.^86^

### Observational evidence for targets with high prioritisation scores

To exercise caution on drawing inferences from observational evidence we decided to focus on targets which had a prioritisation score of >1. Evidence suggestive of benefit or harm was available for drugs associated with two of these targets: *PRKCA* and *PSMB7*.

Midostaurin is a multiple kinase inhibitor used in the treatment of AML. One of its targets is protein kinase C alpha encoded by *PRKCA*. Data from the World Health Organisation (WHO) pharmacovigilance database, Vigibase, was used to perform a disproportionality analysis of reports of AF events. The adjusted reporting odds ratio (aROR) for midostaurin was 3.76 (95% CI 1.50–9.39).^87^ The same study described an increased aROR of 1.41 (95% CI 1.15–1.74) for the proteasome inhibitor bortezomib, used in treatment for multiple myeloma, which is associated with the target gene *PSMB7*. Similar results are reported for the other proteasome inhibitors ixazomib and bortezomib from disproportionality analyses from other pharmacovigilance databases.^88^^,^^89^

### Characteristics of prioritised targets with evidence

We have selected targets which had a high prioritisation score (or were in the same class as those that did) and evidence of effect on AF outcomes for further characterisation. We extracted functional description, subcellular location and pathway information from OpenTargets and have provided them in Supplementary Table S18.

## Discussion

**Fig. 4:**
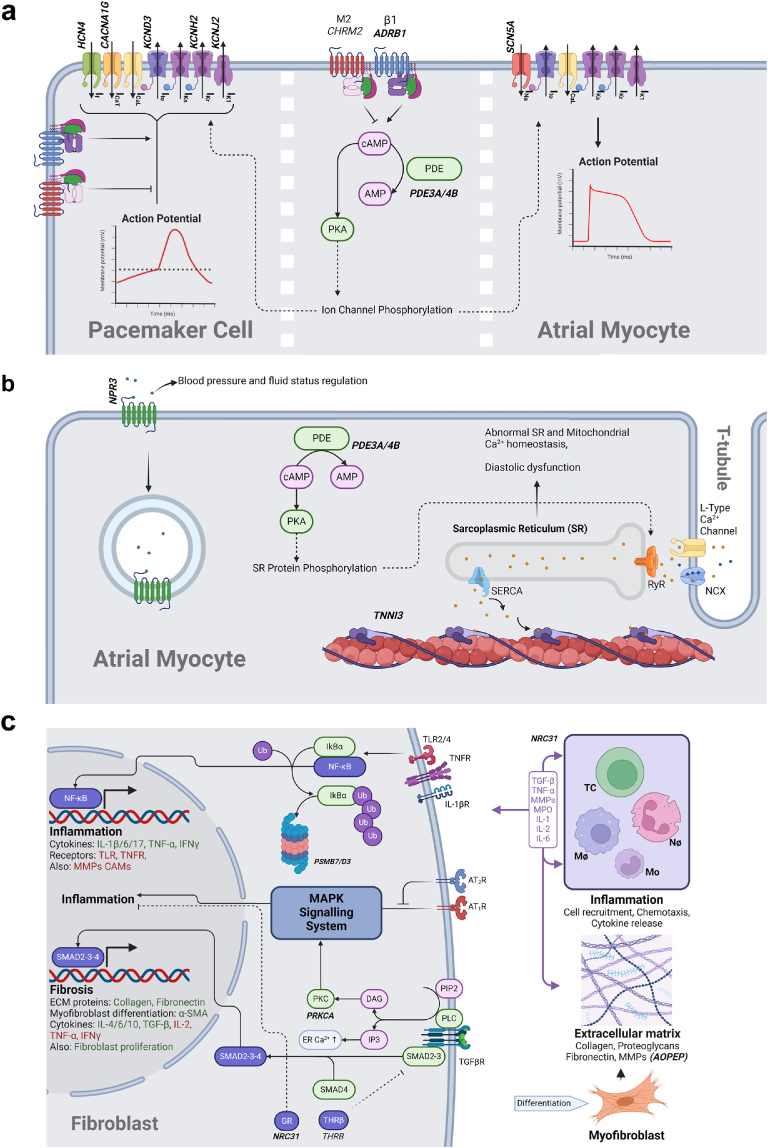
Panel a—Targets influencing electrophysiological functions in atrial myocytes and sino-atrial node. Panel b—Targets influencing mechanisms involved with contractility, calcium handling by sarcoplasmic reticulum and myofibrils and blood pressure/fluid status regulation. Panel c—Targets influencing some pathways involved with atrial fibrosis and inflammation. Key: Italics, drugged target not prioritised, Italics and Bold, drugged target prioritised; Green text, upregulation; Red text, downregulation; Green box, signalling protein; Purple box, Transcription factor/Nuclear receptor; Pink box, second messenger; Dotted line, possible mechanism in AF; Faded line, via intermediate signalling; Tapered line, production or secretion; DAG, Diacylglycerol; IP3, Inositol 1,4,5-trisphosphate; PLC, Phospholipase C; PIP2, Phosphatidylinositol 4; 5-bisphosphate; PKC, Protein kinase C; TGF-β(R), Transforming Growth Factor Beta (receptor); SMAD, Small Mothers Against Decapentaplegic; THRβ, Thyroid Hormone Receptor Beta; GR, Glucocorticoid Receptor; ER, Endoplasmic Reticulum; IL–Interleukin; TNF-α, Tumour Necrosis Factor Alpha; IFN-γ, Interferon Gamma; α-SMA, Alpha Smooth Muscle Actin; MAPK, Mitogen-Activated Protein Kinase; AT_1/2_R, Angiotensin 1/2 receptor; TLR, Toll-Like Receptor; TNFR, Tumour Necrosis Factor Receptor; MMP, Matrix Metalloproteinase; CAM, Cell Adhesion Molecule; TC, T cell; Mo–Monocyte; Mø, Macrophage; Nø, Neutrophil; MPO, Myeloperoxidase; PDE, Phosphodiesterase; (c)AMP, (cyclic) Adenosine Monophosphate; PKA, Protein Kinase A; NF-κB, Nuclear Factor Kappa-light-chain-enhancer of activated B cells; IκBα, nuclear factor of kappa light polypeptide gene enhancer in B-cells inhibitor, alpha; SERCA, Sarcoplasmic Reticulum Calcium ATPase; RyR, Ryanodine Receptor; NCX, Sodium Calcium Exchanger. Created with BioRender.com.

We presented results of an approach combining evidence synthesis with bioinformatics to identify targets for further evaluation into drug development and repurposing opportunities. This approach seems to be robust and valid as it has highlighted drugs which are already in use and recommended for AF management through guidelines.^23^^,^^24^^,^^66^ These drug classes act on adrenergic β1/α1 receptors and calcium, sodium and potassium channels (encoded by *ADRA1A, ADRB1, CACNA1G, SCN10A/5A*, and *KCND3/H2/J2/J5* respectively). The mechanisms by which these targets influence cardiac myoctes from an electrophysiological perspective are outlined in Fig. 4, Panel a.

However, we noted that in addition to this carperitide may have a protective effect on preventing post-operative AF in patients going for cardiac surgery. Natriuretic peptide receptor C encoded by *NPR3*, is an atrial natriuretic peptide receptor that binds all isoforms. Whilst the other natriuretic peptide receptors activate the intracellular cyclic guanosine monophosphate pathway, it functions as a method of clearing these peptides by internalisation and lysosomal degradation^90^ (Fig. 4, Panel b). Therefore, the mechanism by which carperitide may affect the development of AF through this receptor is not likely to be due to its effect on downstream cellular processes in cardiac tissue but by the mediation of circulating natriuretic peptides.

The phosphodiesterase inhibitors milrinone, levosimendan, clilostazol (*PDE3A*) and roflumilast (*PDE4B*) have mixed evidence on whether they are protective or harmful with respect to AF development. PDE3A regulates cardiac myocyte contractility via downstream cyclic adenosine monophosphate signalling on L-type calcium channels and the sarcoplasmic reticulum calcium ATPase (SERCA) and PDE4B upregulates ryanodine receptor activity via protein kinase A (PKA) and subsequent SERCA mediated calcium uptake into the sarcoplasmic reticulum from the cytosol.^91^ Phosphodiesterase activity has been found in animal studies to regulate electrophysiological properties in the sino-atrial node and pulmonary veins which are known to modulate arrhythmogenic activity in AF.^92^ Levosimendan also acts via binding the troponin C–I complex increase the calcium sensitivity of the myofilaments.^93^ This has been shown to be implicated in the pathogenesis of dilated cardiomyopathy through a myopathic process that may affect the atria directly or via diastolic dysfunction.^94^ It is also possible this intracellular calcium leak may be a mechanism for AF development^95^ (Fig. 4, Panel b).

The increased risk of AF from ivabradine use is through the antagonism of the HCN4 channel. It is highly expressed in the sino-atrial node and is an important mediator of the pacemaker action potential. A novel variant associated with AF discovered from targeted sequencing of the *HCN4* coding region was replicated in Chinese Hamster Ovary cells and analyses with immunocytochemistry and confocal microscopy. It was found not to traffic to the cell membrane whilst the wild types did.^96^ It is possible therefore that reduced expression of HCN4 may play a role in the development of AF.

There is evidence from systematic reviews and an RCT suggesting a protective effect of glucocorticoids on AF in cardiac surgery and patients with COPD. Steroids have a wide range of systemic effects and in particular they influence inflammation by binding pro-inflammatory transcription factors such as nuclear factor κB.^97^ Inflammation has been reported to play a role in AF pathogenesis through contributing to electrical and structural remodelling via promoting irregularities in pulmonary vein calcium signalling and cardiac fibrosis through the angiotensin II and transforming growth factor beta 1 pathways.^98^ However, there are also many other systemic side effects such as hyperglycaemia and increased risk of infection and long term use of steroids has been related to increased AF risk.^99^ Furthermore, although there is some overlap between contributory factors for incident AF and post-operative AF, especially when considering pre-existing atrial substrate, there is also a significant component from the surgical modification of the atrium and the transient post-operative phase. Factors such as exacerbation of the autonomic nervous system, cytokine release mediating inflammation and oxidative stress promote triggered activity in the latter transient phase. The benefit derived from glucocorticoids in this context is likely related to action on this inflammatory cytokine release.^100^

Some drugs used in the treatment of haematological malignancies may increase the risk of AF. Tosedostat is an aminopeptidase inhibitor, particularly of the M1 class. It is a new drug used in the treatment of AML. Aminopeptidases belong to the class of enzymes known as matrix metalloproteinases. Whilst there is a lack of evidence on the targets associated with this drug (*AOPEP, ENPEP*) in AF pathology, high circulating matrix metalloproteinase 9 was found in patients with AF as well as increased expression in atrial tissue.^101^^,^^102^

Midostaurin is also a drug used for the treatment of AML. It is a non-selective protein kinase inhibitor acting on protein kinase C alpha. There is observational data from a pharmacovigilance study suggesting an increased risk of AF, however this is risk is also present in the patient population.^87^^,^^103^

Further evidence from pharmacovigilance studies is reported for the proteasome inhibitors, bortezomib, carfilzomib and ixazomib. We have highlighted these drugs as the target *PSMB7* had a high prioritisation score. A mouse study has demonstrated a potential role for the proteasome in AF pathogenesis, possibly via actions on the nuclear factor κB pathway^104^ (Fig. 4, Panel c). However, these drugs are used in the treatment of multiple myeloma and this patient population has a high risk of incident AF.^103^

Due to the low quality of evidence for the role of these targets involved with haematological malignancies, further investigation is necessary before consideration for drug development.

Of the genes associated with AF or AF-related traits, only 68 (8%) interact with approved drugs, or drugs under development. We have provided tables detailing the targets deemed druggable for small molecule drugs and biologic agents that have a prioritisation score of >1 (Supplementary Tables S9 and S10). This list of targets may be a starting point for future pre-clinical validation approaches to aid drug development.

We would like to emphasise that our findings do not represent drugs that are immediately ready for repurposing. Further work is still needed to validate the use of these drugs in the management of AF. For those where a significant benefit is presented in RCTs or systematic reviews, if there is clinical equipoise for the current indication of the drugs with other treatments, an RCT for that relevant patient population with incident AF as a specific outcome may help confirm the potential use of that drug in those patients to reduce the risk of AF.

However, in the absence of good quality clinical evidence we propose this pipeline for further validation of targets. For targets which have no drugs currently available or only observational data available for drugs, initial analysis looking at the effect of knock down variants on AF outcomes (and adverse effects) such as through mendelian randomisation may add confidence to repurposing or drug development opportunities. However, we appreciate that there are often key unmeasured confounding variables in this approach, such as the effect of the knockdown on embryological development. Therefore, other avenues may involve exploring the effect of knockdown targets with in vitro cell culture studies or if compounds are available, their effect on wild type targets. Positive signals from these approaches may reinforce potential suitability for clinical trial validation.

At the same time exploratory observational analyses of electronic healthcare records of drugs which have no clinical evidence of AF outcomes may aid in hypothesis generation and identifying further compounds for repurposing. However, once more, there are several caveats to this. Aside from common biases due to (residual) confounding, these types of studies are particularly vulnerable to bias from missing data (i.e. missingness due to missing at random or missing not at random),^105^ immortal time bias, and bias due to measurement/coding error. Due to this any analysis performed would need to be highly rigorous and despite that still may not reflect causality.

We did not set out sample size thresholds for inclusion of studies to exclude those with insufficient power to detect the effect sizes expected for complex genetic traits. Setting a threshold ex-ante would require a judgement on what a plausible effect size would be which is subject to variation between authors. Furthermore, factors other than sample size such as exposure frequency or population standard deviation in the context of quantitative traits may also affect accuracy. Instead, we employed our prioritisation score to confirm whether variants with plausible effect sizes were replicated across studies, as the probability of a null association decreases respective to the number of replications.

Whilst using the OpenTargets variant to gene pipeline may aid reproducibility of this method and help mitigate the uncertainty of relying on annotation by proximity to transcription site only, we appreciate that this still does not account for the influence of these variants in specific tissues and cell types we hope the inclusion of expression level analyses in out prioritisation score may help with this.

It is possible that some of the targets may have an influence on AF indirectly by signalling molecules up or downstream a given pathway. We appreciate that our approach does not take this into account. Future work can look at identifying these signalling molecules by referencing resources describing pathways such as reactome.^106^

We note that for some drugs such as amiodarone the available data did not find links to some targets known to be associated with it. Furthermore, the druggability data failed to highlight some targets which already have drugs targeting them. This is reflective of existing discrepancies in the data sources used as well as the infancy of methodologies used to predict druggability. Knowledge in this field continues to evolve, with the number of protein targets thought to be druggable resting currently at 4000 to 4500 (22% of the human proteome).^13^ Thus, some of the targets we identified that were not considered druggable, may in the future move to druggable territory. As further data becomes available exploring the genetic background of sub-phenotypes of AF, this process may aid in highlighting drugs which can be used to target these groups. Finally, knowledge on available drug effects and which protein a given drug interacts with is always expanding. The completeness and quality of annotation of datasets will also advance. Future versions of these analyses will provide more detailed mechanistic information on target-disease association. Our methodology has demonstrated clear feasibility in identification of possible candidates for drug repurposing and highlighting drugs and pathways which may potentiate AF disease processes. Further work to enhance this process will exploring potential targets up or downstream from identified targets and pre-clinical studies. Our approach relies on resources freely accessible to the public and can easily be applied to any other disease if associated genetic variants are known. We believe that transposing our methods to different diseases, affecting any organ system, may lead to an acceleration of progress in drug target identification and drug development across medicine.

## Contributors

Dr Kishore Kukendrarajah: Data Curation (review and verification of underlying data), Formal Analysis, Investigation, Visualisation, Writing–original draft, Writing–Review and Editing.

Prof Rui Providencia: Conceptualisation of study, Funding Acquisition, Methodology, Investigation, Data Curation (review and verification of underlying data), Formal Analysis, Project Administration, Supervision, Writing–Review & Editing.

Dr Amand Floriaan Schmidt: Supervision, Writing–Review & Editing.

Dr Aliki-Eleni Farmaki: Investigation, Data Curation, Formal Analysis.

Prof Pier D Lambiase: Supervision, Writing–Review & Editing.

Prof Richard Schilling: Supervision, Writing–Review & Editing.

Dr Chris Finan: Supervision, Writing–Review & Editing.

All authors have read and approved the final version of the manuscript.

Authors KK and RP reviewed and verified all underlying data, and authors KK, RP and AFS were responsible for the decision to submit the manuscript.

## Data sharing statement

All data are available to the public as public open-source databases were utilized. All our analyses and datasets are available as supplementary material.

## Declaration of interests

AFS and CF have received unrestricted funding from New Amsterdam Pharma, for investigating the role of a specific drug not related to AF. CF has received funding from Pfizer investigating the role of specific drug targets not related to AF, he is not the principal investigator on this. PL has received grants from Boston Scientific and Abbott Medical as well as consulting fees from Boston Scientific. RJS has received grants, consulting fees and honoraria from Abbott medical, Biosense Webster, Boston Scientific and Medtronic.
