# Supplementary material for: Advancing drug development for atrial fibrillation by prioritising findings from human genetic association studies
Source: eBioMedicine. 2024 Jun 27;105:105194. doi: 10.1016/j.ebiom.2024.105194 (PMC11260865; doi:10.1016/j.ebiom.2024.105194)
Supplement: Supplementary Tables [file mmc1.pdf]

Supplementary Information

Page 1 - Supplementary Table 1 - MEDLINE search for "Melatonin AND Atrial Fibrillation"

Page 3 - Supplementary Table 2 – Reasons for exclusion after full text review

Page 4 - Supplementary Table 3 – Characteristics of studies selected for discovery sources

Page 12 - Supplementary Table 4 – Characteristics of additional studies supporting discovery samples

Page 13 - Supplementary Table 5 - All targets from discovery analyses, annotated with variant to gene (annotation provided from paper if not available)

Page 39 - Supplementary Table 6 - All targets from analysis looking at the effect of discovery variants on expression levels of genes

Page 57 - Supplementary Table 7 - All targets from rare variant analyses

Page 58 - Supplementary Table 8 - Reports for targets in each analyses type and prioritisation score

Page 91 - Supplementary Table 9 - Prioritised targets with evidence of druggability for small molecules

Page 92 - Supplementary Table 10 - Prioritised targets with evidence of druggability for antibody drugs

Page 95 - Supplementary Table 11 – Drugs associated with targets and GWAS catalog information on other cardiovascular traits

Page 100 - Supplementary Table 12 - Reports for drugged targets in each analyses type and prioritisation score

Page 103 - Supplementary Table 13 – Clinical evidence on the available cardiovascular drugs targeting the identified hits

Page 107 - Supplementary Table 14 – Characteristics of randomised controlled trials (RCTs) providing evidence for AF outcomes on drugs linked to targets

Page 112 - Supplementary Table 15 – Risk of Bias (RoB) for randomised controlled trials (RCTs) providing evidence for AF outcomes on drugs linked to targets

Page 114 - Supplementary Table 16 – Characteristics of systematic reviews providing evidence for AF outcomes on drugs linked to targets

Page 117 - Supplementary Table 17 – Clinical evidence on the available non-cardiovascular drugs targeting the identified hits.

Page 122 - Supplementary Table 18 - Function description, subcellular locations and pathways for each prioritised target where drugs have good quality clinical evidence

Page 135 - Supplementary Figure 1 - Example search on ClinicalTrials.gov

Page 136 - Supplementary Figure 2 - PRISMA diagram

Page 137 - References

Supplementary Table 1 - MEDLINE search for "Melatonin AND Atrial Fibrillation"

PMID	Title	Authors	Citation	First Author	Journal/Book	Publication Year	Create Date	PMCID	DOI
36522127	Nutraceutical activation of Sirt1: a review	DiNicolantonio JJ, McCarty MF, O'Keefe JH.	Open Heart. 2022 Dec;9(2):e002171. doi: 10.1136/openhrt-2022-002171.	DiNicolantonio JJ	Open Heart	2022	15/12/2022	PMC9756291	10.1136/openhrt-2022-002171
37405359	Evaluating the Effects of Melatonin on the Oxidative Stress and Duration of Atrial Fibrillation following Coronary Artery Bypass Graft Surgery: A Randomised Controlled Trial	Dastan F, Barati S, Ahmadi ZH.	Eur Cardiol. 2023 Apr 25;18:e25. doi: 10.15420/ecr.2023.18.PO8. eCollection 2023.	Dastan F	Eur Cardiol	2023	05/07/2023	PMC10316341	10.15420/ecr.2023.18.PO8
32723264	The Effects of Melatonin on the Oxidative Stress and Duration of Atrial Fibrillation after Coronary Artery Bypass Graft Surgery: A Randomized Controlled Trial	Barati S, Jahangirifard A, Ahmadi ZH, Tavakoli-Ardakani M, Dastan F.	Endocr Metab Immune Disord Drug Targets. 2021;21(6):1142-1149. doi: 10.2174/1871530320666200728152307.	Barati S	Endocr Metab Immune Disord Drug Targets	2021	30/07/2020		10.2174/1871530320666200728152307
36639364	Melatonin increases susceptibility to atrial fibrillation in obesity via Akt signaling impairment in response to lipid overload	Qin X, Fu Y, Fan J, Liu B, Liu P, Zhang Y, Jiang T, Zheng Q.	J Pineal Res. 2023 Apr;74(3):e12851. doi: 10.1111/jpi.12851. Epub 2023 Jan 23.	Qin X	J Pineal Res	2023	13/01/2023		10.1111/jpi.12851
35710020	Melatonin inhibits angiotensin II-induced atrial fibrillation through preventing degradation of Ang II Type I Receptor-Associated Protein (ATRAP)	Xie X, Shen TT, Bi HL, Su ZL, Liao ZQ, Zhang Y, Shi L, Xia YL.	Biochem Pharmacol. 2022 Aug;202:115146. doi: 10.1016/j.bcp.2022.115146. Epub 2022 Jun 14.	Xie X	Biochem Pharmacol	2022	16/06/2022		10.1016/j.bcp.2022.115146
34864165	Activation of PKG-CREB-KLF15 by melatonin attenuates Angiotensin II-induced vulnerability to atrial fibrillation via enhancing branched-chain amino acids catabolism	Xu YL, Xu DY, Xue XD, Zhou ZJ, Huang YT, Zhao QS, Luo LY, Wang ZS, Wang HS.	Free Radic Biol Med. 2022 Jan;178:202-214. doi: 10.1016/j.freeradbiomed.2021.11.043. Epub 2021 Dec 2.	Yu LM	Free Radic Biol Med	2022	05/12/2021		10.1016/j.freeradbiomed.2021.11.043
29403578	The Use of Electronic Medical Record Data to Analyze the Association Between Atrial Fibrillation and Birth Month	Matsuda K, Park K, Tatsumi H, Kitada R, Yoshiyama M.	Online J Public Health Inform. 2017 Dec 31;9(3):e199. doi: 10.5210/ojphi.v9i3.7864. eCollection 2017.	Matsuda K	Online J Public Health Inform	2017	07/02/2018	PMC5790432	10.5210/ojphi.v9i3.7864
37075492	The circadian clock remains intact, but with dampened hormonal output in heart failure	Crnko S, Printezi MI, Zwetsloot PM, Leiteris L, Lumley AI, Zhang L, Ernens I, Jansen TPJ, Homsma L, Feyen D, van Faassen M, du Pré BC, Gaillard CAJM, Kemperman H, Oerlemans MIFJ, Doevendans PAFM, May AM, Zuithoff NPA, Sluijter JPG, Devaux Y, van Laake LW.	EBioMedicine. 2023 May;91:104556. doi: 10.1016/j.ebiom.2023.104556. Epub 2023 Apr 17.	Crnko S	EBioMedicine	2023	19/04/2023	PMC10131037	10.1016/j.ebiom.2023.104556
34856769	Circadian rhythms, cardiac arrhythmias and sudden death	Vicent L, Martínez-Sellés M.	Front Biosci (Landmark Ed). 2021 Nov 30;26(11):1305-1311. doi: 10.52586/5025.	Vicent L	Front Biosci (Landmark Ed)	2021	03/12/2021		10.52586/5025

Supplementary Table 1 - MEDLINE search for "Melatonin AND Atrial Fibrillation"

PMID	Title	Authors	Citation	First Author	Journal/Book	Publication Year	Create Date	PMCID	DOI
32581036	Thioretinaco Ozonide: Effects of Glyphosate, Fluoride and Electromagnetic Fields on Mitochondrial Dysfunction in Carcinogenesis, Atherogenesis and Aging	McCully KS.	Ann Clin Lab Sci. 2020 May;50(3):408-411.	McCully KS	Ann Clin Lab Sci	2020	26/06/2020		
Legend: PMID - PubMed ID, PMCID - PubMed Central ID, DOI - Digital Object Identifier									

Supplementary Table 2 – Reasons for exclusion after full text review

Paper	Year	Reason	Additional Note	Ref
Hwang et al.	2022	Catheter ablation patients		[8]
Ulus et al.	2021	Catheter ablation patients		[9]
Lee et al.	2017	Catheter ablation patients		[10]
Husser et al.	2017	Catheter ablation patients		[11]
Husser et al.	2016	Catheter ablation patients		[12]
Husser et al.	2016	Catheter ablation patients		[13]
Zhao et al.	2017	Catheter ablation patients		[14]
Meng et al.	2021	Annotation method not eQTL/TWAS	Association with network modules from WGCNA	[15]
Wang et al.	2020	Annotation method not eQTL/TWAS	Meta-Analysis of epigenome analysis, GWAS analysis and TWAS analysis data	[16]
Van Ouwerkerk et al.	2019	Annotation method not eQTL/TWAS	Integration of chromatin confirmation, epigenome and transcriptome data	[17]
Martin et al.	2015	Annotation method not eQTL/TWAS	Only selected specific regions not genome wide	[18]
Lin et al.	2014	Annotation method not eQTL/TWAS	Difference between right and left atrial expression	[19]
Ebana et al.	2017	Annotation method not eQTL/TWAS	Only selected specific regions not genome wide	[20]
Clausen et al.	2021	Annotation method not eQTL/TWAS	Only selected specific regions not genome wide	[21]
Westphal et al.	2019	Cardiac surgery patients		[22]
Kertai et al.	2015	Cardiac surgery patients		[23]
Barton et al.	2021	Outcome not AF or AF trait		[24]
Larson et al.	2007	Outcome not AF or AF trait		[25]
Lazarte et al.	2021	Analysis not been individual coding variants and traits	Polygenic risk score	[26]
Khera et al.	2018	Analysis not been individual coding variants and traits	Polygenic risk score	[27]
Weng et al.	2017	Analysis not been individual coding variants and traits	Heritability	[28]
Lin et al.	2017	Analysis not been individual coding variants and traits	Methylome wide association study	[29]
Lin et al.	2016	Analysis not been individual coding variants and traits	Gene-Gene interaction	[30]
Chalazan et al.	2021	Only sequenced specific genes/regions, not replication	Sequenced coding regions of 60 candidate genes	[31]
Vad et al.	2020	Only sequenced specific genes/regions, not replication	Sequenced loss of function variants from DCM associated genes	[32]
Wang et al.	2018	Only sequenced specific genes/regions, not replication	Sequenced 4 regions of candidate genes	[33]
Kaab et al.	2009	Only sequenced specific genes/regions, not replication	Sequenced haplotype block on chromosome 4q25	[34]
Kim et al.	2018	Only sequenced specific genes/regions, not replication	Sequenced rs17744182	[35]
Tucker et al.	2017	Only sequenced specific genes/regions, not replication	Sequenced region containing *PRXX1*	[36]
Xu et al.	2020	Review Paper		[37]
Campbell et al.	2018	Review Paper		[38]
Legend: eQTL – expression Quantitative Trait Loci; TWAS – Transcriptome Wide Association Study; WCGNA – Weighted Gene Coexpression Network Analysis; AF – Atrial Fibrillation; DCM – Dilated Cardiomyopathy.				

Supplementary Table 3 – Characteristics of studies selected for discovery sources

Author	Year	Discovery sequencing	Total	Cases	Controls	P-value threshold	Ancestry	Cohorts	Trait	Definition of trait	Meta-Analysis	Additional analysis	Note/Study Ref
Wang et al	2023	WGS	98564	5120	93444	5.0×10^{-8}	EUR + AFR + HIS + EAS + SAS + MID + OTR	All of US (details in table 1, main text)	AF	Phenotype algorithm of ICD codes, procedure and opeartion codes, self reporting	N	RVAS	Ref: [39]
Miyazawa et al	2023	GWAS	1244730	77690	1167040	5.0×10^{-8} for MAF $\geq$1%, 5.71×10^{-9} for MAF <1%	EAS + EUR	BioBank Japan, FinnGen, 36 cohorts from Neilsen et al 2018a (details in methods of main text)	AF	Phenotype methods not detailed in supplementary materials	Y	TWAS, eQTL coloc	Ref: [40]
Emmert et al	2021	GWAS	10509	110	10399	5.0×10^{-8}	EUR	CHRIS (details in methods of main text)	AF	Phenotype determined by participant questionnaire and individual level ECG data (P wave duration 0 ms)	N		Ref: [41]
Ahlberg et al	2021	GWAS	36658	N/A	N/A	5.0×10^{-8}	EUR	UK Biobank (further details published in Bycroft et al)	LAminVol, LAmaxVol, LAAEF, LATEF, LAPEF	Values derived from Cardiac MRI images using convolutional neural network described by Bai et al	N	TWAS, eQTL coloc	Ref: [42]
Baldassari et al	2020	GWAS	34668	N/A	N/A	5.0×10^{-9}	EUR + AFR + HIS + EAS	ARIC, MESA, HCHS/SOL, WHI (details in methods of main text and supplementary table 3)	P wave duration, PR segment, PR interval	Values calculated from individual participant level ECG data	Y	eQTL coloc	Ref: [43]
Weng et al	2020	ExWAS	64440	N/A	N/A	1.9×10^{-6}	EUR + AFR + HIS + EAS	15 Cohorts (detailed in supplementary material of paper and supplementary table 1)	P wave duration	Values calculated from individual participant level ECG data	Y	RVAS	Ref: [44]
Ntalla et al	2020	GWAS	293051	N/A	N/A	5.0×10^{-8}	EUR + AFR + HIS + BR	40 Cohorts (detailed in supplementary material of paper and supplementary tables 1 and 2)	PR Interval	Values calculated from individual participant level ECG data, exclusion criteria provided in methods of main text	Y	TWAS, eQTL coloc	Ref: [45]

Author	Year	Discovery sequencing	Total	Cases	Controls	P-value threshold	Ancestry	Cohorts	Trait	Definition of trait	Meta-Analysis	Additional analysis	Note/Study Ref
Van Setten et al	2019	GWAS	30000*	N/A	N/A	$5.0x10^{-8}$	EUR + AFR	8 Cohorts (detailed in supplementary table 1 and notes)	PR Interval	Values calculated from individual participant level ECG data, exclusion criteria provided in supplementary table 3	Y		approximate value for total, Ref: [46]
Choi et al	2018	WGS	7748	2789	4959	$5.0x10^{-8}$	EUR	ARIC, CCAF, HVH, FHS, MGH, PHCB, WGHS, VAFR, VAFAR, Additional controls from COPDGene, CFS, GALA II + SAGE (details in eAppendix 1 and eTable 1 of supplementary material)	Early onset AF	Multiple studies reported AF diagnoses with methods available in study supplementary material (eTable1), Early onset determined by time of diagnosis <66 years of age.	Y/N	RVAS	Ref: [47]
Thorolfsdottir et al	2018	GWAS	797262	29502	767760	$5.1x10^{-8}$ moderate, $2.1x10^{-7}$, high impact	EUR	UK Biobank, deCODE (details in main text methods)	AF	Phenotypes from ICD codes derived from EHR data, self reporting excluded	Y		Ref: [48]
Nielsen et al	2018	GWAS	1030826	60620	970216	$5.0x10^{-8}$	EUR + AFR + HIS + EAS + BR	HUNT, deCODE, MGI, DiscoverEHR, UK Biobank, AF Gen consortium (31 cohorts), (detailed information in supplementary tables 1, 3 and supplementary information pdf)	AF	Phenotypes from ICD codes derived from EHR data, details in main text methods and supplementary information pdf	Y		Ref: [49]
Van Setten et al	2018	GWAS	92340	N/A	N/A	$5.0x10^{-8}$	EUR	32 Cohorts (detailed in supplementary tables 1 and 2)	PR Interval	Values calculated from individual participant level ECG data, exclusion criteria provided in methods of main text	Y	eQTL coloc	Ref: [50]

Author	Year	Discovery sequencing	Total	Cases	Controls	P-value threshold	Ancestry	Cohorts	Trait	Definition of trait	Meta-Analysis	Additional analysis	Note/Study Ref
Roselli et al	2018	GWAS	58190	65446	522744	1.0×10^{-8}	EUR + AFR + HIS + EAS + BR	UK Biobank, BioBank Japan and 49 cohorts from AFGen and Broad AF consortiums (details in supplementary tables 1 and 17)	AF and/or AFl	Phenotypes from ICD codes derived from EHR data, details in online main text methods	Y	TWAS, eQTL coloc	Ref: [51]
Nielsen et al	2018	GWAS	67944	6337	61607	5.0×10^{-8}	EUR	HUNT (details in methods of main text)	AF	Phenotypes from ICD codes derived from EHR data, details in main text methods	N	eQTL coloc	Ref: [52]
Seyerle et al	2018	GWAS	14756	N/A	N/A	5.0×10^{-8}	HIS	HCHS/SOL, MESA, WHI (details in supplementary text)	PR Interval	Values calculated from individual participant level ECG data	Y		Ref: [53]
Lin et al	2018	ExWAS	92803	N/A	N/A	1.2×10^{-6}	EUR + AFR	27 Cohorts (detailed in methods of main text and main text table 1)	PR Interval	Values calculated from individual participant level ECG data, exclusion criteria provided in methods of main text	Y	RVAS	Ref: [54]
Thorolfsdottir et al	2017	WGS	389194	14255	374939	2.6×10^{-7}, high, 5.1×10^{-8} moderate, 4.6×10^{-9} low, 2.3×10^{-9} other in DHS sites, and 7.9×10^{-10} other	EUR	deCODE (details in methods of main text)	AF	Phenotypes from ICD codes derived from EHR data, details in main text methods	N		Ref: [55]
Christophersen et al	2017	GWAS	44456	N/A	N/A	5.0×10^{-8}	EUR + AFR	12 Cohorts (detailed in methods of main text and supplementary table 1)	P wave duration, PTF	Values calculated from individual participant level ECG data, details of measurement method in main text	Y		Ref: [56]
Low et al	2017	GWAS	36792	8180	28612	5.0×10^{-8}	EAS	BioBank Japan (details in supplementary table 1)	AF	Phenotypes from diagnoses recorded at	N		Ref: [57]

Author	Year	Discovery sequencing	Total	Cases	Controls	P-value threshold	Ancestry	Cohorts	Trait	Definition of trait	Meta-Analysis	Additional analysis	Note/Study Ref
Christophersen et al	2017	GWAS	109934	18398	91536	5.0×10^{-8}	EUR + AFR + HIS + EAS + BR	AFGen consortium (31 cohorts), (details in online methods and supplementary table 1)	AF and/or AFl	Phenotypes from either ECG documented AF, one inpatient diagnosis of AF or two outpatient diagnoses, (further details in supplementary section 2)	Y	eQTL coloc, RVAS	Ref: [58]
Christophersen et al	2017	ExWAS	155418	22806	132612	1.04×10^{-6}	EUR + AFR + HIS + EAS + BR	AFGen consortium (17 cohorts), (details in online methods and supplementary table 1)	AF and/or AFl	Phenotypes from either ECG documented AF, one inpatient diagnosis of AF or two outpatient diagnoses, (further details in supplementary section 2)	Y	eQTL coloc, RVAS	Ref: [58]
Yamada et al	2017	ExWAS	13166	884	12282	1.21×10^{-6}	EAS	Novel cohort from this study, (details in main text methods)	AF	Phenotypes derived from inpatient or outpatient diagnoses made at participating centres, follow up of community based cohort population study and death certifiacte records	N		Ref: [59]
Gudbjartsson et al	2017	WGS/GWAS	339342	1799	337543	2.0×10^{-9}	EUR	deCODE (details in methods of main text)	Early onset AF	Phenotypes derived from ICD codes for derived from hospital episode statistics of two of the largest hospitals in Iceland, early onset defined as <60 years old	N		imputed variants from WGS of 8543 Icelanders, updated analysis of 2015 paper, Ref: [60]

Author	Year	Discovery sequencing	Total	Cases	Controls	P-value threshold	Ancestry	Cohorts	Trait	Definition of trait	Meta-Analysis	Additional analysis	Note/Study Ref
Tsai et al	2016	CNV GWAS	3673	1176	2497	1.0×10^{-3}	EAS	National Taiwan AF Registry (details in methods of main text)	Severe lone AF	Severe lone AF in stage I cohort, is defined as symptomatic persistent AF with very frequent AF attacks (more than one per day) as well as no underlying cardiovascular disease that could contribute to AF, stage II frequency was at least once per week, stage III replication definition not specified	N		Ref: [61]
Gudbjartsson et al	2015	WGS/GWAS	339342	1799	337543	1.8×10^{-9}	EUR	deCODE (details in online methods and supplementary tables 1 and 2)	Early onset AF	Phenotypes derived from ICD codes for derived from hospital episode statistics of two of the largest hospitals in Iceland, early onset defined as <60 years old	N		imputed variants from WGS of 2636 Icelanders, Ref: [62]
Verweij et al	2014	GWAS	16468	N/A	N/A	5.0×10^{-8}	EUR	PREVEND, Lifelines (details in methods of main text)	P wave duration, PR Interval	Values calculated from individual participant level ECG data, exclusion criteria provided in methods of main text	Y		Ref: [63]
Hong et al	2014	GWAS	6805	N/A	N/A	5.0×10^{-8}	EAS	KARE (Details given in main text methods)	PR Interval	Values calculated from individual participant level ECG data, subjects without concurrent cardiac disease, electrolyte abonormalities at time of ECG and no medications that affect measurement	N		Ref: [64]
Butler et al	2012	GWAS	13415	N/A	N/A	5.0×10^{-8}	AFR	ARIC, CFS, JHS, MESA, BLSA, BHS, CHS, HABC, HANDLS, WHI (details in	PR Interval	Values calculated from individual participant level ECG data, exclusion criteria provided in methods of main text	Y		Ref: [65]

Author	Year	Discovery sequencing	Total	Cases	Controls	P-value threshold	Ancestry	Cohorts	Trait	Definition of trait	Meta-Analysis	Additional analysis	Note/Study Ref
								supplementary methods)					
Ellinor et al	2012	GWAS	64046	41099	22947	$5.0x10^{-8}$	EUR	ARIC, AGES, CHS, FHS, RS-1, WGHS, AFNET & KORA S4, HVH, MGH, MIGEN, SHIP, Vanderbilt (details in supplementary note 13)	Lone AF	Phenotypes from ICD codes derived from EHR data, death certificates or follow up ECGs, details in supplementary text	Y		Ref: [66]
Smith et al	2011	GWAS	6247	N/A	N/A	$2.5x10^{-8}$	AFR	ARIC, CFS, JHS, MESA (details in methods section from main text)	PR Interval	Values calculated from individual participant level ECG data	Y		Ref: [67]
Ellinor et al	2010	GWAS	14199	1355	12844	$5.0x10^{-8}$	EUR	AFNET & KORA S4, HVH & CHS, Cleveland Clinic, MGH & FHS (details in methods of main text)	Lone AF	Phenotypes from ICD codes derived from EHR data and diagnoses made by physicans on prospective collection, Lone AF defined as onset less than 66 years old without history of MI, HF or known LVSD, details in online methods text	Y		Ref: [68]
Holm et al	2010	GWAS	19525	N/A	N/A	$1.6x10^{-7}$	EUR	deCODE (details on online methods)	PR Interval	Values calculated from individual participant level ECG data	N		Ref: [69]

Author	Year	Discovery sequencing	Total	Cases	Controls	P-value threshold	Ancestry	Cohorts	Trait	Definition of trait	Meta-Analysis	Additional analysis	Note/Study Ref
Pfeufer et al	2010	GWAS	28517	N/A	N/A	5.0x10^{-8}	EUR	CHARGE (AGES, ARIC, CHS, FHS, KORAl, RS, SardiNIA), (details in online text methods)	PR Interval	Values calculated from individual participant level ECG data, details in online text methods	Y		Ref: [70]
Chambers et al	2010	GWAS	6543	N/A	N/A	5.0x10^{-8}	SAS	LOLIPOP (details given in supplementary table 1)	PR Interval	Values calculated from individual participant level ECG data	N		Ref: [71]
Benjamin et al	2009	GWAS	40518	3413	37105	5.0x10^{-8}	EUR	CHARGE (AGES, ARIC, CHS, FHS, KORAl, RS, SardiNIA), (details in supplementary methods)	AF and/or AFl	Phenotypes from ICD codes derived from EHR data, death certificates or follow up ECGs, details in supplementary text under AF ascertainment	Y		Ref: [72]
Gudbjartsson et al	2009	GWAS	36104	2385	33752	See note	EUR	deCODE (details in supplemenary methods)	AF and/or AFl	Phenotypes derived from AF and/or AFl diagnoses from two of the largest hospitals in the country	N		Not specified in methods but single variant given at 3.1x0^{-6} (9.2x10^{-9} after replication), Ref: [73]
Smith et al	2009	GWAS	1262	N/A	N/A	See note	Micronesian (Kosrae)	Novel cohort from this study (details in main text methods)	PR Interval, P wave duration, PR segement	Values calculated from individual participant level ECG data, details provided in methods of main text	N		Not specified in methods but resuts less than 1x10^{-5} given, Ref: [74]
Vasan et al	2007	GWAS	1238	N/A	N/A	1.0x10^{-5}	EUR	FHS, FOS (details given in methods of main text and original study publications)	LA diameter	Left atrium diameter determined from 2D echocardiography at end systole (further details in main methods of text)	N		Ref: [75]

Author	Year	Discovery sequencing	Total	Cases	Controls	P-value threshold	Ancestry	Cohorts	Trait	Definition of trait	Meta-Analysis	Additional analysis	Note/Study Ref
Gudbjartsson et al	2007	GWAS	5026	550	4476	See note	EUR	deCODE (details in methods of main text)	AF and/or AFl	Phenotypes derived from AF and/or AFl diagnoses from two of the largest hospitals in the country	N		Not specified in methods but highest p-value given at $2.4.1x0^{-3}$ ($9.x10^{-9}$ after replication), Ref: [76]
Cusanovich et al	2016	GWAS	318	N/A	N/A	$1.0x10^{-7}$	Hutterite (Native American)	Novel founder population	LA Volume Index	Values derived from echocardiography of individual particpiants, details in supplementary methods.	N		No signficant variants, Ref: [77]

Legend: GWAS – Genome-Wide Association Study; ExWAS – Exome-Wide Association Study; CNV – Copy Number Variant; WGS – Whole Genome Sequencing; EUR – European; AFR – African; EAS – East Asian; SAS – South Asian; HIS – Hispanic; BR – Brazilian; MID – Middle Eastern; OTR – Other; AF – Atrial Fibrillation; AFl – Atrial Flutter; LAminVol – Left Atrium Minimum Volume; LAmaxVol – Left Atrium maximum Volume; LAPEF – Left Atrial Passive Emptying Fraction; LAAEF – Left Atrial Active Emptying Fraction; LATEF – Left Atrial Total Empyting Fraction; PTF – P wave Terminal Force; ICD - International Statistical Classification of Diseases; MRI – Magnetic Resonance Imaging; ECG – Electrocardiogram; EHR – Electronic Health Record; RVAS – Rare Variant Association Study; TWAS – Transcriptome Wide Association Study; eQTL coloc – expression Quantitative Trait Loci colocalization, Study ref – Study reference.

Supplementary Table 4 – Characteristics of additional studies supporting discovery samples

Author	Year	Study type	Total	Cases	Controls	P-value threshold	Ancestry	Cohorts	Trait	Definition of trait	Meta-Analysis	Note/Study Ref
Gonzalez-Cordero et al	2021	Replication	555	69	486	Not provided	HIS	The Pharmacogenetics of Warfarin in Puerto Ricans, A Genomic Approach for Clopidogrel in Caribbean Hispanics	AF	Details of diagnosis not mentioned in methods	N	Genetic association data not provided, Ref: [78]
Hsu et al	2018	eQTL coloc	Variants from Ellinor et al 2012 and Christopherson et al 2017	N/A	N/A	Variants from Ellinor et al 2012 and Christopherson et al 2017	Variants from Ellinor et al 2012 and Christopherson et al 2017	Variants from Ellinor et al 2012 and Christopherson et al 2017	AF	Variants from Ellinor et al 2012 and Christopherson et al 2017	N/A	Ref: [79]
Sinner et al	2014	Replication	56928	14221	42707	$5.0x10^{-8}$	EUR + EAS	Heath ABC, PROSPER, RS-II, AFNET, KORA S4, DANFIB, HVH, MAC, Ottawa, BioBank Japan (Details in supplementary methods file)	Incident and Prevalent AF	Phenotypes derived from healthcare records and questionnaires from individual cohorts, however specific methods not detailed in supplementary materials	Y	Ref: [80]
Sano et al	2014	Replication	1994	N/A	N/A	0.0011	EAS	JPDSC (Details in main text methods)	PR Interval	Methods for determining PR Interval values not detailed in methods	N	Ref: [81]
Lubitz et al	2014	Replication	75992	15087	60905	$5.0x10^{-8}$	EUR + EAS	AGES, ARIC, CCAF, CHS, FHS, AFNET/KORA, LURIC, MGH, HVH, PHASE/PROSPER, RS-I, RS-II, SHIP, WGHS, BioBank Japan (details in methods of main text)	AF	Phenotypes derived from individual ECGs, Holter recordings, medical records or hospital discharge codes (details in main methods of text)	Y	Ref: [82]

Legend: EUR– European; EAS – East Asian; HIS – Hispanic; AF – Atrial Fibrillation; eQTL coloc – expression Quantitative Trait Loci colocalization; Study ref – Study reference.

Supplementary Table 5 - All targets from discovery analyses, annotated with variant to gene (annoatation provided from paper if not available)

Target	Paper	Sequencing	Main Trait	Trait	Ancestry
ABHD10	van Setten et al 2018, Roselli et al 2018 + Nielsen et al 2018a, Roselli et al 2018, Ntalla et al 2020	GWAS	PR Indices, AF Type	PR Interval, AF and/or AFl, PR Interval	EUR, EUR + AFR + EAS + HIS + BR, EUR + AFR + HIS + BR
ABHD17C	Nielsen et al 2018a	GWAS	AF Type	AF	EUR
ACER1	Yamada et al 2017	ExWAS	AF Type	AF	EAS
ACVR2B	Smith et al 2009*, Ntalla et al 2020	GWAS	PR Indices	PR Interval, PR Interval	Micronesian, EUR
ADAM11	Gudbjartsson et al 2015	WGS	AF Type	Early onset AF	EUR
ADAM15	Thorolfsdottir et al 2017	WGS	AF Type	AF	EUR
ADAMTS5	Ntalla et al 2020	GWAS	PR Indices	PR Interval	EUR + AFR + HIS + BR
ADAR	Roselli et al 2018, Nielsen et al 2018a	GWAS	AF Type	AF and/or AFl, AF	EUR
ADAT1	Yamada et al 2017	ExWAS	AF Type	AF	EAS
ADCY3	Yamada et al 2017	ExWAS	AF Type	AF	EAS
ADCY5	Ntalla et al 2020	GWAS	PR Indices	PR Interval	EUR + AFR + HIS + BR
ADCY7	Yamada et al 2017	ExWAS	AF Type	AF	EAS
ADGRL3	Ntalla et al 2020	GWAS	PR Indices	PR Interval	EUR + AFR + HIS + BR, EUR
ADPRHL1	Ntalla et al 2020, Lin et al 2018	GWAS, ExWAS	PR Indices	PR Interval , PR Interval	EUR + AFR + HIS + BR, EUR + AFR
ADRA1A	Yamada et al 2017	ExWAS	AF Type	AF	EAS
ADRB1	Ntalla et al 2020	GWAS	PR Indices	PR Interval	EUR + AFR + HIS + BR, EUR
AGA	Yamada et al 2017	ExWAS	AF Type	AF	EAS
AGO2	Roselli et al 2018 + Nielsen et al 2018a, Roselli et al 2018, Nielsen et al 2018a, Miyazawa et al 2023	GWAS	AF Type	AF and/or AFl, AF	EUR + AFR + EAS + HIS + BR, EUR, EUR + EAS
AKAP6	Roselli et al 2018 + Nielsen et al 2018a, Roselli et al 2018, Ntalla et al 2020, Nielsen et al 2018a, Miyazawa et al 2023	GWAS	AF Type, PR Indices	AF and/or AFl, PR Interval , AF	EUR + AFR + EAS + HIS + BR, EUR, EUR + AFR + HIS + BR, EAS, EUR + EAS
ALDH18A1	Ntalla et al 2020	GWAS	PR Indices	PR Interval	EUR + AFR + HIS + BR
ALDH8A1	Miyazawa et al 2023	GWAS	AF Type	AF	EUR + EAS
ALPK2	Yamada et al 2017	ExWAS	AF Type	AF	EAS
ALPK3	Ntalla et al 2020, Christopherson et al 2017b	GWAS	PR Indices	PR Interval , PTF	EUR + AFR + HIS + BR, EUR, EUR + AFR
ANKRD1	Ahlberg et al 2020	GWAS	Left Atrium Indices	LAAEF, LAmaxVol, LAminVol	EUR

Target	Paper	Sequencing	Main Trait	Trait	Ancestry
AOPEP	Roselli et al 2018 + Nielsen et al 2018a, Roselli et al 2018, Nielsen et al 2018a, Miyazawa et al 2023, Low et al 2017, Ellinor et al 2012, Christopherson et al 2017a, Ahlberg et al 2020	GWAS	AF Type, Left Atrium Indices	AF and/or AFl, AF, Lone AF, LATEF	EUR + AFR + EAS + HIS + BR, EUR, EUR + EAS, EAS
APOLD1	Miyazawa et al 2023	GWAS	AF Type	AF	EUR + EAS
ARFGEF1	Yamada et al 2017	ExWAS	AF Type	AF	EAS
ARHGAP10	Weng et al 2020, Roselli et al 2018 + Nielsen et al 2018a, Roselli et al 2018, Nielsen et al 2018a, Miyazawa et al 2023	ExWAS, GWAS	PR Indices, AF Type	P wave duration, AF and/or AFl, AF	EUR + AFR + EAS + HIS, EUR, EUR + AFR + EAS + HIS + BR, EAS, EUR + EAS
ARHGAP24	Verweij et al 2014, Seyerle et al 2018, Pfeufer et al 2010, Ntalla et al 2020, Lin et al 2018, Holm et al 2010*, Butler et al 2012, Van Setten et al 2019	GWAS, ExWAS	PR Indices	PR Interval, PR segment, PR Interval	EUR, HIS, EUR + AFR + HIS + BR, EUR + AFR, AFR
ARHGAP44	van Setten et al 2018, Ntalla et al 2020, Van Setten et al 2019	GWAS	PR Indices	PR Interval, PR Interval	EUR, EUR + AFR + HIS + BR, EUR + AFR
ARHGAP8	Yamada et al 2017	ExWAS	AF Type	AF	EAS
ARID2	Ntalla et al 2020	GWAS	PR Indices	PR Interval	EUR + AFR + HIS + BR, EUR
ARL14EP	Ntalla et al 2020	GWAS	PR Indices	PR Interval	EUR + AFR + HIS + BR
ARNT2	Roselli et al 2018 + Nielsen et al 2018a, Roselli et al 2018, Nielsen et al 2018a, Miyazawa et al 2023	GWAS	AF Type	AF and/or AFl, AF	EUR + AFR + EAS + HIS + BR, EUR, EUR + EAS
ARRDC4	Yamada et al 2017	ExWAS	AF Type	AF	EAS
ASAH1	Roselli et al 2018 + Nielsen et al 2018a, Roselli et al 2018, Nielsen et al 2018a, Miyazawa et al 2023, Christopherson et al 2017a	GWAS	AF Type	AF and/or AFl, AF	EUR + AFR + EAS + HIS + BR, EUR, EUR + EAS
ASB13	Yamada et al 2017	ExWAS	AF Type	AF	EAS
AXDND1	Yamada et al 2017	ExWAS	AF Type	AF	EAS
AZIN1	Ntalla et al 2020	GWAS	PR Indices	PR Interval	EUR
B3GALT1	Yamada et al 2017	ExWAS	AF Type	AF	EAS
BCAR1	Ntalla et al 2020	GWAS	PR Indices	RBINT PR Interval	EUR + AFR + HIS + BR
BCKDHB	Yamada et al 2017	ExWAS	AF Type	AF	EAS
BEND5	Roselli et al 2018 + Nielsen et al 2018a, Nielsen et al 2018a	GWAS	AF Type	AF and/or AFl, AF	EUR + AFR + EAS + HIS + BR, EUR
BEND7	Ntalla et al 2020	GWAS	PR Indices	PR Interval	EUR + AFR + HIS + BR
BEST3	Roselli et al 2018 + Nielsen et al 2018a, Roselli et al 2018, Nielsen et al 2018a	GWAS	AF Type	AF and/or AFl, AF	EUR + AFR + EAS + HIS + BR, EUR
BHLHE41	Roselli et al 2018 + Nielsen et al 2018a, Roselli et al 2018, Ntalla et al 2020, Nielsen et al 2018a, Miyazawa et al 2023	GWAS	AF Type, PR Indices	AF and/or AFl, PR Interval , AF	EUR + AFR + EAS + HIS + BR, EUR, EUR + AFR + HIS + BR, EAS, EUR + EAS
BICRA	Miyazawa et al 2023	GWAS	AF Type	AF	EUR + EAS
BLID	Nielsen et al 2018a	GWAS	AF Type	AF	EUR

Target	Paper	Sequencing	Main Trait	Trait	Ancestry
BLK	Yamada et al 2017	ExWAS	AF Type	AF	EAS
BMP2	Roselli et al 2018	GWAS	AF Type	AF and/or AFl	EUR, EUR + AFR + EAS + HIS + BR
BMP8A	Lin et al 2018	ExWAS	PR Indices	PR Interval	EUR + AFR
BMPR1A	Ntalla et al 2020	GWAS	PR Indices	PR Interval	EUR + AFR + HIS + BR, EUR
BNIP1	Ntalla et al 2020, Van Setten et al 2019	GWAS	PR Indices	PR Interval , PR Interval	EUR + AFR + HIS + BR, EUR + AFR
BRINP3	Vasan et al 2007*	GWAS	Left Atrium Indices	LA diameter	EUR
C10orf71	Ntalla et al 2020, Miyazawa et al 2023	GWAS	PR Indices, AF Type	PR Interval , AF	EUR + AFR + HIS + BR, EUR, EUR + EAS
C17orf107	Ntalla et al 2020	GWAS	PR Indices	PR Interval	EUR + AFR + HIS + BR, EUR
C1D	Ntalla et al 2020	GWAS	PR Indices	PR Interval	EUR + AFR + HIS + BR
C1orf185	Roselli et al 2018 + Nielsen et al 2018a, Roselli et al 2018, Nielsen et al 2018a, Lin et al 2018	GWAS, ExWAS	AF Type, PR Indices	AF and/or AFl, AF, PR Interval	EUR + AFR + EAS + HIS + BR, EUR, EUR + AFR
C2orf74	Nielsen et al 2018a	GWAS	AF Type	AF	EUR
C5orf47	Nielsen et al 2018a	GWAS	AF Type	AF	EUR
C8orf48	Ntalla et al 2020	GWAS	PR Indices	PR Interval	EUR, EUR + AFR + HIS + BR
C8orf74	Yamada et al 2017	ExWAS	AF Type	AF	EAS
C9orf3	Miyazawa et al 2023	GWAS	AF Type	AF	EAS
CA4	Yamada et al 2017	ExWAS	AF Type	AF	EAS
CACFD1	Yamada et al 2017	ExWAS	AF Type	AF	EAS
CACNA1G	Ntalla et al 2020	GWAS	PR Indices	PR Interval	EUR + AFR + HIS + BR, EUR
CALM3	Christopherson et al 2017b	GWAS	PR Indices	PTF	EUR, EUR + AFR
CAMK2D	van Setten et al 2018, Roselli et al 2018 + Nielsen et al 2018a, Roselli et al 2018, Ntalla et al 2020, Nielsen et al 2018a, Miyazawa et al 2023, Christopherson et al 2017b	GWAS	PR Indices, AF Type	PR Interval, AF and/or AFl, PR Interval , AF, P wave duration	EUR, EUR + AFR + EAS + HIS + BR, EUR + AFR + HIS + BR, EUR + EAS
CAND2	Weng et al 2020, Sinner et al 2014, Roselli et al 2018 + Nielsen et al 2018a, Roselli et al 2018, Nielsen et al 2018a, Miyazawa et al 2023, Christopherson et al 2017b, Christopherson et al 2017a	ExWAS, Replication, GWAS	PR Indices, AF Type	P wave duration, Lone AF, AF and/or AFl, AF	EUR + AFR + EAS + HIS, EUR, EUR + AFR + EAS + HIS + BR, EUR + EAS, EUR + AFR
CARMIL1	Ntalla et al 2020	GWAS	PR Indices	PR Interval	EUR + AFR + HIS + BR
CASKIN2	Ntalla et al 2020	GWAS	PR Indices	PR Interval	EUR

Target	Paper	Sequencing	Main Trait	Trait	Ancestry
CASQ2	Roselli et al 2018 + Nielsen et al 2018a, Roselli et al 2018, Ntalla et al 2020, Nielsen et al 2018a, Miyazawa et al 2023, Ahlberg et al 2020	GWAS	AF Type, PR Indices, Left Atrium Indices	AF and/or AFl, RBINT PR Interval, AF, LAAEF, LATEF	EUR + AFR + EAS + HIS + BR, EUR, EUR + AFR + HIS + BR, EUR + EAS
CASZ1	Roselli et al 2018 + Nielsen et al 2018a, Roselli et al 2018, Nielsen et al 2018a, Miyazawa et al 2023	GWAS	AF Type	AF and/or AFl, AF	EUR + AFR + EAS + HIS + BR, EUR, EUR + EAS
CAV1	Weng et al 2020, Verweij et al 2014, Seyerle et al 2018, Roselli et al 2018 + Nielsen et al 2018a, Roselli et al 2018, Pfeufer et al 2010, Ntalla et al 2020, Nielsen et al 2018a, Miyazawa et al 2023, Low et al 2017, Lin et al 2018, Holm et al 2010*, Ellinor et al 2012, Christopherson et al 2017b, Christopherson et al 2017a, Butler et al 2012, Sano et al 2014, Van Setten et al 2019	ExWAS, GWAS, Replication	PR Indices, AF Type	P wave duration, PR Interval, PR segment, AF and/or AFl, PR Interval , AF, Lone AF	EUR + AFR + EAS + HIS, EUR, HIS, EUR + AFR + EAS + HIS + BR, EUR + AFR + HIS + BR, EAS, EUR + EAS, EUR + AFR, AFR
CAV2	Sinner et al 2014	Replication	AF Type	Lone AF	EAS
CAVIN2	Ntalla et al 2020	GWAS	PR Indices	PR Interval	EUR + AFR + HIS + BR, EUR
CBARP	Ntalla et al 2020	GWAS	PR Indices	PR Interval	EUR + AFR + HIS + BR, EUR
CBX8	Ntalla et al 2020	GWAS	PR Indices	PR Interval	EUR + AFR + HIS + BR
CCBE1	Yamada et al 2017, Ntalla et al 2020	ExWAS, GWAS	AF Type, PR Indices	AF, PR Interval	EAS, EUR
CCDC134	Miyazawa et al 2023	GWAS	AF Type	AF	EUR + EAS
CCDC168	Yamada et al 2017	ExWAS	AF Type	AF	EAS
CCDC7	Ntalla et al 2020, Miyazawa et al 2023	GWAS	PR Indices, AF Type	PR Interval , AF	EUR + AFR + HIS + BR, EUR, EUR + EAS
CCDC71	Yamada et al 2017	ExWAS	AF Type	AF	EAS
CCNL1	van Setten et al 2018, Ntalla et al 2020	GWAS	PR Indices	PR Interval, PR Interval	EUR, EUR + AFR + HIS + BR
CD69	Yamada et al 2017	ExWAS	AF Type	AF	EAS
CD96	Yamada et al 2017	ExWAS	AF Type	AF	EAS
CDADC1	Ntalla et al 2020	GWAS	PR Indices	PR Interval	EUR + AFR + HIS + BR
CDC42BPG	Yamada et al 2017	ExWAS	AF Type	AF	EAS
CDC7	Ntalla et al 2020	GWAS	PR Indices	PR Interval	EUR + AFR + HIS + BR, EUR
CDH13	van Setten et al 2018, Ntalla et al 2020	GWAS	PR Indices	PR Interval, PR Interval	EUR + AFR, EUR + AFR + HIS + BR
CDH2	Ntalla et al 2020	GWAS	PR Indices	PR Interval	EUR + AFR + HIS + BR
CDHR3	Miyazawa et al 2023	GWAS	AF Type	AF	EUR + EAS
CDKN1A	Roselli et al 2018 + Nielsen et al 2018a, Roselli et al 2018, Ntalla et al 2020, Nielsen et al 2018a, Miyazawa et al 2023	GWAS	AF Type, PR Indices	AF and/or AFl, PR Interval , AF	EUR + AFR + EAS + HIS + BR, EUR, EUR + AFR + HIS + BR, EUR + EAS

Target	Paper	Sequencing	Main Trait	Trait	Ancestry
CELA3B	Ntalla et al 2020	GWAS	PR Indices	PR Interval	EUR
CEP152	Yamada et al 2017	ExWAS	AF Type	AF	EAS
CEP68	Roselli et al 2018 + Nielsen et al 2018a, Roselli et al 2018, Nielsen et al 2018a, Miyazawa et al 2023, Low et al 2017, Christopherson et al 2017a	GWAS	AF Type	AF and/or AFl, AF	EUR + AFR + EAS + HIS + BR, EUR, EUR + EAS, EAS
CEP85L	Roselli et al 2018, Ntalla et al 2020, Miyazawa et al 2023	GWAS	AF Type, PR Indices	AF and/or AFl, PR Interval , AF	EUR, EUR + EAS
CFL2	Roselli et al 2018 + Nielsen et al 2018a, Roselli et al 2018, Nielsen et al 2018a, Miyazawa et al 2023	GWAS	AF Type	AF and/or AFl, AF	EUR + AFR + EAS + HIS + BR, EUR, EUR + EAS
CGA	Nielsen et al 2018a	GWAS	AF Type	AF	EUR
CHDH	Ntalla et al 2020	GWAS	PR Indices	PR Interval	EUR + AFR + HIS + BR
CHN1	Smith et al 2009*	GWAS	PR Indices	PR Interval	Micronesian
CHPF	Ntalla et al 2020	GWAS	PR Indices	PR Interval	EUR + AFR + HIS + BR, EUR
CHRM2	Ntalla et al 2020	GWAS	PR Indices	PR Interval	EUR + AFR + HIS + BR
CHST14	Yamada et al 2017	ExWAS	AF Type	AF	EAS
CILP	Ahlberg et al 2020	GWAS	Left Atrium Indices	LAAEF	EUR
CITED4	Ahlberg et al 2020	GWAS	Left Atrium Indices	LATEF	EUR
CKB	Ntalla et al 2020	GWAS	PR Indices	PR Interval	EUR
CKS1B	Choi et al 2018	WGS	AF Type	Early onset AF	EUR
CLDN25	Ntalla et al 2020	GWAS	PR Indices	PR Interval	EUR + AFR + HIS + BR, EUR
CLIC6	Roselli et al 2018 + Nielsen et al 2018a, Roselli et al 2018, Nielsen et al 2018a, Miyazawa et al 2023	GWAS	AF Type	AF and/or AFl, AF	EUR + AFR + EAS + HIS + BR, EUR, EUR + EAS
CMYA5	Yamada et al 2017	ExWAS	AF Type	AF	EAS
CNKSR3	Ntalla et al 2020	GWAS	PR Indices	RBINT PR Interval	EUR + AFR + HIS + BR
COL13A1	Ntalla et al 2020	GWAS	PR Indices	PR Interval	EUR + AFR + HIS + BR, EUR
COL8A1	Ahlberg et al 2020	GWAS	Left Atrium Indices	LAminVol	EUR
COQ8A	Ntalla et al 2020	GWAS	PR Indices	PR Interval	EUR + AFR + HIS + BR, EUR
CORO2B	Ntalla et al 2020	GWAS	PR Indices	PR Interval	EUR + AFR + HIS + BR, EUR
COX7A2L	Ntalla et al 2020	GWAS	PR Indices	PR Interval	EUR + AFR + HIS + BR, EUR
CPSF6	Christopherson et al 2017a	GWAS	AF Type	AF and/or AFl	EAS
CRAMP1	Nielsen et al 2018a	GWAS	AF Type	AF	EUR

Target	Paper	Sequencing	Main Trait	Trait	Ancestry
CREB5	Roselli et al 2018 + Nielsen et al 2018a, Roselli et al 2018, Nielsen et al 2018a, Miyazawa et al 2023	GWAS	AF Type	AF and/or AFl, AF	EUR + AFR + EAS + HIS + BR, EUR, EUR + EAS
CREBRF	Verweij et al 2014, Pfeufer et al 2010, Ntalla et al 2020, Lin et al 2018	GWAS, ExWAS	PR Indices	PR Interval, PR Interval	EUR, EUR + AFR
CRYBG2	Ntalla et al 2020	GWAS	PR Indices	PR Interval	EUR + AFR + HIS + BR
CSHL1	Ntalla et al 2020	GWAS	PR Indices	PR Interval	EUR + AFR + HIS + BR
CSPG4	Yamada et al 2017	ExWAS	AF Type	AF	EAS
CTDSPL	Ntalla et al 2020	GWAS	PR Indices	PR Interval	EUR
CTNNA3	Miyazawa et al 2023	GWAS	AF Type	AF	EAS
CTU2	Yamada et al 2017	ExWAS	AF Type	AF	EAS
CUX2	Miyazawa et al 2023	GWAS	AF Type	AF	EAS, EUR + EAS
CYB5RL	Ntalla et al 2020, Christopherson et al 2017b	GWAS	PR Indices	PR Interval , P wave duration	EUR + AFR + HIS + BR, EUR, EUR + AFR
CYTH1	Roselli et al 2018 + Nielsen et al 2018a, Nielsen et al 2018a	GWAS	AF Type	AF and/or AFl, AF	EUR + AFR + EAS + HIS + BR, EUR
DAG1	Ntalla et al 2020	GWAS	PR Indices	PR Interval	EUR
DBX1	van Setten et al 2018, Roselli et al 2018 + Nielsen et al 2018a, Roselli et al 2018, Ntalla et al 2020, Nielsen et al 2018a, Miyazawa et al 2023, Choi et al 2018	GWAS, WGS	PR Indices, AF Type	PR Interval, AF and/or AFl, PR Interval , AF, Early onset AF	EUR, EUR + AFR + EAS + HIS + BR, EUR + AFR + HIS + BR, EUR + EAS
DEFB136	Miyazawa et al 2023	GWAS	AF Type	AF	EUR + EAS
DEK	Ntalla et al 2020	GWAS	PR Indices	PR Interval	EUR + AFR + HIS + BR, EUR
DERL3	Ntalla et al 2020	GWAS	PR Indices	PR Interval	EUR + AFR + HIS + BR
DLEC1	Weng et al 2020, Lin et al 2018	ExWAS	PR Indices	P wave duration, PR Interval	EUR + AFR + EAS + HIS, EUR, EUR + AFR
DMRTA2	Nielsen et al 2018b	GWAS	AF Type	AF	EUR
DNAH11	Ntalla et al 2020	GWAS	PR Indices	PR Interval	EUR + AFR + HIS + BR, EUR
DNAH17	Yamada et al 2017	ExWAS	AF Type	AF	EAS
DNAI2	Yamada et al 2017	ExWAS	AF Type	AF	EAS
DNAJC12	Miyazawa et al 2023	GWAS	AF Type	AF	EUR + EAS
DPF3	Roselli et al 2018 + Nielsen et al 2018a, Nielsen et al 2018a, Miyazawa et al 2023	GWAS	AF Type	AF and/or AFl, AF	EUR + AFR + EAS + HIS + BR, EUR, EUR + EAS
DPT	Ntalla et al 2020	GWAS	PR Indices	PR Interval	EUR + AFR + HIS + BR
DSP	Ntalla et al 2020	GWAS	PR Indices	PR Interval	EUR, EUR + AFR + HIS + BR

Target	Paper	Sequencing	Main Trait	Trait	Ancestry
EDN2	Ntalla et al 2020	GWAS	PR Indices	PR Interval	EUR + AFR + HIS + BR, EUR
EEF1AKMT1	van Setten et al 2018, Ntalla et al 2020, Lin et al 2018	GWAS, ExWAS	PR Indices	PR Interval, PR Interval	EUR, EUR + AFR
EFHD1	Yamada et al 2017	ExWAS	AF Type	AF	EAS
EFNA5	Roselli et al 2018 + Nielsen et al 2018a, Nielsen et al 2018a, Miyazawa et al 2023	GWAS	AF Type	AF and/or AFl, AF	EUR + AFR + EAS + HIS + BR, EUR, EUR + EAS
EHD4	Yamada et al 2017	ExWAS	AF Type	AF	EAS
EIF2D	Ahlberg et al 2020	GWAS	Left Atrium Indices	LAmaxVol	EUR
ELMO1	Ntalla et al 2020	GWAS	PR Indices	PR Interval	EUR + AFR + HIS + BR
ELOA2	Yamada et al 2017	ExWAS	AF Type	AF	EAS
ELOVL3	Roselli et al 2018	GWAS	AF Type	AF and/or AFl	EUR
EMB	Baldassari et al 2020	GWAS	PR Indices	P wave duration	EUR + HIS + AFR
EMILIN3	Ntalla et al 2020	GWAS	PR Indices	PR Interval	EUR + AFR + HIS + BR
EML6	Ntalla et al 2020	GWAS	PR Indices	PR Interval	EUR + AFR + HIS + BR, EUR
ENPEP	Nielsen et al 2018a	GWAS	AF Type	AF	EUR
ENTPD6	Ntalla et al 2020	GWAS	PR Indices	RBINT PR Interval	EUR + AFR + HIS + BR
EOMES	van Setten et al 2018, Ntalla et al 2020	GWAS	PR Indices	PR Interval, PR Interval	EUR, EUR + AFR + HIS + BR
EPAS1	Ntalla et al 2020, Christopherson et al 2017b	GWAS	PR Indices	PR Interval , P wave duration	EUR + AFR + HIS + BR, EUR, EUR + AFR
EPHA3	Roselli et al 2018 + Nielsen et al 2018a, Roselli et al 2018, Nielsen et al 2018a, Miyazawa et al 2023	GWAS	AF Type	AF and/or AFl, AF	EUR + AFR + EAS + HIS + BR, EUR, EUR + EAS
EPN1	Yamada et al 2017	ExWAS	AF Type	AF	EAS
EPPK1	Lin et al 2018	ExWAS	PR Indices	PR Interval	EUR + AFR
EPS15	Miyazawa et al 2023	GWAS	AF Type	AF	EUR + EAS
ERBB4	Roselli et al 2018 + Nielsen et al 2018a, Nielsen et al 2018a, Miyazawa et al 2023	GWAS	AF Type	AF and/or AFl, AF	EUR + AFR + EAS + HIS + BR, EUR, EUR + EAS
ESR2	Roselli et al 2018 + Nielsen et al 2018a, Roselli et al 2018, Nielsen et al 2018a, Miyazawa et al 2023, Low et al 2017, Ellinor et al 2012, Christopherson et al 2017a	GWAS, ExWAS	AF Type	AF and/or AFl, AF, Lone AF	EUR + AFR + EAS + HIS + BR, EUR, EAS, EUR + EAS
ETV1	Roselli et al 2018 + Nielsen et al 2018a, Roselli et al 2018, Ntalla et al 2020, Nielsen et al 2018a, Miyazawa et al 2023	GWAS	AF Type, PR Indices	AF and/or AFl, PR Interval , AF	EUR + AFR + EAS + HIS + BR, EUR, EUR + AFR + HIS + BR, EUR + EAS

Target	Paper	Sequencing	Main Trait	Trait	Ancestry
EXOG	Weng et al 2020, Smith et al 2009*, Ntalla et al 2020, Van Setten et al 2019	ExWAS, GWAS	PR Indices	P wave duration, PR Interval, PR Interval	EUR + AFR + HIS + EAS, Micronesian, EUR, EUR + AFR
EXT1	Miyazawa et al 2023	GWAS	AF Type	AF	EUR + EAS
EYA4	Miyazawa et al 2023	GWAS	AF Type	AF	EAS, EUR + EAS
FAAP20	Ntalla et al 2020, Van Setten et al 2019	GWAS	PR Indices	PR Interval , PR Interval	EUR + AFR + HIS + BR, EUR + AFR
FADS1	Weng et al 2020, Verweij et al 2014, Ntalla et al 2020	ExWAS, GWAS	PR Indices	P wave duration, PR Interval	EUR, EUR + AFR + HIS + BR
FADS2	Weng et al 2020, Ntalla et al 2020	ExWAS, GWAS	PR Indices	P wave duration, PR Interval	EUR + AFR + EAS + HIS, EUR + AFR + HIS + BR
FAF1	Ntalla et al 2020	GWAS	PR Indices	PR Interval	EUR + AFR + HIS + BR
FAM117B	Ntalla et al 2020	GWAS	PR Indices	PR Interval	EUR
FAM133B	Weng et al 2020, Roselli et al 2018 + Nielsen et al 2018a, Roselli et al 2018, Nielsen et al 2018a, Miyazawa et al 2023	ExWAS, GWAS	PR Indices, AF Type	P wave duration, AF and/or AFl, AF	EUR + AFR + EAS + HIS, EUR, EUR + AFR + EAS + HIS + BR, EUR + EAS
FAM13B	Roselli et al 2018	GWAS	AF Type	AF and/or AFl	EUR
FAM222A	Miyazawa et al 2023	GWAS	AF Type	AF	EUR + EAS
FAM241A	Nielsen et al 2018a, Miyazawa et al 2023	GWAS	AF Type	AF	EUR, EAS
FANCC	Miyazawa et al 2023	GWAS	AF Type	AF	EAS
FANCL	Yamada et al 2017	ExWAS	AF Type	AF	EAS
FAT1	van Setten et al 2018, Ntalla et al 2020, Lin et al 2018	GWAS, ExWAS	PR Indices	PR Interval, PR Interval	EUR, EUR + AFR + HIS + BR, EUR + AFR
FBN2	Roselli et al 2018 + Nielsen et al 2018a, Nielsen et al 2018a, Miyazawa et al 2023	GWAS	AF Type	AF and/or AFl, AF	EUR + AFR + EAS + HIS + BR, EUR, EUR + EAS
FBRSL1	Miyazawa et al 2023	GWAS	AF Type	AF	EUR + EAS
FBXL7	Ntalla et al 2020	GWAS	PR Indices	RBINT PR Interval	EUR
FBXO11	Ntalla et al 2020	GWAS	PR Indices	PR Interval	EUR + AFR + HIS + BR
FBXO32	Roselli et al 2018 + Nielsen et al 2018a, Roselli et al 2018, Ntalla et al 2020, Nielsen et al 2018a	GWAS	AF Type, PR Indices	AF and/or AFl, PR Interval , AF	EUR + AFR + EAS + HIS + BR, EUR
FCRL1	Yamada et al 2017	ExWAS	AF Type	AF	EAS
FER	Ntalla et al 2020	GWAS	PR Indices	PR Interval	EUR
FERMT2	van Setten et al 2018	GWAS	PR Indices	PR Interval	EUR
FGF12	Ntalla et al 2020	GWAS	PR Indices	PR Interval	EUR + AFR + HIS + BR, EUR
FGF13	Miyazawa et al 2023	GWAS	AF Type	AF	EAS
FGF14	Ntalla et al 2020	GWAS	PR Indices	PR Interval	EUR + AFR + HIS + BR, EUR

Target	Paper	Sequencing	Main Trait	Trait	Ancestry
FGF18	Ntalla et al 2020	GWAS	PR Indices	PR Interval	EUR + AFR + HIS + BR, EUR
FGF5	Roselli et al 2018 + Nielsen et al 2018a, Ntalla et al 2020, Nielsen et al 2018a, Miyazawa et al 2023	GWAS	AF Type, PR Indices	AF and/or AFl, PR Interval , AF	EUR + AFR + EAS + HIS + BR, EUR, EUR + AFR + HIS + BR, EUR + EAS
FGFR1	van Setten et al 2018, Ntalla et al 2020	GWAS	PR Indices	PR Interval, PR Interval	EUR, EUR + AFR + HIS + BR
FGFR2	Ntalla et al 2020	GWAS	PR Indices	PR Interval	EUR + AFR + HIS + BR, EUR
FHL2	Ntalla et al 2020	GWAS	PR Indices	PR Interval	EUR + AFR + HIS + BR, EUR
FIGN	van Setten et al 2018, Ntalla et al 2020	GWAS	PR Indices	PR Interval, PR Interval	EUR, EUR + AFR + HIS + BR
FILIP1	Miyazawa et al 2023	GWAS	AF Type	AF	EUR + EAS
FLRT2	Ntalla et al 2020	GWAS	PR Indices	PR Interval	EUR + AFR + HIS + BR, EUR
FNDC3B	Ntalla et al 2020	GWAS	PR Indices	PR Interval	EUR + AFR + HIS + BR, EUR
FOXC1	Yamada et al 2017	ExWAS	AF Type	AF	EAS
FOXG1	Yamada et al 2017, Ntalla et al 2020	ExWAS, GWAS	AF Type, PR Indices	AF, PR Interval	EAS, EUR + AFR + HIS + BR, EUR
FOXN4	Yamada et al 2017	ExWAS	AF Type	AF	EAS
FOXP1	van Setten et al 2018, Ntalla et al 2020	GWAS	PR Indices	PR Interval, PR Interval	EUR + AFR, EUR + AFR + HIS + BR
FREM2	Yamada et al 2017, Ntalla et al 2020	ExWAS, GWAS	AF Type, PR Indices	AF, PR Interval	EAS, EUR + AFR + HIS + BR, EUR
FRMD4B	van Setten et al 2018, Roselli et al 2018 + Nielsen et al 2018a, Ntalla et al 2020, Nielsen et al 2018a, Miyazawa et al 2023	GWAS	PR Indices, AF Type	and/or AFl, PR Interval , AF	EUR, EUR + AFR + EAS + HIS + BR, EUR + AFR + HIS + BR, EUR + EAS
FTCD	Yamada et al 2017	ExWAS	AF Type	AF	EAS
GAREM1	Ntalla et al 2020	GWAS	PR Indices	PR Interval	EUR + AFR + HIS + BR, EUR
GATA2	Yamada et al 2017	ExWAS	AF Type	AF	EAS
GATA4	Yamada et al 2017, Roselli et al 2018 + Nielsen et al 2018a, Nielsen et al 2018a	ExWAS, GWAS	AF Type	AF, AF and/or AFl	EAS, EUR + AFR + EAS + HIS + BR, EUR
GATA5	Miyazawa et al 2023	GWAS	AF Type	AF	EUR + EAS
GC	Yamada et al 2017	ExWAS	AF Type	AF	EAS
GCG	Yamada et al 2017	ExWAS	AF Type	AF	EAS
GCOM1	Thorolfsdottir et al 2018, Roselli et al 2018 + Nielsen et al 2018a, Nielsen et al 2018a	GWAS	AF Type	AF, AF and/or AFl	EUR, EUR + AFR + EAS + HIS + BR
GET4	Ntalla et al 2020, Miyazawa et al 2023	GWAS	PR Indices, AF Type	PR Interval , AF	EUR + AFR + HIS + BR, EUR, EUR + EAS
GFM1	Ntalla et al 2020	GWAS	PR Indices	PR Interval	EUR

Target	Paper	Sequencing	Main Trait	Trait	Ancestry
GGACT	Emmert et al 2021	GWAS	AF Type	AF	EUR
GH1	Ntalla et al 2020	GWAS	PR Indices	PR Interval	EUR
GIN1	Lin et al 2018	ExWAS	PR Indices	PR Interval	EUR + AFR
GJA1	Ntalla et al 2020, Nielsen et al 2018a, Lin et al 2018	GWAS, ExWAS	PR Indices, AF Type	PR Interval , AF, PR Interval	EUR, EUR + AFR + HIS + BR, EUR + AFR
GJA5	Roselli et al 2018 + Nielsen et al 2018a, Roselli et al 2018, Ntalla et al 2020, Nielsen et al 2018a, Miyazawa et al 2023	GWAS	AF Type, PR Indices	AF and/or AFl, PR Interval , AF	EUR + AFR + EAS + HIS + BR, EUR, EUR + AFR + HIS + BR, EAS, EUR + EAS
GMCL1	Christopherson et al 2017a	GWAS	AF Type	AF and/or AFl	EUR + AFR + EAS + HIS + BR
GMPR	Roselli et al 2018 + Nielsen et al 2018a, Roselli et al 2018, Nielsen et al 2018a, Miyazawa et al 2023	GWAS	AF Type	AF and/or AFl, AF	EUR + AFR + EAS + HIS + BR, EUR, EUR + EAS
GNB4	Roselli et al 2018 + Nielsen et al 2018a, Roselli et al 2018, Nielsen et al 2018a, Miyazawa et al 2023	GWAS	AF Type	AF and/or AFl, AF	EUR + AFR + EAS + HIS + BR, EUR, EUR + EAS
GNPNAT1	Ntalla et al 2020	GWAS	PR Indices	PR Interval	EUR + AFR + HIS + BR
GNS	Ntalla et al 2020	GWAS	PR Indices	PR Interval	EUR
GORAB	Thorolfsdottir et al 2017, Sinner et al 2014	WGS, Replication	AF Type	AF, Lone AF	EUR, EAS
GOSR2	Weng et al 2020	ExWAS	PR Indices	P wave duration	EUR + AFR + EAS + HIS, EUR
GTF2I	Roselli et al 2018 + Nielsen et al 2018a, Roselli et al 2018	GWAS	AF Type	AF and/or AFl	EUR + AFR + EAS + HIS + BR, EUR
GYPC	Roselli et al 2018 + Nielsen et al 2018a, Nielsen et al 2018a	GWAS	AF Type	AF and/or AFl, AF	EUR + AFR + EAS + HIS + BR, EUR
H2BC4	Yamada et al 2017	ExWAS	AF Type	AF	EAS
HAND2	Roselli et al 2018 + Nielsen et al 2018a, Roselli et al 2018, Ntalla et al 2020, Nielsen et al 2018a, Miyazawa et al 2023, Low et al 2017, Lin et al 2018	GWAS, ExWAS	AF Type, PR Indices	AF and/or AFl, PR Interval , AF, PR Interval	EUR + AFR + EAS + HIS + BR, EUR, EUR + AFR + HIS + BR, EAS, EUR + EAS, EUR + AFR
HBEGF	Low et al 2017	GWAS	AF Type	AF	EAS
HBP1	Roselli et al 2018	GWAS	AF Type	AF and/or AFl	EUR + AFR + EAS + HIS + BR
HCFC2	Miyazawa et al 2023	GWAS	AF Type	AF	EAS, EUR + EAS
HCN1	Weng et al 2020, Ntalla et al 2020, Christopherson et al 2017b	ExWAS, GWAS	PR Indices	P wave duration, PR Interval	EUR + AFR + EAS + HIS, EUR, EUR + AFR + HIS + BR, EUR + AFR
HCN4	Roselli et al 2018 + Nielsen et al 2018a, Roselli et al 2018, Ntalla et al 2020, Nielsen et al 2018a, Miyazawa et al 2023, Low et al 2017, Ellinor et al 2012, Christopherson et al 2017a	GWAS	AF Type, PR Indices	AF and/or AFl, PR Interval , AF, Lone AF	EUR + AFR + EAS + HIS + BR, EUR, EUR + AFR + HIS + BR, EUR + EAS, EAS
HDGFL1	Ntalla et al 2020, Miyazawa et al 2023	GWAS	PR Indices, AF Type	PR Interval , AF	EUR + AFR + HIS + BR, EUR, EUR + EAS
HELZ	Yamada et al 2017	ExWAS	AF Type	AF	EAS

Target	Paper	Sequencing	Main Trait	Trait	Ancestry
HERC1	Nielsen et al 2018a	GWAS	AF Type	AF	EUR
HERPUD2	van Setten et al 2018, Van Setten et al 2019	GWAS	PR Indices	PR Interval	EUR, EUR + AFR
HIP1R	Roselli et al 2018 + Nielsen et al 2018a, Nielsen et al 2018a, Miyazawa et al 2023	GWAS	AF Type	AF and/or AFl, AF	EUR + AFR + EAS + HIS + BR, EUR, EUR + EAS
HLA-DPA1	Yamada et al 2017	ExWAS	AF Type	AF	EAS
HLX	Ntalla et al 2020	GWAS	PR Indices	PR Interval	EUR + AFR + HIS + BR, EUR
HMGA1	Miyazawa et al 2023	GWAS	AF Type	AF	EUR + EAS
HMGA2	Weng et al 2020	ExWAS	PR Indices	P wave duration	EUR + AFR + EAS + HIS
HNRNPC	Yamada et al 2017	ExWAS	AF Type	AF	EAS
HP	Thorolfsdottir et al 2017, Sinner et al 2014	WGS, Replication	AF Type	AF, Lone AF	EUR, EAS
HRURF	Yamada et al 2017	ExWAS	AF Type	AF	EAS
HSF2	Sinner et al 2014, Roselli et al 2018 + Nielsen et al 2018a, Roselli et al 2018, Nielsen et al 2018a, Miyazawa et al 2023, Low et al 2017, Christopherson et al 2017a	Replication, GWAS	AF Type	Lone AF, AF and/or AFl, AF	EUR, EUR + AFR + EAS + HIS + BR, EUR + EAS, EAS
HSPB2	Ntalla et al 2020	GWAS	PR Indices	PR Interval	EUR + AFR + HIS + BR, EUR
HSPG2	Roselli et al 2018 + Nielsen et al 2018a, Ntalla et al 2020, Nielsen et al 2018a, Miyazawa et al 2023	GWAS	AF Type, PR Indices	AF and/or AFl, PR Interval , AF	EUR + AFR + EAS + HIS + BR, EUR, EUR + AFR + HIS + BR, EUR + EAS
ID2	van Setten et al 2018, Seyerle et al 2018, Ntalla et al 2020, Lin et al 2018	GWAS, ExWAS	PR Indices	PR Interval, PR Interval	EUR, HIS, EUR + AFR + HIS + BR, EUR + AFR
IGF1R	Roselli et al 2018 + Nielsen et al 2018a, Roselli et al 2018, Nielsen et al 2018a, Miyazawa et al 2023	GWAS	AF Type	AF and/or AFl, AF	EUR + AFR + EAS + HIS + BR, EUR, EUR + EAS
IGFBP5	Ntalla et al 2020	GWAS	PR Indices	PR Interval	EUR
IL17D	Verweij et al 2014	GWAS	PR Indices	PR segment	EUR
IL24	Yamada et al 2017	ExWAS	AF Type	AF	EAS
IL25	van Setten et al 2018	GWAS	PR Indices	PR Interval	EUR
IL6R	Roselli et al 2018, Nielsen et al 2018a	GWAS	AF Type	AF and/or AFl, AF	EUR
IMPDH1	Yamada et al 2017	ExWAS	AF Type	AF	EAS
INKA2	Roselli et al 2018 + Nielsen et al 2018a, Roselli et al 2018, Miyazawa et al 2023, Low et al 2017	GWAS	AF Type	AF and/or AFl, AF	EUR + AFR + EAS + HIS + BR, EUR, EAS, EUR + EAS
IRF2BPL	Roselli et al 2018 + Nielsen et al 2018a, Roselli et al 2018, Nielsen et al 2018a, Miyazawa et al 2023	GWAS	AF Type	AF and/or AFl, AF	EUR + AFR + EAS + HIS + BR, EUR, EUR + EAS
IRF8	Ntalla et al 2020	GWAS	PR Indices	PR Interval	EUR, EUR + AFR + HIS + BR
IRX3	Ntalla et al 2020	GWAS	PR Indices	PR Interval	EUR + AFR + HIS + BR

Target	Paper	Sequencing	Main Trait	Trait	Ancestry
ISOC1	Ntalla et al 2020	GWAS	PR Indices	PR Interval	EUR + AFR + HIS + BR
ITGA2B	Gudbjartsson et al 2017	WGS	AF Type	Early onset AF	EUR
ITGA9	Ntalla et al 2020, Butler et al 2012, Van Setten et al 2019	GWAS	PR Indices	PR Interval , PR Interval	EUR, AFR, EUR + AFR
JAZF1	Weng et al 2020	ExWAS	PR Indices	P wave duration	EUR + AFR + EAS + HIS
JMJD1C	Roselli et al 2018 + Nielsen et al 2018a, Roselli et al 2018, Nielsen et al 2018a, Miyazawa et al 2023	GWAS	AF Type	AF and/or AFl, AF	EUR + AFR + EAS + HIS + BR, EUR, EUR + EAS
KATNAL1	Yamada et al 2017	ExWAS	AF Type	AF	EAS
KCND3	Verweij et al 2014, Roselli et al 2018, Ntalla et al 2020, Nielsen et al 2018a, Christopherson et al 2017b, Van Setten et al 2019	GWAS	PR Indices, AF Type	P wave duration, AF and/or AFl, PR Interval , AF, PTF, PR Interval	EUR, EUR + AFR + HIS + BR, EUR + AFR
KCNH2	Roselli et al 2018 + Nielsen et al 2018a, Roselli et al 2018, Nielsen et al 2018a, Miyazawa et al 2023	GWAS	AF Type	AF and/or AFl, AF	EUR + AFR + EAS + HIS + BR, EUR, EUR + EAS
KCNIP1	Tsai et al 2016	GWAS-CNV	AF Type	Lone AF	EAS
KCNIP2	Roselli et al 2018, Ntalla et al 2020	GWAS	AF Type, PR Indices	AF and/or AFl, PR Interval	EUR + AFR + EAS + HIS + BR, EUR
KCNJ2	Roselli et al 2018, Miyazawa et al 2023	GWAS	AF Type	AF and/or AFl, AF	EUR, EUR + AFR + EAS + HIS + BR, EUR + EAS
KCNJ5	Roselli et al 2018 + Nielsen et al 2018a, Roselli et al 2018, Nielsen et al 2018a, Miyazawa et al 2023, Christopherson et al 2017a	GWAS	AF Type	AF and/or AFl, AF	EUR + AFR + EAS + HIS + BR, EUR, EUR + EAS
KCNN2	Roselli et al 2018 + Nielsen et al 2018a, Roselli et al 2018, Nielsen et al 2018a, Miyazawa et al 2023, Christopherson et al 2017a	GWAS	AF Type	AF and/or AFl, AF	EUR + AFR + EAS + HIS + BR, EUR, EAS, EUR + EAS
KCNN3	Roselli et al 2018 + Nielsen et al 2018a, Roselli et al 2018, Nielsen et al 2018b, Nielsen et al 2018a, Miyazawa et al 2023, Low et al 2017, Ellinor et al 2012, Ellinor et al 2010, Christopherson et al 2017a	GWAS, ExWAS	AF Type	AF and/or AFl, AF, Lone AF	EUR + AFR + EAS + HIS + BR, EUR, EUR + EAS, EAS
KDM1B	Roselli et al 2018 + Nielsen et al 2018a, Roselli et al 2018, Nielsen et al 2018a, Miyazawa et al 2023	GWAS	AF Type	AF and/or AFl, AF	EUR + AFR + EAS + HIS + BR, EUR, EUR + EAS
KIF15	Yamada et al 2017	ExWAS	AF Type	AF	EAS
KIF3C	Roselli et al 2018 + Nielsen et al 2018a, Roselli et al 2018, Nielsen et al 2018a, Miyazawa et al 2023	GWAS	AF Type	AF and/or AFl, AF	EUR + AFR + EAS + HIS + BR, EUR, EUR + EAS
KIF7	Yamada et al 2017	ExWAS	AF Type	AF	EAS
KLF12	Ntalla et al 2020, Miyazawa et al 2023	GWAS	PR Indices, AF Type	RBINT PR Interval, AF	EUR + AFR + HIS + BR, EUR + EAS
KLF17	Yamada et al 2017	ExWAS	AF Type	AF	EAS

Target	Paper	Sequencing	Main Trait	Trait	Ancestry
KLF9	Ntalla et al 2020	GWAS	PR Indices	PR Interval	EUR + AFR + HIS + BR, EUR
KLHL26	Ntalla et al 2020	GWAS	PR Indices	PR Interval	EUR + AFR + HIS + BR, EUR
KLHL3	Christopherson et al 2017a	GWAS	AF Type	AF and/or AFl	EUR + AFR + EAS + HIS + BR
KLHL38	van Setten et al 2018, Roselli et al 2018, Ntalla et al 2020, Nielsen et al 2018a, Miyazawa et al 2023	GWAS	PR Indices, AF Type	and/or AFl, PR Interval , AF	EUR + AFR, EUR, EUR + AFR + HIS + BR, EAS, EUR + EAS
KLHL8	Ntalla et al 2020, Lin et al 2018	GWAS, ExWAS	PR Indices	PR Interval , PR Interval	EUR + AFR + HIS + BR, EUR + AFR
KNL1	Yamada et al 2017	ExWAS	AF Type	AF	EAS
KRTCAP2	van Setten et al 2018, Ntalla et al 2020	GWAS	PR Indices	PR Interval, PR Interval	EUR, EUR + AFR + HIS + BR
LAMB2	Ntalla et al 2020	GWAS	PR Indices	PR Interval	EUR + AFR + HIS + BR
LHX3	Roselli et al 2018 + Nielsen et al 2018a, Nielsen et al 2018a, Miyazawa et al 2023	GWAS	AF Type	AF and/or AFl, AF	EUR + AFR + EAS + HIS + BR, EUR, EUR + EAS
LIN54	Miyazawa et al 2023	GWAS	AF Type	AF	EUR + EAS
LLPH	Ntalla et al 2020	GWAS	PR Indices	PR Interval	EUR + AFR + HIS + BR, EUR
LMAN1	Ntalla et al 2020	GWAS	PR Indices	PR Interval	EUR + AFR + HIS + BR, EUR
LRCH1	Verweij et al 2014, van Setten et al 2018, Ntalla et al 2020, Van Setten et al 2019	GWAS	PR Indices	PR segment, PR Interval, PR Interval	EUR, EUR + AFR + HIS + BR, EUR + AFR
LRCOL1	Roselli et al 2018 + Nielsen et al 2018a, Nielsen et al 2018a	GWAS	AF Type	AF and/or AFl, AF	EUR + AFR + EAS + HIS + BR, EUR
LRIG1	van Setten et al 2018, Roselli et al 2018 + Nielsen et al 2018a, Roselli et al 2018, Nielsen et al 2018a, Miyazawa et al 2023	GWAS	PR Indices, AF Type	PR Interval, AF and/or AFl, AF	EUR + AFR, EUR + AFR + EAS + HIS + BR, EUR, EUR + EAS
LRMDA	Roselli et al 2018 + Nielsen et al 2018a, Roselli et al 2018, Ntalla et al 2020, Nielsen et al 2018a, Miyazawa et al 2023	GWAS	AF Type, PR Indices	AF and/or AFl, PR Interval , AF	EUR + AFR + EAS + HIS + BR, EUR, EAS, EUR + EAS
LRRC10	Nielsen et al 2018a, Miyazawa et al 2023	GWAS	AF Type	AF	EUR, EUR + EAS
LSM12	Gudbjartsson et al 2017	WGS	AF Type	Early onset AF	EUR
LSM3	Ntalla et al 2020	GWAS	PR Indices	PR Interval	EUR + AFR + HIS + BR, EUR
LYRM2	Yamada et al 2017	ExWAS	AF Type	AF	EAS
LYSMD3	Yamada et al 2017	ExWAS	AF Type	AF	EAS
MACF1	Ntalla et al 2020	GWAS	PR Indices	PR Interval	EUR + AFR + HIS + BR
MANBA	Miyazawa et al 2023	GWAS	AF Type	AF	EUR + EAS
MAP3K14	Gudbjartsson et al 2015	WGS	AF Type	Early onset AF	EUR
MAP7D1	Ntalla et al 2020	GWAS	PR Indices	PR Interval	EUR + AFR + HIS + BR
MAPRE2	Ntalla et al 2020	GWAS	PR Indices	PR Interval	EUR + AFR + HIS + BR, EUR

Target	Paper	Sequencing	Main Trait	Trait	Ancestry
MAPT	Roselli et al 2018, Miyazawa et al 2023	GWAS	AF Type	AF and/or AFl, AF	EUR + AFR + EAS + HIS + BR, EUR + EAS
MARCKS	Ntalla et al 2020	GWAS	PR Indices	PR Interval	EUR + AFR + HIS + BR, EUR
MARK3	Ntalla et al 2020	GWAS	PR Indices	PR Interval	EUR + AFR + HIS + BR
MC4R	Weng et al 2020	ExWAS	PR Indices	P wave duration	EUR + AFR + EAS + HIS
MCM10	Yamada et al 2017	ExWAS	AF Type	AF	EAS
MED13L	van Setten et al 2018, Ntalla et al 2020	GWAS	PR Indices	PR Interval, PR Interval	EUR
MEIS1	Verweij et al 2014, Smith et al 2011, Pfeufer et al 2010, Ntalla et al 2020, Lin et al 2018, Butler et al 2012, Van Setten et al 2019	GWAS, ExWAS	PR Indices	PR Interval, PR segment, PR Interval	EUR, AFR, EUR + AFR + HIS + BR, EUR + AFR
MELTF	Ntalla et al 2020	GWAS	PR Indices	PR Interval	EUR + AFR + HIS + BR, EUR
METRN	Miyazawa et al 2023	GWAS	AF Type	AF	EUR + EAS
MEX3C	Roselli et al 2018 + Nielsen et al 2018a, Nielsen et al 2018a	GWAS	AF Type	AF and/or AFl, AF	EUR + AFR + EAS + HIS + BR, EUR
MGAT1	Ahlberg et al 2020	GWAS	Left Atrium Indices	LAPEF	EUR
MGAT5	Yamada et al 2017	ExWAS	AF Type	AF	EAS
MICU2	Verweij et al 2014, Miyazawa et al 2023, Van Setten et al 2019	GWAS	PR Indices, AF Type	PR segment, AF, PR Interval	EUR, EUR + EAS, EUR + AFR
MKLN1	van Setten et al 2018, Ntalla et al 2020	GWAS	PR Indices	PR Interval, PR Interval	EUR, EUR + AFR + HIS + BR
MLLT3	Roselli et al 2018, Miyazawa et al 2023	GWAS	AF Type	AF and/or AFl, AF	EUR + AFR + EAS + HIS + BR, EUR + EAS
MOB1B	Miyazawa et al 2023	GWAS	AF Type	AF	EUR + EAS
MOBP	Lin et al 2018	ExWAS	PR Indices	PR Interval	EUR + AFR
MRPS34	Ntalla et al 2020	GWAS	PR Indices	PR Interval	EUR + AFR + HIS + BR, EUR
MSL2	Roselli et al 2018 + Nielsen et al 2018a, Nielsen et al 2018a, Miyazawa et al 2023	GWAS	AF Type	AF and/or AFl, AF	EUR + AFR + EAS + HIS + BR, EUR, EUR + EAS
MSRB1	Weng et al 2020, Thorolfsdottir et al 2018	ExWAS, GWAS	PR Indices, AF Type	P wave duration, AF	EUR + AFR + EAS + HIS, EUR
MSX2	Ntalla et al 2020	GWAS	PR Indices	PR Interval	EUR
MTNR1A	Ntalla et al 2020	GWAS	PR Indices	PR Interval	EUR
MTSS1	Roselli et al 2018, Ntalla et al 2020	GWAS	AF Type, PR Indices	AF and/or AFl, PR Interval	EUR, EUR + AFR + EAS + HIS + BR, EUR + AFR + HIS + BR
MTUS1	Ntalla et al 2020	GWAS	PR Indices	PR Interval	EUR + AFR + HIS + BR, EUR

Target	Paper	Sequencing	Main Trait	Trait	Ancestry
MUS81	Yamada et al 2017	ExWAS	AF Type	AF	EAS
MVK	Ntalla et al 2020	GWAS	PR Indices	PR Interval	EUR + AFR + HIS + BR, EUR
MYBPHL	van Setten et al 2018	GWAS	PR Indices	PR Interval	EUR
MYH11	Miyazawa et al 2023	GWAS	AF Type	AF	EUR + EAS
MYH6	Weng et al 2020, Thorolfsdottir et al 2017, Roselli et al 2018 + Nielsen et al 2018a, Roselli et al 2018, Ntalla et al 2020, Nielsen et al 2018a, Miyazawa et al 2023, Christopherson et al 2017b	ExWAS, WGS, GWAS	PR Indices, AF Type	P wave duration, AF, AF and/or AFl, PR Interval , PTF	EUR + AFR + EAS + HIS, EUR, EUR + AFR + EAS + HIS + BR, EUR + EAS, EUR + AFR
MYH7	Ntalla et al 2020	GWAS	PR Indices	PR Interval	EUR, EUR + AFR + HIS + BR
MYL4	Gudbjartsson et al 2017, Gudbjartsson et al 2015	WGS	AF Type	Early onset AF	EUR
MYO18B	Roselli et al 2018 + Nielsen et al 2018a, Nielsen et al 2018a, Miyazawa et al 2023, Ahlberg et al 2020	GWAS	AF Type, Left Atrium Indices	AF and/or AFl, AF, LATEF	EUR + AFR + EAS + HIS + BR, EUR, EUR + EAS
MYOCD	Roselli et al 2018 + Nielsen et al 2018a, Roselli et al 2018, Ntalla et al 2020, Nielsen et al 2018a, Miyazawa et al 2023, Lin et al 2018	GWAS, ExWAS	AF Type, PR Indices	Interval , AF, PR Interval	EUR + AFR + EAS + HIS + BR, EUR, EUR + EAS, EUR + AFR
MYOG	Roselli et al 2018 + Nielsen et al 2018a, Roselli et al 2018, Nielsen et al 2018a, Miyazawa et al 2023, Low et al 2017	GWAS	AF Type	AF and/or AFl, AF	EUR + AFR + EAS + HIS + BR, EUR, EAS, EUR + EAS
MYOZ1	Weng et al 2020	ExWAS	PR Indices	P wave duration	EUR + AFR + EAS + HIS, EUR
MYOZ2	Ntalla et al 2020	GWAS	PR Indices	PR Interval	EUR
N4BP2	Yamada et al 2017	ExWAS	AF Type	AF	EAS
NACA	Roselli et al 2018, Nielsen et al 2018a, Miyazawa et al 2023	GWAS	AF Type	AF and/or AFl, AF	EUR, EUR + EAS
NARS2	Yamada et al 2017	ExWAS	AF Type	AF	EAS
NCF1	Nielsen et al 2018a	GWAS	AF Type	AF	EUR
NCKAP5	Ntalla et al 2020	GWAS	PR Indices	PR Interval	EUR + AFR + HIS + BR, EUR
NCOR2	Miyazawa et al 2023	GWAS	AF Type	AF	EUR + EAS
NDE1	Ntalla et al 2020	GWAS	PR Indices	PR Interval	EUR
NDRG2	Ntalla et al 2020	GWAS	PR Indices	PR Interval	EUR + AFR + HIS + BR, EUR
NEBL	Miyazawa et al 2023, Low et al 2017	GWAS	AF Type	AF	EAS, EUR + EAS
NEURL	Nielsen et al 2018b	GWAS	AF Type	AF	EUR
NEURL1	Thorolfsdottir et al 2017, Sinner et al 2014, Roselli et al 2018 + Nielsen et al 2018a, Roselli et al 2018, Nielsen et al 2018a, Miyazawa et al 2023, Low et al 2017, Christopherson et al 2017a, Choi et al 2018	WGS, Replication, GWAS	AF Type	AF, Lone AF, AF and/or AFl, Early onset AF	EUR, EAS, EUR + AFR + EAS + HIS + BR, EUR + EAS
NEUROG2	Nielsen et al 2018a	GWAS	AF Type	AF	EUR
NFATC1	Miyazawa et al 2023	GWAS	AF Type	AF	EUR + EAS

Target	Paper	Sequencing	Main Trait	Trait	Ancestry
NFATC2	Yamada et al 2017	ExWAS	AF Type	AF	EAS
NFKBIA	Ntalla et al 2020	GWAS	PR Indices	PR Interval	EUR + AFR + HIS + BR, EUR
NFYB	Ntalla et al 2020	GWAS	PR Indices	PR Interval	EUR + AFR + HIS + BR, EUR
NGB	Yamada et al 2017	ExWAS	AF Type	AF	EAS
NKD2	Ntalla et al 2020	GWAS	PR Indices	PR Interval	EUR, EUR + AFR + HIS + BR
NKX2-5	Roselli et al 2018 + Nielsen et al 2018a, Roselli et al 2018, Ntalla et al 2020, Nielsen et al 2018a, Miyazawa et al 2023	GWAS	AF Type, PR Indices	AF and/or AFl, PR Interval , AF	EUR + AFR + EAS + HIS + BR, EUR, EUR + EAS
NLRX1	Yamada et al 2017	ExWAS	AF Type	AF	EAS
NMB	Christopherson et al 2017b	GWAS	PR Indices	PTF	EUR
NPR3	Ntalla et al 2020	GWAS	PR Indices	PR Interval	EUR, EUR + AFR + HIS + BR
NR2F1	Ntalla et al 2020	GWAS	PR Indices	PR Interval	EUR + AFR + HIS + BR, EUR
NR2F2	Ntalla et al 2020	GWAS	PR Indices	PR Interval	EUR + AFR + HIS + BR, EUR
NR3C1	Roselli et al 2018 + Nielsen et al 2018a, Roselli et al 2018, Ntalla et al 2020, Nielsen et al 2018a, Miyazawa et al 2023, Van Setten et al 2019	GWAS	AF Type, PR Indices	AF and/or AFl, PR Interval , AF, PR Interval	EUR + AFR + EAS + HIS + BR, EUR, EUR + AFR + HIS + BR, EUR + EAS, EUR + AFR
NRBF2	Yamada et al 2017	ExWAS	AF Type	AF	EAS
NSG2	Ntalla et al 2020	GWAS	PR Indices	PR Interval	EUR, EUR + AFR + HIS + BR
NTF3	Yamada et al 2017	ExWAS	AF Type	AF	EAS
NTMT2	Thorolfsdottir et al 2017, Roselli et al 2018 + Nielsen et al 2018a, Roselli et al 2018, Nielsen et al 2018b, Nielsen et al 2018a, Christopherson et al 2017a	WGS, GWAS	AF Type	AF, AF and/or AFl	EUR, EUR + AFR + EAS + HIS + BR
NUCKS1	Roselli et al 2018 + Nielsen et al 2018a, Roselli et al 2018, Nielsen et al 2018a, Miyazawa et al 2023	GWAS	AF Type	AF and/or AFl, AF	EUR + AFR + EAS + HIS + BR, EUR, EUR + EAS
OAS1	Miyazawa et al 2023	GWAS	AF Type	AF	EUR + EAS
OBSCN	van Setten et al 2018, Ntalla et al 2020	GWAS	PR Indices	PR Interval, PR Interval	EUR, EUR + AFR + HIS + BR
OPN1SW	Roselli et al 2018 + Nielsen et al 2018a, Nielsen et al 2018a	GWAS	AF Type	AF and/or AFl, AF	EUR + AFR + EAS + HIS + BR, EUR
OR4X2	Yamada et al 2017	ExWAS	AF Type	AF	EAS
ORC4	Miyazawa et al 2023	GWAS	AF Type	AF	EUR + EAS
OXR1	van Setten et al 2018, Ntalla et al 2020	GWAS	PR Indices	PR Interval, PR Interval	EUR
PALMD	Miyazawa et al 2023	GWAS	AF Type	AF	EUR + EAS
PAM	van Setten et al 2018, Ntalla et al 2020	GWAS	PR Indices	PR Interval, PR Interval	EUR, EUR + AFR + HIS + BR

Target	Paper	Sequencing	Main Trait	Trait	Ancestry
PAPPA	Miyazawa et al 2023	GWAS	AF Type	AF	EUR + EAS
PBX1	Emmert et al 2021	GWAS	AF Type	AF	EUR
PCDH18	Christopherson et al 2017b	GWAS	PR Indices	PTF	EUR + AFR
PCID2	Roselli et al 2018 + Nielsen et al 2018a, Nielsen et al 2018a, Miyazawa et al 2023	GWAS	AF Type	AF and/or AFl, AF	EUR + AFR + EAS + HIS + BR, EUR, EUR + EAS
PCNX3	Ntalla et al 2020	GWAS	PR Indices	PR Interval	EUR + AFR + HIS + BR
PDCD4	Roselli et al 2018 + Nielsen et al 2018a, Nielsen et al 2018a, Miyazawa et al 2023	GWAS	AF Type	AF and/or AFl, AF	EUR + AFR + EAS + HIS + BR, EUR, EUR + EAS
PDE3A	Ntalla et al 2020	GWAS	PR Indices	PR Interval	EUR + AFR + HIS + BR, EUR
PDE4B	Yamada et al 2017, Vasan et al 2007*	ExWAS, GWAS	AF Type, Left Atrium Indices	AF, LA diameter	EAS, EUR
PDLIM1	Ntalla et al 2020	GWAS	PR Indices	PR Interval	EUR
PDLIM5	Ntalla et al 2020	GWAS	PR Indices	PR Interval	EUR + AFR + HIS + BR, EUR
PDZRN3	van Setten et al 2018, Ntalla et al 2020, Van Setten et al 2019	GWAS	PR Indices	PR Interval, PR Interval	EUR, EUR + AFR + HIS + BR, EUR + AFR
PERM1	Miyazawa et al 2023	GWAS	AF Type	AF	EUR + EAS
PFAS	Yamada et al 2017	ExWAS	AF Type	AF	EAS
PFDN1	Miyazawa et al 2023	GWAS	AF Type	AF	EUR + EAS
PGP	Nielsen et al 2018a	GWAS	AF Type	AF	EUR
PHETA1	Sinner et al 2014, Low et al 2017	Replication, GWAS	AF Type	Lone AF, AF	EAS
PHF20L1	Ntalla et al 2020	GWAS	PR Indices	RBINT PR Interval	EUR + AFR + HIS + BR
PHF5A	Ntalla et al 2020	GWAS	PR Indices	PR Interval	EUR + AFR + HIS + BR, EUR
PHIP	Ntalla et al 2020	GWAS	PR Indices	RBINT PR Interval	EUR + AFR + HIS + BR
PHLDA1	Roselli et al 2018 + Nielsen et al 2018a, Roselli et al 2018, Nielsen et al 2018a, Miyazawa et al 2023	GWAS	AF Type	AF and/or AFl, AF	EUR + AFR + EAS + HIS + BR, EUR, EUR + EAS
PHLDB2	Nielsen et al 2018a, Miyazawa et al 2023	GWAS	AF Type	AF	EUR, EUR + EAS
PITX2	Weng et al 2020, Wang et al 2023, Thorolfsdottir et al 2017, Sinner et al 2014, Roselli et al 2018 + Nielsen et al 2018a, Roselli et al 2018, Nielsen et al 2018b, Nielsen et al 2018a, Miyazawa et al 2023, Low et al 2017, Ellinor et al 2012, Ellinor et al 2010, Christopherson et al 2017a, Choi et al 2018, Benjamin et al 2009, Baldassari et al 2020, Gudbjartsson et al 2007	ExWAS, WGS, Replication, GWAS	PR Indices, AF Type	P wave duration, AF, Lone AF, AF and/or AFl, Early onset AF	EUR + AFR + HIS + EAS, EUR + AFR + EAS + HIS, EUR, EUR + HIS + AFR + EAS + SAS + MID + OTR, EAS, EUR + AFR + EAS + HIS + BR, AFR, EUR + EAS, EUR + HIS + AFR
PKD1L1	Yamada et al 2017	ExWAS	AF Type	AF	EAS
PKP1	Weng et al 2020	ExWAS	PR Indices	P wave duration	EUR + AFR + EAS + HIS

Target	Paper	Sequencing	Main Trait	Trait	Ancestry
PLCD3	Gudbjartsson et al 2017, Gudbjartsson et al 2015	WGS	AF Type	Early onset AF	EUR
PLEC	Thorolfsdottir et al 2017, Ntalla et al 2020	WGS, GWAS	AF Type, PR Indices	AF, PR Interval	EUR, EUR + AFR + HIS + BR
PLEKHA3	Roselli et al 2018 + Nielsen et al 2018a, Roselli et al 2018, Nielsen et al 2018b, Nielsen et al 2018a, Miyazawa et al 2023, Christopherson et al 2017a, Ahlberg et al 2020	GWAS	AF Type, Left Atrium Indices	AF and/or AFl, AF, LAPEF	EUR + AFR + EAS + HIS + BR, EUR, EUR + EAS
PLN	van Setten et al 2018, Roselli et al 2018 + Nielsen et al 2018a, Roselli et al 2018, Ntalla et al 2020, Nielsen et al 2018a, Lin et al 2018, Christopherson et al 2017a, Van Setten et al 2019	GWAS, ExWAS	PR Indices, AF Type	PR Interval, AF and/or AFl, PR Interval , AF	EUR + AFR, EUR + AFR + EAS + HIS + BR, EUR, EUR + AFR + HIS + BR
PLPP7	Ntalla et al 2020	GWAS	PR Indices	PR Interval	EUR + AFR + HIS + BR, EUR
PMFBP1	Roselli et al 2018 + Nielsen et al 2018a, Roselli et al 2018, Nielsen et al 2018b, Nielsen et al 2018a, Miyazawa et al 2023, Low et al 2017, Gudbjartsson et al 2009, Ellinor et al 2012, Christopherson et al 2017a, Choi et al 2018, Benjamin et al 2009	GWAS, ExWAS, WGS	AF Type	AF and/or AFl, AF, Lone AF, Early onset AF	EUR + AFR + EAS + HIS + BR, EUR, EAS, EUR + EAS
POLR2A	Miyazawa et al 2023	GWAS	AF Type	AF	EUR + EAS
POLR2M	Yamada et al 2017	ExWAS	AF Type	AF	EAS
POT1	Yamada et al 2017	ExWAS	AF Type	AF	EAS
PPARGC1A	Ntalla et al 2020	GWAS	PR Indices	PR Interval	EUR, EUR + AFR + HIS + BR
PRKAB2	Ntalla et al 2020	GWAS	PR Indices	PR Interval	EUR
PRKCA	Ntalla et al 2020	GWAS	PR Indices	PR Interval	EUR + AFR + HIS + BR
PRPS1L1	Ntalla et al 2020	GWAS	PR Indices	PR Interval	EUR + AFR + HIS + BR, EUR
PRRX1	Roselli et al 2018, Ntalla et al 2020, Nielsen et al 2018a, Miyazawa et al 2023, Low et al 2017, Ellinor et al 2012, Christopherson et al 2017a, Choi et al 2018	GWAS, WGS	AF Type, PR Indices	AF and/or AFl, PR Interval , AF, Lone AF, Early onset AF	EUR, EUR + AFR + EAS + HIS + BR, EUR + AFR + HIS + BR, EAS, EUR + EAS
PSMB7	Roselli et al 2018	GWAS	AF Type	AF and/or AFl	EUR + AFR + EAS + HIS + BR
PSMG1	Yamada et al 2017	ExWAS	AF Type	AF	EAS
PTCHD1	Miyazawa et al 2023	GWAS	AF Type	AF	EAS, EUR + EAS
PTGES3	Roselli et al 2018 + Nielsen et al 2018a, Roselli et al 2018	GWAS	AF Type	AF and/or AFl	EUR + AFR + EAS + HIS + BR, EUR
PTK2	Ntalla et al 2020	GWAS	PR Indices	PR Interval	EUR + AFR + HIS + BR, EUR
QKI	Ntalla et al 2020	GWAS	PR Indices	PR Interval	EUR + AFR + HIS + BR, EUR
RAB1A	Miyazawa et al 2023	GWAS	AF Type	AF	EAS
RAP2B	Ntalla et al 2020	GWAS	PR Indices	PR Interval	EUR + AFR + HIS + BR, EUR
RAPGEF5	Yamada et al 2017	ExWAS	AF Type	AF	EAS
RARRES1	Yamada et al 2017	ExWAS	AF Type	AF	EAS

Target	Paper	Sequencing	Main Trait	Trait	Ancestry
RASGRF1	Yamada et al 2017	ExWAS	AF Type	AF	EAS
RBPMS	Ntalla et al 2020	GWAS	PR Indices	PR Interval	EUR + AFR + HIS + BR
RBPMS2	Ntalla et al 2020	GWAS	PR Indices	PR Interval	EUR + AFR + HIS + BR, EUR
RCAN2	Ntalla et al 2020	GWAS	PR Indices	PR Interval	EUR + AFR + HIS + BR, EUR
REEP1	Roselli et al 2018 + Nielsen et al 2018a, Roselli et al 2018, Nielsen et al 2018a, Miyazawa et al 2023	GWAS	AF Type	AF and/or AFl, AF	EUR + AFR + EAS + HIS + BR, EUR, EUR + EAS
RHBDL2	Miyazawa et al 2023	GWAS	AF Type	AF	EUR + EAS
RIC8B	Ntalla et al 2020	GWAS	PR Indices	PR Interval	EUR + AFR + HIS + BR, EUR
RNF24	Ntalla et al 2020	GWAS	PR Indices	PR Interval	EUR + AFR + HIS + BR, EUR
RPL32	Miyazawa et al 2023	GWAS	AF Type	AF	EAS
RPL3L	Thorolfsdottir et al 2018, Roselli et al 2018, Nielsen et al 2018a, Miyazawa et al 2023	GWAS	AF Type	AF, AF and/or AFl	EUR, EUR + EAS
RPRML	Roselli et al 2018 + Nielsen et al 2018a, Roselli et al 2018, Miyazawa et al 2023	GWAS	AF Type	AF and/or AFl, AF	EUR + AFR + EAS + HIS + BR, EUR, EUR + EAS
RPS2	Roselli et al 2018 + Nielsen et al 2018a, Roselli et al 2018	GWAS	AF Type	AF and/or AFl	EUR + AFR + EAS + HIS + BR, EUR
RTKN2	Yamada et al 2017	ExWAS	AF Type	AF	EAS
RUNDC3A	Gudbjartsson et al 2017	WGS	AF Type	Early onset AF	EUR
RYR2	Ntalla et al 2020	GWAS	PR Indices	PR Interval	EUR + AFR + HIS + BR, EUR
SALL4	Yamada et al 2017	ExWAS	AF Type	AF	EAS
SAMD8	Ntalla et al 2020	GWAS	PR Indices	PR Interval	EUR
SCN10A	Weng et al 2020, Smith et al 2011, Pfeufer et al 2010, Ntalla et al 2020, Christopherson et al 2017a, Chambers et al 2010, Sano et al 2014, Van Setten et al 2019	ExWAS, GWAS, Replication	PR Indices, AF Type	P wave duration, PR Interval, PR Interval , AF and/or AFl	EUR + AFR + EAS + HIS, EUR, AFR, EUR + AFR + EAS + HIS + BR, SAS, EAS, EUR + AFR
SCN5A	Weng et al 2020, Verweij et al 2014, Smith et al 2011, Seyerle et al 2018, Roselli et al 2018 + Nielsen et al 2018a, Roselli et al 2018, Pfeufer et al 2010, Ntalla et al 2020, Nielsen et al 2018a, Miyazawa et al 2023, Lin et al 2018, Holm et al 2010*, Christopherson et al 2017b, Butler et al 2012, Van Setten et al 2019	ExWAS, GWAS	PR Indices, AF Type	P wave duration, PR Interval, PR segment, AF and/or AFl, PR Interval , AF	EUR + AFR + HIS + EAS, AFR, EUR, HIS, EUR + AFR + EAS + HIS + BR, EUR + AFR + HIS + BR, EUR + EAS, EUR + AFR
SCYL2	Yamada et al 2017	ExWAS	AF Type	AF	EAS
SCYL3	Miyazawa et al 2023	GWAS	AF Type	AF	EAS
SDF2L1	Miyazawa et al 2023	GWAS	AF Type	AF	EUR + EAS
SEC11A	Baldassari et al 2020	GWAS	PR Indices	PR segment	EUR + HIS + AFR
SELENOM	Ntalla et al 2020	GWAS	PR Indices	RBINT PR Interval	EUR + AFR + HIS + BR

Target	Paper	Sequencing	Main Trait	Trait	Ancestry
SEMA3A	Ntalla et al 2020	GWAS	PR Indices	PR Interval	EUR + AFR + HIS + BR, EUR
SEMA6A	Yamada et al 2017	ExWAS	AF Type	AF	EAS
SENP2	van Setten et al 2018, Ntalla et al 2020	GWAS	PR Indices	PR Interval, PR Interval	EUR, EUR + AFR + HIS + BR
SEPHS1	Ahlberg et al 2020	GWAS	Left Atrium Indices	LATEF	EUR
SESN3	Miyazawa et al 2023	GWAS	AF Type	AF	EUR + EAS
SETD6	Ntalla et al 2020	GWAS	PR Indices	PR Interval	EUR + AFR + HIS + BR, EUR
SETD7	Yamada et al 2017	ExWAS	AF Type	AF	EAS
SFR1	Ntalla et al 2020	GWAS	PR Indices	PR Interval	EUR
SGCG	Roselli et al 2018 + Nielsen et al 2018a, Roselli et al 2018, Nielsen et al 2018a, Miyazawa et al 2023	GWAS	AF Type	AF and/or AFl, AF	EUR + AFR + EAS + HIS + BR, EUR, EUR + EAS
SH3BGRL3	Ntalla et al 2020	GWAS	PR Indices	PR Interval	EUR
SH3PXD2A	van Setten et al 2018, Roselli et al 2018, Ntalla et al 2020, Nielsen et al 2018a, Low et al 2017, Christopherson et al 2017a	GWAS	PR Indices, AF Type	PR Interval, AF and/or AFl, PR Interval , AF	EUR, EUR + AFR + HIS + BR, EAS, EUR + AFR + EAS + HIS + BR
SIPA1L1	van Setten et al 2018, Ntalla et al 2020, Lin et al 2018, Van Setten et al 2019	GWAS, ExWAS	PR Indices	PR Interval, PR Interval	EUR, EUR + AFR + HIS + BR, EUR + AFR
SIPA1L2	Ntalla et al 2020	GWAS	PR Indices	PR Interval	EUR + AFR + HIS + BR, EUR
SIRT1	Roselli et al 2018 + Nielsen et al 2018a, Nielsen et al 2018a	GWAS	AF Type	AF and/or AFl, AF	EUR + AFR + EAS + HIS + BR, EUR
SIX5	Ahlberg et al 2020	GWAS	Left Atrium Indices	LAAEF	EUR
SKI	van Setten et al 2018	GWAS	PR Indices	PR Interval	EUR
SLA2	Yamada et al 2017	ExWAS	AF Type	AF	EAS
SLAMF7	Yamada et al 2017	ExWAS	AF Type	AF	EAS
SLC12A7	Ntalla et al 2020	GWAS	PR Indices	PR Interval	EUR
SLC15A5	Yamada et al 2017	ExWAS	AF Type	AF	EAS
SLC18A3	Yamada et al 2017	ExWAS	AF Type	AF	EAS
SLC1A7	Yamada et al 2017	ExWAS	AF Type	AF	EAS
SLC22A24	Yamada et al 2017	ExWAS	AF Type	AF	EAS
SLC25A26	Ntalla et al 2020	GWAS	PR Indices	PR Interval	EUR + AFR + HIS + BR
SLC35F1	Thorolfsdottir et al 2017	WGS	AF Type	AF	EUR
SLC4A7	Yamada et al 2017	ExWAS	AF Type	AF	EAS

Target	Paper	Sequencing	Main Trait	Trait	Ancestry
SLC50A1	Lin et al 2018	ExWAS	PR Indices	PR Interval	EUR + AFR
SLC6A15	Ntalla et al 2020	GWAS	PR Indices	PR Interval	EUR + AFR + HIS + BR, EUR
SLC6A4	Ntalla et al 2020	GWAS	PR Indices	PR Interval	EUR
SLC8A1	Seyerle et al 2018, Ntalla et al 2020, Hong et al	GWAS	PR Indices	PR Interval, PR Interval	HIS, EUR + AFR + HIS + BR, EAS
SLC9B1	Roselli et al 2018 + Nielsen et al 2018a, Roselli et al 2018, Nielsen et al 2018a	GWAS	AF Type	AF and/or AFl, AF	EUR + AFR + EAS + HIS + BR, EUR
SLCO4A1	Roselli et al 2018, Ntalla et al 2020	GWAS	AF Type, PR Indices	AF and/or AFl, PR Interval	EUR + AFR + EAS + HIS + BR, EUR + AFR + HIS + BR
SLCO6A1	Yamada et al 2017	ExWAS	AF Type	AF	EAS
SLFNL1	Roselli et al 2018 + Nielsen et al 2018a, Nielsen et al 2018a, Miyazawa et al 2023	GWAS	AF Type	AF and/or AFl, AF	EUR + AFR + EAS + HIS + BR, EUR, EUR + EAS
SLIT3	Roselli et al 2018 + Nielsen et al 2018a, Nielsen et al 2018a, Miyazawa et al 2023	GWAS	AF Type	AF and/or AFl, AF	EUR + AFR + EAS + HIS + BR, EUR, EUR + EAS
SMAD7	Roselli et al 2018 + Nielsen et al 2018a, Roselli et al 2018, Nielsen et al 2018a, Miyazawa et al 2023	GWAS	AF Type	AF and/or AFl, AF	EUR + AFR + EAS + HIS + BR, EUR, EUR + EAS
SMARCB1	Ntalla et al 2020	GWAS	PR Indices	PR Interval	EUR
SMYD3	Ntalla et al 2020	GWAS	PR Indices	PR Interval	EUR + AFR + HIS + BR, EUR
SNAPC1	Yamada et al 2017	ExWAS	AF Type	AF	EAS
SNRNP27	Roselli et al 2018 + Nielsen et al 2018a, Roselli et al 2018, Nielsen et al 2018a, Miyazawa et al 2023	GWAS	AF Type	AF and/or AFl, AF	EUR + AFR + EAS + HIS + BR, EUR, EUR + EAS
SNRNP48	Ahlberg et al 2020	GWAS	Left Atrium Indices	LAAEF	EUR
SNRPD2	Ahlberg et al 2020	GWAS	Left Atrium Indices	LAminVol	EUR
SNX19	Yamada et al 2017	ExWAS	AF Type	AF	EAS
SORBS1	van Setten et al 2018, Lin et al 2018	GWAS, ExWAS	PR Indices	PR Interval	EUR, EUR + AFR
SORL1	Roselli et al 2018 + Nielsen et al 2018a, Roselli et al 2018, Miyazawa et al 2023	GWAS	AF Type	AF and/or AFl, AF	EUR + AFR + EAS + HIS + BR, EUR, EUR + EAS
SORT1	Ntalla et al 2020	GWAS	PR Indices	PR Interval	EUR + AFR + HIS + BR
SOX11	Ntalla et al 2020	GWAS	PR Indices	PR Interval	EUR + AFR + HIS + BR, EUR

Target	Paper	Sequencing	Main Trait	Trait	Ancestry
SOX5	Weng et al 2020, Verweij et al 2014, Seyerle et al 2018, Roselli et al 2018 + Nielsen et al 2018a, Roselli et al 2018, Pfeufer et al 2010, Ntalla et al 2020, Nielsen et al 2018a, Miyazawa et al 2023, Lin et al 2018, Christopherson et al 2017a, Choi et al 2018, Van Setten et al 2019	ExWAS, GWAS, WGS	PR Indices, AF Type	P wave duration, PR Interval, AF and/or AFl, PR Interval , AF, Early onset AF	EUR + AFR + EAS + HIS, EUR, HIS, EUR + AFR + EAS + HIS + BR, EUR + AFR + HIS + BR, EAS, EUR + EAS, EUR + AFR
SPATA31D1	Ntalla et al 2020	GWAS	PR Indices	PR Interval	EUR + AFR + HIS + BR, EUR
SPATS2L	Roselli et al 2018 + Nielsen et al 2018a, Roselli et al 2018, Nielsen et al 2018a, Miyazawa et al 2023	GWAS	AF Type	AF and/or AFl, AF	EUR + AFR + EAS + HIS + BR, EUR, EAS, EUR + EAS
SPDYE21	Yamada et al 2017	ExWAS	AF Type	AF	EAS
SPEN	Miyazawa et al 2023	GWAS	AF Type	AF	EUR + EAS
SPIDR	Yamada et al 2017	ExWAS	AF Type	AF	EAS
SPON1	Miyazawa et al 2023, Christopherson et al 2017b	GWAS	AF Type, PR Indices	AF, PTF	EUR + EAS, AFR
SRD5A3	Ntalla et al 2020	GWAS	PR Indices	PR Interval	EUR + AFR + HIS + BR, EUR
SRGAP1	Ntalla et al 2020	GWAS	PR Indices	PR Interval	EUR + AFR + HIS + BR, EUR
SRL	Ntalla et al 2020	GWAS	PR Indices	PR Interval	EUR + AFR + HIS + BR
SSH2	Ntalla et al 2020	GWAS	PR Indices	PR Interval	EUR + AFR + HIS + BR
STARD4	Ntalla et al 2020	GWAS	PR Indices	PR Interval	EUR + AFR + HIS + BR, EUR
STIM1	Miyazawa et al 2023	GWAS	AF Type	AF	EUR + EAS
STRN	Ntalla et al 2020	GWAS	PR Indices	PR Interval	EUR + AFR + HIS + BR, EUR
SULF2	Ellinor et al 2010	GWAS	AF Type	Lone AF	EUR
SYDE2	Yamada et al 2017	ExWAS	AF Type	AF	EAS
SYK	Low et al 2017	GWAS	AF Type	AF	EAS
SYNE1	Miyazawa et al 2023	GWAS	AF Type	AF	EAS
SYNE2	Ntalla et al 2020	GWAS	PR Indices	PR Interval	EUR + AFR + HIS + BR, EUR
SYNE3	Ntalla et al 2020	GWAS	PR Indices	PR Interval	EUR + AFR + HIS + BR, EUR
SYNPO2L	Thorolfsdottir et al 2017, Roselli et al 2018 + Nielsen et al 2018a, Roselli et al 2018, Ntalla et al 2020, Nielsen et al 2018a, Miyazawa et al 2023, Low et al 2017, Lin et al 2018, Ellinor et al 2012, Christopherson et al 2017a	WGS, GWAS, ExWAS	AF Type, PR Indices	AF, AF and/or AFl, PR Interval , PR Interval, Lone AF	EUR, EUR + AFR + EAS + HIS + BR, EUR + AFR + HIS + BR, EUR + EAS, EAS, EUR + AFR
TAB2	Roselli et al 2018 + Nielsen et al 2018a, Roselli et al 2018, Ntalla et al 2020, Nielsen et al 2018a, Miyazawa et al 2023	GWAS	AF Type, PR Indices	RBINT PR Interval, AF	EUR + AFR + EAS + HIS + BR, EUR, EUR + AFR + HIS + BR, EUR + EAS
TBX20	Ntalla et al 2020	GWAS	PR Indices	PR Interval	EUR, EUR + AFR + HIS + BR

Target	Paper	Sequencing	Main Trait	Trait	Ancestry
TBX3	Verweij et al 2014, Roselli et al 2018, Pfeufer et al 2010, Ntalla et al 2020, Lin et al 2018, Van Setten et al 2019	GWAS, ExWAS	PR Indices, AF Type	PR Interval, PR segment, AF and/or AFl, PR Interval	EUR, EUR + AFR + EAS + HIS + BR, EUR + AFR + HIS + BR, EUR + AFR
TBX5	Weng et al 2020, Smith et al 2011, Sinner et al 2014, Roselli et al 2018 + Nielsen et al 2018a, Roselli et al 2018, Ntalla et al 2020, Nielsen et al 2018a, Miyazawa et al 2023, Low et al 2017, Lin et al 2018, Holm et al 2010*, Christopherson et al 2017b, Christopherson et al 2017a, Butler et al 2012	ExWAS, GWAS, Replication	PR Indices, AF Type	P wave duration, PR Interval, Lone AF, AF and/or AFl, PR Interval , RBINT PR Interval, AF	EUR + AFR + EAS + HIS, EUR, AFR, EUR + AFR + EAS + HIS + BR, EAS, EUR + EAS, EUR + AFR
TCF21	Weng et al 2020, Ntalla et al 2020	ExWAS, GWAS	PR Indices	P wave duration, PR Interval	EUR + AFR + EAS + HIS, EUR + AFR + HIS + BR, EUR
TESC	Ntalla et al 2020	GWAS	PR Indices	PR Interval	EUR + AFR + HIS + BR, EUR
TFAP4	Ntalla et al 2020	GWAS	PR Indices	PR Interval	EUR
TFEB	Ntalla et al 2020	GWAS	PR Indices	PR Interval	EUR + AFR + HIS + BR, EUR
TGIF1	Yamada et al 2017	ExWAS	AF Type	AF	EAS
TGM2	Ntalla et al 2020, Miyazawa et al 2023	GWAS	PR Indices, AF Type	PR Interval , AF	EUR + AFR + HIS + BR, EUR, EUR + EAS
THRAP3	Ntalla et al 2020	GWAS	PR Indices	PR Interval	EUR
THRB	Roselli et al 2018 + Nielsen et al 2018a, Ntalla et al 2020, Nielsen et al 2018a, Miyazawa et al 2023	GWAS	AF Type, PR Indices	AF and/or AFl, PR Interval , AF	EUR + AFR + EAS + HIS + BR, EUR, EUR + AFR + HIS + BR, EUR + EAS
THSD4	Ntalla et al 2020	GWAS	PR Indices	RBINT PR Interval	EUR + AFR + HIS + BR
TLE3	van Setten et al 2018, Roselli et al 2018, Ntalla et al 2020, Miyazawa et al 2023	GWAS	PR Indices, AF Type	and/or AFl, PR Interval , AF	EUR, EUR + AFR + EAS + HIS + BR, EUR + AFR + HIS + BR, EUR + EAS
TM2D1	Ntalla et al 2020	GWAS	PR Indices	PR Interval	EUR + AFR + HIS + BR
TMCO3	Yamada et al 2017	ExWAS	AF Type	AF	EAS
TMEM182	van Setten et al 2018, Ntalla et al 2020	GWAS	PR Indices	PR Interval, PR Interval	EUR, EUR + AFR + HIS + BR
TMEM245	Yamada et al 2017	ExWAS	AF Type	AF	EAS
TMEM26	Ntalla et al 2020	GWAS	PR Indices	PR Interval	EUR + AFR + HIS + BR, EUR
TMEM40	Low et al 2017	GWAS	AF Type	AF	EAS
TMEM72	Ntalla et al 2020	GWAS	PR Indices	RBINT PR Interval	EUR + AFR + HIS + BR
TMT1B	Nielsen et al 2018a	GWAS	AF Type	AF	EUR
TMX4	Yamada et al 2017	ExWAS	AF Type	AF	EAS
TNFSF12	Roselli et al 2018 + Nielsen et al 2018a, Roselli et al 2018	GWAS	AF Type	AF and/or AFl	EUR + AFR + EAS + HIS + BR, EUR

Target	Paper	Sequencing	Main Trait	Trait	Ancestry
TNFSF12-TNFSF13	Nielsen et al 2018a	GWAS	AF Type	AF	EUR
TNFSF13	Yamada et al 2017	ExWAS	AF Type	AF	EAS
TNNI3	Yamada et al 2017	ExWAS	AF Type	AF	EAS
TP53INP1	Yamada et al 2017	ExWAS	AF Type	AF	EAS
TRAK1	Ntalla et al 2020	GWAS	PR Indices	PR Interval	EUR + AFR + HIS + BR, EUR
TRAM2	Ntalla et al 2020	GWAS	PR Indices	RBINT PR Interval	EUR
TRDN	Ntalla et al 2020	GWAS	PR Indices	PR Interval	EUR
TRIM32	Ntalla et al 2020	GWAS	PR Indices	PR Interval	EUR + AFR + HIS + BR
TRIM40	Yamada et al 2017	ExWAS	AF Type	AF	EAS
TRPM2	Yamada et al 2017	ExWAS	AF Type	AF	EAS
TSC22D2	Ntalla et al 2020	GWAS	PR Indices	PR Interval	EUR + AFR + HIS + BR, EUR
TTC29	Yamada et al 2017	ExWAS	AF Type	AF	EAS
TTC39A	van Setten et al 2018	GWAS	PR Indices	PR Interval	EUR
TTN	Weng et al 2020, van Setten et al 2018, Ntalla et al 2020, Lin et al 2018	ExWAS, GWAS	PR Indices	P wave duration, PR Interval, PR Interval	EUR + AFR + EAS + HIS, EUR, EUR + AFR + HIS + BR, EUR + AFR
TUBA8	Roselli et al 2018 + Nielsen et al 2018a, Roselli et al 2018, Nielsen et al 2018a, Miyazawa et al 2023	GWAS	AF Type	AF and/or AFl, AF	EUR + AFR + EAS + HIS + BR, EUR, EUR + EAS
TUBB3	Yamada et al 2017	ExWAS	AF Type	AF	EAS
UBE4B	Roselli et al 2018	GWAS	AF Type	AF and/or AFl	EUR, EUR + AFR + EAS + HIS + BR
UQCRB	Ahlberg et al 2020	GWAS	Left Atrium Indices	LAAEF	EUR
URB2	Yamada et al 2017	ExWAS	AF Type	AF	EAS
USP3	Roselli et al 2018 + Nielsen et al 2018a, Roselli et al 2018	GWAS	AF Type	AF and/or AFl	EUR + AFR + EAS + HIS + BR, EUR
USP34	Miyazawa et al 2023	GWAS	AF Type	AF	EUR + EAS
UTP4	Yamada et al 2017	ExWAS	AF Type	AF	EAS
VILL	Ntalla et al 2020	GWAS	PR Indices	PR Interval	EUR
VPS13C	Yamada et al 2017	ExWAS	AF Type	AF	EAS
VPS13D	Yamada et al 2017	ExWAS	AF Type	AF	EAS
VWDE	Yamada et al 2017	ExWAS	AF Type	AF	EAS
WDR1	Roselli et al 2018, Miyazawa et al 2023	GWAS	AF Type	AF and/or AFl, AF	EUR + AFR + EAS + HIS + BR, EUR + EAS
WDR27	Yamada et al 2017	ExWAS	AF Type	AF	EAS

Target	Paper	Sequencing	Main Trait	Trait	Ancestry
WIPF1	Roselli et al 2018 + Nielsen et al 2018a, Roselli et al 2018, Nielsen et al 2018a, Miyazawa et al 2023	GWAS	AF Type	AF and/or AFl, AF	EUR + AFR + EAS + HIS + BR, EUR, EUR + EAS
WNT11	Pfeufer et al 2010, Ntalla et al 2020	GWAS	PR Indices	PR Interval, PR Interval	EUR, EUR + AFR + HIS + BR
WNT3	Nielsen et al 2018a	GWAS	AF Type	AF	EUR
WNT8A	Roselli et al 2018 + Nielsen et al 2018a, Roselli et al 2018, Nielsen et al 2018a, Miyazawa et al 2023, Ellinor et al 2012	GWAS	AF Type	AF and/or AFl, AF, Lone AF	EUR + AFR + EAS + HIS + BR, EUR, EUR + EAS
WRNIP1	Ntalla et al 2020, Christopherson et al 2017b	GWAS	PR Indices	PR Interval , PTF	EUR + AFR + HIS + BR, EUR, EUR + AFR
WT1	Ntalla et al 2020	GWAS	PR Indices	PR Interval	EUR + AFR + HIS + BR
XPO1	Roselli et al 2018 + Nielsen et al 2018a, Roselli et al 2018	GWAS	AF Type	AF and/or AFl	EUR + AFR + EAS + HIS + BR, EUR
XPO4	Ntalla et al 2020	GWAS	PR Indices	PR Interval	EUR
XPO7	Roselli et al 2018 + Nielsen et al 2018a, Roselli et al 2018, Ntalla et al 2020, Nielsen et al 2018a, Miyazawa et al 2023	GWAS	AF Type, PR Indices	AF and/or AFl, PR Interval , AF	EUR + AFR + EAS + HIS + BR, EUR, EUR + AFR + HIS + BR, EUR + EAS
XXYLT1	Roselli et al 2018 + Nielsen et al 2018a, Nielsen et al 2018a, Miyazawa et al 2023	GWAS	AF Type	AF and/or AFl, AF	EUR + AFR + EAS + HIS + BR, EUR, EUR + EAS
XYLB	Smith et al 2009*, Ntalla et al 2020, Lin et al 2018, Van Setten et al 2019	GWAS, ExWAS	PR Indices	PR Interval, PR Interval	Micronesian, EUR, EUR + AFR
YARS2	Roselli et al 2018 + Nielsen et al 2018a, Roselli et al 2018, Nielsen et al 2018a, Miyazawa et al 2023	GWAS	AF Type	AF and/or AFl, AF	EUR + AFR + EAS + HIS + BR, EUR, EUR + EAS
YWHAE	Roselli et al 2018 + Nielsen et al 2018a, Nielsen et al 2018a, Miyazawa et al 2023	GWAS	AF Type	AF and/or AFl, AF	EUR + AFR + EAS + HIS + BR, EUR, EUR + EAS
ZC3H15	Ntalla et al 2020	GWAS	PR Indices	PR Interval	EUR + AFR + HIS + BR
ZCCHC24	Ntalla et al 2020	GWAS	PR Indices	PR Interval	EUR
ZDHHC20	van Setten et al 2018, Ntalla et al 2020	GWAS	PR Indices	PR Interval, PR Interval	EUR, EUR + AFR + HIS + BR
ZEB2	Roselli et al 2018 + Nielsen et al 2018a, Ntalla et al 2020, Nielsen et al 2018a, Miyazawa et al 2023	GWAS	AF Type, PR Indices	AF and/or AFl, PR Interval , AF	EUR + AFR + EAS + HIS + BR, EUR, EUR + AFR + HIS + BR, EUR + EAS
ZFPM1	Ntalla et al 2020	GWAS	PR Indices	PR Interval	EUR + AFR + HIS + BR, EUR
ZFPM2	Ntalla et al 2020	GWAS	PR Indices	PR Interval	EUR, EUR + AFR + HIS + BR
ZIC3	Miyazawa et al 2023	GWAS	AF Type	AF	EUR + EAS
ZMIZ1	Ntalla et al 2020, Miyazawa et al 2023	GWAS	PR Indices, AF Type	PR Interval , AF	EUR + AFR + HIS + BR, EUR, EUR + EAS
ZNF25	Yamada et al 2017	ExWAS	AF Type	AF	EAS
ZNF281	Ntalla et al 2020	GWAS	PR Indices	PR Interval	EUR + AFR + HIS + BR, EUR

Target	Paper	Sequencing	Main Trait	Trait	Ancestry
ZNF292	Roselli et al 2018 + Nielsen et al 2018a, Miyazawa et al 2023	GWAS	AF Type	AF and/or AFl, AF	EUR + AFR + EAS + HIS + BR, EUR, EUR + EAS
ZNF358	Ntalla et al 2020	GWAS	PR Indices	PR Interval	EUR + AFR + HIS + BR
ZNF438	Ntalla et al 2020	GWAS	PR Indices	PR Interval	EUR + AFR + HIS + BR
ZNF462	Roselli et al 2018	GWAS	AF Type	AF and/or AFl	EUR + AFR + EAS + HIS + BR
ZNF572	Ntalla et al 2020	GWAS	PR Indices	PR Interval	EUR
ZNF608	Ntalla et al 2020	GWAS	PR Indices	PR Interval	EUR + AFR + HIS + BR
ZNF664	Roselli et al 2018	GWAS	AF Type	AF and/or AFl	EUR, EUR + AFR + EAS + HIS + BR
ZNF689	Miyazawa et al 2023	GWAS	AF Type	AF	EAS
ZNF862	Yamada et al 2017	ExWAS	AF Type	AF	EAS
ZNF879	Yamada et al 2017	ExWAS	AF Type	AF	EAS
ZPBP2	Roselli et al 2018 + Nielsen et al 2018a, Nielsen et al 2018a, Miyazawa et al 2023	GWAS	AF Type	AF and/or AFl, AF	EUR + AFR + EAS + HIS + BR, EUR, EUR + EAS
ZRANB1	Yamada et al 2017	ExWAS	AF Type	AF	EAS
ZSCAN20	Yamada et al 2017	ExWAS	AF Type	AF	EAS

Legend: GWAS – Genome-Wide Association Study; ExWAS – Exome-Wide Association Study; CNV – Copy Number Variant; WGS – Whole Genome Sequencing; EUR – European; AFR – African; EAS – East Asian; SAS – South Asian; HIS – Hispanic; BR – Brazilian; MID – Middle Eastern; OTR – Other; AF – Atrial Fibrillation; AFl – Atrial Flutter; LAminVol – Left Atrium Minimum Volume; LAmaxVol – Left Atrium maximum Volume; LAPEF – Left Atrial Passive Emptying Fraction; LAAEF – Left Atrial Active Emptying Fraction; LATEF – Left Atrial Total Empyting Fraction; PTF – P wave Terminal Force; LA - Left Atrium; RBINT - Rank Based Inverse Normal Transformed; ; Nielsen et al 2018a - Reference (49); Nielsen et al 2018b - Reference (52); Christopherson et al 2017a - Reference (58); Christopherson et al 2017b - Reference (56); *indicates study where variants did not reach contemporary genome wide significance.

Supplementary Table 6 - All targets from analuysis looking at the effect of discovery variants on expression levels of genes

Symbol	TWAS	Paper (TWAS)	eQTL	Paper (eQTL)
AAK1			LA	Hsu et al 2019
ABHD17C	RA, LV	Miyazawa et al 2023		
AC002066.1			LA	Hsu et al 2019
AC006159.3			LA	Hsu et al 2019
AC006159.4			LA	Hsu et al 2019
AC006159.5			LA	Hsu et al 2019
AC007318.5			LA	Hsu et al 2019
AC007386.3			LA	Hsu et al 2019
AC007386.4			LA	Hsu et al 2019
AC007620.3			LV	Roselli et al 2018
AC007880.1			LA	Hsu et al 2019
AC009948.5			LA	Hsu et al 2019
AC009948.7			LA	Hsu et al 2019
AC011747.4	RA	Ntalla et al 2020		
AC016747.3			RA	Roselli et al 2018
AC019206.1			LA	Hsu et al 2019
AC022400.1			LA	Hsu et al 2019
AC022400.2			LA	Hsu et al 2019
AC073130.3			LA	Hsu et al 2019
AC092431.2			LA	Hsu et al 2019
AC092431.3			LA	Hsu et al 2019
AC140912.1			LA	Hsu et al 2019
ACP6	LV	Ntalla et al 2020		
ACTR2			LA	Hsu et al 2019
ACVR2A	RA, LV	Miyazawa et al 2023, Roselli et al 2018		
ADAM15	LV	Miyazawa et al 2023	LA	Christopherson et al 2017a, Hsu et al 2019
ADAR			LA	Hsu et al 2019
ADCY5			RA	Ntalla et al 2020
AF001548.5			RA	Ntalla et al 2020
AGAP5	RA, LV	Miyazawa et al 2023, Ntalla et al 2020	LA	Hsu et al 2019
AKAP6	RA	Miyazawa et al 2023		
AL356475.1			LA	Hsu et al 2019

Symbol	TWAS	Paper (TWAS)	eQTL	Paper (eQTL)
AL590822.1	RA	Ntalla et al 2020	RA	Ntalla et al 2020
ALG1L13P	RA	Ahlberg et al 2020		
ALPK3	RA	Ntalla et al 2020	RA	Ntalla et al 2020
AMT			RA	Ntalla et al 2020
ANKRD1			LV	Ahlberg et al 2020
ANTKMT	RA	Miyazawa et al 2023		
ANXA4			LA, LV	Hsu et al 2019, Christopherson et al 2017a
ANXA7			LA	Hsu et al 2019
AOPEP	RA	Ahlberg et al 2020, Miyazawa et al 2023	LA, RA, LV	Hsu et al 2019, Ahlberg et al 2020
ARHGAP32			LA	Hsu et al 2019
ARL17A			LV	Roselli et al 2018
ARL3	LV	Roselli et al 2018		
ARNT2	RA, LV	Miyazawa et al 2023, Roselli et al 2018	RA	Roselli et al 2018
ASAH1	LV	Miyazawa et al 2023	LA, LV	Christopherson et al 2017a, Hsu et al 2019, Nielsen et al 2018a, Roselli et al 2018
ASPRV1			LA	Hsu et al 2019
ATP5F1D	RA	Ntalla et al 2020		
ATP5MK			LA	Hsu et al 2019
BAZ2A	RA, LV	Miyazawa et al 2023		
BCAT1			LA	Van Setten et al 2018
BEND7	RA	Ntalla et al 2020		
BEST3	LV	Roselli et al 2018		
BHLHE41	LV	Miyazawa et al 2023		
BLOC1S1	LV	Miyazawa et al 2023		
BMP8A			RA, LV	Lin et al 2018
BMPR1A	RA, LV	Ntalla et al 2020		
BMS1P4			LA	Hsu et al 2019
BNIP1	RA, LV	Miyazawa et al 2023	LA	Van Setten et al 2018
BRD8			LA	Hsu et al 2019
BRICD5	LV	Miyazawa et al 2023		
BTRC	RA	Miyazawa et al 2023		
C10orf55			LA	Hsu et al 2019
C10orf71	LV	Ntalla et al 2020		

Symbol	TWAS	Paper (TWAS)	eQTL	Paper (eQTL)
C2orf69	RA	Miyazawa et al 2023		
C4A	RA, LV	Miyazawa et al 2023		
C5orf47	RA	Miyazawa et al 2023		
CAB39L	LV	Ntalla et al 2020	RA, LV	Ntalla et al 2020
CALHM1			LA	Hsu et al 2019
CALHM2	RA, LV	Ntalla et al 2020	LA	Hsu et al 2019
CALHM3			LA	Hsu et al 2019
CAMK2D	LV	Ntalla et al 2020		
CAMK2G			LA	Hsu et al 2019
CAND2			LA	Hsu et al 2019
CASQ2	RA, LV	Ahlberg et al 2020, Miyazawa et al 2023, Roselli et al 2018	RA, LV	Ahlberg et al 2020, Nielsen et al 2018a
CAV1	RA	Miyazawa et al 2023, Roselli et al 2018	LA	Christopherson et al 2017b, Christopherson et al 2017a, Van Setten et al 2018, Hsu et al 2019
CAV2	RA	Miyazawa et al 2023	LA	Christopherson et al 2017b, Christopherson et al 2017a, Hsu et al 2019
CAVIN4	RA	Miyazawa et al 2023		
CBX6	RA, LV	Miyazawa et al 2023		
CBX8	RA	Ntalla et al 2020	LV	Ntalla et al 2020
CCDC116	RA, LV	Miyazawa et al 2023		
CCDC134	RA, LV	Miyazawa et al 2023		
CCDC92	RA, LV	Miyazawa et al 2023, Roselli et al 2018	RA, LV	Roselli et al 2018
CCT2	RA	Miyazawa et al 2023		
CDC23			LA	Hsu et al 2019
CDC25C			LA	Hsu et al 2019
CDC7			LV	Ntalla et al 2020
CDH13	RA, LV	Ntalla et al 2020	RA, LV	Ntalla et al 2020
CDKN1A	LV	Miyazawa et al 2023		
CEP68	RA, LV	Roselli et al 2018, Miyazawa et al 2023	LA, RA, LV	Christopherson et al 2017a, Sinner et al 2014, Hsu et al 2019, Low et al 2017, Roselli et al 2018
CEP85L			LA	Hsu et al 2019
CEPT1	RA	Roselli et al 2018		
CFAP410	RA	Miyazawa et al 2023		
CFAP68	RA, LV	Ntalla et al 2020	RA, LV	Ntalla et al 2020
CFAP70	LV	Ntalla et al 2020		

Symbol	TWAS	Paper (TWAS)	eQTL	Paper (eQTL)
CFDP1	RA	Ntalla et al 2020		
CFL2	RA, LV	Miyazawa et al 2023		
CGA	LV	Miyazawa et al 2023		
CHCHD1			LA	Hsu et al 2019
CHD1L			LV	Ntalla et al 2020
CHDH			LV	Ntalla et al 2020
CHRM2	LV	Ntalla et al 2020	LV	Ntalla et al 2020
CIAO3	LV	Miyazawa et al 2023		
CKS1B			LA	Hsu et al 2019
CMTM5	RA	Ntalla et al 2020, Roselli et al 2018		
COPS4	RA	Miyazawa et al 2023		
CPEB4	LV	Miyazawa et al 2023		
CSPG4P11	RA, LV	Ntalla et al 2020	LA, RA	Baldassari et al 2020, Christopherson et al 2017b, Ntalla et al 2020
CTC-313D10.1			LA	Hsu et al 2019
CTD-2547L16.1			LA	Hsu et al 2019
CTD-2547L16.3			LA	Hsu et al 2019
CTD-2555O16.1			LA	Hsu et al 2019
CTD-2555O16.2			LA	Hsu et al 2019
CTXND1	RA, LV	Miyazawa et al 2023		
DAG1	LV	Ntalla et al 2020	LV	Ntalla et al 2020
DALRD3			RA	Ntalla et al 2020
DCST1			LA	Hsu et al 2019
DCST1-AS1			LA	Hsu et al 2019
DCST2	RA, LV	Miyazawa et al 2023, Roselli et al 2018	LA	Hsu et al 2019
DDX42	RA	Ntalla et al 2020	RA, LV	Ntalla et al 2020
DEK	RA, LV	Miyazawa et al 2023, Ntalla et al 2020, Roselli et al 2018	RA, LV	Ntalla et al 2020, Nielsen et al 2018a, Roselli et al 2018
DESI1	RA, LV	Miyazawa et al 2023		
DMWD	LV	Ahlberg et al 2020	LV	Ahlberg et al 2020
DNAH10OS	RA, LV	Roselli et al 2018	RA, LV	Roselli et al 2018
DNAH11	RA	Ntalla et al 2020	RA	Ntalla et al 2020
DNAJC12	LV	Miyazawa et al 2023		
DNM1P51	RA	Ntalla et al 2020		

Symbol	TWAS	Paper (TWAS)	eQTL	Paper (eQTL)
DPCD	RA	Miyazawa et al 2023		
DPF3	RA, LV	Miyazawa et al 2023		
DRICH1			RA	Ntalla et al 2020
DUSP13	LV	Miyazawa et al 2023		
DUSP29			LV	Ntalla et al 2020
DUSP8P5			LA, RA	Hsu et al 2019, Ntalla et al 2020
EDN2	RA, LV	Ntalla et al 2020	RA, LV	Ntalla et al 2020
EFNA1	LV	Ntalla et al 2020	LV	Ntalla et al 2020
EFNA3			LA	Hsu et al 2019
EFNA4			LA	Hsu et al 2019
EMB	RA, LV	Ntalla et al 2020		
ENPEP			LA	Hsu et al 2019
ERBB2	RA, LV	Miyazawa et al 2023		
ERCC6L2	LV	Roselli et al 2018		
ESR2	RA	Miyazawa et al 2023	LA	Hsu et al 2019
FAAP20	RA, LV	Ntalla et al 2020		
FABP2			RA, LV	Ntalla et al 2020
FADS1	LV	Ntalla et al 2020	LV	Ntalla et al 2020
FAM13B	RA, LV	Miyazawa et al 2023, Roselli et al 2018	LA, RA	Hsu et al 2019, Christopherson et al 2017a
FAM43A	RA	Miyazawa et al 2023, Roselli et al 2018		
FAM53C			LA	Hsu et al 2019
FANCC			LA	Hsu et al 2019
FAT1	RA, LV	Ntalla et al 2020	LA, RA, LV	Van Setten et al 2018, Ntalla et al 2020
FBN2	LV	Miyazawa et al 2023, Roselli et al 2018	RA	Ntalla et al 2020
FBXL20	RA, LV	Miyazawa et al 2023		
FBXL22	RA	Miyazawa et al 2023		
FBXO32	RA, LV	Roselli et al 2018, Miyazawa et al 2023		
FDFT1	LV	Ahlberg et al 2020		
FERMT2			LA	Van Setten et al 2018
FGL1			LA	Hsu et al 2019
FKBP1AP2			LA	Hsu et al 2019
FKBP7	RA, LV	Ntalla et al 2020, Miyazawa et al 2023	LA, RA	Hsu et al 2019, Ntalla et al 2020

Symbol	TWAS	Paper (TWAS)	eQTL	Paper (eQTL)
FLAD1			LA	Hsu et al 2019
FLI1			LA	Hsu et al 2019
FOXK1	RA, LV	Miyazawa et al 2023		
FTCDNL1	LV	Miyazawa et al 2023		
FTSJ3			RA, LV	Ntalla et al 2020
FUT11	RA, LV	Miyazawa et al 2023, Roselli et al 2018, Ntalla et al 2020	LA, LV	Hsu et al 2019, Ntalla et al 2020
GAREM1			RA	Ntalla et al 2020
GBAP1	RA, LV	Ntalla et al 2020	LA, RA, LV	Van Setten et al 2018, Lin et al 2018, Ntalla et al 2020
GBF1	RA	Ntalla et al 2020		
GFM1			RA	Ntalla et al 2020
GFRA3			LA	Hsu et al 2019
GJA1	LV	Miyazawa et al 2023	LA	Sinner et al 2014, Hsu et al 2019
GLUD1P3			LA	Hsu et al 2019
GMCL1	RA, LV	Miyazawa et al 2023, Roselli et al 2018	LA, LV	Hsu et al 2019, Christopherson et al 2017a
GMPPB			RA	Ntalla et al 2020
GNB4	RA, LV	Miyazawa et al 2023, Roselli et al 2018	RA, LV	Roselli et al 2018
GOLGA2P7	RA, LV	Ntalla et al 2020	LA, RA, LV	Baldassari et al 2020, Christopherson et al 2017b, Ntalla et al 2020
GOLGA6L4			RA	Ntalla et al 2020
GORAB			LA	Hsu et al 2019
GORASP1	RA	Ntalla et al 2020		
GPR155	LV	Miyazawa et al 2023		
GPR85	RA	Miyazawa et al 2023, Roselli et al 2018		
GSDMB	RA	Miyazawa et al 2023		
GYPC	RA	Roselli et al 2018	RA, LV	Nielsen et al 2018a
HAGHL	RA, LV	Miyazawa et al 2023, Ahlberg et al 2020		
HAND2-AS1			LV	Roselli et al 2018
HAUS4P1			LA	Hsu et al 2019
HCCAT5			LA	Hsu et al 2019
HCN1	RA	Ntalla et al 2020	RA	Ntalla et al 2020
HCN4			LA	Hsu et al 2019
HERC4	RA, LV	Miyazawa et al 2023		

Symbol	TWAS	Paper (TWAS)	eQTL	Paper (eQTL)
HIP1	RA	Roselli et al 2018		
HLTF	LV	Miyazawa et al 2023		
HMGA1P5	RA	Ntalla et al 2020		
HPS6	RA	Miyazawa et al 2023		
HSF2			LA	Hsu et al 2019
IBA57			LA	Van Setten et al 2018
IFRD2	LV	Ntalla et al 2020		
IFT88			LV	Ntalla et al 2020
IGF1R	LV	Miyazawa et al 2023, Roselli et al 2018	LV	Roselli et al 2018
IHO1	LV	Ntalla et al 2020	RA, LV	Ntalla et al 2020
IKZF3	LV	Miyazawa et al 2023		
IL17D	LV	Ntalla et al 2020	LV	Ntalla et al 2020
IL25	RA	Ntalla et al 2020		
IL6R	RA	Miyazawa et al 2023		
INA			LA	Hsu et al 2019
IQSEC1			LA	Hsu et al 2019
ITGB1	RA	Miyazawa et al 2023		
JAM2	RA, LV	Miyazawa et al 2023		
KANSL1-AS1			LV	Roselli et al 2018
KB-1507C5.2			RA, LV	Ntalla et al 2020
KCND3	RA, LV	Ntalla et al 2020		
KCNJ1			LA	Hsu et al 2019
KCNJ5	RA, LV	Miyazawa et al 2023, Roselli et al 2018	LA, RA, LV	Christopherson et al 2017a, Hsu et al 2019, Roselli et al 2018
KCNJ5-AS1	RA, LV	Miyazawa et al 2023, Roselli et al 2018	LA, LV	Hsu et al 2019, Christopherson et al 2017a, Roselli et al 2018
KCNN2			LA	Christopherson et al 2017a, Hsu et al 2019
KCNN3			LA	Hsu et al 2019
KDM1B	RA, LV	Miyazawa et al 2023, Ntalla et al 2020, Roselli et al 2018	RA, LV	Nielsen et al 2018a, Ntalla et al 2020, Roselli et al 2018
KDM3B	RA	Miyazawa et al 2023, Roselli et al 2018		
KIF20A			LA	Hsu et al 2019
KIFAP3			LA	Christopherson et al 2017a, Hsu et al 2019
KLF12	RA	Miyazawa et al 2023		
KPNA3	RA	Ntalla et al 2020		

Symbol	TWAS	Paper (TWAS)	eQTL	Paper (eQTL)
KRT18P17			LA	Hsu et al 2019
L3MBTL2			RA	Ntalla et al 2020
LENEP			LA	Hsu et al 2019
LIN54	RA	Miyazawa et al 2023		
LINC00467			LA	Sinner et al 2014
LINC00881			LA	Van Setten et al 2018
LINC00933			RA	Ntalla et al 2020
LINC00964	LV	Ntalla et al 2020	LV	Ntalla et al 2020, Roselli et al 2018
LINC01142			LA	Hsu et al 2019
LINC01411			RA	Ntalla et al 2020
LINC01629	RA, LV	Miyazawa et al 2023		
LINC02210			LV	Roselli et al 2018
LINC02245			LA	Hsu et al 2019
LINC-PINT			LA	Van Setten et al 2018
LRCH1	LV	Ntalla et al 2020	LA, LV	Van Setten et al 2018, Ntalla et al 2020
LRRC10	RA, LV	Miyazawa et al 2023		
LRRC75B	RA	Ntalla et al 2020		
LXN			LV	Ntalla et al 2020
LYPLA1P2			LA	Hsu et al 2019
MAIP1	RA, LV	Miyazawa et al 2023		
MALAT1	RA	Ntalla et al 2020		
MAP3K3			LV	Ntalla et al 2020
MAPT	RA, LV	Miyazawa et al 2023, Roselli et al 2018	RA, LV	Roselli et al 2018
MBD5	RA	Miyazawa et al 2023		
MED1	RA, LV	Miyazawa et al 2023		
MEI1			RA, LV	Ntalla et al 2020
MEIOSIN			RA	Ahlberg et al 2020
MEIS1			LA	Van Setten et al 2018
MET			LA	Hsu et al 2019
MICU2	LV	Miyazawa et al 2023		
MIR1307			LA	Christopherson et al 2017a, Hsu et al 2019
MIR193BHG	LV	Miyazawa et al 2023		

Symbol	TWAS	Paper (TWAS)	eQTL	Paper (eQTL)
MIR22HG	RA	Ahlberg et al 2020		
MIR27B			LA	Hsu et al 2019
MIR490			LV	Ntalla et al 2020
MIX23	RA, LV	Miyazawa et al 2023		
MKRN2	RA, LV	Roselli et al 2018	LA, LV	Hsu et al 2019, Roselli et al 2018
MKRN2OS	LV	Roselli et al 2018	LA	Hsu et al 2019
MLF1	RA, LV	Ntalla et al 2020	RA, LV	Ntalla et al 2020
MMAB			LV	Ntalla et al 2020
MMP11	RA, LV	Miyazawa et al 2023, Ntalla et al 2020		
MPPED2	LV	Roselli et al 2018	LV	Ntalla et al 2020
MRPL37	RA	Ntalla et al 2020	RA, LV	Christopherson et al 2017b, Ntalla et al 2020
MRPS15P1			LA	Hsu et al 2019
MSH5	RA	Miyazawa et al 2023		
MSS51			LA	Hsu et al 2019
MSTO2P	LV	Ntalla et al 2020		
MTHFD1	RA, LV	Miyazawa et al 2023	LA	Hsu et al 2019
MTSS1	RA, LV	Miyazawa et al 2023, Ntalla et al 2020, Roselli et al 2018	RA, LV	Ntalla et al 2020, Roselli et al 2018
MXD1	LV	Miyazawa et al 2023	LA	Hsu et al 2019
MYBPHL	RA	Ntalla et al 2020		
MYO15A	LV	Ntalla et al 2020		
MYOCD			LA	Van Setten et al 2018
MYOT			LA	Hsu et al 2019
MYOZ1	RA, LV	Miyazawa et al 2023, Ntalla et al 2020, Roselli et al 2018	LA, RA	Christopherson et al 2017a, Hsu et al 2019, Lin et al 2018, Nielsen et al 2018a, Ntalla et al 2020, Roselli et al 2018
MYOZ2			LV	Ntalla et al 2020
MYPN	LV	Miyazawa et al 2023		
NACA	RA, LV	Miyazawa et al 2023		
NAT1			LA	Hsu et al 2019
NCKIPSD			RA, LV	Ntalla et al 2020
NDRG4			LV	Ntalla et al 2020
NDST2	RA	Ntalla et al 2020	LA	Hsu et al 2019
NDUFAF3			RA	Ntalla et al 2020
NDUFB10	RA, LV	Miyazawa et al 2023		

Symbol	TWAS	Paper (TWAS)	eQTL	Paper (eQTL)
NEO1			LA	Hsu et al 2019
NEURL1	RA, LV	Miyazawa et al 2023, Ntalla et al 2020	LA	Hsu et al 2019
NICN1			RA	Ntalla et al 2020
NKX2-5	RA, LV	Miyazawa et al 2023	LA	Sinner et al 2014
NMB			LA	Christopherson et al 2017b
NME5	RA	Roselli et al 2018	LA	Hsu et al 2019
NPIPA1	LV	Ntalla et al 2020		
NPIPA5	RA	Ntalla et al 2020	LV	Ntalla et al 2020
NPM1P43			LA	Hsu et al 2019
NPR3			RA	Ntalla et al 2020
NPTN			LA	Hsu et al 2019
NPTXR	LV	Miyazawa et al 2023		
NR3C1	LV	Miyazawa et al 2023		
NRBF2			LV	Roselli et al 2018
NSFP1			LV	Roselli et al 2018
NTMT2	LV	Miyazawa et al 2023, Roselli et al 2018	LA	Hsu et al 2019
NUCKS1	RA, LV	Roselli et al 2018	RA, LV	Roselli et al 2018
NUDT13	RA	Ntalla et al 2020		
ORC3	RA, LV	Miyazawa et al 2023		
ORMDL3	RA	Miyazawa et al 2023		
OSBPL6			LA	Hsu et al 2019
OSTF1P1			LA	Hsu et al 2019
OXCT2P1			RA, LV	Lin et al 2018, Ntalla et al 2020
PAPLN	RA	Miyazawa et al 2023		
PBXIP1	RA	Miyazawa et al 2023	LA	Hsu et al 2019
PCBP1-AS1			LA	Hsu et al 2019
PCCB	RA	Miyazawa et al 2023		
PCGF5	RA	Ahlberg et al 2020	RA, LV	Ahlberg et al 2020
PCGF6			LA	Hsu et al 2019
PCM1	LV	Miyazawa et al 2023, Roselli et al 2018	LA, LV	Christopherson et al 2017a, Hsu et al 2019, Roselli et al 2018
PCYOX1	LV	Roselli et al 2018	LV	Christopherson et al 2017a
PDCD11			LA	Hsu et al 2019

Symbol	TWAS	Paper (TWAS)	eQTL	Paper (eQTL)
PDZRN3	RA, LV	Ntalla et al 2020	LA, RA, LV	Van Setten et al 2018, Ntalla et al 2020
PDZRN3-AS1			LA, RA, LV	Van Setten et al 2018, Ntalla et al 2020
PERM1	RA, LV	Miyazawa et al 2023		
PEX26	RA	Miyazawa et al 2023		
PFDN1	RA, LV	Miyazawa et al 2023		
PGAP3	RA, LV	Miyazawa et al 2023, Roselli et al 2018		
PGP	RA, LV	Miyazawa et al 2023		
PHACTR1	RA	Ntalla et al 2020		
PHLDB2	RA, LV	Miyazawa et al 2023, Ntalla et al 2020		
PIK3C2B	RA, LV	Miyazawa et al 2023		
PITX2			LA	Hsu et al 2019
PITX3	RA, LV	Miyazawa et al 2023		
PJVK			LA	Hsu et al 2019
PKD2L2			LA	Hsu et al 2019
PKP2	RA	Miyazawa et al 2023		
PLAU	RA	Miyazawa et al 2023, Roselli et al 2018	LA, RA	Hsu et al 2019, Christopherson et al 2017a
PLCD1	LV	Ntalla et al 2020		
PLEC			RA, LV	Lin et al 2018, Ntalla et al 2020
PLEKHA3			LA	Hsu et al 2019
PLN	RA, LV	Miyazawa et al 2023		
PLPP7	RA, LV	Ntalla et al 2020	LV	Ntalla et al 2020
PM20D1			RA, LV	Roselli et al 2018
PMS2P2			LV	Roselli et al 2018
PMVK	RA	Miyazawa et al 2023	LA	Hsu et al 2019
PNMT	RA, LV	Miyazawa et al 2023		
PPP1R1B	RA	Miyazawa et al 2023		
PPP3CB			LA	Hsu et al 2019
PRKCA			RA, LV	Ntalla et al 2020
PRKRA	RA	Miyazawa et al 2023	LA	Hsu et al 2019
PROZ	RA, LV	Miyazawa et al 2023		
PRRX1	RA, LV	Miyazawa et al 2023, Roselli et al 2018	LA, LV	Christopherson et al 2017a, Hsu et al 2019

Symbol	TWAS	Paper (TWAS)	eQTL	Paper (eQTL)
PSMB7	RA, LV	Miyazawa et al 2023		
PSMD3	LV	Miyazawa et al 2023		
PXN	LV	Roselli et al 2018		
PYGO2			LA	Hsu et al 2019
QRICH1	LV	Ntalla et al 2020	RA, LV	Ntalla et al 2020
RAB1A			LA	Hsu et al 2019
RAB29			RA, LV	Roselli et al 2018
RAF1			LA	Hsu et al 2019
RBM6			RA	Ntalla et al 2020
RCAN2	RA	Ntalla et al 2020	RA	Ntalla et al 2020
RDH5	RA, LV	Miyazawa et al 2023		
REC114			LA	Hsu et al 2019
REEP2	RA, LV	Miyazawa et al 2023, Roselli et al 2018	LV	Christopherson et al 2017a
REEP3	LV	Miyazawa et al 2023		
RIOK1	LV	Miyazawa et al 2023		
RN7SKP89			LA	Hsu et al 2019
RN7SL211P			LA	Hsu et al 2019
RN7SL356P			RA, LV	Roselli et al 2018
RN7SL682P			LA	Hsu et al 2019
RNF11	RA, LV	Miyazawa et al 2023		
RNF144B	RA	Miyazawa et al 2023		
RNU11-3P			LA	Hsu et al 2019
RNU6-548P			LA	Hsu et al 2019
RP11-1070N10.3	RA	Ntalla et al 2020	RA	Ntalla et al 2020
RP11-10A14.4	LV	Miyazawa et al 2023	RA	Ahlberg et al 2020
RP11-137L10.5			LA	Hsu et al 2019
RP11-137L10.6			LA, LV	Hsu et al 2019, Christopherson et al 2017a
RP11-139B1.1			LA	Hsu et al 2019
RP11-145M9.4			RA, LV	Roselli et al 2018
RP11-156K13.1			LA	Hsu et al 2019
RP11-156K13.2			LA	Hsu et al 2019
RP11-156K13.3			LA	Hsu et al 2019

Symbol	TWAS	Paper (TWAS)	eQTL	Paper (eQTL)
RP11-166J22.1			LA	Hsu et al 2019
RP11-171I2.1			LA	Hsu et al 2019
RP11-182J1			LA	Baldassari et al 2020
RP11-182J1.14			LV	Ntalla et al 2020
RP11-210M15.2	RA	Miyazawa et al 2023, Roselli et al 2018	RA	Roselli et al 2018
RP11-225H22.4			LA	Hsu et al 2019
RP11-225H22.5			LA	Hsu et al 2019
RP11-236B18.2	RA, LV	Ahlberg et al 2020	RA, LV	Ahlberg et al 2020
RP11-259G18.2			LV	Roselli et al 2018
RP11-259G18.3			LV	Roselli et al 2018
RP11-272D12.1			LA	Hsu et al 2019
RP11-272D12.2			LA	Hsu et al 2019
RP11-297H3.3			LA	Hsu et al 2019
RP11-29H23.5	LV	Ntalla et al 2020		
RP11-2E17.1			RA	Roselli et al 2018
RP11-325L7.1			LA	Hsu et al 2019
RP11-325L7.2			LA	Hsu et al 2019
RP11-332H17.1			LA	Hsu et al 2019
RP11-337C18.8			RA, LV	Ntalla et al 2020
RP11-344N10.5	LV	Ntalla et al 2020		
RP11-345K20.2			LA	Hsu et al 2019
RP11-379F4.7	LV	Ntalla et al 2020		
RP11-379H18.1			RA	Ntalla et al 2020
RP11-380D23.2			LA	Hsu et al 2019
RP11-380L11.4			LV	Roselli et al 2018
RP11-381K20.2			LA	Hsu et al 2019
RP11-397E7.4	LV	Ntalla et al 2020		
RP11-399K21.11	RA	Ntalla et al 2020	RA	Ntalla et al 2020
RP11-3B7.1	LV	Ntalla et al 2020	RA, LV	Ntalla et al 2020
RP11-464F9.1			LA	Hsu et al 2019
RP11-464F9.20			LA	Hsu et al 2019
RP11-464F9.22	RA, LV	Miyazawa et al 2023		

Symbol	TWAS	Paper (TWAS)	eQTL	Paper (eQTL)
RP11-479G22.8			RA, LV	Ntalla et al 2020
RP11-492A10.1			LA	Hsu et al 2019
RP11-49O14.2			LA	Hsu et al 2019
RP11-49O14.3			LA	Hsu et al 2019
RP11-532N4.2			RA	Ntalla et al 2020
RP11-541N10.3			LA	Hsu et al 2019
RP11-544I20.2			LA	Hsu et al 2019
RP11-54O15.3			LA	Hsu et al 2019
RP11-574K11.5			LA	Hsu et al 2019
RP11-574K11.8			LA	Hsu et al 2019
RP11-576I22.2			LA	Hsu et al 2019
RP11-57K17.1			LA	Hsu et al 2019
RP11-632C17__A.1			LA	Hsu et al 2019
RP11-65L3.1			LA	Hsu et al 2019
RP11-69E11.4			LV	Ntalla et al 2020
RP11-69E11.4			LV	Lin et al 2018
RP11-707O23.5			LV	Roselli et al 2018
RP11-724N1.1	RA	Ntalla et al 2020		
RP11-72I8.1			LV	Ntalla et al 2020
RP11-740D6.3			LA	Hsu et al 2019
RP11-744N12.3			LA	Hsu et al 2019
RP11-767C1.1			LA	Hsu et al 2019
RP11-777N19.1			LA	Hsu et al 2019
RP11-77O7.1			LA	Hsu et al 2019
RP11-795J1.1			LV	Ntalla et al 2020
RP11-7F17.1			RA, LV	Roselli et al 2018
RP11-7F17.7			RA, LV	Roselli et al 2018
RP11-806O11.1			LA, LV	Hsu et al 2019, Christopherson et al 2017a
RP11-80I15.1			LA	Hsu et al 2019
RP11-80I15.4			LA	Hsu et al 2019
RP11-88L24.4			LA	Hsu et al 2019
RP11-95P2.1			RA	Ntalla et al 2020

Symbol	TWAS	Paper (TWAS)	eQTL	Paper (eQTL)
RP1-1J6.2			LV	Ntalla et al 2020
RP1-79C4.1			LA	Hsu et al 2019
RP1-79C4.4	RA, LV	Miyazawa et al 2023	LV	Christopherson et al 2017a, Roselli et al 2018
RP4-764O22.2	LV	Ntalla et al 2020		
RP5-991G20.1			LA	Hsu et al 2019
RP5-991G20.2			LA	Hsu et al 2019
RP5-991G20.4			LA	Hsu et al 2019
RPL32			LA	Hsu et al 2019
RPL3L	RA, LV	Miyazawa et al 2023, Roselli et al 2018	RA	Roselli et al 2018
RPSA	LV	Ntalla et al 2020	RA	Lin et al 2018
SCMH1	LV	Miyazawa et al 2023		
SCN10A	RA, LV	Miyazawa et al 2023, Ntalla et al 2020	LA	Van Setten et al 2018
SCN5A	RA	Ntalla et al 2020	LA	Van Setten et al 2018
SEC23IP	RA	Miyazawa et al 2023		
SEC24C	LV	Miyazawa et al 2023	LA	Hsu et al 2019
SEC31A	RA	Miyazawa et al 2023		
SEMA3C	LV	Miyazawa et al 2023		
SENCR			LA	Hsu et al 2019
SENP2			LA	Van Setten et al 2018
SETD6			LV	Ntalla et al 2020
SF3B1	RA, LV	Miyazawa et al 2023		
SH3PXD2A	RA, LV	Miyazawa et al 2023, Ntalla et al 2020	LA, LV	Van Setten et al 2018, Hsu et al 2019, Ntalla et al 2020
SHC1			LA	Hsu et al 2019
SIGLEC30P			LA	Hsu et al 2019
SIRT1	RA, LV	Miyazawa et al 2023		
SKI			LA	Van Setten et al 2018
SLC16A12	LV	Miyazawa et al 2023		
SLC1A4			LA	Hsu et al 2019
SLC25A26	RA, LV	Ntalla et al 2020, Miyazawa et al 2023	RA, LV	Ntalla et al 2020
SLC25A27			RA, LV	Ntalla et al 2020
SLC27A6	RA, LV	Miyazawa et al 2023, Roselli et al 2018		
SLC2A11	LV	Ntalla et al 2020		

Symbol	TWAS	Paper (TWAS)	eQTL	Paper (eQTL)
SLC2A9	RA, LV	Miyazawa et al 2023		
SLC35A1	RA, LV	Miyazawa et al 2023		
SLC35E1P1			RA	Ntalla et al 2020
SLC35F1			LA	Hsu et al 2019
SLC6A6	RA	Ntalla et al 2020	RA	Ntalla et al 2020
SLK	RA	Ntalla et al 2020	LA	Hsu et al 2019
SMARCB1	RA, LV	Ntalla et al 2020		
SNAPIN	RA	Miyazawa et al 2023		
snoU13			LA	Hsu et al 2019
snoZ185			LA	Hsu et al 2019
SNRNP27			LA, RA, LV	Hsu et al 2019, Roselli et al 2018
SNX1	RA	Ntalla et al 2020		
SPATA20	RA, LV	Ntalla et al 2020	LV	Ntalla et al 2020
SPATS2L	RA, LV	Miyazawa et al 2023		
SPTBN1	RA	Ntalla et al 2020		
SSBP3	RA, LV	Ntalla et al 2020	LA, RA, LV	Christopherson et al 2017b, Ntalla et al 2020
SSPN	RA, LV	Miyazawa et al 2023	RA	Roselli et al 2018
SSXP10	RA	Ntalla et al 2020	RA, LV	Lin et al 2018, Ntalla et al 2020
STIM1	RA, LV	Miyazawa et al 2023		
STN1	RA, LV	Miyazawa et al 2023	LA	Hsu et al 2019
STRN	LV	Ntalla et al 2020	RA, LV	Ntalla et al 2020
SYMPK			RA, LV	Ahlberg et al 2020
SYNE2	RA, LV	Ntalla et al 2020, Miyazawa et al 2023, Roselli et al 2018	LA, RA, LV	Christopherson et al 2017a, Hsu et al 2019, Ntalla et al 2020, Nielsen et al 2018a, Roselli et al 2018
SYNPO2L	RA, LV	Miyazawa et al 2023, Ntalla et al 2020	LA, RA	Christopherson et al 2017a, Hsu et al 2019, Ntalla et al 2020
SYPL2	RA, LV	Ntalla et al 2020	LA	Van Setten et al 2018
TAF5			LA	Hsu et al 2019
TBC1D32			LA	Hsu et al 2019
TBL3	LV	Miyazawa et al 2023		
TBX5	RA, LV	Miyazawa et al 2023, Roselli et al 2018	LA, LV	Christopherson et al 2017b, Christopherson et al 2017a, Sinner et al 2014, Hsu et al 2019, Lin et al 2018
TBX5-AS1			LA	Hsu et al 2019

Symbol	TWAS	Paper (TWAS)	eQTL	Paper (eQTL)
TCHP	LV	Ahlberg et al 2020, Miyazawa et al 2023		
TCTN3	RA, LV	Ntalla et al 2020	LA, RA, LV	Van Setten et al 2018, Ntalla et al 2020
TDRD6			RA, LV	Ntalla et al 2020
TES	LV	Miyazawa et al 2023, Roselli et al 2018		
TESC			LV	Ntalla et al 2020
TEX21P			LA	Hsu et al 2019
TFDP1	RA	Miyazawa et al 2023		
TFEC	LV	Ntalla et al 2020		
THRB	RA, LV	Ntalla et al 2020, Roselli et al 2018, Miyazawa et al 2023	RA, LV	Ntalla et al 2020
TMA7			LV	Ntalla et al 2020
TMEM116			LA	Sinner et al 2014
TMEM182	RA, LV	Ntalla et al 2020	LA, RA, LV	Van Setten et al 2018, Ntalla et al 2020
TMEM40	RA, LV	Miyazawa et al 2023	LA	Hsu et al 2019
TNNT3	LV	Miyazawa et al 2023		
TP53AIP1			LA	Hsu et al 2019
TPMT	RA, LV	Miyazawa et al 2023, Ntalla et al 2020, Roselli et al 2018	RA, LV	Ntalla et al 2020, Roselli et al 2018
TRAK1	RA	Ntalla et al 2020	RA, LV	Ntalla et al 2020
TRDN			LA	Hsu et al 2019
TRDN-AS1			LA	Hsu et al 2019
TRIP4	RA, LV	Ntalla et al 2020	RA, LV	Ntalla et al 2020
TTN			LA	Hsu et al 2019
TTN-AS1			LA	Hsu et al 2019
TYW5	RA, LV	Miyazawa et al 2023		
UBE2D3	RA, LV	Miyazawa et al 2023, Roselli et al 2018	LV	Roselli et al 2018
UBE2J2	RA	Roselli et al 2018		
UBE2L3	RA	Miyazawa et al 2023		
UBE2Q2L	RA	Ntalla et al 2020	RA, LV	Christopherson et al 2017b, Ntalla et al 2020
UBE3B	RA	Ntalla et al 2020		
USMG5			LA	Christopherson et al 2017a
USP28	LV	Miyazawa et al 2023		
USP36	RA	Miyazawa et al 2023		

Symbol	TWAS	Paper (TWAS)	eQTL	Paper (eQTL)
USP54			LA, LV	Hsu et al 2019, Christopherson et al 2017a
VANGL1			RA, LV	Ahlberg et al 2020
VDAC2	LV	Ntalla et al 2020	LV	Ntalla et al 2020
VILL			RA	Ntalla et al 2020
VPREB3	RA	Ntalla et al 2020		
VPS37B	LV	Miyazawa et al 2023		
WDR6			RA, LV	Ntalla et al 2020
WDR73	RA, LV	Ntalla et al 2020	RA, LV	Ntalla et al 2020, Christopherson et al 2017b
WIPF1	RA, LV	Miyazawa et al 2023, Roselli et al 2018	LA, LV	Sinner et al 2014, Nielsen et al 2018a, Roselli et al 2018
WNT3	LV	Miyazawa et al 2023		
WNT8A			LA	Hsu et al 2019
XIRP1	RA	Ntalla et al 2020		
XPO7	LV	Miyazawa et al 2023		
YDJC	RA	Miyazawa et al 2023		
ZBTB25			LA	Hsu et al 2019
ZBTB38	RA	Miyazawa et al 2023		
ZBTB7B	RA, LV	Miyazawa et al 2023	LA	Hsu et al 2019
ZCCHC24	LV	Ntalla et al 2020		
ZFHX3			LA	Hsu et al 2019
ZHX1	LV	Ntalla et al 2020		
ZNF292	LV	Miyazawa et al 2023		
ZNF358			RA	Ntalla et al 2020
ZNF503-AS1	RA, LV	Ntalla et al 2020	RA	Ntalla et al 2020
ZNF589			RA	Ntalla et al 2020
ZNF664	RA, LV	Miyazawa et al 2023, Roselli et al 2018	LV	Roselli et al 2018
ZSWIM8	RA, LV	Miyazawa et al 2023	LA	Hsu et al 2019

Legend: TWAS - Transcriptome Wide Association Study; eQTL - expression Quantitative Trait Loci; LA - Left Atrium; RA -Right Atrium; LV - Left Ventricle. Nielsen et al 2018a - Reference (49); Christopherson et al 2017a - Reference (58); Christopherson et al 2017b - Reference (56);

Supplementary Table 7 - All targets from rare variant analyses

Gene	Paper	Sequencing	Trait	Method
TTN	Weng et al 2020, Wang et al 2023, Choi et al 2018	ExWAS, WGS	P wave duration, AF	SKAT, Burden
DLEC1	Weng et al 2020	ExWAS	P wave duration	SKAT
RPL3L	Weng et al 2020	ExWAS	P wave duration	SKAT
SCN10A	Weng et al 2020	ExWAS	P wave duration	SKAT
MUC5B	Weng et al 2020	ExWAS	P wave duration	Burden
TTC21A	Weng et al 2020	ExWAS	P wave duration INT	SKAT
SH3PXD2A	Christopherson et al 2017a	ExWAS	AF	Burden
MYH6	Lin et al 2018	ExWAS	PR Interval	SKAT
SCN5A	Lin et al 2018	ExWAS	PR Interval	SKAT
GORASP1	Lin et al 2018	ExWAS	PR Interval	SKAT
NEBL	Lin et al 2018	ExWAS	PR Interval	SKAT
TRIML2	Lin et al 2018	ExWAS	PR Interval	SKAT
SLC22A11	Lin et al 2018	ExWAS	PR Interval	SKAT
MTRF1	Lin et al 2018	ExWAS	PR Interval	SKAT
CD36	Lin et al 2018	ExWAS	PR Interval	SKAT
CAPRIN2	Lin et al 2018	ExWAS	PR Interval	SKAT
PIK3R6	Lin et al 2018	ExWAS	PR Interval	SKAT

Legend: ExWAS - Exome-Wide Association Study; WGS - Whole Genome Sequencing; AF - Atrial Fibrillation; INT - Inverse Normal Transformed; SKAT - Sequence Based Kernel Association Test; Christopherson et al 2017a - Reference (58)

Supplementary Table 8 - Reports for targets in each analyses type and prioritisation score

Target	Discovery AF Reports	Discovery PR Reports	Discovery LA Reports	RA TWAS Reports	LV TWAS Reports	RA eQTL Reports	LA eQTL Reports	LV eQTL Reports	RVAS Reports	Priority Score
SH3PXD2A	4	2	0	1	1	0	2	1	1	7
CASQ2	4	1	1	3	2	2	0	2	0	7
THRB	3	1	0	2	1	1	0	1	0	6
TBX5	7	7	0	1	1	0	4	3	0	6
SYNPO2L	8	2	0	2	1	1	2	0	0	6
SCN10A	1	7	0	1	1	0	1	0	1	6
PRRX1	7	1	0	1	2	0	2	1	0	6
MTSS1	1	1	0	2	2	1	0	2	0	6
KCNJ5	5	0	0	2	1	1	2	2	0	6
CEP68	6	0	0	1	2	3	3	3	0	6
AOPEP	7	0	1	2	0	1	1	1	0	6
TMEM182	0	2	0	1	1	1	1	1	0	6
SYNE2	0	1	0	1	3	1	2	4	0	6
PDZRN3	0	3	0	1	1	1	1	1	0	6
FAT1	0	3	0	1	1	1	1	1	0	6
WIPF1	4	0	0	1	2	0	1	2	0	5
SCN5A	4	11	0	1	0	0	1	0	1	5
RPL3L	4	0	0	2	1	1	0	0	1	5
NUCKS1	4	0	0	1	1	1	0	1	0	5
NKX2-5	4	1	0	1	1	0	1	0	0	5
MAPT	2	0	0	2	2	1	0	1	0	5
KDM1B	4	0	0	3	2	3	0	2	0	5
GNB4	4	0	0	2	2	1	0	1	0	5
GMCL1	1	0	0	1	2	0	1	1	0	5
FAM13B	1	0	0	2	1	1	1	0	0	5
SLC25A26	0	1	0	1	2	1	0	1	0	5
MYOZ1	0	1	0	3	1	5	2	0	0	5
EDN2	0	1	0	1	1	1	0	1	0	5
DEK	0	1	0	3	3	1	0	3	0	5
CDH13	0	2	0	1	1	1	0	1	0	5
GOLGA2P7	0	0	0	1	1	2	1	3	0	5
SSBP3	0	0	0	1	1	2	1	2	0	5

Target	Discovery AF Reports	Discovery PR Reports	Discovery LA Reports	RA TWAS Reports	LV TWAS Reports	RA eQTL Reports	LA eQTL Reports	LV eQTL Reports	RVAS Reports	Priority Score
GBAP1	0	0	0	1	1	2	1	2	0	5
TCTN3	0	0	0	1	1	1	1	1	0	5
ZNF664	1	0	0	1	1	0	0	1	0	4
TMEM40	1	0	0	1	1	0	1	0	0	4
SNRNP27	4	0	0	0	0	1	1	1	0	4
PLN	4	4	0	1	1	0	0	0	0	4
PLEC	1	1	0	0	0	2	0	1	0	4
NEURL1	9	0	0	2	1	0	1	0	0	4
KCND3	2	4	0	1	1	0	0	0	0	4
GYPC	2	0	0	1	0	1	0	1	0	4
GJA1	1	2	0	0	1	0	2	0	0	4
FBXO32	3	1	0	1	1	0	0	0	0	4
CAV1	8	11	0	2	0	0	4	0	0	4
ASAH1	5	0	0	0	1	0	2	2	0	4
ARNT2	4	0	0	2	1	1	0	0	0	4
TRAK1	0	1	0	1	0	1	0	1	0	4
STRN	0	1	0	0	1	1	0	1	0	4
PLPP7	0	1	0	1	1	0	0	1	0	4
LRCH1	0	4	0	0	1	0	1	1	0	4
BNIP1	0	2	0	1	1	0	1	0	0	4
RP11-236B18.2	0	0	0	1	1	1	0	1	0	4
KCNJ5-AS1	0	0	0	1	1	0	1	2	0	4
CCDC92	0	0	0	1	2	1	0	1	0	4
FUT11	0	0	0	2	2	0	1	1	0	4
TPMT	0	0	0	3	2	2	0	1	0	4
MLF1	0	0	0	1	1	1	0	1	0	4
CFAP68	0	0	0	1	1	1	0	1	0	4
TRIP4	0	0	0	1	1	1	0	1	0	4
WDR73	0	0	0	1	1	1	0	2	0	4
FKBP7	0	0	0	1	2	1	1	0	0	4
CSPG4P11	0	0	0	1	1	2	1	0	0	4
DNAH10OS	0	0	0	1	1	1	0	1	0	4

Target	Discovery AF Reports	Discovery PR Reports	Discovery LA Reports	RA TWAS Reports	LV TWAS Reports	RA eQTL Reports	LA eQTL Reports	LV eQTL Reports	RVAS Reports	Priority Score
MKRN2	0	0	0	1	1	0	1	1	0	4
XPO7	4	1	0	0	1	0	0	0	0	3
STIM1	1	0	0	1	1	0	0	0	0	3
SPATS2L	4	0	0	1	1	0	0	0	0	3
SIRT1	2	0	0	1	1	0	0	0	0	3
PSMB7	1	0	0	1	1	0	0	0	0	3
PLEKHA3	6	0	1	0	0	0	1	0	0	3
PITX2	16	2	0	0	0	0	1	0	0	3
PHLDB2	2	0	0	1	1	0	0	0	0	3
PGP	1	0	0	1	1	0	0	0	0	3
PFDN1	1	0	0	1	1	0	0	0	0	3
PERM1	1	0	0	1	1	0	0	0	0	3
NTMT2	6	0	0	0	2	0	1	0	0	3
NR3C1	4	2	0	0	1	0	0	0	0	3
NACA	3	0	0	1	1	0	0	0	0	3
MYOCD	4	2	0	0	0	0	1	0	0	3
MYH6	5	3	0	0	0	0	0	0	1	3
MICU2	1	2	0	0	1	0	0	0	0	3
LRRC10	2	0	0	1	1	0	0	0	0	3
KLF12	1	1	0	1	0	0	0	0	0	3
IGF1R	4	0	0	0	2	0	0	1	0	3
HCN4	7	1	0	0	0	0	1	0	0	3
FBN2	3	0	0	0	2	1	0	0	0	3
ESR2	7	0	0	1	0	0	1	0	0	3
DPF3	3	0	0	1	1	0	0	0	0	3
CFL2	4	0	0	1	1	0	0	0	0	3
CEP85L	2	1	0	0	0	0	1	0	0	3
CDKN1A	4	1	0	0	1	0	0	0	0	3
CCDC134	1	0	0	1	1	0	0	0	0	3
CAV2	1	0	0	1	0	0	3	0	0	3
CAND2	6	2	0	0	0	0	1	0	0	3
CAMK2D	4	3	0	0	1	0	0	0	0	3

Target	Discovery AF Reports	Discovery PR Reports	Discovery LA Reports	RA TWAS Reports	LV TWAS Reports	RA eQTL Reports	LA eQTL Reports	LV eQTL Reports	RVAS Reports	Priority Score
C10orf71	1	1	0	0	1	0	0	0	0	3
BHLHE41	4	1	0	0	1	0	0	0	0	3
AKAP6	4	1	0	1	0	0	0	0	0	3
ADAM15	1	0	0	0	1	0	2	0	0	3
ABHD17C	1	0	0	1	1	0	0	0	0	3
TTN	0	4	0	0	0	0	1	0	3	3
SMARCB1	0	1	0	1	1	0	0	0	0	3
RCAN2	0	1	0	1	0	1	0	0	0	3
PRKCA	0	1	0	0	0	1	0	1	0	3
IL17D	0	1	0	0	1	0	0	1	0	3
HCN1	0	3	0	1	0	1	0	0	0	3
FADS1	0	3	0	0	1	0	0	1	0	3
FAAP20	0	2	0	1	1	0	0	0	0	3
EMB	0	1	0	1	1	0	0	0	0	3
DNAH11	0	1	0	1	0	1	0	0	0	3
DAG1	0	1	0	0	1	0	0	1	0	3
CHRM2	0	1	0	0	1	0	0	1	0	3
CBX8	0	1	0	1	0	0	0	1	0	3
BMPR1A	0	1	0	1	1	0	0	0	0	3
BMP8A	0	1	0	0	0	1	0	1	0	3
ALPK3	0	2	0	1	0	1	0	0	0	3
RP1-79C4.4	0	0	0	1	1	0	0	2	0	3
AGAP5	0	0	0	2	1	0	1	0	0	3
DCST2	0	0	0	2	1	0	1	0	0	3
MTHFD1	0	0	0	1	1	0	1	0	0	3
PLAU	0	0	0	2	0	1	1	0	0	3
REEP2	0	0	0	2	2	0	0	1	0	3
SSPN	0	0	0	1	1	1	0	0	0	3
STN1	0	0	0	1	1	0	1	0	0	3
UBE2D3	0	0	0	1	2	0	0	1	0	3
ZBTB7B	0	0	0	1	1	0	1	0	0	3
ZSWIM8	0	0	0	1	1	0	1	0	0	3

Target	Discovery AF Reports	Discovery PR Reports	Discovery LA Reports	RA TWAS Reports	LV TWAS Reports	RA eQTL Reports	LA eQTL Reports	LV eQTL Reports	RVAS Reports	Priority Score
PCGF5	0	0	0	1	0	1	0	1	0	3
UBE2Q2L	0	0	0	1	0	2	0	2	0	3
MRPL37	0	0	0	1	0	2	0	1	0	3
SPATA20	0	0	0	1	1	0	0	1	0	3
DDX42	0	0	0	1	0	1	0	1	0	3
SYPL2	0	0	0	1	1	0	1	0	0	3
SSXP10	0	0	0	1	0	2	0	1	0	3
CALHM2	0	0	0	1	1	0	1	0	0	3
ZNF503-AS1	0	0	0	1	1	1	0	0	0	3
QRICH1	0	0	0	0	1	1	0	1	0	3
IHO1	0	0	0	0	1	1	0	1	0	3
RP11-3B7.1	0	0	0	0	1	1	0	1	0	3
PCM1	0	0	0	0	2	0	2	2	0	3
CAB39L	0	0	0	0	1	1	0	1	0	3
PDZRN3-AS1	0	0	0	0	0	1	1	1	0	3
ZNF292	2	0	0	0	1	0	0	0	0	2
ZMIZ1	1	1	0	0	0	0	0	0	0	2
ZEB2	3	1	0	0	0	0	0	0	0	2
WNT8A	5	0	0	0	0	0	1	0	0	2
WNT3	1	0	0	0	1	0	0	0	0	2
TLE3	2	2	0	0	0	0	0	0	0	2
TGM2	1	1	0	0	0	0	0	0	0	2
TBX3	1	5	0	0	0	0	0	0	0	2
TAB2	4	1	0	0	0	0	0	0	0	2
SPON1	1	1	0	0	0	0	0	0	0	2
SOX5	6	7	0	0	0	0	0	0	0	2
SLCO4A1	1	1	0	0	0	0	0	0	0	2
SLC35F1	1	0	0	0	0	0	1	0	0	2
RPL32	1	0	0	0	0	0	1	0	0	2
RAB1A	1	0	0	0	0	0	1	0	0	2
PDE4B	1	0	1	0	0	0	0	0	0	2
NRBF2	1	0	0	0	0	0	0	1	0	2

Target	Discovery AF Reports	Discovery PR Reports	Discovery LA Reports	RA TWAS Reports	LV TWAS Reports	RA eQTL Reports	LA eQTL Reports	LV eQTL Reports	RVAS Reports	Priority Score
NEBL	2	0	0	0	0	0	0	0	1	2
MYO18B	3	0	1	0	0	0	0	0	0	2
MSRB1	1	1	0	0	0	0	0	0	0	2
LRMDA	4	1	0	0	0	0	0	0	0	2
LRIG1	4	1	0	0	0	0	0	0	0	2
LIN54	1	0	0	1	0	0	0	0	0	2
KLHL38	3	2	0	0	0	0	0	0	0	2
KCNN3	9	0	0	0	0	0	1	0	0	2
KCNN2	5	0	0	0	0	0	2	0	0	2
KCNIP2	1	1	0	0	0	0	0	0	0	2
IL6R	2	0	0	1	0	0	0	0	0	2
HSPG2	3	1	0	0	0	0	0	0	0	2
HSF2	7	0	0	0	0	0	1	0	0	2
HDGFL1	1	1	0	0	0	0	0	0	0	2
HAND2	5	2	0	0	0	0	0	0	0	2
GORAB	2	0	0	0	0	0	1	0	0	2
GJA5	4	1	0	0	0	0	0	0	0	2
GET4	1	1	0	0	0	0	0	0	0	2
FRMD4B	3	2	0	0	0	0	0	0	0	2
FREM2	1	1	0	0	0	0	0	0	0	2
FOXG1	1	1	0	0	0	0	0	0	0	2
FGF5	3	1	0	0	0	0	0	0	0	2
FANCC	1	0	0	0	0	0	1	0	0	2
FAM133B	4	1	0	0	0	0	0	0	0	2
ETV1	4	1	0	0	0	0	0	0	0	2
ENPEP	1	0	0	0	0	0	1	0	0	2
DNAJC12	1	0	0	0	1	0	0	0	0	2
DBX1	5	2	0	0	0	0	0	0	0	2
CKS1B	1	0	0	0	0	0	1	0	0	2
CGA	1	0	0	0	1	0	0	0	0	2
CCDC7	1	1	0	0	0	0	0	0	0	2
CCBE1	1	1	0	0	0	0	0	0	0	2

Target	Discovery AF Reports	Discovery PR Reports	Discovery LA Reports	RA TWAS Reports	LV TWAS Reports	RA eQTL Reports	LA eQTL Reports	LV eQTL Reports	RVAS Reports	Priority Score
C5orf47	1	0	0	1	0	0	0	0	0	2
C1orf185	3	1	0	0	0	0	0	0	0	2
BEST3	3	0	0	0	1	0	0	0	0	2
ARHGAP10	4	1	0	0	0	0	0	0	0	2
ADAR	2	0	0	0	0	0	1	0	0	2
ABHD10	2	2	0	0	0	0	0	0	0	2
ZNF358	0	1	0	0	0	1	0	0	0	2
ZCCHC24	0	1	0	0	1	0	0	0	0	2
VILL	0	1	0	0	0	1	0	0	0	2
TRDN	0	1	0	0	0	0	1	0	0	2
TESC	0	1	0	0	0	0	0	1	0	2
SKI	0	1	0	0	0	0	1	0	0	2
SETD6	0	1	0	0	0	0	0	1	0	2
SENP2	0	2	0	0	0	0	1	0	0	2
NPR3	0	1	0	0	0	1	0	0	0	2
NMB	0	1	0	0	0	0	1	0	0	2
MYOZ2	0	1	0	0	0	0	0	1	0	2
MYBPHL	0	1	0	1	0	0	0	0	0	2
MEIS1	0	7	0	0	0	0	1	0	0	2
IL25	0	1	0	1	0	0	0	0	0	2
GFM1	0	1	0	0	0	1	0	0	0	2
GAREM1	0	1	0	0	0	1	0	0	0	2
FERMT2	0	1	0	0	0	0	1	0	0	2
DLEC1	0	2	0	0	0	0	0	0	1	2
CHDH	0	1	0	0	0	0	0	1	0	2
CDC7	0	1	0	0	0	0	0	1	0	2
BEND7	0	1	0	1	0	0	0	0	0	2
ADCY5	0	1	0	0	0	1	0	0	0	2
ANKRD1	0	0	1	0	0	0	0	1	0	2
AL590822.1	0	0	0	1	0	1	0	0	0	2
RP11-1070N10.3	0	0	0	1	0	1	0	0	0	2

Target	Discovery AF Reports	Discovery PR Reports	Discovery LA Reports	RA TWAS Reports	LV TWAS Reports	RA eQTL Reports	LA eQTL Reports	LV eQTL Reports	RVAS Reports	Priority Score
RP11-210M15.2	0	0	0	2	0	1	0	0	0	2
RP11-399K21.11	0	0	0	1	0	1	0	0	0	2
RP11-464F9.22	0	0	0	1	1	0	0	0	0	2
ACVR2A	0	0	0	2	1	0	0	0	0	2
BAZ2A	0	0	0	1	1	0	0	0	0	2
C4A	0	0	0	1	1	0	0	0	0	2
CBX6	0	0	0	1	1	0	0	0	0	2
CCDC116	0	0	0	1	1	0	0	0	0	2
MIX23	0	0	0	1	1	0	0	0	0	2
DESI1	0	0	0	1	1	0	0	0	0	2
ERBB2	0	0	0	1	1	0	0	0	0	2
FBXL20	0	0	0	1	1	0	0	0	0	2
FOXK1	0	0	0	1	1	0	0	0	0	2
HAGHL	0	0	0	1	2	0	0	0	0	2
HERC4	0	0	0	1	1	0	0	0	0	2
JAM2	0	0	0	1	1	0	0	0	0	2
CTXND1	0	0	0	1	1	0	0	0	0	2
LINC01629	0	0	0	1	1	0	0	0	0	2
MAIP1	0	0	0	1	1	0	0	0	0	2
MED1	0	0	0	1	1	0	0	0	0	2
MMP11	0	0	0	2	2	0	0	0	0	2
NDUFB10	0	0	0	1	1	0	0	0	0	2
ORC3	0	0	0	1	1	0	0	0	0	2
PBXIP1	0	0	0	1	0	0	1	0	0	2
PGAP3	0	0	0	1	2	0	0	0	0	2
PIK3C2B	0	0	0	1	1	0	0	0	0	2
PITX3	0	0	0	1	1	0	0	0	0	2
PMVK	0	0	0	1	0	0	1	0	0	2
PNMT	0	0	0	1	1	0	0	0	0	2
PRKRA	0	0	0	1	0	0	1	0	0	2
PROZ	0	0	0	1	1	0	0	0	0	2

Target	Discovery AF Reports	Discovery PR Reports	Discovery LA Reports	RA TWAS Reports	LV TWAS Reports	RA eQTL Reports	LA eQTL Reports	LV eQTL Reports	RVAS Reports	Priority Score
RDH5	0	0	0	1	1	0	0	0	0	2
RNF11	0	0	0	1	1	0	0	0	0	2
SF3B1	0	0	0	1	1	0	0	0	0	2
SLC27A6	0	0	0	2	1	0	0	0	0	2
SLC2A9	0	0	0	1	1	0	0	0	0	2
SLC35A1	0	0	0	1	1	0	0	0	0	2
TYW5	0	0	0	1	1	0	0	0	0	2
SLC6A6	0	0	0	1	0	1	0	0	0	2
NPIPA5	0	0	0	1	0	0	0	1	0	2
GORASP1	0	0	0	1	0	0	0	0	1	2
NDST2	0	0	0	1	0	0	1	0	0	2
SLK	0	0	0	1	0	0	1	0	0	2
NME5	0	0	0	1	0	0	1	0	0	2
RP11-10A14.4	0	0	0	0	1	1	0	0	0	2
DMWD	0	0	0	0	1	0	0	1	0	2
MXD1	0	0	0	0	1	0	1	0	0	2
SEC24C	0	0	0	0	1	0	1	0	0	2
LINC00964	0	0	0	0	1	0	0	2	0	2
EFNA1	0	0	0	0	1	0	0	1	0	2
RPSA	0	0	0	0	1	1	0	0	0	2
VDAC2	0	0	0	0	1	0	0	1	0	2
PCYOX1	0	0	0	0	1	0	0	1	0	2
MKRN2OS	0	0	0	0	1	0	1	0	0	2
MPPED2	0	0	0	0	1	0	0	1	0	2
SYMPK	0	0	0	0	0	1	0	1	0	2
VANGL1	0	0	0	0	0	1	0	1	0	2
OXCT2P1	0	0	0	0	0	2	0	1	0	2
KB-1507C5.2	0	0	0	0	0	1	0	1	0	2
RP11-337C18.8	0	0	0	0	0	1	0	1	0	2
RP11-479G22.8	0	0	0	0	0	1	0	1	0	2
NCKIPSD	0	0	0	0	0	1	0	1	0	2
WDR6	0	0	0	0	0	1	0	1	0	2

Target	Discovery AF Reports	Discovery PR Reports	Discovery LA Reports	RA TWAS Reports	LV TWAS Reports	RA eQTL Reports	LA eQTL Reports	LV eQTL Reports	RVAS Reports	Priority Score
FABP2	0	0	0	0	0	1	0	1	0	2
SLC25A27	0	0	0	0	0	1	0	1	0	2
TDRD6	0	0	0	0	0	1	0	1	0	2
FTSJ3	0	0	0	0	0	1	0	1	0	2
MEI1	0	0	0	0	0	1	0	1	0	2
DUSP8P5	0	0	0	0	0	1	1	0	0	2
RP11-145M9.4	0	0	0	0	0	1	0	1	0	2
RP11-7F17.1	0	0	0	0	0	1	0	1	0	2
RP11-7F17.7	0	0	0	0	0	1	0	1	0	2
PM20D1	0	0	0	0	0	1	0	1	0	2
RAB29	0	0	0	0	0	1	0	1	0	2
RN7SL356P	0	0	0	0	0	1	0	1	0	2
ANXA4	0	0	0	0	0	0	1	1	0	2
RP11-806O11.1	0	0	0	0	0	0	1	1	0	2
USP54	0	0	0	0	0	0	1	1	0	2
RP11-137L10.6	0	0	0	0	0	0	1	1	0	2
ZSCAN20	1	0	0	0	0	0	0	0	0	1
ZRANB1	1	0	0	0	0	0	0	0	0	1
ZPBP2	3	0	0	0	0	0	0	0	0	1
ZNF879	1	0	0	0	0	0	0	0	0	1
ZNF862	1	0	0	0	0	0	0	0	0	1
ZNF689	1	0	0	0	0	0	0	0	0	1
ZNF462	1	0	0	0	0	0	0	0	0	1
ZNF25	1	0	0	0	0	0	0	0	0	1
ZIC3	1	0	0	0	0	0	0	0	0	1
YWHAE	3	0	0	0	0	0	0	0	0	1
YARS2	4	0	0	0	0	0	0	0	0	1
XXYLT1	3	0	0	0	0	0	0	0	0	1
XPO1	2	0	0	0	0	0	0	0	0	1
WDR27	1	0	0	0	0	0	0	0	0	1
WDR1	2	0	0	0	0	0	0	0	0	1
VWDE	1	0	0	0	0	0	0	0	0	1

Target	Discovery AF Reports	Discovery PR Reports	Discovery LA Reports	RA TWAS Reports	LV TWAS Reports	RA eQTL Reports	LA eQTL Reports	LV eQTL Reports	RVAS Reports	Priority Score
VPS13D	1	0	0	0	0	0	0	0	0	1
VPS13C	1	0	0	0	0	0	0	0	0	1
UTP4	1	0	0	0	0	0	0	0	0	1
USP34	1	0	0	0	0	0	0	0	0	1
USP3	2	0	0	0	0	0	0	0	0	1
URB2	1	0	0	0	0	0	0	0	0	1
UBE4B	1	0	0	0	0	0	0	0	0	1
TUBB3	1	0	0	0	0	0	0	0	0	1
TUBA8	4	0	0	0	0	0	0	0	0	1
TTC29	1	0	0	0	0	0	0	0	0	1
TRPM2	1	0	0	0	0	0	0	0	0	1
TRIM40	1	0	0	0	0	0	0	0	0	1
TP53INP1	1	0	0	0	0	0	0	0	0	1
TNNI3	1	0	0	0	0	0	0	0	0	1
TNFSF13	1	0	0	0	0	0	0	0	0	1
TNFSF12-TNFSF13	1	0	0	0	0	0	0	0	0	1
TNFSF12	2	0	0	0	0	0	0	0	0	1
TMX4	1	0	0	0	0	0	0	0	0	1
TMT1B	1	0	0	0	0	0	0	0	0	1
TMEM245	1	0	0	0	0	0	0	0	0	1
TMCO3	1	0	0	0	0	0	0	0	0	1
TGIF1	1	0	0	0	0	0	0	0	0	1
SYNE1	1	0	0	0	0	0	0	0	0	1
SYK	1	0	0	0	0	0	0	0	0	1
SYDE2	1	0	0	0	0	0	0	0	0	1
SULF2	1	0	0	0	0	0	0	0	0	1
SPIDR	1	0	0	0	0	0	0	0	0	1
SPEN	1	0	0	0	0	0	0	0	0	1
SPDYE21	1	0	0	0	0	0	0	0	0	1
SORL1	3	0	0	0	0	0	0	0	0	1
SNX19	1	0	0	0	0	0	0	0	0	1

Target	Discovery AF Reports	Discovery PR Reports	Discovery LA Reports	RA TWAS Reports	LV TWAS Reports	RA eQTL Reports	LA eQTL Reports	LV eQTL Reports	RVAS Reports	Priority Score
SNAPC1	1	0	0	0	0	0	0	0	0	1
SMAD7	4	0	0	0	0	0	0	0	0	1
SLIT3	3	0	0	0	0	0	0	0	0	1
SLFNL1	3	0	0	0	0	0	0	0	0	1
SLCO6A1	1	0	0	0	0	0	0	0	0	1
SLC9B1	3	0	0	0	0	0	0	0	0	1
SLC4A7	1	0	0	0	0	0	0	0	0	1
SLC22A24	1	0	0	0	0	0	0	0	0	1
SLC1A7	1	0	0	0	0	0	0	0	0	1
SLC18A3	1	0	0	0	0	0	0	0	0	1
SLC15A5	1	0	0	0	0	0	0	0	0	1
SLAMF7	1	0	0	0	0	0	0	0	0	1
SLA2	1	0	0	0	0	0	0	0	0	1
SGCG	4	0	0	0	0	0	0	0	0	1
SETD7	1	0	0	0	0	0	0	0	0	1
SESN3	1	0	0	0	0	0	0	0	0	1
SEMA6A	1	0	0	0	0	0	0	0	0	1
SDF2L1	1	0	0	0	0	0	0	0	0	1
SCYL3	1	0	0	0	0	0	0	0	0	1
SCYL2	1	0	0	0	0	0	0	0	0	1
SALL4	1	0	0	0	0	0	0	0	0	1
RUNDC3A	1	0	0	0	0	0	0	0	0	1
RTKN2	1	0	0	0	0	0	0	0	0	1
RPS2	2	0	0	0	0	0	0	0	0	1
RPRML	3	0	0	0	0	0	0	0	0	1
RHBDL2	1	0	0	0	0	0	0	0	0	1
REEP1	4	0	0	0	0	0	0	0	0	1
RASGRF1	1	0	0	0	0	0	0	0	0	1
RARRES1	1	0	0	0	0	0	0	0	0	1
RAPGEF5	1	0	0	0	0	0	0	0	0	1
PTGES3	2	0	0	0	0	0	0	0	0	1
PTCHD1	1	0	0	0	0	0	0	0	0	1

Target	Discovery AF Reports	Discovery PR Reports	Discovery LA Reports	RA TWAS Reports	LV TWAS Reports	RA eQTL Reports	LA eQTL Reports	LV eQTL Reports	RVAS Reports	Priority Score
PSMG1	1	0	0	0	0	0	0	0	0	1
POT1	1	0	0	0	0	0	0	0	0	1
POLR2M	1	0	0	0	0	0	0	0	0	1
POLR2A	1	0	0	0	0	0	0	0	0	1
PMFBP1	11	0	0	0	0	0	0	0	0	1
PLCD3	2	0	0	0	0	0	0	0	0	1
PKD1L1	1	0	0	0	0	0	0	0	0	1
PHLDA1	4	0	0	0	0	0	0	0	0	1
PHETA1	2	0	0	0	0	0	0	0	0	1
PFAS	1	0	0	0	0	0	0	0	0	1
PDCD4	3	0	0	0	0	0	0	0	0	1
PCID2	3	0	0	0	0	0	0	0	0	1
PBX1	1	0	0	0	0	0	0	0	0	1
PAPPA	1	0	0	0	0	0	0	0	0	1
PALMD	1	0	0	0	0	0	0	0	0	1
ORC4	1	0	0	0	0	0	0	0	0	1
OR4X2	1	0	0	0	0	0	0	0	0	1
OPN1SW	2	0	0	0	0	0	0	0	0	1
OAS1	1	0	0	0	0	0	0	0	0	1
NTF3	1	0	0	0	0	0	0	0	0	1
NLRX1	1	0	0	0	0	0	0	0	0	1
NGB	1	0	0	0	0	0	0	0	0	1
NFATC2	1	0	0	0	0	0	0	0	0	1
NFATC1	1	0	0	0	0	0	0	0	0	1
NEUROG2	1	0	0	0	0	0	0	0	0	1
NEURL	1	0	0	0	0	0	0	0	0	1
NCOR2	1	0	0	0	0	0	0	0	0	1
NCF1	1	0	0	0	0	0	0	0	0	1
NARS2	1	0	0	0	0	0	0	0	0	1
N4BP2	1	0	0	0	0	0	0	0	0	1
MYOG	5	0	0	0	0	0	0	0	0	1
MYL4	2	0	0	0	0	0	0	0	0	1

Target	Discovery AF Reports	Discovery PR Reports	Discovery LA Reports	RA TWAS Reports	LV TWAS Reports	RA eQTL Reports	LA eQTL Reports	LV eQTL Reports	RVAS Reports	Priority Score
MYH11	1	0	0	0	0	0	0	0	0	1
MUS81	1	0	0	0	0	0	0	0	0	1
MSL2	3	0	0	0	0	0	0	0	0	1
MOB1B	1	0	0	0	0	0	0	0	0	1
MLLT3	2	0	0	0	0	0	0	0	0	1
MGAT5	1	0	0	0	0	0	0	0	0	1
MEX3C	2	0	0	0	0	0	0	0	0	1
METRN	1	0	0	0	0	0	0	0	0	1
MCM10	1	0	0	0	0	0	0	0	0	1
MAP3K14	1	0	0	0	0	0	0	0	0	1
MANBA	1	0	0	0	0	0	0	0	0	1
LYSMD3	1	0	0	0	0	0	0	0	0	1
LYRM2	1	0	0	0	0	0	0	0	0	1
LSM12	1	0	0	0	0	0	0	0	0	1
LRCOL1	2	0	0	0	0	0	0	0	0	1
LHX3	3	0	0	0	0	0	0	0	0	1
KNL1	1	0	0	0	0	0	0	0	0	1
KLHL3	1	0	0	0	0	0	0	0	0	1
KLF17	1	0	0	0	0	0	0	0	0	1
KIF7	1	0	0	0	0	0	0	0	0	1
KIF3C	4	0	0	0	0	0	0	0	0	1
KIF15	1	0	0	0	0	0	0	0	0	1
KCNJ2	2	0	0	0	0	0	0	0	0	1
KCNIP1	1	0	0	0	0	0	0	0	0	1
KCNH2	4	0	0	0	0	0	0	0	0	1
KATNAL1	1	0	0	0	0	0	0	0	0	1
JMJD1C	4	0	0	0	0	0	0	0	0	1
ITGA2B	1	0	0	0	0	0	0	0	0	1
IRF2BPL	4	0	0	0	0	0	0	0	0	1
INKA2	4	0	0	0	0	0	0	0	0	1
IMPDH1	1	0	0	0	0	0	0	0	0	1
IL24	1	0	0	0	0	0	0	0	0	1

Target	Discovery AF Reports	Discovery PR Reports	Discovery LA Reports	RA TWAS Reports	LV TWAS Reports	RA eQTL Reports	LA eQTL Reports	LV eQTL Reports	RVAS Reports	Priority Score
HRURF	1	0	0	0	0	0	0	0	0	1
HP	2	0	0	0	0	0	0	0	0	1
HNRNPC	1	0	0	0	0	0	0	0	0	1
HMGA1	1	0	0	0	0	0	0	0	0	1
HLA-DPA1	1	0	0	0	0	0	0	0	0	1
HIP1R	3	0	0	0	0	0	0	0	0	1
HERC1	1	0	0	0	0	0	0	0	0	1
HELZ	1	0	0	0	0	0	0	0	0	1
HCFC2	1	0	0	0	0	0	0	0	0	1
HBP1	1	0	0	0	0	0	0	0	0	1
HBEGF	1	0	0	0	0	0	0	0	0	1
H2BC4	1	0	0	0	0	0	0	0	0	1
GTF2I	2	0	0	0	0	0	0	0	0	1
GMPR	4	0	0	0	0	0	0	0	0	1
GGACT	1	0	0	0	0	0	0	0	0	1
GCOM1	3	0	0	0	0	0	0	0	0	1
GCG	1	0	0	0	0	0	0	0	0	1
GC	1	0	0	0	0	0	0	0	0	1
GATA5	1	0	0	0	0	0	0	0	0	1
GATA4	3	0	0	0	0	0	0	0	0	1
GATA2	1	0	0	0	0	0	0	0	0	1
FTCD	1	0	0	0	0	0	0	0	0	1
FOXN4	1	0	0	0	0	0	0	0	0	1
FOXC1	1	0	0	0	0	0	0	0	0	1
FILIP1	1	0	0	0	0	0	0	0	0	1
FGF13	1	0	0	0	0	0	0	0	0	1
FCRL1	1	0	0	0	0	0	0	0	0	1
FBRSL1	1	0	0	0	0	0	0	0	0	1
FANCL	1	0	0	0	0	0	0	0	0	1
FAM241A	2	0	0	0	0	0	0	0	0	1
FAM222A	1	0	0	0	0	0	0	0	0	1
EYA4	1	0	0	0	0	0	0	0	0	1

Target	Discovery AF Reports	Discovery PR Reports	Discovery LA Reports	RA TWAS Reports	LV TWAS Reports	RA eQTL Reports	LA eQTL Reports	LV eQTL Reports	RVAS Reports	Priority Score
EXT1	1	0	0	0	0	0	0	0	0	1
ERBB4	3	0	0	0	0	0	0	0	0	1
EPS15	1	0	0	0	0	0	0	0	0	1
EPN1	1	0	0	0	0	0	0	0	0	1
EPHA3	4	0	0	0	0	0	0	0	0	1
ELOVL3	1	0	0	0	0	0	0	0	0	1
ELOA2	1	0	0	0	0	0	0	0	0	1
EHD4	1	0	0	0	0	0	0	0	0	1
EFNA5	3	0	0	0	0	0	0	0	0	1
EFHD1	1	0	0	0	0	0	0	0	0	1
DNAI2	1	0	0	0	0	0	0	0	0	1
DNAH17	1	0	0	0	0	0	0	0	0	1
DMRTA2	1	0	0	0	0	0	0	0	0	1
DEFB136	1	0	0	0	0	0	0	0	0	1
CYTH1	2	0	0	0	0	0	0	0	0	1
CUX2	1	0	0	0	0	0	0	0	0	1
CTU2	1	0	0	0	0	0	0	0	0	1
CTNNA3	1	0	0	0	0	0	0	0	0	1
CSPG4	1	0	0	0	0	0	0	0	0	1
CREB5	4	0	0	0	0	0	0	0	0	1
CRAMP1	1	0	0	0	0	0	0	0	0	1
CPSF6	1	0	0	0	0	0	0	0	0	1
CMYA5	1	0	0	0	0	0	0	0	0	1
CLIC6	4	0	0	0	0	0	0	0	0	1
CHST14	1	0	0	0	0	0	0	0	0	1
CEP152	1	0	0	0	0	0	0	0	0	1
CDHR3	1	0	0	0	0	0	0	0	0	1
CDC42BPG	1	0	0	0	0	0	0	0	0	1
CD96	1	0	0	0	0	0	0	0	0	1
CD69	1	0	0	0	0	0	0	0	0	1
CCDC71	1	0	0	0	0	0	0	0	0	1
CCDC168	1	0	0	0	0	0	0	0	0	1

Target	Discovery AF Reports	Discovery PR Reports	Discovery LA Reports	RA TWAS Reports	LV TWAS Reports	RA eQTL Reports	LA eQTL Reports	LV eQTL Reports	RVAS Reports	Priority Score
CASZ1	4	0	0	0	0	0	0	0	0	1
CACFD1	1	0	0	0	0	0	0	0	0	1
CA4	1	0	0	0	0	0	0	0	0	1
C9orf3	1	0	0	0	0	0	0	0	0	1
C8orf74	1	0	0	0	0	0	0	0	0	1
C2orf74	1	0	0	0	0	0	0	0	0	1
BMP2	1	0	0	0	0	0	0	0	0	1
BLK	1	0	0	0	0	0	0	0	0	1
BLID	1	0	0	0	0	0	0	0	0	1
BICRA	1	0	0	0	0	0	0	0	0	1
BEND5	2	0	0	0	0	0	0	0	0	1
BCKDHB	1	0	0	0	0	0	0	0	0	1
B3GALT1	1	0	0	0	0	0	0	0	0	1
AXDND1	1	0	0	0	0	0	0	0	0	1
ASB13	1	0	0	0	0	0	0	0	0	1
ARRDC4	1	0	0	0	0	0	0	0	0	1
ARHGAP8	1	0	0	0	0	0	0	0	0	1
ARFGEF1	1	0	0	0	0	0	0	0	0	1
APOLD1	1	0	0	0	0	0	0	0	0	1
ALPK2	1	0	0	0	0	0	0	0	0	1
ALDH8A1	1	0	0	0	0	0	0	0	0	1
AGO2	4	0	0	0	0	0	0	0	0	1
AGA	1	0	0	0	0	0	0	0	0	1
ADRA1A	1	0	0	0	0	0	0	0	0	1
ADCY7	1	0	0	0	0	0	0	0	0	1
ADCY3	1	0	0	0	0	0	0	0	0	1
ADAT1	1	0	0	0	0	0	0	0	0	1
ADAM11	1	0	0	0	0	0	0	0	0	1
ACER1	1	0	0	0	0	0	0	0	0	1
ZNF608	0	1	0	0	0	0	0	0	0	1
ZNF572	0	1	0	0	0	0	0	0	0	1
ZNF438	0	1	0	0	0	0	0	0	0	1

Target	Discovery AF Reports	Discovery PR Reports	Discovery LA Reports	RA TWAS Reports	LV TWAS Reports	RA eQTL Reports	LA eQTL Reports	LV eQTL Reports	RVAS Reports	Priority Score
ZNF281	0	1	0	0	0	0	0	0	0	1
ZFPM2	0	1	0	0	0	0	0	0	0	1
ZFPM1	0	1	0	0	0	0	0	0	0	1
ZDHHC20	0	2	0	0	0	0	0	0	0	1
ZC3H15	0	1	0	0	0	0	0	0	0	1
XYLB	0	4	0	0	0	0	0	0	0	1
XPO4	0	1	0	0	0	0	0	0	0	1
WT1	0	1	0	0	0	0	0	0	0	1
WRNIP1	0	2	0	0	0	0	0	0	0	1
WNT11	0	2	0	0	0	0	0	0	0	1
TTC39A	0	1	0	0	0	0	0	0	0	1
TSC22D2	0	1	0	0	0	0	0	0	0	1
TRIM32	0	1	0	0	0	0	0	0	0	1
TRAM2	0	1	0	0	0	0	0	0	0	1
TMEM72	0	1	0	0	0	0	0	0	0	1
TMEM26	0	1	0	0	0	0	0	0	0	1
TM2D1	0	1	0	0	0	0	0	0	0	1
THSD4	0	1	0	0	0	0	0	0	0	1
THRAP3	0	1	0	0	0	0	0	0	0	1
TFEB	0	1	0	0	0	0	0	0	0	1
TFAP4	0	1	0	0	0	0	0	0	0	1
TCF21	0	2	0	0	0	0	0	0	0	1
TBX20	0	1	0	0	0	0	0	0	0	1
SYNE3	0	1	0	0	0	0	0	0	0	1
STARD4	0	1	0	0	0	0	0	0	0	1
SSH2	0	1	0	0	0	0	0	0	0	1
SRL	0	1	0	0	0	0	0	0	0	1
SRGAP1	0	1	0	0	0	0	0	0	0	1
SRD5A3	0	1	0	0	0	0	0	0	0	1
SPATA31D1	0	1	0	0	0	0	0	0	0	1
SOX11	0	1	0	0	0	0	0	0	0	1
SORT1	0	1	0	0	0	0	0	0	0	1

Target	Discovery AF Reports	Discovery PR Reports	Discovery LA Reports	RA TWAS Reports	LV TWAS Reports	RA eQTL Reports	LA eQTL Reports	LV eQTL Reports	RVAS Reports	Priority Score
SORBS1	0	2	0	0	0	0	0	0	0	1
SMYD3	0	1	0	0	0	0	0	0	0	1
SLC8A1	0	3	0	0	0	0	0	0	0	1
SLC6A4	0	1	0	0	0	0	0	0	0	1
SLC6A15	0	1	0	0	0	0	0	0	0	1
SLC50A1	0	1	0	0	0	0	0	0	0	1
SLC12A7	0	1	0	0	0	0	0	0	0	1
SIPA1L2	0	1	0	0	0	0	0	0	0	1
SIPA1L1	0	4	0	0	0	0	0	0	0	1
SH3BGRL3	0	1	0	0	0	0	0	0	0	1
SFR1	0	1	0	0	0	0	0	0	0	1
SEMA3A	0	1	0	0	0	0	0	0	0	1
SELENOM	0	1	0	0	0	0	0	0	0	1
SEC11A	0	1	0	0	0	0	0	0	0	1
SAMD8	0	1	0	0	0	0	0	0	0	1
RYR2	0	1	0	0	0	0	0	0	0	1
RNF24	0	1	0	0	0	0	0	0	0	1
RIC8B	0	1	0	0	0	0	0	0	0	1
RBPMS2	0	1	0	0	0	0	0	0	0	1
RBPMS	0	1	0	0	0	0	0	0	0	1
RAP2B	0	1	0	0	0	0	0	0	0	1
QKI	0	1	0	0	0	0	0	0	0	1
PTK2	0	1	0	0	0	0	0	0	0	1
PRPS1L1	0	1	0	0	0	0	0	0	0	1
PRKAB2	0	1	0	0	0	0	0	0	0	1
PPARGC1A	0	1	0	0	0	0	0	0	0	1
PKP1	0	1	0	0	0	0	0	0	0	1
PHIP	0	1	0	0	0	0	0	0	0	1
PHF5A	0	1	0	0	0	0	0	0	0	1
PHF20L1	0	1	0	0	0	0	0	0	0	1
PDLIM5	0	1	0	0	0	0	0	0	0	1
PDLIM1	0	1	0	0	0	0	0	0	0	1

Target	Discovery AF Reports	Discovery PR Reports	Discovery LA Reports	RA TWAS Reports	LV TWAS Reports	RA eQTL Reports	LA eQTL Reports	LV eQTL Reports	RVAS Reports	Priority Score
PDE3A	0	1	0	0	0	0	0	0	0	1
PCNX3	0	1	0	0	0	0	0	0	0	1
PCDH18	0	1	0	0	0	0	0	0	0	1
PAM	0	2	0	0	0	0	0	0	0	1
OXR1	0	2	0	0	0	0	0	0	0	1
OBSCN	0	2	0	0	0	0	0	0	0	1
NSG2	0	1	0	0	0	0	0	0	0	1
NR2F2	0	1	0	0	0	0	0	0	0	1
NR2F1	0	1	0	0	0	0	0	0	0	1
NKD2	0	1	0	0	0	0	0	0	0	1
NFYB	0	1	0	0	0	0	0	0	0	1
NFKBIA	0	1	0	0	0	0	0	0	0	1
NDRG2	0	1	0	0	0	0	0	0	0	1
NDE1	0	1	0	0	0	0	0	0	0	1
NCKAP5	0	1	0	0	0	0	0	0	0	1
MYH7	0	1	0	0	0	0	0	0	0	1
MVK	0	1	0	0	0	0	0	0	0	1
MTUS1	0	1	0	0	0	0	0	0	0	1
MTNR1A	0	1	0	0	0	0	0	0	0	1
MSX2	0	1	0	0	0	0	0	0	0	1
MRPS34	0	1	0	0	0	0	0	0	0	1
MOBP	0	1	0	0	0	0	0	0	0	1
MKLN1	0	2	0	0	0	0	0	0	0	1
MELTF	0	1	0	0	0	0	0	0	0	1
MED13L	0	2	0	0	0	0	0	0	0	1
MC4R	0	1	0	0	0	0	0	0	0	1
MARK3	0	1	0	0	0	0	0	0	0	1
MARCKS	0	1	0	0	0	0	0	0	0	1
MAPRE2	0	1	0	0	0	0	0	0	0	1
MAP7D1	0	1	0	0	0	0	0	0	0	1
MACF1	0	1	0	0	0	0	0	0	0	1
LSM3	0	1	0	0	0	0	0	0	0	1

Target	Discovery AF Reports	Discovery PR Reports	Discovery LA Reports	RA TWAS Reports	LV TWAS Reports	RA eQTL Reports	LA eQTL Reports	LV eQTL Reports	RVAS Reports	Priority Score
LMAN1	0	1	0	0	0	0	0	0	0	1
LLPH	0	1	0	0	0	0	0	0	0	1
LAMB2	0	1	0	0	0	0	0	0	0	1
KRTCAP2	0	2	0	0	0	0	0	0	0	1
KLHL8	0	2	0	0	0	0	0	0	0	1
KLHL26	0	1	0	0	0	0	0	0	0	1
KLF9	0	1	0	0	0	0	0	0	0	1
JAZF1	0	1	0	0	0	0	0	0	0	1
ITGA9	0	3	0	0	0	0	0	0	0	1
ISOC1	0	1	0	0	0	0	0	0	0	1
IRX3	0	1	0	0	0	0	0	0	0	1
IRF8	0	1	0	0	0	0	0	0	0	1
IGFBP5	0	1	0	0	0	0	0	0	0	1
ID2	0	4	0	0	0	0	0	0	0	1
HSPB2	0	1	0	0	0	0	0	0	0	1
HMGA2	0	1	0	0	0	0	0	0	0	1
HLX	0	1	0	0	0	0	0	0	0	1
HERPUD2	0	2	0	0	0	0	0	0	0	1
GOSR2	0	1	0	0	0	0	0	0	0	1
GNS	0	1	0	0	0	0	0	0	0	1
GNPNAT1	0	1	0	0	0	0	0	0	0	1
GIN1	0	1	0	0	0	0	0	0	0	1
GH1	0	1	0	0	0	0	0	0	0	1
FOXP1	0	2	0	0	0	0	0	0	0	1
FNDC3B	0	1	0	0	0	0	0	0	0	1
FLRT2	0	1	0	0	0	0	0	0	0	1
FIGN	0	2	0	0	0	0	0	0	0	1
FHL2	0	1	0	0	0	0	0	0	0	1
FGFR2	0	1	0	0	0	0	0	0	0	1
FGFR1	0	2	0	0	0	0	0	0	0	1
FGF18	0	1	0	0	0	0	0	0	0	1
FGF14	0	1	0	0	0	0	0	0	0	1

Target	Discovery AF Reports	Discovery PR Reports	Discovery LA Reports	RA TWAS Reports	LV TWAS Reports	RA eQTL Reports	LA eQTL Reports	LV eQTL Reports	RVAS Reports	Priority Score
FGF12	0	1	0	0	0	0	0	0	0	1
FER	0	1	0	0	0	0	0	0	0	1
FBXO11	0	1	0	0	0	0	0	0	0	1
FBXL7	0	1	0	0	0	0	0	0	0	1
FAM117B	0	1	0	0	0	0	0	0	0	1
FAF1	0	1	0	0	0	0	0	0	0	1
FADS2	0	2	0	0	0	0	0	0	0	1
EXOG	0	4	0	0	0	0	0	0	0	1
EPPK1	0	1	0	0	0	0	0	0	0	1
EPAS1	0	2	0	0	0	0	0	0	0	1
EOMES	0	2	0	0	0	0	0	0	0	1
ENTPD6	0	1	0	0	0	0	0	0	0	1
EML6	0	1	0	0	0	0	0	0	0	1
EMILIN3	0	1	0	0	0	0	0	0	0	1
ELMO1	0	1	0	0	0	0	0	0	0	1
EEF1AKMT1	0	3	0	0	0	0	0	0	0	1
DSP	0	1	0	0	0	0	0	0	0	1
DPT	0	1	0	0	0	0	0	0	0	1
DERL3	0	1	0	0	0	0	0	0	0	1
CYB5RL	0	2	0	0	0	0	0	0	0	1
CTDSPL	0	1	0	0	0	0	0	0	0	1
CSHL1	0	1	0	0	0	0	0	0	0	1
CRYBG2	0	1	0	0	0	0	0	0	0	1
CREBRF	0	4	0	0	0	0	0	0	0	1
COX7A2L	0	1	0	0	0	0	0	0	0	1
CORO2B	0	1	0	0	0	0	0	0	0	1
COQ8A	0	1	0	0	0	0	0	0	0	1
COL13A1	0	1	0	0	0	0	0	0	0	1
CNKSR3	0	1	0	0	0	0	0	0	0	1
CLDN25	0	1	0	0	0	0	0	0	0	1
CKB	0	1	0	0	0	0	0	0	0	1
CHPF	0	1	0	0	0	0	0	0	0	1

Target	Discovery AF Reports	Discovery PR Reports	Discovery LA Reports	RA TWAS Reports	LV TWAS Reports	RA eQTL Reports	LA eQTL Reports	LV eQTL Reports	RVAS Reports	Priority Score
CHN1	0	1	0	0	0	0	0	0	0	1
CELA3B	0	1	0	0	0	0	0	0	0	1
CDH2	0	1	0	0	0	0	0	0	0	1
CDADC1	0	1	0	0	0	0	0	0	0	1
CCNL1	0	2	0	0	0	0	0	0	0	1
CBARP	0	1	0	0	0	0	0	0	0	1
CAVIN2	0	1	0	0	0	0	0	0	0	1
CASKIN2	0	1	0	0	0	0	0	0	0	1
CARMIL1	0	1	0	0	0	0	0	0	0	1
CALM3	0	1	0	0	0	0	0	0	0	1
CACNA1G	0	1	0	0	0	0	0	0	0	1
C8orf48	0	1	0	0	0	0	0	0	0	1
C1D	0	1	0	0	0	0	0	0	0	1
C17orf107	0	1	0	0	0	0	0	0	0	1
BCAR1	0	1	0	0	0	0	0	0	0	1
AZIN1	0	1	0	0	0	0	0	0	0	1
ARL14EP	0	1	0	0	0	0	0	0	0	1
ARID2	0	1	0	0	0	0	0	0	0	1
ARHGAP44	0	3	0	0	0	0	0	0	0	1
ARHGAP24	0	8	0	0	0	0	0	0	0	1
ALDH18A1	0	1	0	0	0	0	0	0	0	1
ADRB1	0	1	0	0	0	0	0	0	0	1
ADPRHL1	0	2	0	0	0	0	0	0	0	1
ADGRL3	0	1	0	0	0	0	0	0	0	1
ADAMTS5	0	1	0	0	0	0	0	0	0	1
ACVR2B	0	2	0	0	0	0	0	0	0	1
UQCRB	0	0	1	0	0	0	0	0	0	1
SNRPD2	0	0	1	0	0	0	0	0	0	1
SNRNP48	0	0	1	0	0	0	0	0	0	1
SIX5	0	0	1	0	0	0	0	0	0	1
SEPHS1	0	0	1	0	0	0	0	0	0	1
MGAT1	0	0	1	0	0	0	0	0	0	1

Target	Discovery AF Reports	Discovery PR Reports	Discovery LA Reports	RA TWAS Reports	LV TWAS Reports	RA eQTL Reports	LA eQTL Reports	LV eQTL Reports	RVAS Reports	Priority Score
EIF2D	0	0	1	0	0	0	0	0	0	1
COL8A1	0	0	1	0	0	0	0	0	0	1
CITED4	0	0	1	0	0	0	0	0	0	1
CILP	0	0	1	0	0	0	0	0	0	1
BRINP3	0	0	1	0	0	0	0	0	0	1
AC011747.4	0	0	0	1	0	0	0	0	0	1
RP11-724N1.1	0	0	0	1	0	0	0	0	0	1
BTRC	0	0	0	1	0	0	0	0	0	1
CFAP410	0	0	0	1	0	0	0	0	0	1
C2orf69	0	0	0	1	0	0	0	0	0	1
CCT2	0	0	0	1	0	0	0	0	0	1
COPS4	0	0	0	1	0	0	0	0	0	1
DPCD	0	0	0	1	0	0	0	0	0	1
ANTKMT	0	0	0	1	0	0	0	0	0	1
FAM43A	0	0	0	2	0	0	0	0	0	1
FBXL22	0	0	0	1	0	0	0	0	0	1
GPR85	0	0	0	2	0	0	0	0	0	1
GSDMB	0	0	0	1	0	0	0	0	0	1
HPS6	0	0	0	1	0	0	0	0	0	1
ITGB1	0	0	0	1	0	0	0	0	0	1
KDM3B	0	0	0	2	0	0	0	0	0	1
MBD5	0	0	0	1	0	0	0	0	0	1
MSH5	0	0	0	1	0	0	0	0	0	1
CAVIN4	0	0	0	1	0	0	0	0	0	1
ORMDL3	0	0	0	1	0	0	0	0	0	1
PAPLN	0	0	0	1	0	0	0	0	0	1
PCCB	0	0	0	1	0	0	0	0	0	1
PEX26	0	0	0	1	0	0	0	0	0	1
PKP2	0	0	0	1	0	0	0	0	0	1
PPP1R1B	0	0	0	1	0	0	0	0	0	1
RNF144B	0	0	0	1	0	0	0	0	0	1
SEC23IP	0	0	0	1	0	0	0	0	0	1

Target	Discovery AF Reports	Discovery PR Reports	Discovery LA Reports	RA TWAS Reports	LV TWAS Reports	RA eQTL Reports	LA eQTL Reports	LV eQTL Reports	RVAS Reports	Priority Score
SEC31A	0	0	0	1	0	0	0	0	0	1
SNAPIN	0	0	0	1	0	0	0	0	0	1
TFDP1	0	0	0	1	0	0	0	0	0	1
UBE2L3	0	0	0	1	0	0	0	0	0	1
USP36	0	0	0	1	0	0	0	0	0	1
YDJC	0	0	0	1	0	0	0	0	0	1
ZBTB38	0	0	0	1	0	0	0	0	0	1
ALG1L13P	0	0	0	1	0	0	0	0	0	1
MIR22HG	0	0	0	1	0	0	0	0	0	1
SPTBN1	0	0	0	1	0	0	0	0	0	1
MALAT1	0	0	0	1	0	0	0	0	0	1
UBE3B	0	0	0	1	0	0	0	0	0	1
DNM1P51	0	0	0	1	0	0	0	0	0	1
ATP5F1D	0	0	0	1	0	0	0	0	0	1
VPREB3	0	0	0	1	0	0	0	0	0	1
XIRP1	0	0	0	1	0	0	0	0	0	1
KPNA3	0	0	0	1	0	0	0	0	0	1
CMTM5	0	0	0	2	0	0	0	0	0	1
PHACTR1	0	0	0	1	0	0	0	0	0	1
HMGA1P5	0	0	0	1	0	0	0	0	0	1
GBF1	0	0	0	1	0	0	0	0	0	1
NUDT13	0	0	0	1	0	0	0	0	0	1
SNX1	0	0	0	1	0	0	0	0	0	1
CFDP1	0	0	0	1	0	0	0	0	0	1
LRRC75B	0	0	0	1	0	0	0	0	0	1
UBE2J2	0	0	0	1	0	0	0	0	0	1
CEPT1	0	0	0	1	0	0	0	0	0	1
HIP1	0	0	0	1	0	0	0	0	0	1
RP11-29H23.5	0	0	0	0	1	0	0	0	0	1
RP11-344N10.5	0	0	0	0	1	0	0	0	0	1
RP11-379F4.7	0	0	0	0	1	0	0	0	0	1
RP11-397E7.4	0	0	0	0	1	0	0	0	0	1

Target	Discovery AF Reports	Discovery PR Reports	Discovery LA Reports	RA TWAS Reports	LV TWAS Reports	RA eQTL Reports	LA eQTL Reports	LV eQTL Reports	RVAS Reports	Priority Score
RP4-764O22.2	0	0	0	0	1	0	0	0	0	1
BLOC1S1	0	0	0	0	1	0	0	0	0	1
BRICD5	0	0	0	0	1	0	0	0	0	1
CPEB4	0	0	0	0	1	0	0	0	0	1
DUSP13	0	0	0	0	1	0	0	0	0	1
FTCDNL1	0	0	0	0	1	0	0	0	0	1
GPR155	0	0	0	0	1	0	0	0	0	1
HLTF	0	0	0	0	1	0	0	0	0	1
IKZF3	0	0	0	0	1	0	0	0	0	1
MIR193BHG	0	0	0	0	1	0	0	0	0	1
MYPN	0	0	0	0	1	0	0	0	0	1
CIAO3	0	0	0	0	1	0	0	0	0	1
NPTXR	0	0	0	0	1	0	0	0	0	1
PSMD3	0	0	0	0	1	0	0	0	0	1
REEP3	0	0	0	0	1	0	0	0	0	1
RIOK1	0	0	0	0	1	0	0	0	0	1
SCMH1	0	0	0	0	1	0	0	0	0	1
SEMA3C	0	0	0	0	1	0	0	0	0	1
SLC16A12	0	0	0	0	1	0	0	0	0	1
TBL3	0	0	0	0	1	0	0	0	0	1
TCHP	0	0	0	0	2	0	0	0	0	1
TES	0	0	0	0	2	0	0	0	0	1
TNNT3	0	0	0	0	1	0	0	0	0	1
USP28	0	0	0	0	1	0	0	0	0	1
VPS37B	0	0	0	0	1	0	0	0	0	1
FDFT1	0	0	0	0	1	0	0	0	0	1
ACP6	0	0	0	0	1	0	0	0	0	1
SLC2A11	0	0	0	0	1	0	0	0	0	1
MSTO2P	0	0	0	0	1	0	0	0	0	1
TFEC	0	0	0	0	1	0	0	0	0	1
ZHX1	0	0	0	0	1	0	0	0	0	1
CFAP70	0	0	0	0	1	0	0	0	0	1

Target	Discovery AF Reports	Discovery PR Reports	Discovery LA Reports	RA TWAS Reports	LV TWAS Reports	RA eQTL Reports	LA eQTL Reports	LV eQTL Reports	RVAS Reports	Priority Score
PLCD1	0	0	0	0	1	0	0	0	0	1
IFRD2	0	0	0	0	1	0	0	0	0	1
NPIPA1	0	0	0	0	1	0	0	0	0	1
MYO15A	0	0	0	0	1	0	0	0	0	1
ERCC6L2	0	0	0	0	1	0	0	0	0	1
ARL3	0	0	0	0	1	0	0	0	0	1
PXN	0	0	0	0	1	0	0	0	0	1
MEIOSIN	0	0	0	0	0	1	0	0	0	1
AF001548.5	0	0	0	0	0	1	0	0	0	1
RP11-379H18.1	0	0	0	0	0	1	0	0	0	1
RP11-532N4.2	0	0	0	0	0	1	0	0	0	1
RP11-95P2.1	0	0	0	0	0	1	0	0	0	1
ZNF589	0	0	0	0	0	1	0	0	0	1
DALRD3	0	0	0	0	0	1	0	0	0	1
AMT	0	0	0	0	0	1	0	0	0	1
RBM6	0	0	0	0	0	1	0	0	0	1
NDUFAF3	0	0	0	0	0	1	0	0	0	1
GMPPB	0	0	0	0	0	1	0	0	0	1
NICN1	0	0	0	0	0	1	0	0	0	1
LINC01411	0	0	0	0	0	1	0	0	0	1
LINC00933	0	0	0	0	0	1	0	0	0	1
GOLGA6L4	0	0	0	0	0	1	0	0	0	1
DRICH1	0	0	0	0	0	1	0	0	0	1
L3MBTL2	0	0	0	0	0	1	0	0	0	1
SLC35E1P1	0	0	0	0	0	1	0	0	0	1
AC016747.3	0	0	0	0	0	1	0	0	0	1
RP11-2E17.1	0	0	0	0	0	1	0	0	0	1
MIR1307	0	0	0	0	0	0	2	0	0	1
KIFAP3	0	0	0	0	0	0	2	0	0	1
USMG5	0	0	0	0	0	0	1	0	0	1
LINC00881	0	0	0	0	0	0	1	0	0	1
LINC-PINT	0	0	0	0	0	0	1	0	0	1

Target	Discovery AF Reports	Discovery PR Reports	Discovery LA Reports	RA TWAS Reports	LV TWAS Reports	RA eQTL Reports	LA eQTL Reports	LV eQTL Reports	RVAS Reports	Priority Score
BCAT1	0	0	0	0	0	0	1	0	0	1
IBA57	0	0	0	0	0	0	1	0	0	1
LINC00467	0	0	0	0	0	0	1	0	0	1
TMEM116	0	0	0	0	0	0	1	0	0	1
RP11-182J1	0	0	0	0	0	0	1	0	0	1
EFNA3	0	0	0	0	0	0	1	0	0	1
FLAD1	0	0	0	0	0	0	1	0	0	1
PYGO2	0	0	0	0	0	0	1	0	0	1
EFNA4	0	0	0	0	0	0	1	0	0	1
DCST1-AS1	0	0	0	0	0	0	1	0	0	1
SHC1	0	0	0	0	0	0	1	0	0	1
DCST1	0	0	0	0	0	0	1	0	0	1
RP11-297H3.3	0	0	0	0	0	0	1	0	0	1
SIGLEC30P	0	0	0	0	0	0	1	0	0	1
RP11-576I22.2	0	0	0	0	0	0	1	0	0	1
LINC01142	0	0	0	0	0	0	1	0	0	1
AL356475.1	0	0	0	0	0	0	1	0	0	1
RP11-332H17.1	0	0	0	0	0	0	1	0	0	1
HAUS4P1	0	0	0	0	0	0	1	0	0	1
RP1-79C4.1	0	0	0	0	0	0	1	0	0	1
LENEP	0	0	0	0	0	0	1	0	0	1
ASPRV1	0	0	0	0	0	0	1	0	0	1
PCBP1-AS1	0	0	0	0	0	0	1	0	0	1
AC092431.3	0	0	0	0	0	0	1	0	0	1
AC019206.1	0	0	0	0	0	0	1	0	0	1
RP11-77O7.1	0	0	0	0	0	0	1	0	0	1
AAK1	0	0	0	0	0	0	1	0	0	1
AC092431.2	0	0	0	0	0	0	1	0	0	1
RN7SL211P	0	0	0	0	0	0	1	0	0	1
RNU6-548P	0	0	0	0	0	0	1	0	0	1
ACTR2	0	0	0	0	0	0	1	0	0	1
AC007386.3	0	0	0	0	0	0	1	0	0	1

Target	Discovery AF Reports	Discovery PR Reports	Discovery LA Reports	RA TWAS Reports	LV TWAS Reports	RA eQTL Reports	LA eQTL Reports	LV eQTL Reports	RVAS Reports	Priority Score
LINC02245	0	0	0	0	0	0	1	0	0	1
SLC1A4	0	0	0	0	0	0	1	0	0	1
AC007386.4	0	0	0	0	0	0	1	0	0	1
AC007318.5	0	0	0	0	0	0	1	0	0	1
AC007880.1	0	0	0	0	0	0	1	0	0	1
OSBPL6	0	0	0	0	0	0	1	0	0	1
PJVK	0	0	0	0	0	0	1	0	0	1
AC009948.5	0	0	0	0	0	0	1	0	0	1
TTN-AS1	0	0	0	0	0	0	1	0	0	1
AC009948.7	0	0	0	0	0	0	1	0	0	1
RP11-65L3.1	0	0	0	0	0	0	1	0	0	1
RP11-88L24.4	0	0	0	0	0	0	1	0	0	1
RP11-171I2.1	0	0	0	0	0	0	1	0	0	1
KRT18P17	0	0	0	0	0	0	1	0	0	1
RP11-767C1.1	0	0	0	0	0	0	1	0	0	1
IQSEC1	0	0	0	0	0	0	1	0	0	1
snoU13	0	0	0	0	0	0	0	1	0	1
RP11-777N19.1	0	0	0	0	0	0	1	0	0	1
LYPLA1P2	0	0	0	0	0	0	1	0	0	1
RP11-380D23.2	0	0	0	0	0	0	1	0	0	1
RN7SKP89	0	0	0	0	0	0	1	0	0	1
RP11-492A10.1	0	0	0	0	0	0	1	0	0	1
CTC-313D10.1	0	0	0	0	0	0	1	0	0	1
RP11-325L7.1	0	0	0	0	0	0	1	0	0	1
RP11-325L7.2	0	0	0	0	0	0	1	0	0	1
CDC25C	0	0	0	0	0	0	1	0	0	1
MYOT	0	0	0	0	0	0	1	0	0	1
CDC23	0	0	0	0	0	0	1	0	0	1
KIF20A	0	0	0	0	0	0	1	0	0	1
RP11-166J22.1	0	0	0	0	0	0	1	0	0	1
RN7SL682P	0	0	0	0	0	0	1	0	0	1
RP11-381K20.2	0	0	0	0	0	0	1	0	0	1

Target	Discovery AF Reports	Discovery PR Reports	Discovery LA Reports	RA TWAS Reports	LV TWAS Reports	RA eQTL Reports	LA eQTL Reports	LV eQTL Reports	RVAS Reports	Priority Score
PKD2L2	0	0	0	0	0	0	1	0	0	1
BRD8	0	0	0	0	0	0	1	0	0	1
GFRA3	0	0	0	0	0	0	1	0	0	1
FAM53C	0	0	0	0	0	0	1	0	0	1
RP11-57K17.1	0	0	0	0	0	0	1	0	0	1
RP11-632C17__A.1	0	0	0	0	0	0	1	0	0	1
TBC1D32	0	0	0	0	0	0	1	0	0	1
TRDN-AS1	0	0	0	0	0	0	1	0	0	1
AC073130.3	0	0	0	0	0	0	1	0	0	1
AC002066.1	0	0	0	0	0	0	1	0	0	1
AC006159.3	0	0	0	0	0	0	1	0	0	1
AC006159.5	0	0	0	0	0	0	1	0	0	1
snoZ185	0	0	0	0	0	0	1	0	0	1
AC006159.4	0	0	0	0	0	0	1	0	0	1
MET	0	0	0	0	0	0	1	0	0	1
FGL1	0	0	0	0	0	0	1	0	0	1
RP11-156K13.1	0	0	0	0	0	0	1	0	0	1
CTD-2547L16.3	0	0	0	0	0	0	1	0	0	1
CTD-2547L16.1	0	0	0	0	0	0	1	0	0	1
NAT1	0	0	0	0	0	0	1	0	0	1
RP11-156K13.3	0	0	0	0	0	0	1	0	0	1
RP11-156K13.2	0	0	0	0	0	0	1	0	0	1
RP11-49O14.3	0	0	0	0	0	0	1	0	0	1
RP11-54O15.3	0	0	0	0	0	0	1	0	0	1
RP11-80I15.1	0	0	0	0	0	0	1	0	0	1
RP11-80I15.4	0	0	0	0	0	0	1	0	0	1
MIR27B	0	0	0	0	0	0	1	0	0	1
RP11-49O14.2	0	0	0	0	0	0	1	0	0	1
GLUD1P3	0	0	0	0	0	0	1	0	0	1
C10orf55	0	0	0	0	0	0	1	0	0	1
PPP3CB	0	0	0	0	0	0	1	0	0	1

Target	Discovery AF Reports	Discovery PR Reports	Discovery LA Reports	RA TWAS Reports	LV TWAS Reports	RA eQTL Reports	LA eQTL Reports	LV eQTL Reports	RVAS Reports	Priority Score
AC022400.2	0	0	0	0	0	0	1	0	0	1
CHCHD1	0	0	0	0	0	0	1	0	0	1
RP11-464F9.1	0	0	0	0	0	0	1	0	0	1
RP11-464F9.20	0	0	0	0	0	0	1	0	0	1
BMS1P4	0	0	0	0	0	0	1	0	0	1
MSS51	0	0	0	0	0	0	1	0	0	1
CAMK2G	0	0	0	0	0	0	1	0	0	1
AC022400.1	0	0	0	0	0	0	1	0	0	1
RP11-574K11.5	0	0	0	0	0	0	1	0	0	1
RP11-137L10.5	0	0	0	0	0	0	1	0	0	1
RP11-345K20.2	0	0	0	0	0	0	1	0	0	1
ANXA7	0	0	0	0	0	0	1	0	0	1
RP11-574K11.8	0	0	0	0	0	0	1	0	0	1
ATP5MK	0	0	0	0	0	0	1	0	0	1
CALHM3	0	0	0	0	0	0	1	0	0	1
PDCD11	0	0	0	0	0	0	1	0	0	1
TAF5	0	0	0	0	0	0	1	0	0	1
CALHM1	0	0	0	0	0	0	1	0	0	1
RP11-225H22.5	0	0	0	0	0	0	1	0	0	1
PCGF6	0	0	0	0	0	0	1	0	0	1
RNU11-3P	0	0	0	0	0	0	1	0	0	1
RP11-225H22.4	0	0	0	0	0	0	1	0	0	1
INA	0	0	0	0	0	0	1	0	0	1
RP11-541N10.3	0	0	0	0	0	0	1	0	0	1
RP11-744N12.3	0	0	0	0	0	0	1	0	0	1
SENCR	0	0	0	0	0	0	1	0	0	1
RP11-740D6.3	0	0	0	0	0	0	1	0	0	1
TP53AIP1	0	0	0	0	0	0	1	0	0	1
ARHGAP32	0	0	0	0	0	0	1	0	0	1
FLI1	0	0	0	0	0	0	1	0	0	1
KCNJ1	0	0	0	0	0	0	1	0	0	1
TBX5-AS1	0	0	0	0	0	0	1	0	0	1

Target	Discovery AF Reports	Discovery PR Reports	Discovery LA Reports	RA TWAS Reports	LV TWAS Reports	RA eQTL Reports	LA eQTL Reports	LV eQTL Reports	RVAS Reports	Priority Score
RP11-139B1.1	0	0	0	0	0	0	1	0	0	1
OSTF1P1	0	0	0	0	0	0	1	0	0	1
ZBTB25	0	0	0	0	0	0	1	0	0	1
CTD-2555O16.1	0	0	0	0	0	0	1	0	0	1
TEX21P	0	0	0	0	0	0	1	0	0	1
RP11-544I20.2	0	0	0	0	0	0	1	0	0	1
CTD-2555O16.2	0	0	0	0	0	0	1	0	0	1
RP11-272D12.2	0	0	0	0	0	0	1	0	0	1
REC114	0	0	0	0	0	0	1	0	0	1
NPTN	0	0	0	0	0	0	1	0	0	1
NPM1P43	0	0	0	0	0	0	1	0	0	1
NEO1	0	0	0	0	0	0	1	0	0	1
RP11-272D12.1	0	0	0	0	0	0	1	0	0	1
FKBP1AP2	0	0	0	0	0	0	1	0	0	1
MRPS15P1	0	0	0	0	0	0	1	0	0	1
RP5-991G20.1	0	0	0	0	0	0	1	0	0	1
ZFHX3	0	0	0	0	0	0	1	0	0	1
RP5-991G20.2	0	0	0	0	0	0	1	0	0	1
RP5-991G20.4	0	0	0	0	0	0	1	0	0	1
AC140912.1	0	0	0	0	0	0	1	0	0	1
HCCAT5	0	0	0	0	0	0	1	0	0	1
RP11-69E11.4	0	0	0	0	0	0	0	1	0	1
RP1-1J6.2	0	0	0	0	0	0	0	1	0	1
RP11-182J1.14	0	0	0	0	0	0	0	1	0	1
RP11-69E11.4	0	0	0	0	0	0	0	1	0	1
RP11-72I8.1	0	0	0	0	0	0	0	1	0	1
RP11-795J1.1	0	0	0	0	0	0	0	1	0	1
CHD1L	0	0	0	0	0	0	0	1	0	1
TMA7	0	0	0	0	0	0	0	1	0	1
LXN	0	0	0	0	0	0	0	1	0	1
MIR490	0	0	0	0	0	0	0	1	0	1
MMAB	0	0	0	0	0	0	0	1	0	1

Target	Discovery AF Reports	Discovery PR Reports	Discovery LA Reports	RA TWAS Reports	LV TWAS Reports	RA eQTL Reports	LA eQTL Reports	LV eQTL Reports	RVAS Reports	Priority Score
NDRG4	0	0	0	0	0	0	0	1	0	1
MAP3K3	0	0	0	0	0	0	0	1	0	1
DUSP29	0	0	0	0	0	0	0	1	0	1
IFT88	0	0	0	0	0	0	0	1	0	1
AC007620.3	0	0	0	0	0	0	0	1	0	1
RP11-259G18.2	0	0	0	0	0	0	0	1	0	1
RP11-259G18.3	0	0	0	0	0	0	0	1	0	1
RP11-380L11.4	0	0	0	0	0	0	0	1	0	1
RP11-707O23.5	0	0	0	0	0	0	0	1	0	1
PMS2P2	0	0	0	0	0	0	0	1	0	1
ARL17A	0	0	0	0	0	0	0	1	0	1
LINC02210	0	0	0	0	0	0	0	1	0	1
KANSL1-AS1	0	0	0	0	0	0	0	1	0	1
NSFP1	0	0	0	0	0	0	0	1	0	1
HAND2-AS1	0	0	0	0	0	0	0	1	0	1
MUC5B	0	0	0	0	0	0	0	0	1	1
TTC21A	0	0	0	0	0	0	0	0	1	1
TRIML2	0	0	0	0	0	0	0	0	1	1
SLC22A11	0	0	0	0	0	0	0	0	1	1
MTRF1	0	0	0	0	0	0	0	0	1	1
CD36	0	0	0	0	0	0	0	0	1	1
CAPRIN2	0	0	0	0	0	0	0	0	1	1
PIK3R6	0	0	0	0	0	0	0	0	1	1

Legend: AF - Atrial Fibrillation; LA - Left Atrium; RA - Right Atrium; LV - Left Ventricle; TWAS - Transcriptome Wide Association Study; eQTL - expression Quantitative Trait Loci colocalisation; RVAS - Rare Variant Association Study; Green shading indicates at least one report.

Supplementary Table 9 - Prioritised targets with evidence of druggability for small molecules

Target	Gene Biotype	Druggable Family	High-Quality Pocket	Med-Quality Pocket	Small Molecule Druggable Score	Priority Score
CASQ2	Protein Coding	FALSE	FALSE	TRUE	1	7
KDM1B	Protein Coding	TRUE	FALSE	FALSE	1	5
TPMT	Protein Coding	TRUE	TRUE	FALSE	2	4
FKBP7	Protein Coding	TRUE	FALSE	FALSE	1	4
PLEC	Protein Coding	FALSE	FALSE	TRUE	1	4
PLAU	Protein Coding	TRUE	TRUE	FALSE	2	3
CAMK2D	Protein Coding	TRUE	TRUE	FALSE	2	3
MTHFD1	Protein Coding	FALSE	TRUE	FALSE	1	3
BMPR1A	Protein Coding	TRUE	FALSE	FALSE	1	3
CDKN1A	Protein Coding	TRUE	FALSE	FALSE	1	3
TTN	Protein Coding	TRUE	FALSE	FALSE	1	3
HCN1	Protein Coding	TRUE	FALSE	FALSE	1	3
SLK	Protein Coding	TRUE	FALSE	TRUE	2	2
PROZ	Protein Coding	TRUE	FALSE	TRUE	2	2
PNMT	Protein Coding	TRUE	TRUE	FALSE	2	2
FABP2	Protein Coding	TRUE	FALSE	TRUE	2	2
TGM2	Protein Coding	TRUE	TRUE	FALSE	2	2
KCNN2	Protein Coding	TRUE	FALSE	FALSE	1	2
MMP11	Protein Coding	TRUE	FALSE	FALSE	1	2
SETD6	Protein Coding	FALSE	FALSE	TRUE	1	2
WNT3	Protein Coding	TRUE	FALSE	FALSE	1	2
ACVR2A	Protein Coding	TRUE	FALSE	FALSE	1	2
SYMPK	Protein Coding	FALSE	FALSE	TRUE	1	2
SLC6A6	Protein Coding	TRUE	FALSE	FALSE	1	2
PIK3C2B	Protein Coding	TRUE	FALSE	FALSE	1	2
TBX3	Protein Coding	FALSE	FALSE	TRUE	1	2
KCNN3	Protein Coding	TRUE	FALSE	FALSE	1	2
TYW5	Protein Coding	FALSE	TRUE	FALSE	1	2
ADCY5	Protein Coding	TRUE	FALSE	FALSE	1	2
KLHL38	Protein Coding	TRUE	FALSE	FALSE	1	2
SPON1	Protein Coding	FALSE	TRUE	FALSE	1	2

Supplementary Table 10 - Prioritised targets with evidence of druggability for antibody drugs

Target	Gene Biotype	GO High Confidence	Human Protein Atlas High Confience	UniProt High Confidence	Antibody Druggable Score	Priority Score
FAT1	Protein Coding	TRUE	FALSE	TRUE	2	6
SYNE2	Protein Coding	FALSE	FALSE	TRUE	1	6
MTSS1	Protein Coding	FALSE	TRUE	FALSE	1	6
CDH13	Protein Coding	TRUE	TRUE	FALSE	2	5
GNB4	Protein Coding	FALSE	TRUE	FALSE	1	5
WIPF1	Protein Coding	FALSE	TRUE	FALSE	1	5
EDN2	Protein Coding	TRUE	FALSE	FALSE	1	5
ASAH1	Protein Coding	TRUE	FALSE	TRUE	2	4
GYPC	Protein Coding	TRUE	TRUE	FALSE	2	4
GJA1	Protein Coding	TRUE	FALSE	TRUE	2	4
CAV1	Protein Coding	TRUE	FALSE	FALSE	1	4
NEURL1	Protein Coding	TRUE	FALSE	FALSE	1	4
KCNJ5-AS1	Long Non-Coding RNA	FALSE	FALSE	TRUE	1	4
PLEC	Protein Coding	TRUE	FALSE	FALSE	1	4
PHLDB2	Protein Coding	TRUE	TRUE	TRUE	3	3
DAG1	Protein Coding	TRUE	TRUE	TRUE	3	3
PLAU	Protein Coding	TRUE	FALSE	TRUE	2	3
FBN2	Protein Coding	TRUE	FALSE	TRUE	2	3
CAMK2D	Protein Coding	FALSE	TRUE	TRUE	2	3
HCN1	Protein Coding	TRUE	FALSE	TRUE	2	3
SPATA20	Protein Coding	FALSE	FALSE	TRUE	1	3
CCDC134	Protein Coding	FALSE	FALSE	TRUE	1	3
CAV2	Protein Coding	TRUE	FALSE	FALSE	1	3
BMPR1A	Protein Coding	TRUE	FALSE	FALSE	1	3
UBE2D3	Protein Coding	FALSE	FALSE	TRUE	1	3
PFDN1	Protein Coding	FALSE	TRUE	FALSE	1	3
PLEKHA3	Protein Coding	FALSE	FALSE	TRUE	1	3
SSPN	Protein Coding	TRUE	FALSE	FALSE	1	3
ABHD17C	Protein Coding	TRUE	FALSE	FALSE	1	3
SYPL2	Protein Coding	FALSE	TRUE	FALSE	1	3
ADAM15	Protein Coding	TRUE	FALSE	FALSE	1	3

Target	Gene Biotype	GO High Confidence	Human Protein Atlas High Confience	UniProt High Confidence	Antibody Druggable Score	Priority Score
TTN	Protein Coding	TRUE	FALSE	FALSE	1	3
CFL2	Protein Coding	FALSE	TRUE	FALSE	1	3
STIM1	Protein Coding	TRUE	FALSE	FALSE	1	3
EMB	Protein Coding	TRUE	FALSE	FALSE	1	3
BMP8A	Protein Coding	FALSE	FALSE	TRUE	1	3
QRICH1	Protein Coding	FALSE	FALSE	TRUE	1	3
ZSWIM8	Protein Coding	FALSE	TRUE	FALSE	1	3
JAM2	Protein Coding	TRUE	TRUE	TRUE	3	2
CCBE1	Protein Coding	TRUE	TRUE	TRUE	3	2
TRDN	Protein Coding	TRUE	TRUE	TRUE	3	2
TGM2	Protein Coding	TRUE	TRUE	TRUE	3	2
TAB2	Protein Coding	TRUE	FALSE	TRUE	2	2
WNT8A	Protein Coding	TRUE	FALSE	TRUE	2	2
TESC	Protein Coding	TRUE	FALSE	TRUE	2	2
MMP11	Protein Coding	TRUE	FALSE	TRUE	2	2
WNT3	Protein Coding	TRUE	FALSE	TRUE	2	2
SLC2A9	Protein Coding	TRUE	FALSE	TRUE	2	2
SLC27A6	Protein Coding	TRUE	FALSE	TRUE	2	2
SYMPK	Protein Coding	TRUE	TRUE	FALSE	2	2
SLC6A6	Protein Coding	TRUE	FALSE	TRUE	2	2
PIK3C2B	Protein Coding	TRUE	FALSE	TRUE	2	2
CGA	Protein Coding	TRUE	FALSE	TRUE	2	2
FGF5	Protein Coding	TRUE	FALSE	TRUE	2	2
HSPG2	Protein Coding	TRUE	TRUE	FALSE	2	2
LRIG1	Protein Coding	TRUE	FALSE	TRUE	2	2
EFNA1	Protein Coding	TRUE	FALSE	TRUE	2	2
ADCY5	Protein Coding	TRUE	FALSE	TRUE	2	2
NMB	Protein Coding	TRUE	TRUE	FALSE	2	2
SLK	Protein Coding	FALSE	TRUE	FALSE	1	2
FERMT2	Protein Coding	TRUE	FALSE	FALSE	1	2
KCNN2	Protein Coding	TRUE	FALSE	FALSE	1	2

Target	Gene Biotype	GO High Confidence	Human Protein Atlas High Confience	UniProt High Confidence	Antibody Druggable Score	Priority Score
SLCO4A1	Protein Coding	TRUE	FALSE	FALSE	1	2
GORASP1	Protein Coding	FALSE	FALSE	TRUE	1	2
RAB29	Protein Coding	FALSE	FALSE	TRUE	1	2
KCNIP2	Protein Coding	TRUE	FALSE	FALSE	1	2
ACVR2A	Protein Coding	TRUE	FALSE	FALSE	1	2
KCNN3	Protein Coding	TRUE	FALSE	FALSE	1	2
FABP2	Protein Coding	FALSE	TRUE	FALSE	1	2
FREM2	Protein Coding	FALSE	FALSE	TRUE	1	2
PGAP3	Protein Coding	FALSE	TRUE	FALSE	1	2
PM20D1	Protein Coding	TRUE	FALSE	FALSE	1	2
SENP2	Protein Coding	FALSE	FALSE	TRUE	1	2
SLC35A1	Protein Coding	TRUE	FALSE	FALSE	1	2
IL25	Protein Coding	TRUE	FALSE	FALSE	1	2
VANGL1	Protein Coding	FALSE	TRUE	FALSE	1	2
SEC24C	Protein Coding	FALSE	FALSE	TRUE	1	2
WDR6	Protein Coding	TRUE	FALSE	FALSE	1	2
DMWD	Protein Coding	FALSE	TRUE	FALSE	1	2
ANXA4	Protein Coding	TRUE	FALSE	FALSE	1	2
NCKIPSD	Protein Coding	FALSE	TRUE	FALSE	1	2
C4A	Protein Coding	TRUE	FALSE	FALSE	1	2
GJA5	Protein Coding	TRUE	FALSE	FALSE	1	2

Legend: GO High Confidence - High confidence that subcellular location is plasma membrane, extracellular region/matrix, or secretion from gene ontology database; UniProt High Confidence - High confidence that the subcellular location is either plasma membrane, extracellular region/matrix, or secretion from UniProt database; Human Protein Atlas High Confidence - High confidence that subcellular location is plasma membrane from human protein atlas.

Supplementary Table 11 – Drugs associated with targets and GWAS catalog information on other cardiovascular traits.

Symbol	Drugs	GWAS Information Regarding Other CV Traits
ACVR2B	Bimagrumab	None
ADRA1A	Alfuzosin, Alseroxylon, Aviptadil, Bucindolol, Bunazosin, Carvedilol, Dabuzalgron, Dapiprazole, Dipivefrin, Doxazosin, Droxidopa, Ephedrine, Epinephrine, Ergoloid, Ergotamine, Hydroxyamphetamine, Isoxsuprine, Labetalol, Mephentermine, Metaraminol, Methoxamine, Midodrine, Moxisylyte, Naphazoline, Norepinephrine, Oxymetazoline, Phenoxybenzamine, Phentolamine, Phenylephrine, Phenylpropanolamine, Prazosin, Rauwolfia Serpentina, Sertindole, Silodosin, Tamsulosin, Tedatioxetine, Terazosin, Tetrahydrozoline, Tolazoline, Trimipramine	None
ADRB1	Acebutolol, Atenolol, Betaxolol, Bisoprolol, Bucindolol, Carteolol, Carvedilol, Celiprolol, Dilevalol, Dipivefrin, Dobutamine, Dopamine, Droxidopa, Ephedrine, Epinephrine, Esmolol, Hydroxyamphetamine, Isoproterenol, Labetalol, Landiolol, Levobetaxolol, Levobunolol, LY377604, Mephentermine, Metipranolol, Metoprolol, Nadolol, Nebivolol, Norepinephrine, Oxprenolol, Penbutolol, Pindolol, Practolol, Pronetalol, Propafenone, Propranolol, Sotalol, Timolol, Xamoterol	BP, CVD, DBP, DBP interaction with cigarette smoking, DBP interaction with alcohol consumption, Hypertension, MAP, MAP interaction with alcohol consumption, PAC frequency, SBP, SBP interaction with cigarette smoking, SBP interaction with alchohol consumption,
AOPEP (C9orf3)	Tosedostat	JT interval, QT interval, QRS duration, Cardioembolic Stroke, Cardiac Arrhythmia
BLK	Dasatinib, Ilorasertib, XL-228, TG100-801, ENMD-981693	None
CA4	Dichlorphenamide, Sulthiame, Ethoxzolamide, Acetazolamide, Methazolamide, Topiramate	None
CACNA1G	ACT-709478, Atagabalin, Bepridil, Gabapentin, Gabapentin Enacarbil, Imagabalin, Methsuximide, Mibefradil, Paramethadione, Phensuximide, Pregabalin, Suloctidil, Suvecaltamide, Terodiline, Trimethadione	None
CDC7	BMS-863233, NMS-1116354, RXDX-103, Simurosertib	None
CHRM2	Afacifenacin, Alcuronium, ASM-024, Atropine, Batefenterol, Bethanechol, Darifenacin, Darotropium, Fesoterodine, Oxybutynin, Propantheline, Sofpironium, Solifenacin, Talsaclidine, Terodiline, Tolterodine, Trospium	HR, HR response to exercise, HR response to recovery from exercise, PP, Resting HR
ENPEP	Firibastat, Tosedostat	SBP, DBP, MAP, Hypertension, PP, CVD, Ischaemic stroke in dyslipidaemia
EPAS1	Belzutifan	JT interval, QRS duration
EPHA3	KB-004, Vandetanib	DBP change trajectory

Symbol	Drugs	GWAS Information Regarding Other CV Traits
ERBB2	AC-480, AEE-788, Afatinib, Allitinib, BDTX189, BMS-690514, Canertinib, CP-724714, CUDC-101, Dacomitinib, Ertumaxomab, Gancotamab, HEMAY-022, INSM-18, JNJ-26483327, KBP5209, Lapatinib, Margetuximab, MDX-210, MM-111, MP-412, Mubritinib, Neratinib, Pertuzumab, Poziotinib, Pyrotinib, S-222611, Sapitinib, T-DM1, TAK-285, Tarloxotinib, Tesevatinib, Trastuzumab, Trastuzumab Deruxtecan, Trastuzumab Duocarmazine, Trastuzumab Emtansine, Trastuzumab Emtansine, Tucatinib, Vandetanib, Varlitinib, Zenocutuzumab	Asthma and CVD
ERBB4	AC-480, Afatinib, BMS-690514, Canertinib, Dacomitinib, JNJ-26483327, KBP5209, Neratinib, Poziotinib, Tarloxotinib, Vandetanib	CAD, QT interval interaction with ambient particulate matter
ESR2	Acolbifene, Afimoxifene, Allylestrenol, Arzoxifene, AUS-131, Bazedoxifene, CHF4227, Chlorotrianisene, CR 1447, Cyclofenil, Diethylstilbestrol Diphosphate, Droloxifene, Erteberel, Estetrol, Estramustine Phosphate, Estriol, Estrogens, Conjugated, Estrogens, Conjugated Synthetic A, Estrogens, Esterified, Fispemifene, Fulvestrant, Idoxifene, Lasofoxifene, LY2245461, MF101, MK-6913, Ospemifene, Prinaberel, Quinestrol, Raloxifene, Synthetic Conjugated Estrogens, B, Toremifene	None
FDFT1	Lapaquistat Acetate	None
FGFR1	AZD-4547, Brivanib, CP-459632, Derazantinib, E-7090, ENMD-981693, Erdafitinib, FGFR Inhibitor Debio 1347, Futibatinib, HMPL-453, Infigratinib, Lucitanib, LY-2874455, Nintedanib, Orantinib, Pazopanib, PD-0166285, Pemigatinib, Regorafenib, RG-1530, Rogaratinib, Surufatinib, TG100-801, XL-999	SBP
FGFR2	Aprutumab Ixadotin, AZD-4547, Brivanib, CP-459632, Derazantinib, E-7090, ENMD-981693, Erdafitinib, FGFR Inhibitor Debio 1347, FPA144, Futibatinib, HMPL-453, Infigratinib, Lucitanib, LY-2874455, Nintedanib, Orantinib, Palifermin, Pemigatinib, Regorafenib, RG-1530, Rogaratinib, TG100-801, Trafermin, XL-999	None
HBEGF	KHK-2866	PP, SBP
HCN4	Dronedarone, Ivabradine	BP (Pleiotropy model 1 and 2), Cardioembolic Stroke, CAD in dyslipidaemia, HR variability traits (pvRSA/HF, RMSSD, SDNN), Resting HR
IGF1R	AEW-541, AVE-1642, AXL-1717, BIIB-022, BMS-754807, Cixutumumab, Conteltinib , Dalotuzumab, Figitumumab, Ganitumab, INSM-18, Istiratumab, KW-2450, Linsitinib, Mecasermin, PL-225B, Robatumumab, Teprotumumab, XL-228	JT interval, Left ventricular wall thickness, Left ventricular mass, Left ventricular mass to end-diastolic volume ratio, TPE interval, QRS duration
IL6R	Levilimab, Sarilumab, Satralizumab, Tocilizumab, Vobarilizumab	Abdominal aortic aneurysm, Angina pectoris, CAD, Arrhythmia, DBP, MI, Hypertension
IMPDH1	AVN-944, Merimepodib, Mizoribine, Mycophenolate Mofetil, Ribavirin, Thioguanine	Arrhythmia, DCM, HCM
ITGA2B	Tadocizumab, Tirofiban, Abciximab, Eptifibatide	None
ITGB1	Abituzumab, ATN-161, Firategrast, Natalizumab, Volociximab	None
KCND3	Amifampridine, Dalfampridine, Guanidine, Nerispirdine, Tedisamil, Vernakalant	Arrhythmia, Early cardiac repolarization, JT interval. QRS duration
KCNH2	Amifampridine, Amiodarone, AZD1305, AZD7009, Dalfampridine, Dofetilide, Guanidine, Ibutilide, Nerispirdine, Sotalol, Tedisamil, Vernakalant	Cardioembolic Stroke, DBP, SBP, QT interval, QT interval dynamics during exercise, JT interval, QTc interval, T wave morphology restitution during exercise, TPE interval
KCNJ2	Dronedarone	QT interval

Symbol	Drugs	GWAS Information Regarding Other CV Traits
KCNJ5	Vernakalant	BP (Pleiotropy model 1), Cardioembolic Stroke, DBP, Resting HR
LAMB2	Ocriplasmin	CAD, MI, QT interval
MAPT	Gosuranemab, JNJ-63733657, Semorinemab, Tilavonemab, Zagotenemab	Arrhythmia, DBP, QRS complex (12 lead sum), SBP,
MC4R	Bremelanotide, PF-00446687, Setmelanotide	CVD, CAD, Hypertension, PP, PW-ASI, HR
MET	Savolitinib, Telisotuzumab Vedotin, Amuvatinib, Cabozantinib, Ningetinib, Altiratinib, SGX-523, Terevalefim, Tepotinib, Foretinib, Onartuzumab, BMS-698769, SAR-125844, AMG-208, MK-8033, ARRY-300, AMG-337, BMS-794833, Capmatinib, TAS-115, Amivantamab, MK-2461, Merestinib, BMS-777607, JNJ-38877605, Glesatinib, BMS-817378, Beperminogene, Crizotinib, EMD-1204831, Tivantinib, Telisotuzumab, Emibetuzumab, BPI-9016, PF-04217903, Golvatinib	PP, DBP, SBP, HR
MTNR1A	Tasimelteon, Melatonin, Piromelatine, Agomelatine, Ramelteon	None
MYH6	Mavacamten, Danicamtiv, Omecamtiv Mecarbil	BP (pleiotropy model 1 and 2), Cardiac Dysrhythmias (time to diagnosis), Descending thoracic aorta diameter, DBP, SBP, PP, HR, Resting HR, Predicted HR recovery to exercise
MYH7	Mavacamten, Danicamtiv, Omecamtiv Mecarbil	Descending aorta maximum area
MYL4	Mavacamten, Danicamtiv, Omecamtiv Mecarbil	SBP, PP
NDUFAF3	ME-344, Metformin, NV-128	CAD, MI, DBP, Hypertension, PP, HR, SBP
NDUFB10	ME-344, Metformin, NV-128	Arrhythmia, CAD, DBP, Hypertension, PP, PW-ASI
NPR3	Carperitide	Hypertension, MAP, SBP, DBP, CVD, PP, Other Cardiomyopathy
NR3C1	Alclometasone Dipropionate, Amcinonide, AZD-5423, Beclomethasone Dipropionate, Betamethasone, Budesonide, Ciclesonide, Clobetasol Propionate, Clocortolone Pivalate, CORT 108297, Cortisone Acetate, Cortivazol, Cyproterone Acetate, Dagrocorat, Deflazacort, Desonide, Desoximetasone, Dexamethasone, Diflorasone Diacetate, Difluprednate, Flumethasone Pivalate, Flunisolide, Fluocinolone Acetonide, Fluocinonide, Fluorometholone, Flurandrenolide, Fluticasone Furoate, Fosdagrocorat, Halcinonide, Halobetasol Propionate, Hydrocortamate, Hydrocortisone, Loteprednol Etabonate, Mapracorat, Medrysone, Meprednisone, Methylprednisolone, Mifepristone, Mometasone Furoate, ORG-34517, Paramethasone Acetate, Prednicarbate, Prednisolone, Prednisone, Relacorilant, Rimexolone, Triamcinolone	None
PDE3A	Aminophylline, Anagrelide, Cilostazol, Dipyridamole, Dyphylline, Enoximone, Ibudilast, Inamrinone, K-134, Levosimendan, Milrinone, Oxtriphylline, Pentoxifylline, Theophylline	BP (pleiotropy model 1 and 2), Coronary artery calcified plaque in T2DM, DBP, Hypertension, Ischaemic Stroke, MAP, PP, QT interval, Resting HR, Stroke, SBP, SBP interaction with alcohol consumption.

Symbol	Drugs	GWAS Information Regarding Other CV Traits
PDE4B	Aminophylline, Amlexanox, Apremilast, CC-11050, CHF-6001, Cilomilast, Crisaborole, Difamilast, Dipyridamole, Drotaverine, Dyphylline, Flavoxate, GSK-256066, HT-0712, Ibudilast, Lotamilast, MK-0873, Oglemilast, Oxtriphylline, Pentoxifylline, Roflumilast, Tetomilast, Theophylline, Tofimilast	PP, SBP, HR, QT interval
POLR2A	TAS-106	Cardioembolic Stroke
PRKCA	Aprinocarsen, CEP-2563, GSK-690693, Midostaurin, Sotrastaurin, UCN-01	BP (pleiotropy model 2), Brugada syndrome, Cardiorespiratory fitness, Coronary artery calcification, DBP, DCM, HF, High-senstivity cardiac troponin T levels, HCM, JT interval, MAP, QRS complex (leadsum), QRS duration, QT dynamics during exercise, QT dynamics during recovery from exercise, QT interval, QTc interval, SBP
PSMB7	Bortezomib, Carfilzomib, Ixazomib, Marizomib, Oprozomib	High sensitivity troponin level, SBP, PP
PSMD3	Bortezomib, Carfilzomib, Ixazomib, Oprozomib	None
PTK2	GSK-2256098, BI-853520, VS-4718, Defactinib, Conteltinib, CEP-37440, PF-00562271	SBP, DPB
RAF1	XL-281, Sorafenib, MLN-2480, LGX-806, ARQ-736, LY-3009120, LXH254, Regorafenib, RG-7376, Belvarafenib	None
RPL32	ELX-02, Ataluren, MT-3724, Cycloheximide, Dorlimomab Aritox	None
RPS2	Ataluren, Cycloheximide, Dorlimomab Aritox, ELX-02, MT-3724	None
RPSA	Ataluren, Cycloheximide, Dorlimomab Aritox, ELX-02, MT-3724	PW-ASI
SCN10A	Afacifenacin, Articaine, Benoxinate, Carbamazepine, Cenobamate, Chloroprocaine, Cocaine, Dibucaine, Dichlorobenzyl Alcohol, Disopyramide, Dronedarone, DSP-2230, Dyclonine, Encainide, Erlosamide, Eslicarbazepine, Eslicarbazepine Acetate, Ethotoin, Etidocaine, Evenamide, Fosphenytoin, Hexylcaine, Indecainide, Irampanel, Lamotrigine, Lidocaine, Mephenytoin, Mepivacaine, Merethoxylline Procaine, Mexiletine, Moricizine, Nerispirdine, NKTR-171, Orphenadrine, Oxcarbazepine, PF-04531083, Phenacemide, Phenazopyridine, Phenytoin, Prilocaine, Primidone, Procainamide, Procaine, Propafenone, Proparacaine, Propoxycaine, Quinidine, Ralfinamide, Riluzole, Ropivacaine, Rufinamide, Tetracaine, Tocainide, Topiramate, Zonisamide	Atrioventricular conduction, QRS duration, QT interval, TPE, HR response to exercise, HR response to recovery after exercise, T wave morphology restitution during exercise, T wave morphology restitution during recovery after exercise, Sick sinus syndrome, Brugada syndrome, Supraventricular ectopy, Resting HR, PP, VO2 Max
SCN5A	Articaine, AZD1305, AZD7009, Benoxinate, Carbamazepine, Cenobamate, Chloroprocaine, Cocaine, Dibucaine, Dichlorobenzyl Alcohol, Disopyramide, Dronedarone, Dyclonine, Encainide, Erlosamide, Eslicarbazepine, Eslicarbazepine Acetate, Ethotoin, Etidocaine, Evenamide, Flecainide, Fosphenytoin, Hexylcaine, Indecainide, Irampanel, Lamotrigine, Lidocaine, Mephenytoin, Mepivacaine, Merethoxylline Procaine, Mexiletine, Moricizine, Nerispirdine, NKTR-171, Orphenadrine, Oxcarbazepine, Phenacemide, Phenazopyridine, Phenytoin, Prenylamine, Prilocaine, Primidone, Procainamide, Procaine, Propafenone, Proparacaine, Propoxycaine, Quinidine, Ralfinamide, Ranolazine, Riluzole, Ropivacaine, Rufinamide, Tetracaine, Tocainide, Topiramate, Vernakalant, Zonisamide	BP (pleiotropy model 1 and 2), Brugada syndrome, QRS duration, JT interval, QT interval, QT dynamics during exercise, QTc interval, PAC frequency, Supraventricular ectopy, Resting HR, Pulse rate, TPE interval, T wave morphology restitution during recovery after exercise
SIRT1	SRT-2104	None
SLAMF7	Azintuxizumab Vedotin, Elotuzumab	None

Symbol	Drugs	GWAS Information Regarding Other CV Traits
SLC6A4	Dapoxetine, Fluoxetine, Sibutramine, Vortioxetine, Tedatioxetine, Paroxetine, Liafensine, Protriptyline, Nortriptyline, Trazodone, Duloxetine, Nomifensine, Chlorphentermine, GSK163090, Vilazodone, Milnacipran, Sertraline, Fluvoxamine, Methamphetamine, Levomilnacipran, Centanafadine, Mazindol, Amoxapine, Nefazodone, Citalopram, Venlafaxine, Imipramine, Dasotraline, Faxeladol, Amitriptyline, Escitalopram, Tesofensine, NS-2359, Desvenlafaxine, Bicifadine, Lumateperone, Indalpine, Dothiepin, Clomipramine, Amitifadine	None
SMAD7	Mongersen Sodium	CAD, MI
SRD5A3	Abiraterone, Dutasteride	DBP, SBP
SYK	Cerdulatinib, Entospletinib, Fostamatinib, Gusacitinib, HMPL-523, Lanraplenib, Mivavotinib, PRT-2607, R-112, R-333, R-343, R-348, R-406	None
THRB	Dextrothyroxine, Eprotirome, Levothyroxine, Liothyronine, MB07811, Resmetirom	JT interval, QRS duration, QT interval, SBP in sickle cell anaemia
TNFSF12	BIIB-023, RO-5458640	Cardioembolic Stroke, DBP interaction with smoking status, PP, SBP, SBP interaction with smoking status
TNFSF13	Atacicept	Hypertension, CVD, DBP, SBP, PP
TNNI3	Levosimendan	None
TNNT3	Tirasemtiv	MAP, Hypertension, PP, SBP, DBP, PW-ASI, HF and antihypertensive medications, CVD
TUBB3	ABT-751, AGS-16C3F, ANG1005, Aprutumab Ixadotin, ASG-5ME, Azintuxizumab Vedotin, Belantamab Mafodotin, Bivatuzumab Mertansine, BMS-275183, Brentuximab Vedotin, Cabazitaxel, Cantuzumab Mertansine, Colchicine, Crolibulin, Davunetide, Docetaxel, Dolastatin-10, Enapotamab Vedotin, Enfortumab Vedotin, Epothilone D, Eribulin, Glembatumumab Vedotin, Indibulin, Indusatumab Vedotin, Ixabepilone, KOS-1584, Ladiratuzumab Vedotin, Larotaxel, Lexibulin, Lifastuzumab Vedotin, Lorvotuzumab Mertansine, Mirvetuximab Soravtansine, Paclitaxel, Patupilone, PF-06263507, Pinatuzumab Vedotin, Plinabulin, Polatuzumab Vedotin, Praluzatamab Ravtansine, RG-7600, RG-7636, RG-7841, Sagopilone, SAR-408701, Sofituzumab Vedotin, T-900607, Telisotuzumab Vedotin, Tisotumab Vedotin, Trastuzumab Emtansine, Vandortuzumab Vedotin, Verubulin, Vinblastine, Vincristine, Vinflunine, Vinorelbine	DBP, Hypertension, CVD
WT1	Galinpepimut-S, Ombipepimut-S	Brugada syndrome, CVD, CAD, PP, SBP
XPO1	Selinexor	SBP, DBP, QRS duration

Key: GWAS – Target from genome wide association study; ExWAS – Target from exome or rare variant association study; EXP – Target from expression level association data; BP – Blood Pressure; SBP/DBP; Systolic Blood Pressure/Diastolic Blood Pressure; CVD – Cardiovascular Disease; MAP – Mean Arterial Pressure; PAC – Premature Atrial Contraction; HR – Heart Rate; PP – Pulse Pressure; HF – Heart Failure; CAD – Coronary Artery Disease; RMSSD - the Root Mean Square of the Successive Differences of inter beat intervals; pvRSA/HF - peak-valley Respiratory Sinus Arrhythmia or High Frequency power; SDNN - the Standard Deviation of Normal-to-Normal RR intervals; TPE interval – T-peak-T-end Interval; MI – Myocardial Infarction; DCM – Dilated Cardiomyopathy; HCM – Hypertrophic Cardiomyopathy; PW-ASI – Pulse Wave Arterial Stiffness Index; T2DM – Type 2 Diabetes.

Supplementary Table 12 - Reports for drugged targets in each analyses type and prioritisation score

Target	Discovery AF Reports	Discovery PR Reports	Discovery LA Reports	RA TWAS Reports	LV TWAS Reports	RA eQTL Reports	LA eQTL Reports	LV eQTL Reports	RVAS Reports	Priority Score
KCNJ5	5	0	0	2	1	1	1	2	0	6
SCN10A	1	7	0	1	1	0	1	0	1	6
THRB	3	1	0	2	1	1	0	1	0	6
AOPEP	7	0	1	2	0	1	0	1	0	5
MAPT	2	0	0	2	2	1	0	1	0	5
SCN5A	4	11	0	1	0	0	1	0	1	5
KCND3	2	4	0	1	1	0	0	0	0	4
CHRM2	0	1	0	0	1	0	0	1	0	3
IGF1R	4	0	0	0	2	0	0	1	0	3
MYH6	5	3	0	0	0	0	0	0	1	3
NR3C1	4	2	0	0	1	0	0	0	0	3
PRKCA	0	1	0	0	0	1	0	1	0	3
PSMB7	1	0	0	1	1	0	0	0	0	3
SIRT1	2	0	0	1	1	0	0	0	0	3
CDC7	0	1	0	0	0	0	0	1	0	2
ERBB2	0	0	0	1	1	0	0	0	0	2
ESR2	7	0	0	1	0	0	0	0	0	2
HCN4	7	1	0	0	0	0	0	0	0	2
IL6R	2	0	0	1	0	0	0	0	0	2
NDUFB10	0	0	0	1	1	0	0	0	0	2
NPR3	0	1	0	0	0	1	0	0	0	2
PDE4B	1	0	1	0	0	0	0	0	0	2
RPSA	0	0	0	0	1	1	0	0	0	2
RAF1	0	0	0	0	0	0	1	0	0	1
ACVR2B	0	2	0	0	0	0	0	0	0	1
ADRA1A	1	0	0	0	0	0	0	0	0	1
ADRB1	0	1	0	0	0	0	0	0	0	1
BLK	1	0	0	0	0	0	0	0	0	1
CA4	1	0	0	0	0	0	0	0	0	1
CACNA1G	0	1	0	0	0	0	0	0	0	1
ENPEP	1	0	0	0	0	0	0	0	0	1

Target	Discovery AF Reports	Discovery PR Reports	Discovery LA Reports	RA TWAS Reports	LV TWAS Reports	RA eQTL Reports	LA eQTL Reports	LV eQTL Reports	RVAS Reports	Priority Score
EPAS1	0	2	0	0	0	0	0	0	0	1
EPHA3	4	0	0	0	0	0	0	0	0	1
ERBB4	3	0	0	0	0	0	0	0	0	1
FDFT1	0	0	0	0	1	0	0	0	0	1
FGFR1	0	2	0	0	0	0	0	0	0	1
FGFR2	0	1	0	0	0	0	0	0	0	1
HBEGF	1	0	0	0	0	0	0	0	0	1
IMPDH1	1	0	0	0	0	0	0	0	0	1
ITGA2B	1	0	0	0	0	0	0	0	0	1
ITGB1	0	0	0	1	0	0	0	0	0	1
KCNH2	4	0	0	0	0	0	0	0	0	1
KCNJ2	2	0	0	0	0	0	0	0	0	1
LAMB2	0	1	0	0	0	0	0	0	0	1
MC4R	0	1	0	0	0	0	0	0	0	1
MET	0	0	0	0	0	0	1	0	0	1
MTNR1A	0	1	0	0	0	0	0	0	0	1
MYH7	0	1	0	0	0	0	0	0	0	1
MYL4	2	0	0	0	0	0	0	0	0	1
NDUFAF3	0	0	0	0	0	1	0	0	0	1
PDE3A	0	1	0	0	0	0	0	0	0	1
POLR2A	1	0	0	0	0	0	0	0	0	1
PSMD3	0	0	0	0	1	0	0	0	0	1
PTK2	0	1	0	0	0	0	0	0	0	1
RPL32	1	0	0	0	0	0	0	0	0	1
RPS2	2	0	0	0	0	0	0	0	0	1
SLAMF7	1	0	0	0	0	0	0	0	0	1
SLC6A4	0	1	0	0	0	0	0	0	0	1
SMAD7	4	0	0	0	0	0	0	0	0	1
SRD5A3	0	1	0	0	0	0	0	0	0	1
SYK	1	0	0	0	0	0	0	0	0	1
TNFSF12	2	0	0	0	0	0	0	0	0	1

Target	Discovery AF Reports	Discovery PR Reports	Discovery LA Reports	RA TWAS Reports	LV TWAS Reports	RA eQTL Reports	LA eQTL Reports	LV eQTL Reports	RVAS Reports	Priority Score
TNFSF13	1	0	0	0	0	0	0	0	0	1
TNNI3	1	0	0	0	0	0	0	0	0	1
TNNT3	0	0	0	0	1	0	0	0	0	1
TUBB3	1	0	0	0	0	0	0	0	0	1
WT1	0	1	0	0	0	0	0	0	0	1
XPO1	2	0	0	0	0	0	0	0	0	1

Legend: AF - Atrial Fibrillation; LA - Left Atrium; RA - Right Atrium; LV - Left Ventricle; TWAS - Transcriptome Wide Association Study; eQTL - expression Quantitative Trait Loci colocalisation; RVAS - Rare Variant Association Study; Green shading indicates at least one report.

Supplementary Table 13 – Clinical evidence on the available cardiovascular drugs targeting the identified hits.

N=40

Drug (phase)	Clinical Indication	Clinical evidence regarding drug and AF or AF-related outcomes	Maximum level of evidence	Entries on PubMed*	Entries on Clinicaltrials.gov*
Mavacamtam (4)	Hypertrophic cardiomyopathy	A meta-analysis of 3 randomised controlled trials where mavacamtan was compared with placebo for treatment of obstructive hypertrophic cardiomyopathy demonstrated no significant difference in the outcome of incident AF (RR 1.00 95% CI 0.32-3.13)[83].	Systematic Review	16	-
Omecamtiv Mecarbil (3)	Heart Failure (not yet approved by FDA and EMA)	A sub-analysis of the GALACTIC-HF trial showed that unlike patients in sinus rhythm, patients with HFREF and AF at baseline experience no benefit in reduction of HF events and cardiovascular deaths when treated with omecamtiv mecarbil [84,85]	RCT	9	-
Ranolazine (4)	Angina	A post-hoc analysis of the MERLIN trial showed that patients assigned to ranolazine had a trend towards fewer episodes of AF than those on placebo (2.4% vs. 1.7%; P = 0.08) [86].<br>A reduction in AF burden by 59% was observed in subjects with paroxysmal AF and dual-chamber pacemakers treated with a combination of ranolazine (750 mg twice daily) and dronedarone (225 mg twice daily) (P=0.008) in the HARMONY Trial [87]<br>In the RAFFAELLO trial, there was a trend for reduction in AF recurrence in patients treated with higher doses of ranolazine (500mg BD and 750mg BD) (41.7% and 39.7%, respectively vs. 56.4% with placebo; P=0.053) [88].<br>The 2016 ESC AF guidelines state that *"... there is insufficient evidence to recommend ranolazine as an antiarrhythmic drug, alone or in combination with other antiarrhythmic drugs"* [89].	RCT<br>RCT<br>RCT	184	8
Ivabradine (4)	Angina & Heart failure	A meta-analysis of 7 RCTs demonstrated an increased risk of AF (RR 1.15 95% CI 1.07-1.24) where ivabradine was compared with controls (placebo, beta-blocker, amlodipine or ranolazine) [90].<br>A single-centre study of 16 patients with permanent AF treated with ivabradine showed improvement in rate control (-16.8bpm in average), with the magnitude of reduction positively correlated with pre-treatment heart rate (R=-0.5) [91].<br>A propensity-matched comparison of ivabradine vs beta-blockers in patients with AF showed significantly higher mortality (HR=2.01; 95% CI 1.67-2.42) and HF hospitalization (HR=1.47, 95% CI 1.11-1.94) risk in the ivabradine arm [92].	Systematic Review<br>Observational	113	7
Prenylamine (4)	Anti-angina (Withdrawn)	No controlled studies. Withdrawn due to concerns with QT prolongation and torsade de pointes.	-	1	-
Suloctidil (4)	Antiplatelet (Withdrawn)	No controlled studies. Withdrawn due to liver toxicity.	-	1	-
Bepridil (4)	Angina, & AF	Recommended as the standard antiarrhythmic drug for rhythm control in persistent AF in the Japanese pharmacotherapy guidelines for AF [93].<br>Bepidril is more effective than Amiodarone in cardioverting patients with persistent AF [94].<br>Bepridil is more effective than Aprindine in preventing post-operative AF recurrence [95].<br>The drug was withdrawn in the US due to concerns regarding QTc prolongation and Torsade de pointes.	Guideline<br>RCT<br>RCT	81	1
Acebutolol (4)	Hypertension, AF, SVT, MI & Angina	Pooled analyses of 2 trials showed a reduction in AF or SVT post-cardiac surgery with Acebutolol (RR=0.09, 95% CI 0.01-0.59) [96].	Systematic Review	13	2
Atenolol (4)	Angina, AF, SVT, MI, Hypertension, & Migraine	2020 ESC AF guidelines [97]<br>2014 ACC/AHA guidelines [98]<br>Pooling of 3 trials showing a reduction in AF or SVT post-cardiac surgery with Acebutolol (RR=0.37, 95% CI 0.16-0.83) [96].	Guideline<br>Systematic Review	143	6
Betaxolol (4)	Hypertension, & Glaucoma	Post-cardiac surgery AF was less frequent with Betaxolol than with metoprolol (12.0% vs. 21.4%; P=0.0001) [99].	RCT	6	1
Bisoprolol (4)	Angina, Heart failure, & AF	2020 ESC AF guidelines [97]<br>2014 ACC/AHA guidelines [98]	Guideline	107	10
Bucindolol (2)	AF (Not Approved)	In the GENETIC-AF trial, AF relapse rate in AF patients with ADRB1 Arg389Arg genotype and LV ejection fraction <50% was comparable with bucindolol or metoprolol (HR=1.01, 95% CI 0.71-1.42) [100].	RCT	15	1
Carvedilol (4)	AF, Angina, Hypertension, & Heart failure.	2020 ESC AF guidelines [97]<br>2014 ACC/AHA guidelines [98]	Guideline	151	11
Celiprolol (4)	Hypertension	No controlled studies.	-	4	-
Dilevalol / Labetalol (4)	Hypertension (Dilevalol withdrawn)	No controlled studies. Dilevalol withdrawn in UK due to hepatotoxicity	-	7	-
Esmolol (4)	AF, SVT, & Hypertension	2020 ESC AF guidelines [97]<br>2014 ACC/AHA guidelines [98]	Guideline	84	6
Landiolol (3)	AF, & SVT	2020 ESC AF guidelines [97]	Guideline	128	5

	(Not approved)	Once RCT showed a reduction in incident AF or SVT post cardiac surgery (RR = 0.29 95%CI 0.13-0.63) [101].	RCT		
Metoprolol (4)	Hypertension, Angina, AF, SVT, MI, Migraine, & Hyperthyroidism	2020 ESC AF guidelines [97]<br>2014 ACC/AHA guidelines [98]<br>Pooling of 7 trials showing a reduction in AF or SVT post-cardiac surgery with Acebutolol (RR=0.54, 95% CI 0.36-0.81) [96].	Guideline	336	31
Nadolol (4)	Hypertension, AF, SVT, Angina, Migraine, & Hyperthyroidism	2014 ACC/AHA guidelines [98]	Guideline	30	-
Nebivolol (4)	Hypertension, Heart failure, & AF	2020 ESC AF guidelines [97]	Guideline	15	3
Oxprenolol (4)	Hypertension, Angina, AF, & SVT	No controlled studies.	-	1	-
Pindolol (4)	Angina, & Hypertension	No controlled studies.	-	25	-
Practolol (4)	AF, & SVT (Withdrawn)	No controlled studies. Withdrawn due to severe ocular side effects.	-	42	-
Pronetalol (4)	Hypertension (Withdrawn)	No controlled studies. Withdrawn as it was found to be carcinogenic in mice.	-	2	-
Propranolol (4)	Hyperthyroidism, Hypertension, Portal hypertension, Phaeochromocytom a, Angina, MI, Anxiety, Tremor, Migraine, Hypertension, AF & SVT.	2014 ACC/AHA guidelines [98]<br>Pooling of 16 trials showing a reduction in AF or SVT post-cardiac surgery with Acebutolol (RR=0.54, 95% CI 0.48-0.60) [96].	Guideline Systematic Review	437	5
Timolol (4)	Hypertension, Angina, MI, Migraine, & Glaucoma	Pooling of 16 trials showing a reduction in AF or SVT post-cardiac surgery with Acebutolol (RR=0.33, 95% CI 0.13-0.79) [96].	Systematic Review	21	-
Xamoterol (4)	Heart failure, & AF	No controlled studies.	-	11	-
Dobutamine (4)	Inotropic support, & Cardiac stress testing	Dobutamine elicits AF triggers similarly to isoprenaline [102].	RCT	111	7
Dopamine (4)	Inotropic support	No controlled studies.	-	97	4
Droxidopa (4)	Neurogenic orthostic hypotension	No controlled studies.	-	4	-
Mephentermine (4)	Hypotension	No controlled studies.	-	1	-
Ephedrine (4)	Hypotension, reversible airway obstruction, Neurophatic oedema, & Nasal congestion	No controlled studies. Increase in atrial contractility in mouse and human atrial preparations [103].	-	17	1
Adrenaline/ Epinephrine (4)	Cardiopulmonary resuscitation, anaphylaxis, & hypotension,	No controlled studies. Known to cause AF [104,105].	-	238	12
Noradrenaline/ Norepinephrine (4)	Hypotension, & Sepsis	No controlled studies. Known to cause AF [104,105].	-	248	10
Isoproterenol (4)	Bradycardia, atrioventricular block, & asthma	No controlled studies.	-	535	27
Metaraminol (4)	Hypotension	No controlled studies.	-	6	-
Methoxamine (4)	Hypertension	No controlled studies.	-	9	-
Phenoxybenzam ine (4)	Hypertension	No controlled studies.	-	8	-
Phentolamine (4)	Hypertension	No controlled studies.	-	9	-
Phenylephrine (4)	Nasal Congestion, Mydriatic, Hypotension	No controlled studies.	-	47	2
Midodrine (4)	Hypotension	No controlled studies.	-	3	-
Doxazosin (4)	Hypertension, BPH	A sub-analysis of the ALLHAT trial showed that doxazosin was associated with higher incidence of new-onset AF/atrial flutter compared with chlorthalidone (OR: 1.35; p = 0.02) [106].	RCT	9	-

Prazosin (4)	Hypertension, BPH	No controlled studies	-	20	-
Terazosin (4)	Hypertension, BPH	No controlled studies	-	1	1
Atropine (4)	Cycloplegia, anterior uveitis, bradycardia, organophosphorus poisoning, & gastrointestinal spasm	No controlled studies. Known to cause AF [105].	-	304	-
Cilostazol (4)	Intermittent claudication	Patients treated with cilostazol and aspirin had more incident AF than those receiving only aspirin (5% vs. 2%, P=0.007) [107].<br>Suggested as a potential option for treating AF associated with bradycardia [108].<br>May supress AF inducibility [109] or attenuate fibrosis [110] in animal models.	RCT<br>RCT	36	2
Pentoxifylline (4)	Peripheral vascular disease	No controlled studies.	-	4	-
Dipyridamole (4)	Stroke	No controlled studies.	-	170	6
Enoximone (4)	Heart failure	No controlled studies.	-	6	-
Inamrinone / Amrinone (4)	Heart failure	No controlled studies.	-	22	-
Milrinone (4)	Heart failure	Milrinone associated with a lower risk of post-cardiac surgery AF than dobutamine [111].<br>Milrinone associated with increased risk of post-cardiac surgery AF [112].	RCT<br>Observational	38	3
Levosimendan (3)	Heart failure, Sepsis	Compared to dobutamine in patients undergoing CABG and with moderate to severely reduced LVEF, patients treated with levosimendan had lower incidence of AF (5% vs. 20%; P=0.044) [113].<br>Compared vs. placebo in patients undergoing CABG, AF occurred in 12% of levosimendan vs. 36% of controls (P<0.05), and duration of AF was significantly shorter (4.83±1.12 vs. 6.50±1.55 hours, P=0.028) [114].<br>In the SURVIVE trial, compared to dobutamine in patients with acute heart failure there was higher incidence of AF in the levosimendan-treated group (9.1% vs. 6.1%, P=0.05) [115].	RCT<br>RCT<br>RCT	52	1
Moricizine/ Ethmozine (4)	VA (Discontinued)	No controlled studies.<br>Prescribed in the AFFIRM trial at the discretion of physicians in the rhythm control arm [116].	-	20	1
Lidocaine (4)	VA, local anaesthetic & pain relief	Local infiltration of lidocaine around the pulmonary veins in patients undergoing lobectomy for lung cancer was associated with a lower incidence of post-operative AF (7.5% lidocaine infiltration vs. 24.4% in controls; P<0.05) [117].<br>In patients referred for elective cardioversion of AF, high-dose bolus lidocaine was ineffective in converting patients to sinus rhythm (0% success rate) [118].	Observational<br>RCT	307	6
Mexiletine (4)	VA, Myotonia	No controlled studies.	-	39	-
Alseroxylon / Rauwolfia Serpentina (4)	Hypertension (Discontinued)	No controlled studies. Ajmaline is as an alkaloid found in the roots of Rawolfia Serpentina.	-	38	-
AZD1305 (2)	AF (Discontinued)	Trend for lower AF recurrence in patients treated with oral AZD1305 (14% vs 36% in the placebo group; HR=0.36, p=0.056) [119]<br>AZD1305 was effective in converting AF to SR, with 8% to 50% success rate within 90min of starting drug infusion (vs 0% in the placebo group) [120].<br>The AZD1305 development programme was discontinued as the drug was associated with QT prolongation and torsade de pointes.	RCTs	10	3
AZD7009 (2)	AF (Discontinued)	AZD7009 infusion was used for cardioversion of patients with persistent AF or atrial flutter. The three highest concentrations converted 70%, 64%, and 45% of AF patients, after a 26 to 62min, vs 0% in the placebo-arm [121].<br>Comparison of 30min (3.25mg/min) vs 15min (3.25 or 4.4mg/min) AZD7009 infusion vs. placebo showed that longer duration infusion cardioverted more patients, and placebo was ineffective (50% vs. 17.9% vs. 19.4% vs. 0%) [122].	RCTs	14	1
Tedisamil (3)	AF (Discontinued)	Initially developed as an anti-angina drug. Tedisamil at dosages of 0.4 and 0.6 mg/kg was superior to placebo in converting AF or atrial flutter (41 to 51% vs 7%; P<0.001) [123].<br>Discontinued due to the narrow therapeutic margin and risk of side effects.	RCT	24	3
Tocainide (4)	VA (Discontinued)	No controlled studies.	-	6	-
Procainamide (4)	AF, VA	Low efficacy of IV Procainamide vs DCCV for atrial flutter (27% vs. 93%) [124].<br>Procainamide IV over 30-60 min cardioverted 52-54% acute AF patients vs. 88.4-96% in the DC cardioversion arm (p<0.05) [125,126].<br>Trials assessed Procainamide vs propafenone or placebo for treatment [127] and prevention [128,129] of AF post-cardiac surgery with a reduction in events.<br>For cardioversion of pre-excited AF without haemodynamic compromise: 2014 AHA/ACC/HRS guidelines [98]	RCT<br>Guideline	370	8
Vernakalant (4)	AF	2020 ESC AF guidelines [97]	Guideline	191	16

	(EMA only)				
Dronedarone (4)	AF	2020 ESC AF guidelines [97]<br>2014 ACC/AHA guidelines [98]	Guideline	503	39
Flecainide (4)	AF, SVT, VA	2020 ESC AF guidelines [97]<br>2014 AHA/ACC/HRS guidelines [98]	Guideline	725	44
Encainide (4)	AF, SVT, VA (withdrawn)	No controlled studies.	-	35	-
Propafenone (4)	AF, SVT, VA	2020 ESC AF guidelines [98]<br>2014 AHA/ACC/HRS guidelines [98]	Guideline	629	34
Quinidine (4)	AF, Brugada Syndrome, Short QT syndrome, Idiopathic VF, Malaria	2014 AHA/ACC/HRS guidelines [98]	Guideline	1084	9
Disopyramide (4)	AF, HOCM, VA	2014 AHA/ACC/HRS guidelines [98]	Guideline	233	3
Amiodarone (4)	AF, SVT, VA	2020 ESC AF guidelines [97]<br>2014 AHA/ACC/HRS guidelines [98]	Guideline	3039	135
Ibutilide (4)	AF, SVT	2020 ESC AF guidelines [97]<br>2014 AHA/ACC/HRS guidelines [98]	Guideline	269	5
Dofetilide (4)	AF<br>(not available in Europe or Australia)	2014 AHA/ACC/HRS guidelines [98]	Guideline	305	12
Sotalol (4)	AF, SVT, VA	2020 ESC AF guidelines [97]<br>2014 AHA/ACC/HRS guidelines [98]	Guideline	709	41
Carperitide (3)	Acute HF<br>Approved in Japan	In the NU-HIT *for Left Ventricular Dysfunction study*, carperitide significantly reduced the incidence of post-operative AF (41/335 vs 110/333 in the placebo group; p<0.0001) [130]. Similar findings had been previously reported in a trial of patients undergoing emergency CABG for acute coronary syndrome [131].	RCT	24	-
Abiciximab (4)	ACS – PCI, CVA-percutaenous management	No controlled studies.	-	10	-
Eptifibatide (4)	ACS – PCI	No controlled studies.	-	9	-
Tirofiban (4)	ACS – PCI, CVA-percutaenous management	No controlled studies.	-	14	-
Acetazolamide (4)	Heart failure, Glaucoma	No controlled studies.	-	6	

Legend: FDA – US Food and Drug Administration; EMA – European Medicines Agency; GALACTIC-HF - Registrational Study With Omecamtiv Mecarbil (AMG 423) to Treat Chronic Heart Failure With Reduced Ejection Fraction; HFREF – Heart Failure with Reduced Ejection Fraction; MERLIN - Metabolic Efficiency With Ranolazine for Less Ischemia in Non ST-Elevation Acute Coronary Syndrome; HARMONY - A Study to Evaluate the Effect of Ranolazine and Dronedarone When Given Alone and in Combination in Patients With Paroxysmal Atrial Fibrillation; RAFFAELLO -Ranolazine in Atrial Fibrillation Following An ELectricaL CardiOversion; ESC – European Society of Cardiology; RR- Risk Ratio; CI – Confidence Interval; HR – Hazard Ratio; LVEF – Left Ventricular Ejection Fraction; ALLHAT - Antihypertensive and Lipid-Lowering Treatment to Prevent Heart Attack Trial; ARB – Angiotensin Receptor Blocker; OR - Odds Ratio; SVT – Supraventricular Tachycadia; AHA/ACC/HRS – American Heart Association/American College of Cardiology/Heart Rhythm Society; MI – Myocardial Infarction; BPH – Benign Prostatic Hypertrophy; CABG – Coronary Artery Bypass Graft; VA – Ventricular Arrhythmias; AFFIRM - Atrial Fibrillation Follow-Up Investigation of Rhythm Management; DCCV – Direct-Current Cardioversion; VT/VF – Ventricular Tachycardia/Fibrillation; HOCM – Hypertrophic Obstructive Cardiomyopathy; NU-HIT - Nihon University working group study of low-dose Human ANP Infusion Therapy during cardiac surgery.

Supplementary Table 14 – Characteristics of randomised controlled trials (RCTs) providing evidence for AF outcomes on drugs linked to targets.

Study	CV indication	Total	Patient population	Age (SD)	Female (%)	Intervention	Int N	Comparison	Comp N	Outcome	Effect Estimate / Results	Overall
Teerlink et al. 2021	Y	8232	Symptomatic chronic heart failure and an LVEF ≤35%	64.5 (11.3)	1749 (21.2)	Omecamtiv Mecarbil	4120	Placebo	4112	HF and cardiovascular death	HR for composite of a heart-failure event or cardiovascular death: 1.05 (0.93-1.18) in AF or atrial flutter patients, and 0.86 (0.79-0.94) in patients without AF	Neutral
Scirica et al. 2015	Y	6560	Non-ST elevation ACS within 48 hours of ischemic symptom	17.5% > 75yrs	2269 (35)	Ranolazine	3279	Placebo	3281	Clinical AF	HR for any clinical AF event: 0.71, 0.55-0.92, p=0.01; trend towards fewer episodes of AF:75 (2.4%) vs. 55 (1.7%) patients, p= 0.08; pattern of AF burden on continuous ECG: paroxysmal AF (0.01–98%), 18 patients on ranolazine vs. 48 on placebo; predominantly chronic AF (>98%): 28 vs. 20 patients; and clinically insignificant AF (<0.01%): 5 vs. 7 patients; three-way p= 0.01	Beneficial
Reifell et al. 2015	Y	131	Paroxysmal AF and implanted pacemakers	72.2 (8.8)	68 (52)	Ranolazine+Dronedarone	53	Ranolazine alone, Dronedarone alone, Placebo	78	AF burden	No measure, only rates and means were reported: ranolazine 750 mg BID/dronedarone 225 mg BID reduced AF burden by 59% versus placebo (P=0.008), whereas ranolazine 750 mg BID/dronedarone 150 mg BID reduced burden by 43% (P=0.072); Mean percent change from baseline in atrial fibrillation burden over 12 weeks: −45.5±10.7 and −59.1±10.5; p=0.008.	Beneficial
De Ferrari et al. 2015	Y	238	Persistent AF last < 6 months, 2h after cardioversion	65 (10)	54 (22.7)	Ranolazine	183	Placebo	55	AF recurrence	HR for AF recurrence: 0.74, 0.50–1.03, p=0.053 comparing higher doses of ranolazine vs placebo, and 0.73, 0.51–1.00, p=0.035 comparing higher doses of Ranolazine vs lower dose; median time to first documented AF recurrence was 58, 51, and 117 days in the placebo, ranolazine 375-mg, and ranolazine 750-mg groups, respectively. In the ranolazine 500-mg group, the estimate could not be calculated because the actuarial event rate did not reach 50%	Beneficial

Study	CV indication	Total	Patient population	Age (SD)	Female (%)	Intervention	Int N	Comparison	Comp N	Outcome	Effect Estimate / Results	Overall
Yamase et al. 2012	Y	40	Persistent AF	61 (8)	5 (12.5)	Bepridil	20	Amiodarone	20	Cardioversion to sinus rhythm	No measure, only rates reported: sinus rhythm was restored in seven (35%) of Amiodarone treated patients and in 17 (85%) of Bepridil treated patients, p<0.05; After pharmacological or DC cardioversion, sinus rhythm could be maintained in 50% of Amiodarone-treated patients and in 75% of Bepridil treated patients during an average follow-up of 15 months.	Beneficial
Ozawa et al. 2015	Y	72	AF post-cardiovascular surgery	68 (8)	45 (62.5)	Bepridil	37	Aprindine	35	AF recurrence	No measure, only rates reported: AF recurrence at 14 days occurred in 51% of patients treated with Bepridil and 24% of those treated with Aprindine; long rank p=0.0278)	Harmful* (vs other active treatment targeting SCN5A)
Sezai et al. 2011	Y	140	Adults undergoing CABG	67.6 (7.1)	12 (8.5)	Landiol	70	Control	70	Incident AF or SVT post-cardiac surgery	RR of incident AF 0.29, 0.13-0.63, p=0.002	Beneficial
Iluita et al. 2009	Y	1352	Adults undergoing CABG	63 (12.5)	441 (32.6)	Betaxolol	674	Metoprolol	678	In-hospital AF post-cardiac surgery	No measure, only rates reported: AF in hospital was seen in 81/674 (12.02%) of Betaxolol treated patients and 145/678 (21.39%) of patients treated with metoprolol, p=0.0001	Beneficial* (vs other active substance of the same drug class)
Piccini et al. 2019	Y	258	Patients with LVEF<50%, symptomatic AF, and the ADRB1 Arg389Arg genotype	65.6 (10.1)	46 (18)	Bucindolol	126	Metoprolol	132	Composite of atrial flutter, AF or all-cause mortality	HR: 1.01, 0.71-1.42 for all-cause atrial arrhythmia relapse and all-cause mortality; Analysis of implantable monitor data showed similar results for time to first atrial arrhythmia relapse: HR: 0.75, 0.43-1.32	Neutral* (vs other active compound of the same drug class)
Gianni et al. 2020	Y	50	Patients undergoing AF ablation	63 (7)	14 (28)	Dobutamine	cross-over	Isoproterenol	cross-over	Atrial arrhythmias	No measure, only rates/counts were provided: PAC 37 of 48 versus 43 of 48, sPACs in 7 of 48 versus 8 of 48, and AT in 9 of 48 versus 10 of 48; and PACs triggering AF in 2 of 48 versus 5 of 48, whereas no AFL was induced during either drug challenge	Neutral* (vs other active compound of the same drug class)
Haywood et al. 2009	Y	18087	Hypertensive patients with one additional CAD risk factor	67 (8)	85001 (47)	Doxazosin	11695	Chlorthalidone	6392	New-onset AF or atrial flutter	OR: 1.35; p=0.02; doxazosin vs chlorthalidone	Harmful
Aoki et al. 2000	Y	1194	Noncardioembolic Stroke Patients	69 (13.4)	401 (33.6)	Cilostazol + Aspirin	596	Aspirin	598	Incident AF	No measure, only rates were reported for incident AF: cilostazol and aspirin 5% vs aspirin 2% (p=0.007)	Harmful
Feneck et al. 2001	Y	120	Patients with low cardiac output after cardiac surgery	64 (1)	49 (40.8)	Milrinone	60	Dobutamine	60	Incident AF post-cardiac surgery	No measure, only rates were provided: incident AF occurred more often in Dobutamine-treated (18%) than in Milrinone-treated patients; p < 0.04.	Beneficial* (vs other active tratment)

Study	CV indication	Total	Patient population	Age (SD)	Female (%)	Intervention	Int N	Comparison	Comp N	Outcome	Effect Estimate / Results	Overall
Kandasamy et al. 2007	Y	80	Patients with moderate to severe LV dysfunction undergoing off-pump coronary artery bypass grafting	55 (3)	16 (20)	Levosimendan	40	Dobutamine	40	Post-operative AF	No measure, only rates were provided: incident AF occurred more often in Dobutamine-treated (20%) than in Levosimendan-treated patients (5%); p=0.044.	Beneficial* (vs other active substance)
Abacilar et al. 2013	Y	200	Patients with poor LV function undergoing coronary artery bypass grafting	61 (1)	86 (43)	Levosimendan	100	Placebo	100	Post-operative AF	No measure, only rates and means were provided: incident AF occurred more often in Placebo-treated (36%) than in Levosimendan-treatd patients (12%); p<0.05; Duration of AF in the levosimendan group was significantly shorter than that in the control group (4.83±1.12 and 6.50±1.55 hours, respectively; P = 0.028)	Beneficial
Mebazaa et al. 2007	Y	1327	Patients with acute HF in need of IV inotropic support	67 (12)	371 (28.0)	Levosimendan	664	Dobutamine	663	Post-operative AF	No measure, only rates and means were provided: incident AF occurred more often in Levosimendam-treated (9.1%) than in Dobutamine-treatd patients (6.1%); p=0.02	Harmful* (vs other active tratment)
Marrouche et al. 2000	Y	20	Patients referred for cardioversion of AF	62 (12)	5 (25)	Lidocaine	10	Placebo	10	Cardioversion to sinus rhythm	No measure, only rates were provided: 0% cardioverted to SR with lidocaine and 0% with placebo	Neutral
Egstrup et al. 2011	Y	65	Patients with history of AF in stable sinus rhythm	65 (10)	26 (40)	AZD1305	43	Placebo	22	AF recurrence	No measure, only rates were provided: 6 (14%) treated with AZD1305 and 8 (36%) treated with placebo relapsed; p=NS	Beneficial
Ronaszeki et al. 2011	Y	154	Patients in AF	65 (9)	62 (40.3)	AZD1305	128	Placebo	26	Cardioversion to sinus rhythm	No measure, only rates were provided: 46 (35.9%) treated with AZD1305 and 0 (0%) treated with placebo were cardioverted; p<0.05	Beneficial
Crijns et al. 2006	Y	122	Patients in persistent AF or atrial flutter	64 (11)	48 (39.3)	AZD7009	86	Placebo	36	Cardioversion to sinus rhythm	No measure, only rates were provided: 22 (25.6%) treated with AZD7009 and 0 (0%) treated with placebo were cardioverted; p<0.05; Shorter time to cardioversion was observed with higher doses	Beneficial
Geller et al. 2009	Y	167	Patients in AF scheduled for cardioversion	62 (10)	46 (27.5)	AZD7009	124	Placebo	43	Cardioversion to sinus rhythm	No measure, only rates were provided: 43 (37.1%) treated with AZD7009 and 0 (0%) treated with placebo were cardioverted; p<0.05;	Beneficial
Hohnloser et al. 2004	Y	175	Patients with symptomatic paroxysmal AF or atrial flutter	63.60 (13.7)	67 (38)	Tedisamil	116	Placebo	59	Cardioversion to sinus rhythm	No measure, only rates were provided: 52 (45.6%) treated with tedisamil and 4 (7%) treated with placebo were cardioverted; p<0.001	Beneficial

Study	CV indication	Total	Patient population	Age (SD)	Female (%)	Intervention	Int N	Comparison	Comp N	Outcome	Effect Estimate / Results	Overall
Stiell et al. 2021	Y	76	Patients with acute atrial flutter	66.3 (13.1)	30 (39.5)	Procainamide	33	Placebo	43	Cardioversion to sinus rhythm	No measure, only rates were provided: 7 (21.2%) treated with Procainamide and 2 (4.7%) treated with placebo were cardioverted; p<0.05	Beneficial
Scheuermeyer et al. 2019	Y	84	Patients with acute paroxysmal atrial fibrillation	58.6 (10.6)	32 (38.1)	Procainamide	43	Placebo	41	Cardioversion to sinus rhythm	No measure, only rates were provided: 2 (54%) treated with Procainamide and 0 (0%) treated with placebo were cardioverted; p<0.05	Beneficial
Stiell et al. 2021	Y	396	Patients with acute paroxysmal atrial fibrillation	66 (15)	136 (34.3)	Procainamide	204	Placebo	192	Cardioversion to sinus rhythm	No measure, only rates were provided: 111 (54%) treated with Procainamide and 16 (8.3%) treated with placebo were cardioverted; p<0.05	Beneficial
Gold et al. 1996	Y	100	Patients undergoing CABG	64 (1)	21 (21)	Procainamide	50	Placebo	50	Post-operative AF	No measure, only rates were provided: 13 (26%) treated with Procainamide and 19 (38%) treated with placebo were cardioverted; p=NS	Neutral
Laub et al. 1993	Y	46	Patients undergoing CABG	63.6 (-)	7 (15.2)	Procainamide	22	Placebo	24	Post-operative AF (number of episodes)	No measure, only rates were provided: procainamide group had 5 episodes in 129 patient days at risk [3.9%/day at risk] vs controls having 17 episodes in 161 patient days at risk [10.6%/day at risk], p=0.04	Beneficial
Sezai et al. 2007	Y	124	Patients undergoing CABG	67 (10)	37 (29.8)	Carperitide	63	Placebo	61	Post-operative arrhythmia	No measure, only rates were provided: 14 (22.2%) treated with Carperitide and 26 (42.6%) treated with placebo developed AF; p<0.05	Beneficial
Sezai et al. 2015	Y	668	Patients undergoing CABG	67 (9)	121 (18.1)	Carperitide	335	Placebo	333	Post-operative AF	No measure, only rates were provided: 41(12.2%) from the carperitide group vs 110 (32.7%) from the placebo group (P<0.0001)	Beneficial
Raskin et al 2005	N	348	Bilateral peripheral neuropathy secondary to diabetes	58.8 (10.1)	186 (53.4)	Duloxetine	232	Placebo	116	Incident AF	Event rates reported: 1 (0.9%) in duloxetine group vs 0 (0%) in placebo group (P = 0.3)	Neutral
Perez et al 2015	N	26379	Postmenopausal women	63.3 (7.3)	26379 (100)	Conjugated Estrogens	13319	Placebo	13060	Incident AF	HR of incident atrial fibrillaion 1.12 (1.00-1.24) p=0.05	Neutral
Gridelli et al 2001	N	98	Patients >70 years old with stage III/IV NSCLC	75 (3.5)	15 (15.3)	Vinorebline (+ gemicitabine)	49	Gemcitabine	49	Incident AF	Event rates reported: 4 (8.2%) in vinorebline + gemcitabine group vs 0 (0%) in gemcitabine only group (P=0.04)	Harmful
Janssen et al 2021	N	231	Patients >65 years old with AML	70 (3.53)	83 (36)	Tosedostat (+ routine care)	116	Routine care	115	Incident AF	Event rates reported: AF developed in 20 (18%) in tosedostat + routine care group vs 5 (4%) in routine care only group from cycle 1 (P=0.007), 6 (9%) vs 4 (5%) respectively in cycle 2 (P=0.3)	Harmful

Study	CV indication	Total	Patient population	Age (SD)	Female (%)	Intervention	Int N	Comparison	Comp N	Outcome	Effect Estimate / Results	Overall
Iskandar et al 2017	N	60	Patients attending for index AF ablation procedure	63 (8.7)	14 (23)	Prednisone	30	Placebo	30	Freedom from AF at 12 months	Event rates reported: 18 (60%) in prednisolone group vs 21 (70%) in placebo group P = 0.417	Neutral
Iskandar et al 2017	N	60	Patients attending for index AF ablation procedure	63 (8.7)	14 (23)	Prednisone	30	Placebo	30	Early Recurrence of AF (0-3 months)	Event rates reported: 8 (27%) in prednisolone group vs 5 (17%) in placebo group P = 0.347	Neutral
Papi et al 2018	N	1532	Patients with symptomatic COPD	64.5 (7.7	432 (28.2)	Beclometasone (+ Formoterol + Glycopyrronium)	764	Indacterol + Glycopyrronium	768	Incident AF	Event rates reported: 7 (0.9%) in Indacterol + glycopyrronium group vs 0 (0%) in beclometasone + formoterol + glycopyrronium group (P =0.008)	Beneficial
Shaibani et al 2009	N	468	Patients with painful diabetic neuropathy	59.8 (10.)	204 (43.5)	Erlosamide/Lacosamide	403	Placebo	65	Incident AF	Event rates reported: 2 (0.5%) in lacosamide group vs 0 (0%) in placebo group (P=0.57)	Neutral
Alboni et al 1997	N	71	Patients ≥45 years old with clinical evidence of sinus node dysfunction on ECG and symptoms	72 (10.9)	31 (43.7)	Theophylline	36	Placebo	35	Chronic AF	Event rates reported: 2 (6%) in theophylline patients vs 4 (11%) in placebo patients. P value not given but not significant.	Neutral
Bethge et al 1982	N	40	Patients with diagnosed clinical depression	41.9 (10..0)	24 (60)	Nomifensine	20	Maprotiline	20	Incident AF	Event rates reported: 0 (0%) in nomifensine patients vs 0 (0%) in maprotiline patients.	Neutral

Legend: CV – Cardiovascular; SD – Standard Deviation; Int N – Intervention arm total; Comp N – Comparison arm total; LVEF – Left Ventricular Ejection Fraction; HF – Heart Failure; AF – Atrial Fibrillation; HR – Hazard Ratio; RR – Relative Risk or Risk Ratio; OR – Odds Ratio; ACS -Acute Coronary Syndrome; ECG – Electrocardiogram; BID – Twice daily dosing; CABG – Coronary Artery Bypass Grafting; DC – Direct Current; SVT – Supraventricular Tachycardia; CAD – Coronary Artery Disease; PAC – Premature Atrial Contraction; LV – Left Ventricle; IV – Intravenous; NSCLC – Non-Small Cell Lung Cancer; AML – Acute Myeloid Leukaemia; COPD – Chronic Obstructive Pulmonary Disease.

Supplementary Table 15 – Risk of Bias (RoB) for randomised controlled trials (RCTs) providing evidence for AF outcomes on drugs linked to targets.

Study	CV indication	Random Sequence Generation	Allocation Concealment	Blinding of Participants & Personel	Blinding of Outcome Assessment	Incomplete Outcome Data	Selective Reporting	Other Sources of Bias
Teerlink et al. 2021	Y	1	1	1	1	1	1	2 (industry funding)
Scirica et al. 2015	Y	1	1	1	1	1	2 (post-hoc analysis)	2 (industry funding)
Reifell et al. 2015	Y	2 (no information provided)	2 (no information provided)	1	1	1	1	2 (industry funding)
De Ferrari et al. 2015	Y	2 (no information provided)	2 (no information provided)	1	1	1	1	2 (industry funding)
Yamase et al. 2012	Y	2 (no information provided)	2 (no information provided)	2 (unclear; objective endpoints)	2 (unclear; objective endpoints)	1	1	2 (not registered on a plaftform)
Ozawa et al. 2015	Y	2 (no information provided)	2 (no information provided)	2 (unclear; objective endpoints)	2 (unclear; objective endpoints)	1	1	1
Sezai et al. 2011	Y	1	2 (no information provided)	1	1	1	1	1
Iluita et al. 2009	Y	2 (no information provided)	1	2 (unclear; objective endpoints)	2 (unclear; objective endpoints)	1	1	2 (not registered on a plaftform)
Piccini et al. 2019	Y	2 (insufficient information)	2 (insufficient information)	1	1	3 (high withdrawal rate: 20%)	1	2 (industry funding)
Gianni et al. 2020	Y	2 (insufficient information)	2 (insufficient information)	2 (unclear; objective endpoints)	2 (unclear; objective endpoints)	1	1	2 (cross-over vs drug half lives)
Haywood et al. 2009	Y	1	1	1	1	1	1	2 (some industry funding)
Aoki et al. 2000	Y	1	1	2 (unclear; objective endpoints)	2 (unclear; objective endpoints)	1	2 (post-hoc analysis)	2 (not registered on a plaftform)
Feneck et al. 2001	Y	2 (insufficient information)	1	2 (unclear; objective endpoints)	2 (unclear; objective endpoints)	1	1	2 (not registered on a plaftform)
Kandasamy et al. 2007	Y	1	2 (no information provided)	1	1	1	1	2 (not registered on a plaftform)
Abacilar et al. 2013	Y	2 (insufficient information)	2 (insufficient information)	1	2 (unclear; objective endpoints)	1	1	2 (not registered on a plaftform)
Mebazaa et al. 2007	Y	1	1	1	1	1	1	2 (industry funding)
Marrouche et al. 2000	Y	2 (insufficient information)	2 (insufficient information)	1	1	1	1	2 (not registered on a plaftform)
Egstrup et al. 2011	Y	2 (insufficient information)	1	1	1	1	2 (post-hoc analysis)	2 (industry funding)
Ronaszeki et al. 2011	Y	1	1	1	1	1	1	2 (industry funding)
Crijns et al. 2006	Y	2 (insufficient information)	2 (insufficient information)	1	1	1	1	2 (industry funding)
Geller et al. 2009	Y	2 (insufficient information)	2 (insufficient information)	1	1	1	1	2 (not registered on a plaftform)
Hohnloser et al. 2004	Y	2 (insufficient information)	2 (insufficient information)	1	1	1	1	2 (industry funding)

Study	CV indication	Random Sequence Generation	Allocation Concealment	Blinding of Participants & Personel	Blinding of Outcome Assessment	Incomplete Outcome Data	Selective Reporting	Other Sources of Bias
Stiell et al. 2021	Y	1	1	1	1	1	2 (reported but no planned endpoint)	1
Scheuermeyer et al. 2019	Y	1	1	1	1	1	2 (reported but no planned endpoint)	1
Stiell et al. 2021	Y	1	1	1	1	1	2 (reported but no planned endpoint)	1
Gold et al. 1996	Y	2 (insufficient information)	2 (insufficient information)	1	1	1	1	3 unsure about randomization as differences present at baseline across groups
Laub et al. 1993	Y	2 (insufficient information)	2 (insufficient information)	1	1	1	1	2 (not registered on a plaftform)
Sezai et al. 2007	Y	2 (insufficient information)	2 (insufficient information)	2 (insufficient information)	2 (insufficient information)	1	1	2 (not registered on a plaftform)
Sezai et al. 2015	Y	2 (insufficient information)	2 (insufficient information)	1	1	1	1	1
Raskin et al 2005	N	1	2 (insufficient information)	1	1	2 (high withdrawal rate: 15%)	1	2 (industry funding, not registered on a plaftform)
Perez et al 2015	N	2 (insufficient information)	2 (insufficient information)	2 (insufficient information)	2 (insufficient information)	1	1	1
Gridelli et al 2001	N	1	2 (insufficient information)	2 (insufficient information)	2 (insufficient information)	1	1	2 (industry funding)
Janssen et al 2021	N	2 (insufficient information)	2 (insufficient information)	2 (insufficient information)	2 (insufficient information)	1	1	2 (industry funding, not registered on a plaftform)
Iskandar et al 2017	N	2 (insufficient information)	1	1	1	1	1	2 (not registered on a plaftform)
Iskandar et al 2017	N	2 (insufficient information)	1	1	1	1	1	2 (not registered on a plaftform)
Papi et al 2018	N	1	1	1	1	1	1	2 (industry funding)
Shaibani et al 2009	N	1	1	1	1	1	1	1
Alboni et al 1997	N	2 (insufficient information)	2 (insufficient information)	2 (insufficient information)	2 (insufficient information)	1	1	2 (not registered on a plaftform)
Bethge et al 1982	N	2 (insufficient information)	2 (insufficient information)	2 (insufficient information)	2 (insufficient information)	2 (insufficient information)	1	2 (not registered on a plaftform)

Legend: CV – Cardiovascular; Green shading and score of 1 – Low risk of bias; Yellow shading and score of 2 – Moderate risk of bias; Red shading and score of 3 – High risk of bias.

Supplementary Table 16 – Characteristics of systematic reviews providing evidence for AF outcomes on drugs linked to targets.

Study	CV indication	Number of studies	Total	Patient population	Intervention	Int N	Comparison	Comp N	Outcome	Effect Estimate / Results	Overall	Quality of evidence measure	Quality of evidence assessment
Ismayl et al 2023	Y	3	421	Patients with obstructive hypertrophic cardiomyopathy	Mavacamtam	219	Placebo	202	Incident AF	RR: 1.00 (0.32-3.13), P=0.99, I^2 = 0%	Neutral	RoB	All trials were considered low risk of bias apart from MAVERICK-HCM which had a moderate risk level for selective outcome reporting
Martin et al. 2014	Y	11	21571	Heart failure and/or coronary artery disease patients	Ivabradine	Not provided	Placebo, or beta-blocker, amlodipine or ranolazine	Not provided	Incident AF	RR of AF 1.15, 1.07-1.24, P=0.0027; I^2=0	Harmful	RoB	All trials were considered low risk of bias
Arsenault et al. 2013	Y	2	171	Adults undergoing cardiac surgery	Acebutol	82	Control	89	Incident AF or SVT post-cardiac surgery	RR of incident AF 0.09, 0.01-0.59, P=0.01; I^2=41%	Beneficial	RoB	Daudon 1986: 3 domains with unclear and 2 domains with high risk; Materne 1985: all domains unclear risk
Arsenault et al. 2013	Y	3	210	Adults undergoing cardiac surgery	Atenolol	105	Control	105	Incident AF or SVT post-cardiac surgery	RR of incident AF 0.37, 0.16-0.83, P=0.02; I^2=31	Beneficial	RoB	Lamb 1988: 6 domains with unclear risk; Matangi 1989: 5 domains with unclear risk; Yazicioglu 2002: 5 domains with unclear risk
Arsenault et al. 2013	Y	7	1794	Adults undergoing cardiac surgery	Metoprolol	892	Control	902	Incident AF or SVT post-cardiac surgery	RR of incident AF 0.54, 0.36-0.81, P=0.003; I^2=72%	Beneficial	RoB	Auer 2004: low risk for all domains;Connolly 2003: 2 domains unclear and 1 domain high risk; Imrem 2007: high risk for 1 domain; Janssen 1986: three domains unclear risk and two high-risk domains; Lucio 2004: one domain unclear risk and two high-risk domains; Paull 1997: 4 domains unclear and one high-risk domain; Wencke 1999: two unclear risk domains.

Study	CV indication	Number of studies	Total	Patient population	Intervention	Int N	Comparison	Comp N	Outcome	Effect Estimate / Results	Overall	Quality of evidence measure	Quality of evidence assessment
Arsenault et al. 2013	Y	16	1859	Adults undergoing cardiac surgery	Propranolol	886	Control	973	Incident AF or SVT post-cardiac surgery	RR of incident AF 0.48, 0.38-0.60, P<0.00001; I^2=6%	Beneficial	RoB	Abel 1983: 3 unclear risk domains and 3 high-risk domains; Babin-Ebbel 1996: one unclear risk domain and 4 high-risk domains; Bert 2001: 2 domains with unclear risk and 1 with high-risk; Gun 1998: 2 unclear risk domains and 1 high-risk domain; Ivey 1983: one high-risk domain; Martinussen 1998: 3 domains unclear risk and one high-risk domain; Matangi 1985: three domains unclear risk; Mohr 1981: three domains unknown risk and 4 high-risk domains; Myhre 1984: one domain unclear risk, and 7 domains high-risk of bias; Oka 1980: 3 domains unclear risk and 4 high-risk; Ormerod 1984: all domains unclear risk; Rubin 1987: five domains high-risk; Salazar 1979: 5 domains unclear risk and 2 high-risk domains; Silverman 1982: 3 domains unclear risk and 4 high-risk domains; Stephensen 1980: 6 domains unclear risk and 1 high-risk domain; Williams 1982: 5 domains unclear risk and 2 high-risk domains.
Arsenault et al. 2013	Y	2	173	Adults undergoing cardiac surgery	Timolol	87	Control	86	Incident AF or SVT post-cardiac surgery	Relative risk of incident AF 0.33, 0.13-0.79, P=0.01; I^2=0%	Beneficial	RoB	Vecht 1986: 4 domains unclear risk; White 1984: 4 domains unclear risk.
Kommu et al 2023	N	6	1588	Cardiac surgery patients	Colchicine	793	Placebo	795	AF incidence over study follow up	RR: 0.70 (0.58-0.84), P<0.001, I^2 = 0%	Benefit	None provided	-

Study	CV indication	Number of studies	Total	Patient population	Intervention	Int N	Comparison	Comp N	Outcome	Effect Estimate / Results	Overall	Quality of evidence measure	Quality of evidence assessment
Kommu et al 2023	N	2	328	Post PVI patients	Colchicine	145	Placebo	183	AF reucrrence over 12 months follow up	RR: 0.58 (0.42-0.80), P <0.001, I^2 = 0%	Benefit	None provided	-
Tharmapoopathy et al 2021	N	7	793	Patients attending for adult cardiac surgery	Liothryonine	343	Placebo	450	Incident AF	RR: 1.03 (0.87-1.27), P = 0.99, I^2 = 0%	Neutral	RoB	Low for all trials
Oba et al 2013	N	14	11257	Patients with COPD	Roflumilast	5766	Placebo	5491	Incident AF	Incidence rate difference: 0.0025 (0.0005 - 0.0045), P = 0.02, I^2 not provided	Benefit	None provided	-
Whitlock et al 2008	N	2	696	Cardiac surgery patients	Hydrocortisone	346	Placebo	350	New onset AF	RR: 0.77 (0.41-1.42), P =0.40, I^2 = 47%	Neutral	Jadad score	Halonen et al: High quality (5), Sano et al: Low quality (1)
Whitlock et al 2008	N	4	1297	Cardiac surgery patients	Dexamethasone	646	Placebo	651	New onset AF	RR: 0.69 (0.53-0.90), P =0.005, I^2 = 15%	Benefit	Jadad score	High quality (4-5) for all studies
Whitlock et al 2008	N	8	510	Cardiac surgery patients	Methylprednisolone	268	Placebo	242	New onset AF	RR: 0.71 (0.54-0.94), P =0.02, I^2 = 35%	Benefit	Jadad score	High quality (3-5) for all studies apart from Schurr et alL: Low quality (1)

Legend: CV – Cardiovascular; Int N – Intervention arm total; Comp N – Comparison arm total; AF – Atrial Fibrillation; HR – Hazard Ratio; RR – Relative Risk or Risk Ratio; I^2 – Heterogeneity measure (Random effects measure used if greater than 40%); RoB – Risk of Bias; SVT – Supraventricular Tachycardia; COPD – Chronic Obstructive Pulmonary Disease; PVI – Pulmonary Vein Isolation

Supplementary Table 17 – Clinical evidence on the available non-cardiovascular drugs targeting the identified hits.

N=30

Drug (phase)	Clinical Indication	Clinical evidence regarding drug and AF or AF-related outcomes	Maximum level of evidence	Entries on PubMed*	Clinicaltrials.gov
Dalfampridine/4-aminopyridine (4)	Multiple Sclerosis	No controlled studies. Animal and cell studies show different impact of Dalfampridine on atrial action potential duration in sinus rhythm vs. AF [132].	-	33	-
Guanidine (4)	Eaton-Lambert syndrome	No controlled studies.	-	104	-
Metformin (4)	Type-2 Diabetes mellitus	After adjustment for confounders, initiation of metformin, when compared to other non-insulin monotherapies, was not associated with reducing the risk of developing AF (HR=0.92, 95%CI 0.69-1.21) [133]<br>After adjustment to confounders, use of Metformin was associated with increased risk of new onset AF among patients with type-2 Diabetes mellitus (HR=1.09, 95%CI 1.01-1.18) [134].<br>In the Taiwan National Health Insurance Research Database, after adjustment, in patients not treated with other antidiabetic agents, use of metformin was associated with a reduction of new onset AF (HR=0.81, 95% 0.76-0.86) [135].<br>A network meta-analysis including one RCT, and 4 observational studies (3 of them nationwide registries), showed that GLP-1 receptor antagonists significantly reduced AF vs. metformin (OR=0.17, 95% CI 0.04-0.61) [136].<br>Three ongoing RCTs post-catheter ablation for AF [137,138].	Observational<br>Systematic Review - Observational<br>Ongoing RCT	70	4
Topiramate (4)	Migraine, Epilepsy	No controlled studies.	-	4	-
Lamotrigine (4)	Epilepsy & bipolar disorder	No controlled studies.	-	5	-
Erlosamide/ Lacosamide (4)	Epilepsy	Shaibani et al. reported 1.5% incident AF cases with Lacosamide 600mg OD vs. ≤1.5% in the Placebo or low-dose groups [139].	RCT	7	-
Carbamazepine (4)	Epilepsy, Trigeminal neuralgia, bipolar disorder, diabetic neuropathy	No controlled studies.	-	21	-
Zonisamide (4)	Epilepsy	No controlled studies.	-	2	-
Oxcarbazepine (4)	Epilepsy	No controlled studies.	-	7	-
Phenytoin (4)	Epilepsy	No controlled studies.	-	84	-
Riluzole (4)	amyotrophic lateral sclerosis	Ongoing placebo-controlled trial for reduction of AF episodes in patients with paroxysmal AF [140].	Ongoing RCT	1	1
Primidone (4)	Epilepsy & Tremor	No controlled studies.	-	4	-
Gabapentin (4)	Epilepsy, & neuropathic pain,	No controlled studies.	-	10	-
Pregabalin (4)	Epilepsy, neuropathic pain, & anxiety	No controlled studies.	-	8	-
Sertindole (4)	Anti-Psychotic (Withdrawn)	No controlled studies. Withdrawn due to cardiac toxicity.	-	1	-
Ergoloid (4)	Cognitive Impairment	No controlled studies.	-	1	-
Ergotamine (4)	Migraine, Cluster Headaches	No controlled studies.	-	5	-
Cocaine (4)	Local anaesthetic	Cocaine is associated with increased incidence of AF (HR=1.61, 95%CI 1.55-1.68) [141].	Observational	22	1
Methamphetamine (4)	ADHD, Obesity	Methamphetamine is associated with increased incidence of AF (HR=1.86, 95%CI 1.81-1.91) [141].	Observational	7	-
Mepivacaine (4)	Local anaesthetic	No controlled studies.	-	10	-
Ropivacaine (4)	Local anaesthetic	Ongoing placebo-controlled trial for reduction of post-cardiac surgery AF [142].	Ongoing RCT	14	2
Tetracaine (4)	Local anaesthetic	No controlled studies.	-	7	-
Procaine (4)	Local anaesthetic	No controlled studies.	-	57	-
Rocuronium (4)	Neuromuscular blockade	No controlled studies.	-	12	0
Vecuronium (4)	Neuromuscular blockade	No controlled studies.	-	7	0
Suxamethonium /	Neuromuscular blockade	No controlled studies.	-	9	1

Succinylcholine (4)					
Pancuronium (4)	Neuromuscular blockade	No controlled studies.	-	9	-
Pipecuronium (4)	Neuromuscular blockade	No controlled studies.	-	-	1
Atracurium (4)	Neuromuscular blockade	No controlled studies.	-	2	1
Cisatracurium (4)	Neuromuscular blockade	No controlled studies. Animal studies show a dose-dependent suppression of AF and atrial action potential shortening accompanying vagus nerve stimulation [143].	-	3	0
Mivacurium (4)	Neuromuscular blockade	No controlled studies. Animal studies show that similarly to cisatracuirum, mivacurium selectively targets the atrium without altering sinus and atrioventricular nodal function, which suggests a potentially selective mechanism for the suppression of AF [144].	-	1	0
Palifermin (4)	Mucositis	No controlled studies.	-	2	-
Aminophylline (4)	Asthma, & COPD	No controlled studies. Thought to induce AF in toxic doses. [145,146].	-	22	1
Theophylline (4)	Asthma	In the UK primary care dataset, use of theophylline was associated with potential increase in the risk of AF (RR=1.8; 95%CI 0.9-3.7) [147].<br>In the THEOPACE trial, use of theophylline in sick sinus syndrome patients did not lead to an increase in paroxysmal tachycardia or development of chronic AF [148].	Observational<br>RCT	57	-
Roflumilast (4)	COPD	In a meta-analysis of 6 trials, AF was more common with roflumilast compared with placebo (0.4% vs 0.2%, P = 0.02) [149]	Systematic Review	2	-
Beclomethasone (4)	COPD	In the TRIBUTE trial there were no AF events in the beclomethasone group, and 1 AF episode was observed in controls [150].	RCT	2	-
Betamethasone (4)	Eczema, dermatitis, psoriasis, among others	No controlled studies.	-	2	-
Budesonide (4)	Asthma, COPD, among others	No controlled studies.	-	2	-
Cortisone (4)	Asthma, Dermatomyositis, SLE, among others.	No controlled studies.	-	5	-
Dexamethasone (4)	Inflammatory and Allergic disorders, Cerebral edema, Congenital adrenal hyperplasia, etc.	Intra-operative DXM (1mg/kg) vs. placebo in 4494 adult patients undergoing cardiac surgery: no protective effect on the occurrence of AF (33.1% DXM vs 35.2% placebo; RR=0.94, 95%CI 0.87-1.02) [151].<br>Two additional trials showed no benefit [152,153]. The lack of benefit is contradicted by two trials of DXM given before and after CABG to prevent AF [154–156].<br>A meta analysis of 4 trials in patients going for cardiac surgery demonstrated reduced AF risk (RR = 0.69 95% CI 0.53-0.90)[157]<br>Incidence of AF or atrial flutter within the first 3M post-ablation was 29.0% in the single administration DXM group versus 24.5% in controls (P=0.80) [158].<br>Preoperative administration of DXM 8-12mg did not affect the incidence of AF after pneumonectomy [159].	RCTs<br>Systematic Review<br>Observational	63	2
Fluticasone (4)	Asthma, allergic rhinitis & inflammatory skin disorders	No controlled studies.	-	2	-
Hydrocortisone (4)	Acute hypersensitivity reactions, Adrenal insufficiency, Asthma, Inflammatory disease, etc.	Hydrocortisone + bisoprolol significantly reduced post-CABG AF compared to bisoprolol only (15.1% vs. 23.9%;P=0.02) [160].<br>A meta analysis of 2 trials in patients going for cardiac surgery demonstrated no reduction in AF risk (RR = 0.77 95%CI 0.41-1.42)[157]<br>A meta-analysis suggested that hydrocortisone 200-1000 mg/day reduced the risk of AF following surgery, with no effect on morbidity or mortality [161].<br>Ongoing trial of dexmedetomidine + hydrocortisone vs standard management to prevent post-operative AF [162].	RCT<br>Systematic Reviews<br>Ongoing RCT	59	5
Hydrocortamate (4)	Dermatoses	No controlled studies.	-	-	5
Medrysone (4)	Eye inflammation	No controlled studies.	-	-	1
Methylprednisolone / Prednisolone (4)	Inflammatory & Allergic diseases, graft rejection, etc.	Methylprednisolone (vs. placebo) significantly reduced AF relapse within the first 6 weeks and need of early reintervention following catheter ablation [163].<br>A meta analysis of 8 trials in patients going for cardiac surgery demonstrated no reduction in AF risk (RR = 0.71 95%CI 0.54-0.94)[157]	RCT<br>Systematic Review	89	2
Prednisone / Meprednisone (4)	Inflammatory and Allergic diseases, thrombocytopenia, etc.	Prednisone (given 2 days before, on the day of procedure and the day after) for reduction of AF relapse following ablation showed no benefit vs placebo [164].	RCT	74	2

Fluprednisolone (4)	Inflammatory and Allergic diseases, thrombocytopenia, etc.	No controlled studies.	-	1	-
Prednicarbate (4)	Inflammatory & Allergic skin conditions	No controlled studies.	-	-	1
Triamcinolone (4)	Inflammatory and Allergic conditions	No controlled studies.	-	6	1
Levothyroxine/T4 (4)	Hypothyroidism	Among 183,360 older adults treated with levothyroxine, high and medium-dose exposure was associated with increased risk of AF (OR=1.29, 95%CI 1.23-1.35 & OR=1.08, 95%CI 1.04-1.11, respectively, vs low-dose) [165].	Observational	365	2
Liothyronine/T3 (4)	Hypothyroidism	A tendency for an increased incidence of AF during the first 48h was observed in the Liothyronine group (19% vs. 5% in the placebo group, p = 0.13) [166].<br>A systematic review of RCTs showed no detrimental effect of short duration thyronine therapy in patients undergoing cardiac surgery [167].	RCT<br><br>Systematic review	191	8
Dextrothyroxine (4)	Hypothyroidism	No controlled studies.	-	1	0
Teprotumumab (4)	Graves' Ophtalmopathy	No controlled studies.	-	1	0
Tocilizumab (4)	RA, GCA, COVID-19	No controlled studies.	-	9	-
Natalizumab (4)	RA, IBD	No controlled studies	-	1	-
Tosedostat (2)	Leukaemia, Pancreatic cancer, MDS (EMA only)	Occurrence of AF was more common in the Tosedostat arm (18% vs 4% in cycle I of Tosedostat 120mg; 9% vs 5% in cycle II) [168].	RCT	2	-
Afatinib (4)	Non-small cell lung cancer	No controlled studies.	-	3	-
Vandetanib (4)	Medullary thyroid cancer	No controlled studies.	-	2	-
Pazopanib (4)	Renal cell carcinoma & Soft-tissue sarcoma	No controlled studies.	-	3	-
Regorafenib (4)	Colorectal cancer, GIST & hepatocellular carcinoma	A pharmacovigilance study bases on data from the USA FDA Adverse Events Reporting System demonstrated rate of reporting and proportional reporting ratios for AF (ROR=1.36 95% CI 1.03-1.81; PRR=1.36 χ^2=4.6) [169].	Observational	2	-
UCN-01 (2)	Cancer (not yet FDA or EMA approved)	No controlled studies.	-	2	0
Alectinib (4)	Lung cancer	No controlled studies.	-	1	-
Ceritinib (4)	Lung cancer	No controlled studies.	-	1	-
Crizotinib (4)	Lung cancer	No controlled studies.	-	4	-
Cabozantinib (4)	Medullary thyroid cancer, renal cell carcinoma & hepatocellular carcinoma	No controlled studies.	-	2	-
Dasatinib (4)	CML, ALL	No controlled studies.. Mouse models using dasatinib and quercetin to induce aging demonstrated greater atrial fibrosis and burst-pace induced AF compared with controls [170].	-	3	-
Sorafenib (4)	Renal cell carcinoma & hepatocellular carcinoma	A pharmacovigilance study bases on data from the USA FDA Adverse Events Reporting System demonstrated increased rate of reporting and proportional reporting ratios for AF (ROR=7.33 95% CI 6.88-7.82; PRR=6.97 χ^2=5155.99) [169].	Observational	2	-
Trastuzumab (4)	Breast cancer, & gastric cancer	Propensity-matched data from the Taiwan National Health Insurance database suggest that patients treated with Trastuzumab did not have inferior AF-free survival compared with controls (P =0.13), nor was treatment associated with an increased risk of AF (HR = 0.759 95 CI 0.649–0.889, P = 0.0006) [171].<br>Increased rate of AF following early-stage breast cancer does not seem to be associated with the use of Trastuzumab [172].	Observational	18	-
Ixazomib (4)	Multiple Myeloma	Increase in rate of reporting (OR=1.9, 95% CI 1.5-2.3) in the USA FDA Adverse Events Reporting System [173].	Observational	1	-
Carfilzomib (4)	Multiple Myeloma	Increased proportional reporting ratio (PRR=1.53, 95% CI 1.33-1.77) in the USA FDA Adverse Events Reporting System [174].	Observational	1	-
Bortezomib (4)	Multiple Myeloma	Increased proportional reporting ratio (PRR=1.65, 95% CI 1.52-1.79) in the USA FDA Adverse Events Reporting System [110], and in the WHO pharmacovigilance system (ROR=1.41, 95%CI 1.15-1.74) [175].	Observational	13	-
Elotuzumab (4)	Multiple Myeloma	Retrospective pharmacovigilance analysis of FAERS database for drugs reporting AF as AE found ROR for elotuzumab to be highest at 5.8 (95% CI 4-4 - 4.7) [173].	Observational	1	-

Polatuzumab Vedotin (4)	Diffuse Large B cell Lymphoma	No controlled studies.	-	1	-
Cabazitaxel (4)	Prostate cancer	No controlled studies.	-	1	-
Docetaxel (4)	Breast cancer, Lung cancer, Gastric cancer, Prostate cancer, Head & Neck cancer	Pharmacovigilance study using data from the WHO VigiBase demonstrate significant disproportionate reporting of AF for Docetaxel (ROR 1.37, 95% CI 1.09-1.73) [175].	Observational	9	-
Paclitaxel (4)	Ovarian cancer, Breast cancer, Pancreatic cancer & Kaposi sarcoma	Suggestion of mildly increased proportional reporting ratio (PRR=1.08) in the USA FDA Adverse Events Reporting System, with the result being driven by an increase in PRR in women (PRR=1.59, 95%CI 1.44–1.76) [174].	-	29	-
Mycophenolate Mofetil (4)	Cardiac, Renal & Hepatic transplantation	No controlled studies.	-	5	-
Midostaurin (4)	AML & Mastocytosis	Increase in reporting OR in the WHO pharmacovigilance database (ROR=3.76, 95%CI 1.50-9.39) [175].	Observational	- *	-
Vinblastine (4)	Leukaemias, Lymphomas & solid tumours (e.g. Breast and Lung cancer)	No controlled studies.	-	1	-
Vinorelbine (4)	Breast cancer & Lung cancer	Phase II study with Gemcitabine vs Gemcitabine and Vinorelbine. 4 patients (8.2%) treated with Gemcitabine and Vinorelbine developed AF/atrial flutter (one of these patients already had controlled AF), vs 0% in the Gemcitabine group [176].	RCT	5	-
Vincristine (4)	Lymphomas & solid tumours (e.g. Breast and Lung cancer)	No controlled studies.	-	13	-
Verubulin (2)	Multiple cancer types	No controlled studies.	-	1	-
Cyproterone (4)	Prostate cancer, hypersexuality in ♂, acne or hirsutism in ♀	No controlled studies.	-	-	4
Abiraterone (4)	Prostate cancer	In a combined analysis of the French pharmacovigilance database, the European pharmacovigilance database and the international pharmacovigilance database VigiBase the reporting OR was 3.2 to 4.1 times higher when compared to enzalutamide or other drugs [177].<br>Increase in reporting OR in the WHO pharmacovigilance database (ROR=1.84, 95%CI 1.35-2.50) [175].	Observational	10	1
Raloxifene (4)	Post-menopausal osteoporosis, Breast cancer	On Taiwan's National Health Insurance database, Raloxifene was not associated with risk of AF when compared with Alendronate (HR=0.94, 95%CI 0.76-1.18) [178].	Observational	5	0
Conjugated Estrogens (4)	Menopausal symptoms	Incident AF was more frequent in patients with previous hysterectomy and treated with conjugated equine estrogens vs. placebo (HR=1.17, CI95% 1.00-1.36) [179].<br>An analysis of the Taiwanese National Health Insurance Research Dataset showed that patients treated with conjugated equine estrogens had significantly higher incidence of AF when compared with patients treated with estradiol, (HR=1.96, 95%CI 1.03-3.73) [180].	RCT<br>Observational	5	0
Diethylstilbestrol (4)	Breast & Prostate cancer	No controlled studies.	-	1	4
MK-6913 (2)	Estrogen receptor agonist<br>Not approved	No controlled studies	-	-	1
Apremilast (4)	Psoriasis Vulgaris	No controlled studies.	-	2	-
Colchicine (4)	Gout, Pericarditis, FMF	Pooled data from 6 RCTs suggested that Colchicine is protective for post-cardiac surgery AF (RR=0.70; 95%CI 0.58-0.84; P= 0.0001).<br>Pooling of two post-AF ablation studies (one RCT and one controlled study) also showed protective effect up to 12 months (RR, 0.59; 95% CI, 0.43-0.81, P = 0.0011). No significant benefit was found for preventing AF in patients with coronary artery disease from 2 RCTs (pooled RR, 0.86; 95% CI, 0.69-1.06, P = 0.1628). [181]	Systematic Review	128	17
Cicloheximide (NA)	None	Utilized in cell culture studies on atrial fibrosis [182] and protein instability-induced AF [183].	-	2	-
Ribavirin (4)	RSV, Hepatitis C & VHF	No controlled studies.	-	4	-
Ocriplasmin (4)	Vitreomacular Traction	No controlled studies, but suggested as potential target drug for AF [184].	-	1	-
Carteolol (4)	Glaucoma	No controlled studies.	-	2	-
Metipranolol (4)	Glaucoma (Withdrawn)	No controlled studies. Withdrawn in UK due to uveitis.	-	1	-
Phenylpropanolamine (4)	Nasal Decongestant, Weight Loss (Withdrawn)	No controlled studies. Withdrawn in US due to risk of haemorrhagic stroke	-	8	-

Naphazoline (4)	Nasal Congestion, Ocular Inflammation	No controlled studies.	-	-	1
Oxymetazoline (4)	Nasal Congestion, Ocular Inflammation, Rosacea related Inflammation	No controlled studies.	-	1	2
Bethanechol (4)	Urinary retention	No controlled studies. Known to cause AF [185].	-	2	-
Darifenacin (4)	Urinary frequency, urgency & incontinence	No controlled studies. May block vagal-induced or programmed stimulation-induced AF in mice [186].	-	2	-
Oxybutynin (4)	Urinary frequency, urgency & incontinence	No controlled studies.	-	1	-
Propantheline (4)	Adult enuresis, hyperhidrosis, & gastrointestinal spasm	No controlled studies.	-	1	-
Solifenacin (4)	Overactive Bladder	No controlled studies.	-	1	-
Tolterodine (4)	Overactive Bladder	No controlled studies.	-	2	-
Tamsulosin (4)	BPH, Chronic Prostatitis	No controlled studies.	-	1	-
Citalopram (4)	Depression, anxiety	No controlled studies.	-	12	-
Escitalopram (4)	Depression, anxiety	No controlled studies.	-	4	-
Sertraline (4)	Depression, anxiety	No controlled studies.	-	4	-
Paroxetine (4)	Depression, anxiety	No controlled studies.	-	6	-
Fluoxetine (4)	Depression, anxiety	No controlled studies.	-	11	-
Duloxetine (4)	Depression, anxiety, pain, stress urinary incontinence	One study where duloxetine was compared with placebo for diabetic neuropathic pain reported 1 case of AF (0.9%) with duloxetine with 0% for placebo. (Taken from systematic review for duloxetine and cardiovascular events) [187]	RCT	2	-
Venlafaxine (4)	Depression, Anxiety	No controlled studies.	-	3	-
Trazodone (4)	Depression	No controlled studies.	-	5	-
Imipramine (4)	Depression	No controlled studies.	-	8	-
Fluvoxamine (4)	Depression, OCD	No controlled studies.	-	5	-
Clomipramine (4)	OCD	No controlled studies.	-	2	-
Amitryptiline (4)	Depression, neuropathic pain, migraine, tension headache	No controlled studies.	-	5	-
Protryptiline (4)	Depression	No controlled studies.	-	1	-
Nortryptiline (4)	Depression	No controlled studies.	-	1	-
Nomifensine (4)	Depression, Anxiety	Patients randomised to nomifensine or maprotiline received 10h ambulatory ECG monitoring at various intervals of treatment, no atrial arrhythmias were recorded in either arm [188].	RCT	1	-
Ramelteon (4)	Insomnia	No controlled studies.	-	1	-
Capamatinib (4)	Lung cancer	No controlled studies.	-	1	-
Melatonin (4)	Insomnia	One study evaluating the effect of melatonin vs placebo on AF and oxidative stress post CABG demonstrated no difference in AF incidence but a significant reduction in AF duration (P=0.01). Cannot obtain full text [189].	RCT (not available)	12	1 (withdrawn due to lack of staffing)

Legend: Phase – maximum clinical trial phase of development; GLP-1 – Glucagon Like Peptide 1; MESA – Multi-Ethnic Study of Atherosclerosis; RA – Rheumatoid Arthritis; IBD – Inflammatory Bowel Disease; GCA – Giant Cell Arteritis; MDS – Myelodysplastic Syndrome; DXM – Dexamethasone; TRIBUTE - Extrafine inhaled triple therapy versus dual bronchodilator therapy in chronic obstructive pulmonary disease; DECS - The DExamethasone for Cardiac Surgery; FIT-1/2 – Fracture Intervention Trial 1/2; FAERS – FDA Adverse Event Reporting System; PRR - Proportional Reporting Ratio; ROR – Reporting Odds Ratio; WHO ICSR – World Health Organisation Individual Case Safety Report; CAD – Coronary Artery Disease; RSV – Respiratory Syncytial Virus; VHF – Viral Haemorrhagic Fever; FMF – Familial Mediterranean Fever; BPH – Benign Prostatic Hypertrophy
* despite no results on Medline and clinicaltrials.gov, data identified from Alexandre et al. [175]

Supplementary Table 18 - Function description, subcellular locations and pathways for each prioritised target where drugs have good quality clinical evidence.

Target	Approved Name	Function Descriptions*	Subcellular Location	Target Class	Pathway
AOPEP	aminopeptidase O (putative)	Aminopeptidase which catalyzes the hydrolysis of amino acid residues from the N-terminus of peptide or protein substrates.	Nucleus, [Isoform 3]: Cytoplasm	Enzyme	Metabolism of Angiotensinogen to Angiotensins
ENPEP	glutamyl aminopeptidase	Regulates central hypertension through its calcium-modulated preference to cleave N-terminal acidic residues from peptides such as angiotensin II.	Cell membrane	Enzyme	Metabolism of Angiotensinogen to Angiotensins
HCN4	hyperpolarization activated cyclic nucleotide gated potassium channel 4	Hyperpolarization-activated ion channel with very slow activation and inactivation exhibiting weak selectivity for potassium over sodium ions. Contributes to the native pacemaker currents in heart (If) that regulate the rhythm of heart beat.	Cell membrane	Ion channel	HCN channels
NPR3	natriuretic peptide receptor 3	Receptor for the natriuretic peptide hormones, binding with similar affinities atrial natriuretic peptide NPPA/ANP, brain natriuretic peptide NPPB/BNP, and C-type natriuretic peptide NPPC/CNP. May function as a clearance receptor for NPPA, NPPB and NPPC, regulating their local concentrations and effects. May regulate diuresis, blood pressure and skeletal development. Does not have guanylate cyclase activity.)	Cell membrane	Membrane receptor, Enzyme	
PDE4B	phosphodiesterase 4B	Hydrolyzes the second messenger cAMP	[Isoform PDE4B5]: Cytoplasm, Cell membrane	Enzyme	DARPP-32 events
PDE3A	phosphodiesterase 3A	Cyclic nucleotide phosphodiesterase with specificity for the second messengers cAMP and cGMP, which are key regulators of many important physiological processes	Membrane, Cytoplasm	Enzyme	G alpha (s) signalling events
PSMD3	proteasome 26S subunit, non-ATPase 3	Component of the 26S proteasome, a multiprotein complex involved in the ATP-dependent degradation of ubiquitinated proteins. This complex plays a key role in the maintenance of protein homeostasis by removing misfolded or damaged proteins, which could impair cellular functions, and by removing proteins whose functions are no longer required.	Nucleoplasm	Enzyme	Hedgehog 'on' state
PSMD3	proteasome 26S subunit, non-ATPase 3	Component of the 26S proteasome, a multiprotein complex involved in the ATP-dependent degradation of ubiquitinated proteins. This complex plays a key role in the maintenance of protein homeostasis by removing misfolded or damaged proteins, which could impair cellular functions, and by removing proteins whose functions are no longer required.	Nucleoplasm	Enzyme	APC/C:Cdh1 mediated degradation of Cdc20 and other APC/C:Cdh1 targeted proteins in late mitosis/early G1
PSMD3	proteasome 26S subunit, non-ATPase 3	Component of the 26S proteasome, a multiprotein complex involved in the ATP-dependent degradation of ubiquitinated proteins. This complex plays a key role in the maintenance of protein homeostasis by removing misfolded or damaged proteins, which could impair cellular functions, and by removing proteins whose functions are no longer required.	Nucleoplasm	Enzyme	Ubiquitin-dependent degradation of Cyclin D
PSMD3	proteasome 26S subunit, non-ATPase 3	Component of the 26S proteasome, a multiprotein complex involved in the ATP-dependent degradation of ubiquitinated proteins. This complex plays a key role in the maintenance of protein homeostasis by removing misfolded or damaged proteins, which could impair cellular functions, and by removing proteins whose functions are no longer required.	Nucleoplasm	Enzyme	Regulation of expression of SLITs and ROBOs
PSMD3	proteasome 26S subunit, non-ATPase 3	Component of the 26S proteasome, a multiprotein complex involved in the ATP-dependent degradation of ubiquitinated proteins. This complex plays a key role in the maintenance of protein homeostasis by removing misfolded or damaged proteins, which could impair cellular functions, and by removing proteins whose functions are no longer required.	Nucleoplasm	Enzyme	Regulation of RUNX3 expression and activity
PSMD3	proteasome 26S subunit, non-ATPase 3	Component of the 26S proteasome, a multiprotein complex involved in the ATP-dependent degradation of ubiquitinated proteins. This complex plays a key role in the maintenance of protein homeostasis by removing misfolded or damaged proteins, which could impair cellular functions, and by removing proteins whose functions are no longer required.	Nucleoplasm	Enzyme	Degradation of AXIN

Target	Approved Name	Function Descriptions*	Subcellular Location	Target Class	Pathway
PSMD3	proteasome 26S subunit, non-ATPase 3	Component of the 26S proteasome, a multiprotein complex involved in the ATP-dependent degradation of ubiquitinated proteins. This complex plays a key role in the maintenance of protein homeostasis by removing misfolded or damaged proteins, which could impair cellular functions, and by removing proteins whose functions are no longer required.	Nucleoplasm	Enzyme	Antigen processing: Ubiquitination & Proteasome degradation
PSMD3	proteasome 26S subunit, non-ATPase 3	Component of the 26S proteasome, a multiprotein complex involved in the ATP-dependent degradation of ubiquitinated proteins. This complex plays a key role in the maintenance of protein homeostasis by removing misfolded or damaged proteins, which could impair cellular functions, and by removing proteins whose functions are no longer required.	Nucleoplasm	Enzyme	The role of GTSE1 in G2/M progression after G2 checkpoint
PSMD3	proteasome 26S subunit, non-ATPase 3	Component of the 26S proteasome, a multiprotein complex involved in the ATP-dependent degradation of ubiquitinated proteins. This complex plays a key role in the maintenance of protein homeostasis by removing misfolded or damaged proteins, which could impair cellular functions, and by removing proteins whose functions are no longer required.	Nucleoplasm	Enzyme	Regulation of PTEN stability and activity
PSMD3	proteasome 26S subunit, non-ATPase 3	Component of the 26S proteasome, a multiprotein complex involved in the ATP-dependent degradation of ubiquitinated proteins. This complex plays a key role in the maintenance of protein homeostasis by removing misfolded or damaged proteins, which could impair cellular functions, and by removing proteins whose functions are no longer required.	Nucleoplasm	Enzyme	G2/M Checkpoints
PSMD3	proteasome 26S subunit, non-ATPase 3	Component of the 26S proteasome, a multiprotein complex involved in the ATP-dependent degradation of ubiquitinated proteins. This complex plays a key role in the maintenance of protein homeostasis by removing misfolded or damaged proteins, which could impair cellular functions, and by removing proteins whose functions are no longer required.	Nucleoplasm	Enzyme	Ub-specific processing proteases
PSMD3	proteasome 26S subunit, non-ATPase 3	Component of the 26S proteasome, a multiprotein complex involved in the ATP-dependent degradation of ubiquitinated proteins. This complex plays a key role in the maintenance of protein homeostasis by removing misfolded or damaged proteins, which could impair cellular functions, and by removing proteins whose functions are no longer required.	Nucleoplasm	Enzyme	Regulation of RUNX2 expression and activity
PSMD3	proteasome 26S subunit, non-ATPase 3	Component of the 26S proteasome, a multiprotein complex involved in the ATP-dependent degradation of ubiquitinated proteins. This complex plays a key role in the maintenance of protein homeostasis by removing misfolded or damaged proteins, which could impair cellular functions, and by removing proteins whose functions are no longer required.	Nucleoplasm	Enzyme	SCF(Skp2)-mediated degradation of p27/p21
PSMD3	proteasome 26S subunit, non-ATPase 3	Component of the 26S proteasome, a multiprotein complex involved in the ATP-dependent degradation of ubiquitinated proteins. This complex plays a key role in the maintenance of protein homeostasis by removing misfolded or damaged proteins, which could impair cellular functions, and by removing proteins whose functions are no longer required.	Nucleoplasm	Enzyme	Vpu mediated degradation of CD4
PSMD3	proteasome 26S subunit, non-ATPase 3	Component of the 26S proteasome, a multiprotein complex involved in the ATP-dependent degradation of ubiquitinated proteins. This complex plays a key role in the maintenance of protein homeostasis by removing misfolded or damaged proteins, which could impair cellular functions, and by removing proteins whose functions are no longer required.	Nucleoplasm	Enzyme	KEAP1-NFE2L2 pathway
PSMD3	proteasome 26S subunit, non-ATPase 3	Component of the 26S proteasome, a multiprotein complex involved in the ATP-dependent degradation of ubiquitinated proteins. This complex plays a key role in the maintenance of protein homeostasis by removing misfolded or damaged proteins, which could impair cellular functions, and by removing proteins whose functions are no longer required.	Nucleoplasm	Enzyme	Oxygen-dependent proline hydroxylation of Hypoxia-inducible Factor Alpha

Target	Approved Name	Function Descriptions*	Subcellular Location	Target Class	Pathway
PSMD3	proteasome 26S subunit, non-ATPase 3	Component of the 26S proteasome, a multiprotein complex involved in the ATP-dependent degradation of ubiquitinated proteins. This complex plays a key role in the maintenance of protein homeostasis by removing misfolded or damaged proteins, which could impair cellular functions, and by removing proteins whose functions are no longer required.	Nucleoplasm	Enzyme	Degradation of GLI1 by the proteasome
PSMD3	proteasome 26S subunit, non-ATPase 3	Component of the 26S proteasome, a multiprotein complex involved in the ATP-dependent degradation of ubiquitinated proteins. This complex plays a key role in the maintenance of protein homeostasis by removing misfolded or damaged proteins, which could impair cellular functions, and by removing proteins whose functions are no longer required.	Nucleoplasm	Enzyme	Degradation of beta-catenin by the destruction complex
PSMD3	proteasome 26S subunit, non-ATPase 3	Component of the 26S proteasome, a multiprotein complex involved in the ATP-dependent degradation of ubiquitinated proteins. This complex plays a key role in the maintenance of protein homeostasis by removing misfolded or damaged proteins, which could impair cellular functions, and by removing proteins whose functions are no longer required.	Nucleoplasm	Enzyme	MAPK6/MAPK4 signaling
PSMD3	proteasome 26S subunit, non-ATPase 3	Component of the 26S proteasome, a multiprotein complex involved in the ATP-dependent degradation of ubiquitinated proteins. This complex plays a key role in the maintenance of protein homeostasis by removing misfolded or damaged proteins, which could impair cellular functions, and by removing proteins whose functions are no longer required.	Nucleoplasm	Enzyme	Regulation of RAS by GAPs
PSMD3	proteasome 26S subunit, non-ATPase 3	Component of the 26S proteasome, a multiprotein complex involved in the ATP-dependent degradation of ubiquitinated proteins. This complex plays a key role in the maintenance of protein homeostasis by removing misfolded or damaged proteins, which could impair cellular functions, and by removing proteins whose functions are no longer required.	Nucleoplasm	Enzyme	Autodegradation of Cdh1 by Cdh1:APC/C
PSMD3	proteasome 26S subunit, non-ATPase 3	Component of the 26S proteasome, a multiprotein complex involved in the ATP-dependent degradation of ubiquitinated proteins. This complex plays a key role in the maintenance of protein homeostasis by removing misfolded or damaged proteins, which could impair cellular functions, and by removing proteins whose functions are no longer required.	Nucleoplasm	Enzyme	Interleukin-1 signaling
PSMD3	proteasome 26S subunit, non-ATPase 3	Component of the 26S proteasome, a multiprotein complex involved in the ATP-dependent degradation of ubiquitinated proteins. This complex plays a key role in the maintenance of protein homeostasis by removing misfolded or damaged proteins, which could impair cellular functions, and by removing proteins whose functions are no longer required.	Nucleoplasm	Enzyme	AUF1 (hnRNP D0) binds and destabilizes mRNA
PSMD3	proteasome 26S subunit, non-ATPase 3	Component of the 26S proteasome, a multiprotein complex involved in the ATP-dependent degradation of ubiquitinated proteins. This complex plays a key role in the maintenance of protein homeostasis by removing misfolded or damaged proteins, which could impair cellular functions, and by removing proteins whose functions are no longer required.	Nucleoplasm	Enzyme	Ubiquitin Mediated Degradation of Phosphorylated Cdc25A
PSMD3	proteasome 26S subunit, non-ATPase 3	Component of the 26S proteasome, a multiprotein complex involved in the ATP-dependent degradation of ubiquitinated proteins. This complex plays a key role in the maintenance of protein homeostasis by removing misfolded or damaged proteins, which could impair cellular functions, and by removing proteins whose functions are no longer required.	Nucleoplasm	Enzyme	Downstream TCR signaling
PSMD3	proteasome 26S subunit, non-ATPase 3	Component of the 26S proteasome, a multiprotein complex involved in the ATP-dependent degradation of ubiquitinated proteins. This complex plays a key role in the maintenance of protein homeostasis by removing misfolded or damaged proteins, which could impair cellular functions, and by removing proteins whose functions are no longer required.	Nucleoplasm	Enzyme	Hedgehog ligand biogenesis

Target	Approved Name	Function Descriptions*	Subcellular Location	Target Class	Pathway
PSMD3	proteasome 26S subunit, non-ATPase 3	Component of the 26S proteasome, a multiprotein complex involved in the ATP-dependent degradation of ubiquitinated proteins. This complex plays a key role in the maintenance of protein homeostasis by removing misfolded or damaged proteins, which could impair cellular functions, and by removing proteins whose functions are no longer required.	Nucleoplasm	Enzyme	Autodegradation of the E3 ubiquitin ligase COP1
PSMD3	proteasome 26S subunit, non-ATPase 3	Component of the 26S proteasome, a multiprotein complex involved in the ATP-dependent degradation of ubiquitinated proteins. This complex plays a key role in the maintenance of protein homeostasis by removing misfolded or damaged proteins, which could impair cellular functions, and by removing proteins whose functions are no longer required.	Nucleoplasm	Enzyme	APC/C:Cdc20 mediated degradation of Securin
PSMD3	proteasome 26S subunit, non-ATPase 3	Component of the 26S proteasome, a multiprotein complex involved in the ATP-dependent degradation of ubiquitinated proteins. This complex plays a key role in the maintenance of protein homeostasis by removing misfolded or damaged proteins, which could impair cellular functions, and by removing proteins whose functions are no longer required.	Nucleoplasm	Enzyme	ABC-family proteins mediated transport
PSMD3	proteasome 26S subunit, non-ATPase 3	Component of the 26S proteasome, a multiprotein complex involved in the ATP-dependent degradation of ubiquitinated proteins. This complex plays a key role in the maintenance of protein homeostasis by removing misfolded or damaged proteins, which could impair cellular functions, and by removing proteins whose functions are no longer required.	Nucleoplasm	Enzyme	Cdc20:Phospho-APC/C mediated degradation of Cyclin A
PSMD3	proteasome 26S subunit, non-ATPase 3	Component of the 26S proteasome, a multiprotein complex involved in the ATP-dependent degradation of ubiquitinated proteins. This complex plays a key role in the maintenance of protein homeostasis by removing misfolded or damaged proteins, which could impair cellular functions, and by removing proteins whose functions are no longer required.	Nucleoplasm	Enzyme	Regulation of activated PAK-2p34 by proteasome mediated degradation
PSMD3	proteasome 26S subunit, non-ATPase 3	Component of the 26S proteasome, a multiprotein complex involved in the ATP-dependent degradation of ubiquitinated proteins. This complex plays a key role in the maintenance of protein homeostasis by removing misfolded or damaged proteins, which could impair cellular functions, and by removing proteins whose functions are no longer required.	Nucleoplasm	Enzyme	Assembly of the pre-replicative complex
PSMD3	proteasome 26S subunit, non-ATPase 3	Component of the 26S proteasome, a multiprotein complex involved in the ATP-dependent degradation of ubiquitinated proteins. This complex plays a key role in the maintenance of protein homeostasis by removing misfolded or damaged proteins, which could impair cellular functions, and by removing proteins whose functions are no longer required.	Nucleoplasm	Enzyme	Activation of NF-kappaB in B cells
PSMD3	proteasome 26S subunit, non-ATPase 3	Component of the 26S proteasome, a multiprotein complex involved in the ATP-dependent degradation of ubiquitinated proteins. This complex plays a key role in the maintenance of protein homeostasis by removing misfolded or damaged proteins, which could impair cellular functions, and by removing proteins whose functions are no longer required.	Nucleoplasm	Enzyme	RUNX1 regulates transcription of genes involved in differentiation of HSCs
PSMD3	proteasome 26S subunit, non-ATPase 3	Component of the 26S proteasome, a multiprotein complex involved in the ATP-dependent degradation of ubiquitinated proteins. This complex plays a key role in the maintenance of protein homeostasis by removing misfolded or damaged proteins, which could impair cellular functions, and by removing proteins whose functions are no longer required.	Nucleoplasm	Enzyme	Cross-presentation of soluble exogenous antigens (endosomes)
PSMD3	proteasome 26S subunit, non-ATPase 3	Component of the 26S proteasome, a multiprotein complex involved in the ATP-dependent degradation of ubiquitinated proteins. This complex plays a key role in the maintenance of protein homeostasis by removing misfolded or damaged proteins, which could impair cellular functions, and by removing proteins whose functions are no longer required.	Nucleoplasm	Enzyme	Defective CFTR causes cystic fibrosis

Target	Approved Name	Function Descriptions*	Subcellular Location	Target Class	Pathway
PSMD3	proteasome 26S subunit, non-ATPase 3	Component of the 26S proteasome, a multiprotein complex involved in the ATP-dependent degradation of ubiquitinated proteins. This complex plays a key role in the maintenance of protein homeostasis by removing misfolded or damaged proteins, which could impair cellular functions, and by removing proteins whose functions are no longer required.	Nucleoplasm	Enzyme	Asymmetric localization of PCP proteins
PSMD3	proteasome 26S subunit, non-ATPase 3	Component of the 26S proteasome, a multiprotein complex involved in the ATP-dependent degradation of ubiquitinated proteins. This complex plays a key role in the maintenance of protein homeostasis by removing misfolded or damaged proteins, which could impair cellular functions, and by removing proteins whose functions are no longer required.	Nucleoplasm	Enzyme	UCH proteinases
PSMD3	proteasome 26S subunit, non-ATPase 3	Component of the 26S proteasome, a multiprotein complex involved in the ATP-dependent degradation of ubiquitinated proteins. This complex plays a key role in the maintenance of protein homeostasis by removing misfolded or damaged proteins, which could impair cellular functions, and by removing proteins whose functions are no longer required.	Nucleoplasm	Enzyme	ER-Phagosome pathway
PSMD3	proteasome 26S subunit, non-ATPase 3	Component of the 26S proteasome, a multiprotein complex involved in the ATP-dependent degradation of ubiquitinated proteins. This complex plays a key role in the maintenance of protein homeostasis by removing misfolded or damaged proteins, which could impair cellular functions, and by removing proteins whose functions are no longer required.	Nucleoplasm	Enzyme	Separation of Sister Chromatids
PSMD3	proteasome 26S subunit, non-ATPase 3	Component of the 26S proteasome, a multiprotein complex involved in the ATP-dependent degradation of ubiquitinated proteins. This complex plays a key role in the maintenance of protein homeostasis by removing misfolded or damaged proteins, which could impair cellular functions, and by removing proteins whose functions are no longer required.	Nucleoplasm	Enzyme	Negative regulation of NOTCH4 signaling
PSMD3	proteasome 26S subunit, non-ATPase 3	Component of the 26S proteasome, a multiprotein complex involved in the ATP-dependent degradation of ubiquitinated proteins. This complex plays a key role in the maintenance of protein homeostasis by removing misfolded or damaged proteins, which could impair cellular functions, and by removing proteins whose functions are no longer required.	Nucleoplasm	Enzyme	Orc1 removal from chromatin
PSMD3	proteasome 26S subunit, non-ATPase 3	Component of the 26S proteasome, a multiprotein complex involved in the ATP-dependent degradation of ubiquitinated proteins. This complex plays a key role in the maintenance of protein homeostasis by removing misfolded or damaged proteins, which could impair cellular functions, and by removing proteins whose functions are no longer required.	Nucleoplasm	Enzyme	GSK3B and BTRC:CUL1-mediated-degradation of NFE2L2
PSMD3	proteasome 26S subunit, non-ATPase 3	Component of the 26S proteasome, a multiprotein complex involved in the ATP-dependent degradation of ubiquitinated proteins. This complex plays a key role in the maintenance of protein homeostasis by removing misfolded or damaged proteins, which could impair cellular functions, and by removing proteins whose functions are no longer required.	Nucleoplasm	Enzyme	Degradation of GLI2 by the proteasome
PSMD3	proteasome 26S subunit, non-ATPase 3	Component of the 26S proteasome, a multiprotein complex involved in the ATP-dependent degradation of ubiquitinated proteins. This complex plays a key role in the maintenance of protein homeostasis by removing misfolded or damaged proteins, which could impair cellular functions, and by removing proteins whose functions are no longer required.	Nucleoplasm	Enzyme	CDK-mediated phosphorylation and removal of Cdc6
PSMD3	proteasome 26S subunit, non-ATPase 3	Component of the 26S proteasome, a multiprotein complex involved in the ATP-dependent degradation of ubiquitinated proteins. This complex plays a key role in the maintenance of protein homeostasis by removing misfolded or damaged proteins, which could impair cellular functions, and by removing proteins whose functions are no longer required.	Nucleoplasm	Enzyme	FBXL7 down-regulates AURKA during mitotic entry and in early mitosis

Target	Approved Name	Function Descriptions*	Subcellular Location	Target Class	Pathway
PSMD3	proteasome 26S subunit, non-ATPase 3	Component of the 26S proteasome, a multiprotein complex involved in the ATP-dependent degradation of ubiquitinated proteins. This complex plays a key role in the maintenance of protein homeostasis by removing misfolded or damaged proteins, which could impair cellular functions, and by removing proteins whose functions are no longer required.	Nucleoplasm	Enzyme	CLEC7A (Dectin-1) signaling
PSMD3	proteasome 26S subunit, non-ATPase 3	Component of the 26S proteasome, a multiprotein complex involved in the ATP-dependent degradation of ubiquitinated proteins. This complex plays a key role in the maintenance of protein homeostasis by removing misfolded or damaged proteins, which could impair cellular functions, and by removing proteins whose functions are no longer required.	Nucleoplasm	Enzyme	Hh mutants are degraded by ERAD
PSMD3	proteasome 26S subunit, non-ATPase 3	Component of the 26S proteasome, a multiprotein complex involved in the ATP-dependent degradation of ubiquitinated proteins. This complex plays a key role in the maintenance of protein homeostasis by removing misfolded or damaged proteins, which could impair cellular functions, and by removing proteins whose functions are no longer required.	Nucleoplasm	Enzyme	GLI3 is processed to GLI3R by the proteasome
PSMD3	proteasome 26S subunit, non-ATPase 3	Component of the 26S proteasome, a multiprotein complex involved in the ATP-dependent degradation of ubiquitinated proteins. This complex plays a key role in the maintenance of protein homeostasis by removing misfolded or damaged proteins, which could impair cellular functions, and by removing proteins whose functions are no longer required.	Nucleoplasm	Enzyme	Degradation of DVL
PSMD3	proteasome 26S subunit, non-ATPase 3	Component of the 26S proteasome, a multiprotein complex involved in the ATP-dependent degradation of ubiquitinated proteins. This complex plays a key role in the maintenance of protein homeostasis by removing misfolded or damaged proteins, which could impair cellular functions, and by removing proteins whose functions are no longer required.	Nucleoplasm	Enzyme	Neutrophil degranulation
PSMD3	proteasome 26S subunit, non-ATPase 3	Component of the 26S proteasome, a multiprotein complex involved in the ATP-dependent degradation of ubiquitinated proteins. This complex plays a key role in the maintenance of protein homeostasis by removing misfolded or damaged proteins, which could impair cellular functions, and by removing proteins whose functions are no longer required.	Nucleoplasm	Enzyme	Regulation of ornithine decarboxylase (ODC)
PSMD3	proteasome 26S subunit, non-ATPase 3	Component of the 26S proteasome, a multiprotein complex involved in the ATP-dependent degradation of ubiquitinated proteins. This complex plays a key role in the maintenance of protein homeostasis by removing misfolded or damaged proteins, which could impair cellular functions, and by removing proteins whose functions are no longer required.	Nucleoplasm	Enzyme	SCF-beta-TrCP mediated degradation of Emi1
PSMD3	proteasome 26S subunit, non-ATPase 3	Component of the 26S proteasome, a multiprotein complex involved in the ATP-dependent degradation of ubiquitinated proteins. This complex plays a key role in the maintenance of protein homeostasis by removing misfolded or damaged proteins, which could impair cellular functions, and by removing proteins whose functions are no longer required.	Nucleoplasm	Enzyme	Neddylation
PSMD3	proteasome 26S subunit, non-ATPase 3	Component of the 26S proteasome, a multiprotein complex involved in the ATP-dependent degradation of ubiquitinated proteins. This complex plays a key role in the maintenance of protein homeostasis by removing misfolded or damaged proteins, which could impair cellular functions, and by removing proteins whose functions are no longer required.	Nucleoplasm	Enzyme	Vif-mediated degradation of APOBEC3G
PSMD3	proteasome 26S subunit, non-ATPase 3	Component of the 26S proteasome, a multiprotein complex involved in the ATP-dependent degradation of ubiquitinated proteins. This complex plays a key role in the maintenance of protein homeostasis by removing misfolded or damaged proteins, which could impair cellular functions, and by removing proteins whose functions are no longer required.	Nucleoplasm	Enzyme	Dectin-1 mediated noncanonical NF-kB signaling

Target	Approved Name	Function Descriptions*	Subcellular Location	Target Class	Pathway
PSMD3	proteasome 26S subunit, non-ATPase 3	Component of the 26S proteasome, a multiprotein complex involved in the ATP-dependent degradation of ubiquitinated proteins. This complex plays a key role in the maintenance of protein homeostasis by removing misfolded or damaged proteins, which could impair cellular functions, and by removing proteins whose functions are no longer required.	Nucleoplasm	Enzyme	FCERI mediated NF-kB activation
PSMD3	proteasome 26S subunit, non-ATPase 3	Component of the 26S proteasome, a multiprotein complex involved in the ATP-dependent degradation of ubiquitinated proteins. This complex plays a key role in the maintenance of protein homeostasis by removing misfolded or damaged proteins, which could impair cellular functions, and by removing proteins whose functions are no longer required.	Nucleoplasm	Enzyme	NIK-->noncanonical NF-kB signaling
PSMD3	proteasome 26S subunit, non-ATPase 3	Component of the 26S proteasome, a multiprotein complex involved in the ATP-dependent degradation of ubiquitinated proteins. This complex plays a key role in the maintenance of protein homeostasis by removing misfolded or damaged proteins, which could impair cellular functions, and by removing proteins whose functions are no longer required.	Nucleoplasm	Enzyme	TNFR2 non-canonical NF-kB pathway
PSMB7	proteasome 20S subunit beta 7	Component of the 20S core proteasome complex involved in the proteolytic degradation of most intracellular proteins. This complex plays numerous essential roles within the cell by associating with different regulatory particles.	Cytoplasm, Nucleus	Enzyme, Unclassified protein	Hedgehog 'on' state
PSMB7	proteasome 20S subunit beta 7	Component of the 20S core proteasome complex involved in the proteolytic degradation of most intracellular proteins. This complex plays numerous essential roles within the cell by associating with different regulatory particles.	Cytoplasm, Nucleus	Enzyme, Unclassified protein	APC/C:Cdh1 mediated degradation of Cdc20 and other APC/C:Cdh1 targeted proteins in late mitosis/early G1
PSMB7	proteasome 20S subunit beta 7	Component of the 20S core proteasome complex involved in the proteolytic degradation of most intracellular proteins. This complex plays numerous essential roles within the cell by associating with different regulatory particles.	Cytoplasm, Nucleus	Enzyme, Unclassified protein	Ubiquitin-dependent degradation of Cyclin D
PSMB7	proteasome 20S subunit beta 7	Component of the 20S core proteasome complex involved in the proteolytic degradation of most intracellular proteins. This complex plays numerous essential roles within the cell by associating with different regulatory particles.	Cytoplasm, Nucleus	Enzyme, Unclassified protein	Regulation of expression of SLITs and ROBOs
PSMB7	proteasome 20S subunit beta 7	Component of the 20S core proteasome complex involved in the proteolytic degradation of most intracellular proteins. This complex plays numerous essential roles within the cell by associating with different regulatory particles.	Cytoplasm, Nucleus	Enzyme, Unclassified protein	Regulation of RUNX3 expression and activity
PSMB7	proteasome 20S subunit beta 7	Component of the 20S core proteasome complex involved in the proteolytic degradation of most intracellular proteins. This complex plays numerous essential roles within the cell by associating with different regulatory particles.	Cytoplasm, Nucleus	Enzyme, Unclassified protein	Degradation of AXIN
PSMB7	proteasome 20S subunit beta 7	Component of the 20S core proteasome complex involved in the proteolytic degradation of most intracellular proteins. This complex plays numerous essential roles within the cell by associating with different regulatory particles.	Cytoplasm, Nucleus	Enzyme, Unclassified protein	Antigen processing: Ubiquitination & Proteasome degradation
PSMB7	proteasome 20S subunit beta 7	Component of the 20S core proteasome complex involved in the proteolytic degradation of most intracellular proteins. This complex plays numerous essential roles within the cell by associating with different regulatory particles.	Cytoplasm, Nucleus	Enzyme, Unclassified protein	The role of GTSE1 in G2/M progression after G2 checkpoint
PSMB7	proteasome 20S subunit beta 7	Component of the 20S core proteasome complex involved in the proteolytic degradation of most intracellular proteins. This complex plays numerous essential roles within the cell by associating with different regulatory particles.	Cytoplasm, Nucleus	Enzyme, Unclassified protein	Regulation of PTEN stability and activity
PSMB7	proteasome 20S subunit beta 7	Component of the 20S core proteasome complex involved in the proteolytic degradation of most intracellular proteins. This complex plays numerous essential roles within the cell by associating with different regulatory particles.	Cytoplasm, Nucleus	Enzyme, Unclassified protein	G2/M Checkpoints

Target	Approved Name	Function Descriptions*	Subcellular Location	Target Class	Pathway
PSMB7	proteasome 20S subunit beta 7	Component of the 20S core proteasome complex involved in the proteolytic degradation of most intracellular proteins. This complex plays numerous essential roles within the cell by associating with different regulatory particles.	Cytoplasm, Nucleus	Enzyme, Unclassified protein	Ub-specific processing proteases
PSMB7	proteasome 20S subunit beta 7	Component of the 20S core proteasome complex involved in the proteolytic degradation of most intracellular proteins. This complex plays numerous essential roles within the cell by associating with different regulatory particles.	Cytoplasm, Nucleus	Enzyme, Unclassified protein	Regulation of RUNX2 expression and activity
PSMB7	proteasome 20S subunit beta 7	Component of the 20S core proteasome complex involved in the proteolytic degradation of most intracellular proteins. This complex plays numerous essential roles within the cell by associating with different regulatory particles.	Cytoplasm, Nucleus	Enzyme, Unclassified protein	SCF(Skp2)-mediated degradation of p27/p21
PSMB7	proteasome 20S subunit beta 7	Component of the 20S core proteasome complex involved in the proteolytic degradation of most intracellular proteins. This complex plays numerous essential roles within the cell by associating with different regulatory particles.	Cytoplasm, Nucleus	Enzyme, Unclassified protein	Vpu mediated degradation of CD4
PSMB7	proteasome 20S subunit beta 7	Component of the 20S core proteasome complex involved in the proteolytic degradation of most intracellular proteins. This complex plays numerous essential roles within the cell by associating with different regulatory particles.	Cytoplasm, Nucleus	Enzyme, Unclassified protein	KEAP1-NFE2L2 pathway
PSMB7	proteasome 20S subunit beta 7	Component of the 20S core proteasome complex involved in the proteolytic degradation of most intracellular proteins. This complex plays numerous essential roles within the cell by associating with different regulatory particles.	Cytoplasm, Nucleus	Enzyme, Unclassified protein	Oxygen-dependent proline hydroxylation of Hypoxia-inducible Factor Alpha
PSMB7	proteasome 20S subunit beta 7	Component of the 20S core proteasome complex involved in the proteolytic degradation of most intracellular proteins. This complex plays numerous essential roles within the cell by associating with different regulatory particles.	Cytoplasm, Nucleus	Enzyme, Unclassified protein	Degradation of GLI1 by the proteasome
PSMB7	proteasome 20S subunit beta 7	Component of the 20S core proteasome complex involved in the proteolytic degradation of most intracellular proteins. This complex plays numerous essential roles within the cell by associating with different regulatory particles.	Cytoplasm, Nucleus	Enzyme, Unclassified protein	Degradation of beta-catenin by the destruction complex
PSMB7	proteasome 20S subunit beta 7	Component of the 20S core proteasome complex involved in the proteolytic degradation of most intracellular proteins. This complex plays numerous essential roles within the cell by associating with different regulatory particles.	Cytoplasm, Nucleus	Enzyme, Unclassified protein	MAPK6/MAPK4 signaling
PSMB7	proteasome 20S subunit beta 7	Component of the 20S core proteasome complex involved in the proteolytic degradation of most intracellular proteins. This complex plays numerous essential roles within the cell by associating with different regulatory particles.	Cytoplasm, Nucleus	Enzyme, Unclassified protein	Regulation of RAS by GAPs
PSMB7	proteasome 20S subunit beta 7	Component of the 20S core proteasome complex involved in the proteolytic degradation of most intracellular proteins. This complex plays numerous essential roles within the cell by associating with different regulatory particles.	Cytoplasm, Nucleus	Enzyme, Unclassified protein	Autodegradation of Cdh1 by Cdh1:APC/C
PSMB7	proteasome 20S subunit beta 7	Component of the 20S core proteasome complex involved in the proteolytic degradation of most intracellular proteins. This complex plays numerous essential roles within the cell by associating with different regulatory particles.	Cytoplasm, Nucleus	Enzyme, Unclassified protein	AUF1 (hnRNP D0) binds and destabilizes mRNA
PSMB7	proteasome 20S subunit beta 7	Component of the 20S core proteasome complex involved in the proteolytic degradation of most intracellular proteins. This complex plays numerous essential roles within the cell by associating with different regulatory particles.	Cytoplasm, Nucleus	Enzyme, Unclassified protein	Interleukin-1 signaling
PSMB7	proteasome 20S subunit beta 7	Component of the 20S core proteasome complex involved in the proteolytic degradation of most intracellular proteins. This complex plays numerous essential roles within the cell by associating with different regulatory particles.	Cytoplasm, Nucleus	Enzyme, Unclassified protein	Ubiquitin Mediated Degradation of Phosphorylated Cdc25A

Target	Approved Name	Function Descriptions*	Subcellular Location	Target Class	Pathway
PSMB7	proteasome 20S subunit beta 7	Component of the 20S core proteasome complex involved in the proteolytic degradation of most intracellular proteins. This complex plays numerous essential roles within the cell by associating with different regulatory particles.	Cytoplasm, Nucleus	Enzyme, Unclassified protein	Downstream TCR signaling
PSMB7	proteasome 20S subunit beta 7	Component of the 20S core proteasome complex involved in the proteolytic degradation of most intracellular proteins. This complex plays numerous essential roles within the cell by associating with different regulatory particles.	Cytoplasm, Nucleus	Enzyme, Unclassified protein	Hedgehog ligand biogenesis
PSMB7	proteasome 20S subunit beta 7	Component of the 20S core proteasome complex involved in the proteolytic degradation of most intracellular proteins. This complex plays numerous essential roles within the cell by associating with different regulatory particles.	Cytoplasm, Nucleus	Enzyme, Unclassified protein	Autodegradation of the E3 ubiquitin ligase COP1
PSMB7	proteasome 20S subunit beta 7	Component of the 20S core proteasome complex involved in the proteolytic degradation of most intracellular proteins. This complex plays numerous essential roles within the cell by associating with different regulatory particles.	Cytoplasm, Nucleus	Enzyme, Unclassified protein	APC/C:Cdc20 mediated degradation of Securin
PSMB7	proteasome 20S subunit beta 7	Component of the 20S core proteasome complex involved in the proteolytic degradation of most intracellular proteins. This complex plays numerous essential roles within the cell by associating with different regulatory particles.	Cytoplasm, Nucleus	Enzyme, Unclassified protein	ABC-family proteins mediated transport
PSMB7	proteasome 20S subunit beta 7	Component of the 20S core proteasome complex involved in the proteolytic degradation of most intracellular proteins. This complex plays numerous essential roles within the cell by associating with different regulatory particles.	Cytoplasm, Nucleus	Enzyme, Unclassified protein	Cdc20:Phospho-APC/C mediated degradation of Cyclin A
PSMB7	proteasome 20S subunit beta 7	Component of the 20S core proteasome complex involved in the proteolytic degradation of most intracellular proteins. This complex plays numerous essential roles within the cell by associating with different regulatory particles.	Cytoplasm, Nucleus	Enzyme, Unclassified protein	Regulation of activated PAK-2p34 by proteasome mediated degradation
PSMB7	proteasome 20S subunit beta 7	Component of the 20S core proteasome complex involved in the proteolytic degradation of most intracellular proteins. This complex plays numerous essential roles within the cell by associating with different regulatory particles.	Cytoplasm, Nucleus	Enzyme, Unclassified protein	Assembly of the pre-replicative complex
PSMB7	proteasome 20S subunit beta 7	Component of the 20S core proteasome complex involved in the proteolytic degradation of most intracellular proteins. This complex plays numerous essential roles within the cell by associating with different regulatory particles.	Cytoplasm, Nucleus	Enzyme, Unclassified protein	Activation of NF-kappaB in B cells
PSMB7	proteasome 20S subunit beta 7	Component of the 20S core proteasome complex involved in the proteolytic degradation of most intracellular proteins. This complex plays numerous essential roles within the cell by associating with different regulatory particles.	Cytoplasm, Nucleus	Enzyme, Unclassified protein	RUNX1 regulates transcription of genes involved in differentiation of HSCs
PSMB7	proteasome 20S subunit beta 7	Component of the 20S core proteasome complex involved in the proteolytic degradation of most intracellular proteins. This complex plays numerous essential roles within the cell by associating with different regulatory particles.	Cytoplasm, Nucleus	Enzyme, Unclassified protein	Cross-presentation of soluble exogenous antigens (endosomes)
PSMB7	proteasome 20S subunit beta 7	Component of the 20S core proteasome complex involved in the proteolytic degradation of most intracellular proteins. This complex plays numerous essential roles within the cell by associating with different regulatory particles.	Cytoplasm, Nucleus	Enzyme, Unclassified protein	Defective CFTR causes cystic fibrosis
PSMB7	proteasome 20S subunit beta 7	Component of the 20S core proteasome complex involved in the proteolytic degradation of most intracellular proteins. This complex plays numerous essential roles within the cell by associating with different regulatory particles.	Cytoplasm, Nucleus	Enzyme, Unclassified protein	Asymmetric localization of PCP proteins
PSMB7	proteasome 20S subunit beta 7	Component of the 20S core proteasome complex involved in the proteolytic degradation of most intracellular proteins. This complex plays numerous essential roles within the cell by associating with different regulatory particles.	Cytoplasm, Nucleus	Enzyme, Unclassified protein	UCH proteinases

Target	Approved Name	Function Descriptions*	Subcellular Location	Target Class	Pathway
PSMB7	proteasome 20S subunit beta 7	Component of the 20S core proteasome complex involved in the proteolytic degradation of most intracellular proteins. This complex plays numerous essential roles within the cell by associating with different regulatory particles.	Cytoplasm, Nucleus	Enzyme, Unclassified protein	ER-Phagosome pathway
PSMB7	proteasome 20S subunit beta 7	Component of the 20S core proteasome complex involved in the proteolytic degradation of most intracellular proteins. This complex plays numerous essential roles within the cell by associating with different regulatory particles.	Cytoplasm, Nucleus	Enzyme, Unclassified protein	Separation of Sister Chromatids
PSMB7	proteasome 20S subunit beta 7	Component of the 20S core proteasome complex involved in the proteolytic degradation of most intracellular proteins. This complex plays numerous essential roles within the cell by associating with different regulatory particles.	Cytoplasm, Nucleus	Enzyme, Unclassified protein	Negative regulation of NOTCH4 signaling
PSMB7	proteasome 20S subunit beta 7	Component of the 20S core proteasome complex involved in the proteolytic degradation of most intracellular proteins. This complex plays numerous essential roles within the cell by associating with different regulatory particles.	Cytoplasm, Nucleus	Enzyme, Unclassified protein	Orc1 removal from chromatin
PSMB7	proteasome 20S subunit beta 7	Component of the 20S core proteasome complex involved in the proteolytic degradation of most intracellular proteins. This complex plays numerous essential roles within the cell by associating with different regulatory particles.	Cytoplasm, Nucleus	Enzyme, Unclassified protein	GSK3B and BTRC:CUL1-mediated-degradation of NFE2L2
PSMB7	proteasome 20S subunit beta 7	Component of the 20S core proteasome complex involved in the proteolytic degradation of most intracellular proteins. This complex plays numerous essential roles within the cell by associating with different regulatory particles.	Cytoplasm, Nucleus	Enzyme, Unclassified protein	Degradation of GLI2 by the proteasome
PSMB7	proteasome 20S subunit beta 7	Component of the 20S core proteasome complex involved in the proteolytic degradation of most intracellular proteins. This complex plays numerous essential roles within the cell by associating with different regulatory particles.	Cytoplasm, Nucleus	Enzyme, Unclassified protein	CDK-mediated phosphorylation and removal of Cdc6
PSMB7	proteasome 20S subunit beta 7	Component of the 20S core proteasome complex involved in the proteolytic degradation of most intracellular proteins. This complex plays numerous essential roles within the cell by associating with different regulatory particles.	Cytoplasm, Nucleus	Enzyme, Unclassified protein	FBXL7 down-regulates AURKA during mitotic entry and in early mitosis
PSMB7	proteasome 20S subunit beta 7	Component of the 20S core proteasome complex involved in the proteolytic degradation of most intracellular proteins. This complex plays numerous essential roles within the cell by associating with different regulatory particles.	Cytoplasm, Nucleus	Enzyme, Unclassified protein	CLEC7A (Dectin-1) signaling
PSMB7	proteasome 20S subunit beta 7	Component of the 20S core proteasome complex involved in the proteolytic degradation of most intracellular proteins. This complex plays numerous essential roles within the cell by associating with different regulatory particles.	Cytoplasm, Nucleus	Enzyme, Unclassified protein	Hh mutants are degraded by ERAD
PSMB7	proteasome 20S subunit beta 7	Component of the 20S core proteasome complex involved in the proteolytic degradation of most intracellular proteins. This complex plays numerous essential roles within the cell by associating with different regulatory particles.	Cytoplasm, Nucleus	Enzyme, Unclassified protein	GLI3 is processed to GLI3R by the proteasome
PSMB7	proteasome 20S subunit beta 7	Component of the 20S core proteasome complex involved in the proteolytic degradation of most intracellular proteins. This complex plays numerous essential roles within the cell by associating with different regulatory particles.	Cytoplasm, Nucleus	Enzyme, Unclassified protein	Degradation of DVL
PSMB7	proteasome 20S subunit beta 7	Component of the 20S core proteasome complex involved in the proteolytic degradation of most intracellular proteins. This complex plays numerous essential roles within the cell by associating with different regulatory particles.	Cytoplasm, Nucleus	Enzyme, Unclassified protein	Neutrophil degranulation
PSMB7	proteasome 20S subunit beta 7	Component of the 20S core proteasome complex involved in the proteolytic degradation of most intracellular proteins. This complex plays numerous essential roles within the cell by associating with different regulatory particles.	Cytoplasm, Nucleus	Enzyme, Unclassified protein	Regulation of ornithine decarboxylase (ODC)

Target	Approved Name	Function Descriptions*	Subcellular Location	Target Class	Pathway
PSMB7	proteasome 20S subunit beta 7	Component of the 20S core proteasome complex involved in the proteolytic degradation of most intracellular proteins. This complex plays numerous essential roles within the cell by associating with different regulatory particles.	Cytoplasm, Nucleus	Enzyme, Unclassified protein	SCF-beta-TrCP mediated degradation of Emi1
PSMB7	proteasome 20S subunit beta 7	Component of the 20S core proteasome complex involved in the proteolytic degradation of most intracellular proteins. This complex plays numerous essential roles within the cell by associating with different regulatory particles.	Cytoplasm, Nucleus	Enzyme, Unclassified protein	Neddylation
PSMB7	proteasome 20S subunit beta 7	Component of the 20S core proteasome complex involved in the proteolytic degradation of most intracellular proteins. This complex plays numerous essential roles within the cell by associating with different regulatory particles.	Cytoplasm, Nucleus	Enzyme, Unclassified protein	Vif-mediated degradation of APOBEC3G
PSMB7	proteasome 20S subunit beta 7	Component of the 20S core proteasome complex involved in the proteolytic degradation of most intracellular proteins. This complex plays numerous essential roles within the cell by associating with different regulatory particles.	Cytoplasm, Nucleus	Enzyme, Unclassified protein	Dectin-1 mediated noncanonical NF-kB signaling
PSMB7	proteasome 20S subunit beta 7	Component of the 20S core proteasome complex involved in the proteolytic degradation of most intracellular proteins. This complex plays numerous essential roles within the cell by associating with different regulatory particles.	Cytoplasm, Nucleus	Enzyme, Unclassified protein	FCERI mediated NF-kB activation
PSMB7	proteasome 20S subunit beta 7	Component of the 20S core proteasome complex involved in the proteolytic degradation of most intracellular proteins. This complex plays numerous essential roles within the cell by associating with different regulatory particles.	Cytoplasm, Nucleus	Enzyme, Unclassified protein	NIK-->noncanonical NF-kB signaling
PSMB7	proteasome 20S subunit beta 7	Component of the 20S core proteasome complex involved in the proteolytic degradation of most intracellular proteins. This complex plays numerous essential roles within the cell by associating with different regulatory particles.	Cytoplasm, Nucleus	Enzyme, Unclassified protein	TNFR2 non-canonical NF-kB pathway
PRKCA	protein kinase C alpha	Calcium-activated, phospholipid- and diacylglycerol (DAG)- dependent serine/threonine-protein kinase that is involved in positive and negative regulation of cell proliferation, apoptosis, differentiation, migration and adhesion, tumorigenesis, cardiac hypertrophy, angiogenesis, platelet function and inflammation.	Cytoplasm, Cell membrane, Mitochondrion membrane	Enzyme	Signaling by SCF-KIT
PRKCA	protein kinase C alpha	Calcium-activated, phospholipid- and diacylglycerol (DAG)- dependent serine/threonine-protein kinase that is involved in positive and negative regulation of cell proliferation, apoptosis, differentiation, migration and adhesion, tumorigenesis, cardiac hypertrophy, angiogenesis, platelet function and inflammation.	Cytoplasm, Cell membrane, Mitochondrion membrane	Enzyme	RET signaling
PRKCA	protein kinase C alpha	Calcium-activated, phospholipid- and diacylglycerol (DAG)- dependent serine/threonine-protein kinase that is involved in positive and negative regulation of cell proliferation, apoptosis, differentiation, migration and adhesion, tumorigenesis, cardiac hypertrophy, angiogenesis, platelet function and inflammation.	Cytoplasm, Cell membrane, Mitochondrion membrane	Enzyme	Regulation of KIT signaling
PRKCA	protein kinase C alpha	Calcium-activated, phospholipid- and diacylglycerol (DAG)- dependent serine/threonine-protein kinase that is involved in positive and negative regulation of cell proliferation, apoptosis, differentiation, migration and adhesion, tumorigenesis, cardiac hypertrophy, angiogenesis, platelet function and inflammation.	Cytoplasm, Cell membrane, Mitochondrion membrane	Enzyme	SHC1 events in ERBB2 signaling
PRKCA	protein kinase C alpha	Calcium-activated, phospholipid- and diacylglycerol (DAG)- dependent serine/threonine-protein kinase that is involved in positive and negative regulation of cell proliferation, apoptosis, differentiation, migration and adhesion, tumorigenesis, cardiac hypertrophy, angiogenesis, platelet function and inflammation.	Cytoplasm, Cell membrane, Mitochondrion membrane	Enzyme	Inactivation, recovery and regulation of the phototransduction cascade

Target	Approved Name	Function Descriptions*	Subcellular Location	Target Class	Pathway
PRKCA	protein kinase C alpha	Calcium-activated, phospholipid- and diacylglycerol (DAG)- dependent serine/threonine-protein kinase that is involved in positive and negative regulation of cell proliferation, apoptosis, differentiation, migration and adhesion, tumorigenesis, cardiac hypertrophy, angiogenesis, platelet function and inflammation.	Cytoplasm, Cell membrane, Mitochondrion membrane	Enzyme	HuR (ELAVL1) binds and stabilizes mRNA
PRKCA	protein kinase C alpha	Calcium-activated, phospholipid- and diacylglycerol (DAG)- dependent serine/threonine-protein kinase that is involved in positive and negative regulation of cell proliferation, apoptosis, differentiation, migration and adhesion, tumorigenesis, cardiac hypertrophy, angiogenesis, platelet function and inflammation.	Cytoplasm, Cell membrane, Mitochondrion membrane	Enzyme	Ca2+ pathway
PRKCA	protein kinase C alpha	Calcium-activated, phospholipid- and diacylglycerol (DAG)- dependent serine/threonine-protein kinase that is involved in positive and negative regulation of cell proliferation, apoptosis, differentiation, migration and adhesion, tumorigenesis, cardiac hypertrophy, angiogenesis, platelet function and inflammation.	Cytoplasm, Cell membrane, Mitochondrion membrane	Enzyme	Disinhibition of SNARE formation
PRKCA	protein kinase C alpha	Calcium-activated, phospholipid- and diacylglycerol (DAG)- dependent serine/threonine-protein kinase that is involved in positive and negative regulation of cell proliferation, apoptosis, differentiation, migration and adhesion, tumorigenesis, cardiac hypertrophy, angiogenesis, platelet function and inflammation.	Cytoplasm, Cell membrane, Mitochondrion membrane	Enzyme	Acetylcholine regulates insulin secretion
PRKCA	protein kinase C alpha	Calcium-activated, phospholipid- and diacylglycerol (DAG)- dependent serine/threonine-protein kinase that is involved in positive and negative regulation of cell proliferation, apoptosis, differentiation, migration and adhesion, tumorigenesis, cardiac hypertrophy, angiogenesis, platelet function and inflammation.	Cytoplasm, Cell membrane, Mitochondrion membrane	Enzyme	ROBO receptors bind AKAP5
PRKCA	protein kinase C alpha	Calcium-activated, phospholipid- and diacylglycerol (DAG)- dependent serine/threonine-protein kinase that is involved in positive and negative regulation of cell proliferation, apoptosis, differentiation, migration and adhesion, tumorigenesis, cardiac hypertrophy, angiogenesis, platelet function and inflammation.	Cytoplasm, Cell membrane, Mitochondrion membrane	Enzyme	EGFR Transactivation by Gastrin
PRKCA	protein kinase C alpha	Calcium-activated, phospholipid- and diacylglycerol (DAG)- dependent serine/threonine-protein kinase that is involved in positive and negative regulation of cell proliferation, apoptosis, differentiation, migration and adhesion, tumorigenesis, cardiac hypertrophy, angiogenesis, platelet function and inflammation.	Cytoplasm, Cell membrane, Mitochondrion membrane	Enzyme	G alpha (z) signalling events
PRKCA	protein kinase C alpha	Calcium-activated, phospholipid- and diacylglycerol (DAG)- dependent serine/threonine-protein kinase that is involved in positive and negative regulation of cell proliferation, apoptosis, differentiation, migration and adhesion, tumorigenesis, cardiac hypertrophy, angiogenesis, platelet function and inflammation.	Cytoplasm, Cell membrane, Mitochondrion membrane	Enzyme	VEGFR2 mediated cell proliferation
PRKCA	protein kinase C alpha	Calcium-activated, phospholipid- and diacylglycerol (DAG)- dependent serine/threonine-protein kinase that is involved in positive and negative regulation of cell proliferation, apoptosis, differentiation, migration and adhesion, tumorigenesis, cardiac hypertrophy, angiogenesis, platelet function and inflammation.	Cytoplasm, Cell membrane, Mitochondrion membrane	Enzyme	Calmodulin induced events
PRKCA	protein kinase C alpha	Calcium-activated, phospholipid- and diacylglycerol (DAG)- dependent serine/threonine-protein kinase that is involved in positive and negative regulation of cell proliferation, apoptosis, differentiation, migration and adhesion, tumorigenesis, cardiac hypertrophy, angiogenesis, platelet function and inflammation.	Cytoplasm, Cell membrane, Mitochondrion membrane	Enzyme	Syndecan interactions

Target	Approved Name	Function Descriptions*	Subcellular Location	Target Class	Pathway
PRKCA	protein kinase C alpha	Calcium-activated, phospholipid- and diacylglycerol (DAG)- dependent serine/threonine-protein kinase that is involved in positive and negative regulation of cell proliferation, apoptosis, differentiation, migration and adhesion, tumorigenesis, cardiac hypertrophy, angiogenesis, platelet function and inflammation.	Cytoplasm, Cell membrane, Mitochondrion membrane	Enzyme	RHO GTPases Activate NADPH Oxidases
PRKCA	protein kinase C alpha	Calcium-activated, phospholipid- and diacylglycerol (DAG)- dependent serine/threonine-protein kinase that is involved in positive and negative regulation of cell proliferation, apoptosis, differentiation, migration and adhesion, tumorigenesis, cardiac hypertrophy, angiogenesis, platelet function and inflammation.	Cytoplasm, Cell membrane, Mitochondrion membrane	Enzyme	WNT5A-dependent internalization of FZD4
PRKCA	protein kinase C alpha	Calcium-activated, phospholipid- and diacylglycerol (DAG)- dependent serine/threonine-protein kinase that is involved in positive and negative regulation of cell proliferation, apoptosis, differentiation, migration and adhesion, tumorigenesis, cardiac hypertrophy, angiogenesis, platelet function and inflammation.	Cytoplasm, Cell membrane, Mitochondrion membrane	Enzyme	Depolymerisation of the Nuclear Lamina
PRKCA	protein kinase C alpha	Calcium-activated, phospholipid- and diacylglycerol (DAG)- dependent serine/threonine-protein kinase that is involved in positive and negative regulation of cell proliferation, apoptosis, differentiation, migration and adhesion, tumorigenesis, cardiac hypertrophy, angiogenesis, platelet function and inflammation.	Cytoplasm, Cell membrane, Mitochondrion membrane	Enzyme	Response to elevated platelet cytosolic Ca2+
PRKCA	protein kinase C alpha	Calcium-activated, phospholipid- and diacylglycerol (DAG)- dependent serine/threonine-protein kinase that is involved in positive and negative regulation of cell proliferation, apoptosis, differentiation, migration and adhesion, tumorigenesis, cardiac hypertrophy, angiogenesis, platelet function and inflammation.	Cytoplasm, Cell membrane, Mitochondrion membrane	Enzyme	Trafficking of GluR2-containing AMPA receptors

Legend: *Function descriptions are excerpts from OpenTargets platform summary; All data from OpenTargets Platform Target Database. Ref (190)

Supplementary Figure 1

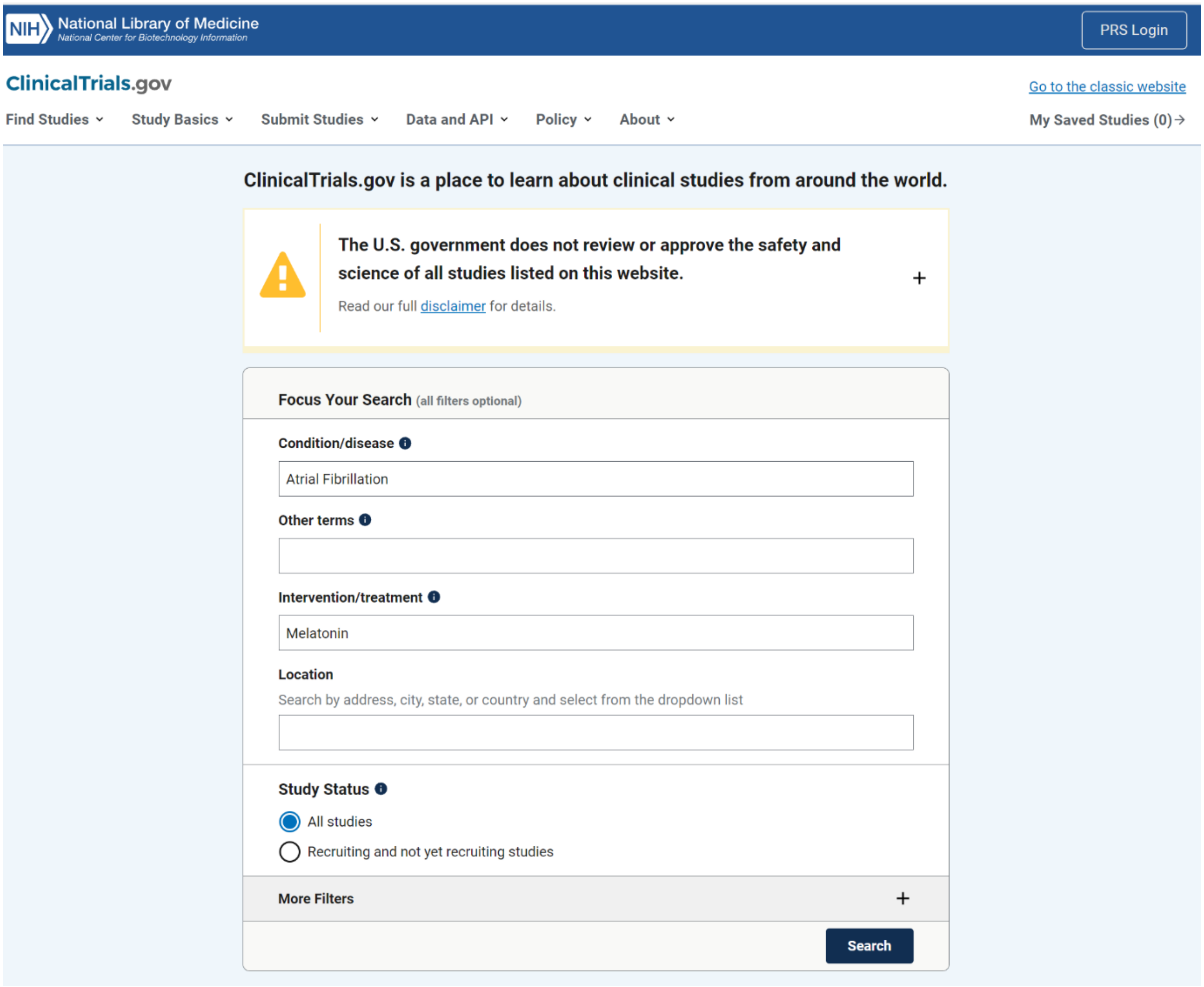

Example search on ClinicalTrials.gov for Melatonin and Atrial Fibrillation

Supplementary Figure 2

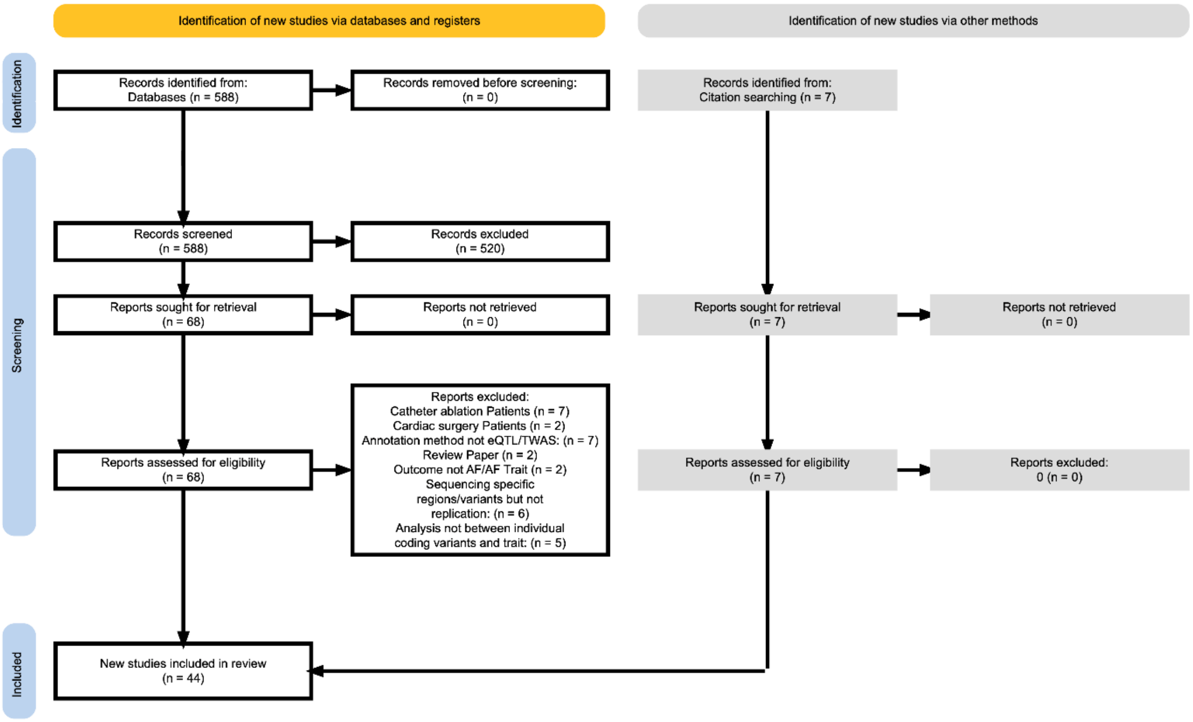

PRIMSA diagram created with PRISMA2020 web application[184]

References:

1. Hwang I, Kwon OS, Park JW, Yu HT, Uhm JS, Joung B, et al. Association of ZFHX3 Genetic Polymorphisms and Extra-Pulmonary Vein Triggers in Patients With Atrial Fibrillation Who Underwent Catheter Ablation. Front Physiol [Internet]. 2022 Jan 5 [cited 2024 Mar 12];12. Available from: https://www.frontiersin.org/journals/physiology/articles/10.3389/fphys.2021.807545/full

2. Ulus T, Dural M, Meşe P, Yetmiş F, Mert KU, Görenek B, et al. Genetic variants associated with atrial fibrillationand long-term recurrence after catheter ablation for atrialfibrillation in Turkish patients. Anatol J Cardiol. 2021;25(2):129–38.

3. Lee JY, Kim TH, Yang PS, Lim HE, Choi EK, Shim J, et al. Korean atrial fibrillation network genome-wide association study for early-onset atrial fibrillation identifies novel susceptibility loci. Eur Heart J. 2017 Sep 7;38(34):2586–94.

4. Husser D, Büttner P, Ueberham L, Dinov B, Sommer P, Arya A, et al. Association of atrial fibrillation susceptibility genes, atrial fibrillation phenotypes and response to catheter ablation: a gene-based analysis of GWAS data. J Transl Med. 2017 Apr 5;15(1):71.

5. Husser D, Büttner P, Ueberham L, Dinov B, Sommer P, Arya A, et al. Genomic Contributors to Rhythm Outcome of Atrial Fibrillation Catheter Ablation – Pathway Enrichment Analysis of GWAS Data. PLOS ONE. 2016 Nov 21;11(11):e0167008.

6. Husser D, Ueberham L, Dinov B, Kosiuk J, Kornej J, Hindricks G, et al. Genomic contributors to atrial electroanatomical remodeling and atrial fibrillation progression: Pathway enrichment analysis of GWAS data. Sci Rep. 2016 Nov 18;6(1):36630.

7. Zhao LQ, Zhang GB, Wen ZJ, Huang CK, Wu HQ, Xu J, et al. Common variants predict recurrence after nonfamilial atrial fibrillation ablation in Chinese Han population. Int J Cardiol. 2017 Jan 15;227:360–6.

8. Meng X, Fan C, Yuan Y. Identification of Atrial Fibrillation-Associated Genes ERBB2 and MYPN Using Genome-Wide Association and Transcriptome Expression Profile Data on Left–Right Atrial Appendages. Front Genet [Internet]. 2021 Jun 30 [cited 2024 Mar 12];12. Available from: https://www.frontiersin.org/journals/genetics/articles/10.3389/fgene.2021.696591/full

9. Wang B, Lunetta KL, Dupuis J, Lubitz SA, Trinquart L, Yao L, et al. Integrative Omics Approach to Identifying Genes Associated With Atrial Fibrillation. Circ Res. 2020 Jan 31;126(3):350–60.

10. van Ouwerkerk AF, Bosada FM, van Duijvenboden K, Hill MC, Montefiori LE, Scholman KT, et al. Identification of atrial fibrillation associated genes and functional non-coding variants. Nat Commun. 2019 Oct 18;10(1):4755.

11. Martin RIR, Babaei MS, Choy MK, Owens WA, Chico TJA, Keenan D. Genetic variants associated with risk of atrial fibrillation regulate expression of PITX2, CAV1, MYOZ1, C9orf3 and FANCC. J Mol Cell Cardiol. 2015 Aug 1;85:207–14.

12. Lin H, Dolmatova EV, Morley MP, Lunetta KL, McManus DD, Magnani JW, et al. Gene expression and genetic variation in human atria. Heart Rhythm. 2014 Feb 1;11(2):266–71.

13. Ebana Y, Ozaki K, Liu L, Hachiya H, Hirao K, Isobe M, et al. Clinical utility and functional analysis of variants in atrial fibrillation-associated locus 4q25. J Cardiol. 2017 Oct 1;70(4):366–73.

14. Clausen AG, Vad OB, Andersen JH, Olesen MS. Loss-of-Function Variants in the SYNPO2L Gene Are Associated With Atrial Fibrillation. Front Cardiovasc Med. 2021 Mar 9;8:650667.

15. Westphal S, Stoppe C, Gruenewald M, Bein B, Renner J, Cremer J, et al. Genome-wide association study of myocardial infarction, atrial fibrillation, acute stroke, acute kidney injury and delirium after cardiac surgery – a sub-analysis of the RIPHeart-Study. BMC Cardiovasc Disord. 2019 Jan 24;19(1):26.

16. Kertai MD, Li YJ, Ji Y, Qi W, Lombard FW, Shah SH, et al. Genome-wide association study of new-onset atrial fibrillation after coronary artery bypass grafting surgery. Am Heart J. 2015 Sep 1;170(3):580-590.e28.

17. Barton AR, Sherman MA, Mukamel RE, Loh PR. Whole-exome imputation within UK Biobank powers rare coding variant association and fine-mapping analyses. Nat Genet. 2021 Aug;53(8):1260–9.

18. Larson MG, Atwood LD, Benjamin EJ, Cupples LA, D'Agostino RB, Fox CS, et al. Framingham Heart Study 100K project: genome-wide associations for cardiovascular disease outcomes. BMC Med Genet. 2007 Sep 19;8(1):S5.

19. Lazarte J, Dron JS, McIntyre AD, Skanes AC, Gula LJ, Tang AS, et al. Role of Common Genetic Variation in Lone Atrial Fibrillation. Circ Genomic Precis Med. 2021 Feb;14(1):e003179.

20. Khera AV, Chaffin M, Aragam KG, Haas ME, Roselli C, Choi SH, et al. Genome-wide polygenic scores for common diseases identify individuals with risk equivalent to monogenic mutations. Nat Genet. 2018 Sep;50(9):1219–24.

21. Weng LC, Choi SH, Klarin D, Smith JG, Loh PR, Chaffin M, et al. Heritability of Atrial Fibrillation. Circ Cardiovasc Genet. 2017 Dec;10(6):e001838.

22. Lin H, Yin X, Xie Z, Lunetta KL, Lubitz SA, Larson MG, et al. Methylome-wide Association Study of Atrial Fibrillation in Framingham Heart Study. Sci Rep. 2017 Jan 9;7(1):40377.

23. Lin H, Mueller-Nurasyid M, Smith AV, Arking DE, Barnard J, Bartz TM, et al. Gene-gene Interaction Analyses for Atrial Fibrillation. Sci Rep. 2016 Nov 8;6(1):35371.

24. Chalazan B, Mol D, Darbar FA, Ornelas-Loredo A, Al-Azzam B, Chen Y, et al. Association of Rare Genetic Variants and Early-Onset Atrial Fibrillation in Ethnic Minority Individuals. JAMA Cardiol. 2021 Jul 1;6(7):811–9.

25. Vad OB, Paludan-Müller C, Ahlberg G, Kalstø SM, Ghouse J, Andreasen L, et al. Loss-of-Function Variants in Cytoskeletal Genes Are Associated with Early-Onset Atrial Fibrillation. J Clin Med. 2020 Feb;9(2):372.

26. Wang P, Qin W, Wang P, Huang Y, Liu Y, Zhang R, et al. Genomic Variants in NEURL, GJA1 and CUX2 Significantly Increase Genetic Susceptibility to Atrial Fibrillation. Sci Rep. 2018 Feb 19;8(1):3297.

27. Kääb S, Darbar D, van Noord C, Dupuis J, Pfeufer A, Newton-Cheh C, et al. Large scale replication and meta-analysis of variants on chromosome 4q25 associated with atrial fibrillation. Eur Heart J. 2009 Apr 1;30(7):813–9.

28. Kim HO, Lim JE, Kim MJ, Kang JO, Kim SM, Nam JM, et al. GAREM1 regulates the PR interval on electrocardiograms. J Hum Genet. 2018 Mar;63(3):297–307.

29. Tucker NR, Dolmatova EV, Lin H, Cooper RR, Ye J, Hucker WJ, et al. Diminished PRRX1 Expression Is Associated With Increased Risk of Atrial Fibrillation and Shortening of the Cardiac Action Potential. Circ Cardiovasc Genet. 2017 Oct;10(5):e001902.

30. Xu C, Zhang R, Xia Y, Xiong L, Yang W, Wang P. Annotation of susceptibility SNPs associated with atrial fibrillation. Aging. 2020 Sep 9;12(17):16981–98.

31. Campbell HM, Wehrens XHT. Genetics of atrial fibrillation: an update. Curr Opin Cardiol. 2018 May;33(3):304.

32. Wang X, Ryu J, Kim J, Ramirez A, Mayo KR, Condon H, et al. Common and rare variants associated with cardiometabolic traits across 98,622 whole-genome sequences in the All of Us research program. J Hum Genet. 2023 Aug;68(8):565–70.

33. Miyazawa K, Ito K, Ito M, Zou Z, Kubota M, Nomura S, et al. Cross-ancestry genome-wide analysis of atrial fibrillation unveils disease biology and enables cardioembolic risk prediction. Nat Genet. 2023;55(2):187–97.

34. Emmert DB, Vukovic V, Dordevic N, Weichenberger CX, Losi C, D'Elia Y, et al. Genetic and Metabolic Determinants of Atrial Fibrillation in a General Population Sample: The CHRIS Study. Biomolecules. 2021 Nov;11(11):1663.

35. Ahlberg G, Andreasen L, Ghouse J, Bertelsen L, Bundgaard H, Haunsø S, et al. Genome-wide association study identifies 18 novel loci associated with left atrial volume and function. Eur Heart J. 2021 Nov 21;42(44):4523–34.

36. Baldassari AR, Sitlani CM, Highland HM, Arking DE, Buyske S, Darbar D, et al. Multi-Ethnic Genome-Wide Association Study of Decomposed Cardioelectric Phenotypes Illustrates Strategies to Identify and Characterize Evidence of Shared Genetic Effects for Complex Traits. Circ Genomic Precis Med. 2020 Aug;13(4):e002680.

37. Weng LC, Hall AW, Choi SH, Jurgens SJ, Haessler J, Bihlmeyer NA, et al. Genetic Determinants of Electrocardiographic P-wave Duration and Relation to Atrial Fibrillation. Circ Genomic Precis Med. 2020 Oct;13(5):387–95.

38. Ntalla I, Weng LC, Cartwright JH, Hall AW, Sveinbjornsson G, Tucker NR, et al. Multi-ancestry GWAS of the electrocardiographic PR interval identifies 202 loci underlying cardiac conduction. Nat Commun. 2020 May 21;11:2542.

39. van Setten J, Verweij N, Mbarek H, Niemeijer MN, Trompet S, Arking DE, et al. Genome-wide association meta-analysis of 30,000 samples identifies seven novel loci for quantitative ECG traits. Eur J Hum Genet. 2019 Jun;27(6):952–62.

40. Choi SH, Weng LC, Roselli C, Lin H, Haggerty CM, Shoemaker MB, et al. Association Between Titin Loss-of-Function Variants and Early-Onset Atrial Fibrillation. JAMA. 2018 Dec 11;320(22):2354–64.

41. Thorolfsdottir RB, Sveinbjornsson G, Sulem P, Nielsen JB, Jonsson S, Halldorsson GH, et al. Coding variants in RPL3L and MYZAP increase risk of atrial fibrillation. Commun Biol. 2018 Jun 12;1:68.

42. Nielsen JB, Thorolfsdottir RB, Fritsche LG, Zhou W, Skov MW, Graham SE, et al. Biobank-driven genomic discovery yields new insight into atrial fibrillation biology. Nat Genet. 2018 Sep;50(9):1234–9.

43. van Setten J, Brody JA, Jamshidi Y, Swenson BR, Butler AM, Campbell H, et al. PR interval genome-wide association meta-analysis identifies 50 loci associated with atrial and atrioventricular electrical activity. Nat Commun. 2018 Jul 25;9(1):2904.

44. Roselli C, Chaffin MD, Weng LC, Aeschbacher S, Ahlberg G, Albert CM, et al. Multi-ethnic genome-wide association study for atrial fibrillation. Nat Genet. 2018 Sep;50(9):1225–33.

45. Nielsen JB, Fritsche LG, Zhou W, Teslovich TM, Holmen OL, Gustafsson S, et al. Genome-wide Study of Atrial Fibrillation Identifies Seven Risk Loci and Highlights Biological Pathways and Regulatory Elements Involved in Cardiac Development. Am J Hum Genet. 2018 Jan 4;102(1):103–15.

46. Seyerle AA, Lin HJ, Gogarten SM, Stilp A, Méndez-Giraldez R, Soliman EZ, et al. Genome-Wide Association Study of PR Interval in Hispanics/Latinos Identifies Novel Locus at ID2. Heart Br Card Soc. 2018 Jun;104(11):904–11.

47. Lin H, van Setten J, Smith AV, Bihlmeyer NA, Warren HR, Brody JA, et al. Common and Rare Coding Genetic Variation Underlying the Electrocardiographic PR Interval. Circ Genomic Precis Med. 2018 May;11(5):e002037.

48. Thorolfsdottir RB, Sveinbjornsson G, Sulem P, Helgadottir A, Gretarsdottir S, Benonisdottir S, et al. A Missense Variant in *PLEC* Increases Risk of Atrial Fibrillation. J Am Coll Cardiol. 2017 Oct 24;70(17):2157–68.

49. Christophersen IE, Magnani JW, Yin X, Barnard J, Weng LC, Arking DE, et al. Fifteen Genetic Loci Associated With the Electrocardiographic P Wave. Circ Cardiovasc Genet. 2017 Aug;10(4):e001667.

50. Low SK, Takahashi A, Ebana Y, Ozaki K, Christophersen IE, Ellinor PT, et al. Identification of six new genetic loci associated with atrial fibrillation in the Japanese population. Nat Genet. 2017 Jun;49(6):953–8.

51. Christophersen IE, Rienstra M, Roselli C, Yin X, Geelhoed B, Barnard J, et al. Large-scale analyses of common and rare variants identify 12 new loci associated with atrial fibrillation. Nat Genet. 2017 Jun;49(6):946–52.

52. Yamada Y, Sakuma J, Takeuchi I, Yasukochi Y, Kato K, Oguri M, et al. Identification of TNFSF13, SPATC1L, SLC22A25 and SALL4 as novel susceptibility loci for atrial fibrillation by an exome-wide association study. Mol Med Rep. 2017 Nov;16(5):5823–32.

53. Gudbjartsson DF, Holm H, Sulem P, Masson G, Oddsson A, Magnusson OTh, et al. A frameshift deletion in the sarcomere gene MYL4 causes early-onset familial atrial fibrillation. Eur Heart J. 2017 Jan 1;38(1):27–34.

54. Tsai CT, Hsieh CS, Chang SN, Chuang EY, Ueng KC, Tsai CF, et al. Genome-wide screening identifies a KCNIP1 copy number variant as a genetic predictor for atrial fibrillation. Nat Commun. 2016 Feb 2;7(1):10190.

55. Gudbjartsson DF, Helgason H, Gudjonsson SA, Zink F, Oddson A, Gylfason A, et al. Large-scale whole-genome sequencing of the Icelandic population. Nat Genet. 2015 May;47(5):435–44.

56. Verweij N, Leach IM, van den Boogaard M, van Veldhuisen DJ, Christoffels VM, Hillege HL, et al. Genetic Determinants of P Wave Duration and PR Segment. Circ Cardiovasc Genet. 2014 Aug;7(4):475–81.

57. Hong KW, Lim JE, Kim JW, Tabara Y, Ueshima H, Miki T, et al. Identification of three novel genetic variations associated with electrocardiographic traits (QRS duration and PR interval) in East Asians. Hum Mol Genet. 2014 Dec 15;23(24):6659–67.

58. Butler AM, Yin X, Evans DS, Nalls MA, Smith EN, Tanaka T, et al. Novel Loci Associated with PR Interval in a Genome-Wide Association Study of Ten African American Cohorts. Circ Cardiovasc Genet. 2012 Dec 1;5(6):639–46.

59. Ellinor PT, Lunetta KL, Albert CM, Glazer NL, Ritchie MD, Smith AV, et al. Meta-analysis identifies six new susceptibility loci for atrial fibrillation. Nat Genet. 2012 Apr 29;44(6):670–5.

60. Smith JG, Magnani JW, Palmer C, Meng YA, Soliman EZ, Musani SK, et al. Genome-Wide Association Studies of the PR Interval in African Americans. PLoS Genet. 2011 Feb 10;7(2):e1001304.

61. Ellinor PT, Lunetta KL, Glazer NL, Pfeufer A, Alonso A, Chung MK, et al. Common Variants in KCNN3 are Associated with Lone Atrial Fibrillation. Nat Genet. 2010 Mar;42(3):240–4.

62. Holm H, Gudbjartsson DF, Arnar DO, Thorleifsson G, Thorgeirsson G, Stefansdottir H, et al. Several common variants modulate heart rate, PR interval and QRS duration. Nat Genet. 2010 Feb;42(2):117–22.

63. Pfeufer A, van Noord C, Marciante KD, Arking DE, Larson MG, Smith AV, et al. Genome-wide association study of PR interval. Nat Genet. 2010 Feb;42(2):153–9.

64. Chambers JC, Zhao J, Terracciano CMN, Bezzina CR, Zhang W, Kaba R, et al. Genetic variation in SCN10A influences cardiac conduction. Nat Genet. 2010 Feb;42(2):149–52.

65. Benjamin EJ, Rice KM, Arking DE, Pfeufer A, van Noord C, Smith AV, et al. Variants in ZFHX3 are associated with atrial fibrillation in individuals of European ancestry. Nat Genet. 2009 Aug;41(8):879–81.

66. Gudbjartsson DF, Holm H, Gretarsdottir S, Thorleifsson G, Walters GB, Thorgeirsson G, et al. A sequence variant in ZFHX3 on 16q22 associates with atrial fibrillation and ischemic stroke. Nat Genet. 2009 Aug;41(8):876–8.

67. Smith JG, Lowe JK, Kovvali S, Maller JB, Salit J, Daly MJ, et al. Genome-wide association study of electrocardiographic conduction measures in an isolated founder population: Kosrae. Heart Rhythm Off J Heart Rhythm Soc. 2009 May;6(5):634–41.

68. Vasan RS, Larson MG, Aragam J, Wang TJ, Mitchell GF, Kathiresan S, et al. Genome-wide association of echocardiographic dimensions, brachial artery endothelial function and treadmill exercise responses in the Framingham Heart Study. BMC Med Genet. 2007 Sep 19;8(Suppl 1):S2.

69. Gudbjartsson DF, Arnar DO, Helgadottir A, Gretarsdottir S, Holm H, Sigurdsson A, et al. Variants conferring risk of atrial fibrillation on chromosome 4q25. Nature. 2007 Jul;448(7151):353–7.

70. Cusanovich DA, Caliskan M, Billstrand C, Michelini K, Chavarria C, De Leon S, et al. Integrated analyses of gene expression and genetic association studies in a founder population. Hum Mol Genet. 2016 May 15;25(10):2104–12.

71. Gonzalez-Cordero AF, Duconge-Soler J, Franqui-Rivera H, Feliu-Maldonado R, Roche-Lima A, Almodovar-Rivera I. Insight on the Genetics of Atrial Fibrillation in Puerto Rican Hispanics. Stroke Res Treat. 2021 Jan 8;2021:e8819896.

72. Hsu J, Gore-Panter S, Tchou G, Castel L, Lovano B, Moravec CS, et al. Genetic Control of Left Atrial Gene Expression Yields Insights into the Genetic Susceptibility for Atrial Fibrillation. Circ Genomic Precis Med. 2018 Mar;11(3):e002107.

73. Sinner MF, Tucker NR, Lunetta KL, Ozaki K, Smith JG, Trompet S, et al. Integrating Genetic, Transcriptional, and Functional Analyses to Identify Five Novel Genes for Atrial Fibrillation. Circulation. 2014 Oct 7;130(15):1225–35.

74. Sano M, Kamitsuji S, Kamatani N, Hong KW, Han BG, Kim Y, et al. Genome-wide association study of electrocardiographic parameters identifies a new association for PR interval and confirms previously reported associations. Hum Mol Genet. 2014 Dec 15;23(24):6668–76.

75. Lubitz SA, Lunetta KL, Lin H, Arking DE, Trompet S, Li G, et al. Novel Genetic Markers Associate with Atrial Fibrillation Risk in Europeans and Japanese. J Am Coll Cardiol. 2014 Apr 1;63(12):1200–10.

76. Ismayl M, Abbasi MA, Marar R, Geske JB, Gersh BJ, Anavekar NS. Mavacamten Treatment for Hypertrophic Cardiomyopathy: A Systematic Review and Meta-

Analysis of Randomized Controlled Trials. Curr Probl Cardiol. 2023 Jan 1;48(1):101429.

77. Solomon SD, Claggett BL, Miao ZM, Diaz R, Felker GM, McMurray JJV, et al. Influence of atrial fibrillation on efficacy and safety of omecamtiv mecarbil in heart failure: the GALACTIC-HF trial. Eur Heart J. 2022 Jun 14;43(23):2212–20.

78. Teerlink JR, Diaz R, Felker GM, McMurray JJV, Metra M, Solomon SD, et al. Cardiac Myosin Activation with Omecamtiv Mecarbil in Systolic Heart Failure. N Engl J Med. 2021 Jan 14;384(2):105–16.

79. Scirica BM, Belardinelli L, Chaitman BR, Waks JW, Volo S, Karwatowska-Prokopczuk E, et al. Effect of ranolazine on atrial fibrillation in patients with non-ST elevation acute coronary syndromes: observations from the MERLIN-TIMI 36 trial. EP Eur. 2015 Jan 1;17(1):32–7.

80. Reiffel JA, Camm AJ, Belardinelli L, Zeng D, Karwatowska-Prokopczuk E, Olmsted A, et al. The HARMONY Trial: Combined Ranolazine and Dronedarone in the Management of Paroxysmal Atrial Fibrillation: Mechanistic and Therapeutic Synergism. Circ Arrhythm Electrophysiol. 2015 Oct;8(5):1048–56.

81. Ferrari GMD, Maier LS, Mont L, Schwartz PJ, Simonis G, Leschke M, et al. Ranolazine in the treatment of atrial fibrillation: Results of the dose-ranging RAFFAELLO (Ranolazine in Atrial Fibrillation Following An ELectricaL CardiOversion) study. Heart Rhythm. 2015 May 1;12(5):872–8.

82. Kirchhof P, Benussi S, Kotecha D, Ahlsson A, Atar D, Casadei B, et al. 2016 ESC Guidelines for the management of atrial fibrillation developed in collaboration with EACTS. Eur J Cardio-Thorac Surg Off J Eur Assoc Cardio-Thorac Surg. 2016 Nov;50(5):e1–88.

83. Martin RIR, Pogoryelova O, Koref MS, Bourke JP, Teare MD, Keavney BD. Atrial fibrillation associated with ivabradine treatment: meta-analysis of randomised controlled trials. Heart. 2014 Oct 1;100(19):1506–10.

84. Chan YH, Hai JJ, Wong CK, Lau CP, Cheung BMY, Tse HF. Ventricular rate control with ivabradine in patients with permanent atrial fibrillation. J Interv Card Electrophysiol. 2022 Dec 1;65(3):597–9.

85. Hu WS, Lin CL. Beta blocker versus ivabradine for cardiovascular outcomes among patients with atrial fibrillation. Postgrad Med J. 2022 Aug 22;

86. JCS Joint Working Group. Guidelines for Pharmacotherapy of Atrial Fibrillation (JCS 2013): – Digest Version –. Circ J. 2014;78(8):1997–2021.

87. Yamase M, Nakazato Y, Daida H. Effectiveness of amiodarone versus bepridil in achieving conversion to sinus rhythm in patients with persistent atrial fibrillation: a randomised trial. Heart. 2012 Jul 15;98(14):1067–71.

88. Ozawa M, Komatsu T, Sato Y, Kunugita F, Tachibana H, Tashiro A, et al. Comparison of the effects of bepridil and aprindine for the prevention of atrial fibrillation after

cardiac and aortic surgery: A prospective randomized study. J Arrhythmia. 2015 Oct;31(5):302–6.

89. Arsenault KA, Yusuf AM, Crystal E, Healey JS, Morillo CA, Nair GM, et al. Interventions for preventing post-operative atrial fibrillation in patients undergoing heart surgery. Cochrane Database Syst Rev. 2013 Jan 31;2013(1):CD003611.

90. Hindricks G, Potpara T, Dagres N, Arbelo E, Bax JJ, Blomström-Lundqvist C, et al. 2020 ESC Guidelines for the diagnosis and management of atrial fibrillation developed in collaboration with the European Association of Cardio-Thoracic Surgery (EACTS). Eur Heart J. 2020;1–126.

91. January CT, Wann LS, Alpert JS, Calkins H, Cigarroa JE, Cleveland JC, et al. 2014 AHA/ACC/HRS Guideline for the Management of Patients With Atrial Fibrillation: Executive Summary. Circulation. 2014 Dec 2;130(23):2071–104.

92. Iliuta L, Christodorescu R, Filpescu D, Moldovan H, Radulescu B, Vasile R. Prevention of perioperative atrial fibrillation with betablockers in coronary surgery: betaxolol versus metoprolol. Interact Cardiovasc Thorac Surg. 2009 Jul 1;9(1):89–93.

93. Piccini JP, Abraham WT, Dufton C, Carroll IA, Healey JS, van Veldhuisen DJ, et al. GENETIC-AF: Bucindolol for the Maintenance of Sinus Rhythm in a Genotype-Defined Heart Failure Population. JACC Heart Fail. 2019 Jul;7(7):586–98.

94. Sezai A, Minami K, Nakai T, Hata M, Yoshitake I, Wakui S, et al. Landiolol hydrochloride for prevention of atrial fibrillation after coronary artery bypass grafting: New evidence from the PASCAL trial. J Thorac Cardiovasc Surg. 2011 Jun 1;141(6):1478–87.

95. Gianni C, Sanchez JE, Mohanty S, Trivedi C, Della Rocca DG, Al-Ahmad A, et al. High-Dose Dobutamine for Inducibility of Atrial Arrhythmias During Atrial Fibrillation Ablation. JACC Clin Electrophysiol. 2020 Dec;6(13):1701–10.

96. Neumann J, Azatsian K, Höhm C, Hofmann B, Gergs U. Cardiac effects of ephedrine, norephedrine, mescaline, and 3,4-methylenedioxymethamphetamine (MDMA) in mouse and human atrial preparations. Naunyn Schmiedebergs Arch Pharmacol. 2023;396(2):275–87.

97. Sharma PL. EFFECTS OF ADRENALINE AND NORADRENALINE ON ATRIAL FIBRILLATION PRODUCED BY ACETYLCHOLINE: WITH OBSERVATIONS ON THE MECHANISM OF FIBRILLATION. Q J Exp Physiol Cogn Med Sci. 1964 Apr 7;49(2):134–40.

98. van der Hooft CS, Heeringa J, van Herpen G, Kors JA, Kingma JH, Stricker BHCh. Drug-induced atrial fibrillation. J Am Coll Cardiol. 2004 Dec 7;44(11):2117–24.

99. Haywood LJ, Ford CE, Crow RS, Davis BR, Massie BM, Einhorn PT, et al. Atrial Fibrillation at Baseline and During Follow-Up in ALLHAT (Antihypertensive and Lipid-Lowering Treatment to Prevent Heart Attack Trial). J Am Coll Cardiol. 2009 Nov 24;54(22):2023–31.

100. Aoki J, Iguchi Y, Urabe T, Yamagami H, Todo K, Fujimoto S, et al. Cilostazol uncovers covert atrial fibrillation in non-cardioembolic stroke. J Neurol Sci. 2020 Jun;413:116796.

101. Toyonaga S, Nakatsu T, Murakami T, Kusachi S, Mashima K, Tominaga Y, et al. Effects of cilostazol on heart rate and its variation in patients with atrial fibrillation associated with bradycardia. J Cardiovasc Pharmacol Ther. 2000 Jul;5(3):183–91.

102. Zhao Z, Li R, Wang X, Li J, Xu X, Liu T, et al. Suppression of experimental atrial fibrillation in a canine model of rapid atrial pacing by the phosphodiesterase 3 inhibitor cilostazol. J Electrocardiol. 2020 May 1;60:151–8.

103. Zhao Z, Wang Y, Chen Y, Wang X, Li J, Yuan M, et al. Cilostazol Prevents Atrial Structural Remodeling through the MEK/ERK Pathway in a Canine Model of Atrial Tachycardia. Cardiology. 2016 Jan 1;135(4):240–8.

104. Feneck RO, Sherry KM, Withington PS, Oduro-Dominah A, European Milrinone Multicenter Trial Group. Comparison of the hemodynamic effects of milrinone with dobutamine in patients after cardiac surgery. J Cardiothorac Vasc Anesth. 2001 Jun;15(3):306–15.

105. Fleming GA, Murray KT, Yu C, Byrne JG, Greelish JP, Petracek MR, et al. Milrinone Use is Associated With Postoperative Atrial Fibrillation Following Cardiac Surgery. Circulation. 2008 Oct 14;118(16):1619–25.

106. Kandasamy A, Simon HA, Murthy P, Annadurai M, Ali MM, Ramanathan G. Comparison of Levosimendan versus Dobutamine in Patients with Moderate to Severe Left Ventricular Dysfunction Undergoing Off-pump Coronary Artery Bypass Grafting: A Randomized Prospective Study. Ann Card Anaesth. 2017;20(2):200–6.

107. Abacilar AF, Dogan OF. Levosimendan use decreases atrial fibrillation in patients after coronary artery bypass grafting: a pilot study. Heart Surg Forum. 2013 Oct;16(5):E287-294.

108. Mebazaa A, Nieminen MS, Packer M, Cohen-Solal A, Kleber FX, Pocock SJ, et al. Levosimendan vs Dobutamine for Patients With Acute Decompensated Heart FailureThe SURVIVE Randomized Trial. JAMA. 2007 May 2;297(17):1883–91.

109. A Comparison of Rate Control and Rhythm Control in Patients with Atrial Fibrillation. N Engl J Med. 2002 Dec 5;347(23):1825–33.

110. Akkuş M, Öner E. Can local infiltration of lidocaine reduce the postoperative atrial fibrillation rate in patients undergoing lobectomy for lung cancer? Acta Chir Belg. 2020 Jul 3;120(4):265–70.

111. Marrouche NF, Reddy RK, Wittkowsky AK, Bardy GH. High-dose bolus lidocaine for chemical cardioversion of atrial fibrillation: A prospective, randomized, double-blind crossover trial. Am Heart J. 2000 Jun 1;139(6):E8–11.

112. Egstrup K, Bergfeldt L, Duris T, Gullestad L, Kochmanski M, Kuśnierz B, et al. QT Response after a Test Dose and during Maintenance Therapy with AZD1305 in Patients with Atrial Fibrillation. Am J Cardiovasc Drugs. 2011 Jun 1;11(3):199–208.

113. Rónaszéki A, Alings M, Egstrup K, Gaciong Z, Hranai M, Király C, et al. Pharmacological cardioversion of atrial fibrillation—a double-blind, randomized, placebo-controlled, multicentre, dose-escalation study of AZD1305 given intravenously. EP Eur. 2011 Aug 1;13(8):1148–56.

114. Crijns HJ, Van Gelder IC, Walfridsson H, Kulakowski P, Rónaszéki A, Dedek V, et al. Safe and effective conversion of persistent atrial fibrillation to sinus rhythm by intravenous AZD7009. Heart Rhythm. 2006 Nov 1;3(11):1321–31.

115. Geller JC, Egstrup K, Kulakowski P, Rosenqvist M, Jansson MA, Berggren A, et al. Rapid Conversion of Persistent Atrial Fibrillation to Sinus Rhythm by Intravenous AZD7009. J Clin Pharmacol. 2009;49(3):312–22.

116. Hohnloser SH, Dorian P, Straub M, Beckmann K, Kowey P. Safety and efficacy of intravenously administered tedisamil for rapid conversion of recent-onset atrial fibrillation or atrial flutter. J Am Coll Cardiol. 2004 Jul 7;44(1):99–104.

117. Stiell IG, Sivilotti MLA, Taljaard M, Birnie D, Vadeboncoeur A, Hohl CM, et al. A randomized, controlled comparison of electrical versus pharmacological cardioversion for emergency department patients with acute atrial flutter. Can J Emerg Med. 2021 May 1;23(3):314–24.

118. Stiell IG, Sivilotti MLA, Taljaard M, Birnie D, Vadeboncoeur A, Hohl CM, et al. Electrical versus pharmacological cardioversion for emergency department patients with acute atrial fibrillation (RAFF2): a partial factorial randomised trial. The Lancet. 2020 Feb 1;395(10221):339–49.

119. Scheuermeyer FX, Andolfatto G, Christenson J, Villa-Roel C, Rowe B. A Multicenter Randomized Trial to Evaluate a Chemical-first or Electrical-first Cardioversion Strategy for Patients With Uncomplicated Acute Atrial Fibrillation. Acad Emerg Med. 2019;26(9):969–81.

120. Geelen P, O’Hara GE, Roy N, Talajic M, Roy D, Plante S, et al. Comparison of propafenone versus procainamide for the acute treatment of atrial fibrillation after cardiac surgery. Am J Cardiol. 1999 Aug 1;84(3):345–7.

121. Gold MR, O’Gara PT, Buckley MJ, DeSanctis RW. Efficacy and safety of procainamide in preventing arrhythmias after coronary artery bypass surgery. Am J Cardiol. 1996 Nov 1;78(9):975–9.

122. Laub GW, Janeira L, Muralidharan S, Riebman JB, Chen C, Neary M, et al. Prophylactic procainamide for prevention of atrial fibrillation after coronary artery bypass grafting: A prospective, double-blind, randomized, placebo-controlled pilot study. Crit Care Med. 1993 Oct;21(10):1474–8.

123. Sezai A, Iida M, Yoshitake I, Wakui S, Osaka S, Kimura H, et al. Carperitide and Atrial Fibrillation After Coronary Bypass Grafting. Circ Arrhythm Electrophysiol. 2015 Jun;8(3):546–53.

124. Sezai A, Hata M, Wakui S, Niino T, Takayama T, Hirayama A, et al. Efficacy of Continuous Low-Dose hANP Administration in Patients Undergoing Emergent

Coronary Artery Bypass Grafting for Acute Coronary Syndrome. Circ J. 2007;71(9):1401–7.

125. Wettwer E, Hála O, Christ T, Heubach JF, Dobrev D, Knaut M, et al. Role of IKur in Controlling Action Potential Shape and Contractility in the Human Atrium. Circulation. 2004 Oct 19;110(16):2299–306.

126. Iqbal A, Tekin Z, Kattan MW, Ji X, Milinovich A, Pantalone KM, et al. Association between first-line monotherapy with metformin and the risk of atrial fibrillation (AMRAF) in patients with type 2 diabetes. J Diabetes Complications. 2022 Nov 1;36(11):108315.

127. Fauchier G, Bisson A, Bodin A, Herbert J, Angoulvant D, Ducluzeau PH, et al. Glucose-lowering drug use and new-onset atrial fibrillation in patients with diabetes mellitus. Diabetologia. 2021 Nov;64(11):2602–5.

128. Chang SH, Wu LS, Chiou MJ, Liu JR, Yu KH, Kuo CF, et al. Association of metformin with lower atrial fibrillation risk among patients with type 2 diabetes mellitus: A population-based dynamic cohort and in vitro studies. Cardiovasc Diabetol. 2014;13(1):1–8.

129. Shi W, Zhang W, Zhang D, Ren G, Wang P, Gao L, et al. Comparison of the effect of glucose-lowering agents on the risk of atrial fibrillation: A network meta-analysis. Heart Rhythm. 2021 Jul;18(7):1090–6.

130. Oral H. Metformin as an Adjunctive Therapy to Catheter Ablation in Atrial Fibrillation [Internet]. clinicaltrials.gov; 2023 Feb [cited 2023 May 10]. Report No.: NCT04625946. Available from: https://clinicaltrials.gov/ct2/show/NCT04625946

131. MD MC. Upstream Targeting for the Prevention of Atrial Fibrillation: Targeting Risk Interventions and Metformin for Atrial Fibrillation (TRIM-AF) [Internet]. clinicaltrials.gov; 2023 Jan [cited 2023 May 10]. Report No.: NCT03603912. Available from: https://clinicaltrials.gov/ct2/show/NCT03603912

132. Shaibani A, Fares S, Selam JL, Arslanian A, Simpson J, Sen D, et al. Lacosamide in painful diabetic neuropathy: an 18-week double-blind placebo-controlled trial. J Pain. 2009 Aug;10(8):818–28.

133. Munger M. ASsessment Of RiLuzole To Reduce Paroxysmal Episodes of Atrial FIbrillatiON (The SOLUTION Study) [Internet]. clinicaltrials.gov; 2022 Nov [cited 2023 May 10]. Report No.: NCT05292209. Available from: https://clinicaltrials.gov/ct2/show/NCT05292209

134. Lin AL, Nah G, Tang JJ, Vittinghoff E, Dewland TA, Marcus GM. Cannabis, cocaine, methamphetamine, and opiates increase the risk of incident atrial fibrillation. Eur Heart J. 2022 Dec 14;43(47):4933–42.

135. University of Minnesota. Paravertebral Block to Reduce the Incidence of New Onset Atrial Fibrillation After Cardiac Surgery: A Prospective Randomized Controlled Pilot Trial [Internet]. clinicaltrials.gov; 2023 Mar [cited 2023 May 10]. Report No.: NCT04472299. Available from: https://clinicaltrials.gov/ct2/show/NCT04472299

136. Patterson E, Scherlag BJ, Zhou J, Jackman WM, Lazzara R, Coscia D, et al. Antifibrillatory Actions of Cisatracurium: An Atrial Specific M2 Receptor Antagonist. J Cardiovasc Electrophysiol. 2008;19(8):861–8.

137. Patterson E, Lu Z, Lin J, Scherlag BJ, Po SS, Coscia D, et al. Antifibrillatory Properties of Mivacurium in a Canine Model of Atrial Fibrillation. J Cardiovasc Pharmacol. 2008 Mar;51(3):293.

138. Varriale P, Ramaprasad S. Aminophylline Induced Atrial Fibrillation. Pacing Clin Electrophysiol. 1993;16(10):1953–5.

139. Tisdale JE, Chung MK, Campbell KB, Hammadah M, Joglar JA, Leclerc J, et al. Drug-Induced Arrhythmias: A Scientific Statement From the American Heart Association. Circulation. 2020 Oct 13;142(15):e214–33.

140. Huerta C, Lanes SF, García Rodríguez LA. Respiratory Medications and the Risk of Cardiac Arrhythmias. Epidemiology. 2005 May;16(3):360.

141. Alboni P, Menozzi C, Brignole M, Paparella N, Gaggioli G, Lolli G, et al. Effects of Permanent Pacemaker and Oral Theophylline in Sick Sinus Syndrome: The THEOPACE Study: A Randomized Controlled Trial. Circulation. 1997 Jul;96(1):260–6.

142. Oba Y, Lone NA. Efficacy and safety of roflumilast in patients with chronic obstructive pulmonary disease: a systematic review and meta-analysis. Ther Adv Respir Dis. 2013 Feb 1;7(1):13–24.

143. Papi A, Vestbo J, Fabbri L, Corradi M, Prunier H, Cohuet G, et al. Extrafine inhaled triple therapy versus dual bronchodilator therapy in chronic obstructive pulmonary disease (TRIBUTE): a double-blind, parallel group, randomised controlled trial. The Lancet. 2018 Mar 17;391(10125):1076–84.

144. Osch D van, Dieleman JM, Dijk D van, Jacob KA, Kluin J, Doevendans PA, et al. Dexamethasone for the prevention of postoperative atrial fibrillation. Int J Cardiol. 2015 Mar 1;182:431–7.

145. Jacob KA, Dieleman JM, Nathoe HM, Van Osch D, De Waal EEC, Cramer MJ, et al. The effects of intraoperative dexamethasone on left atrial function and postoperative atrial fibrillation in cardiac surgical patients. Neth Heart J. 2015 Mar;23(3):168–73.

146. Yared JP, Bakri MH, Erzurum SC, Moravec CS, Laskowski DM, Wagoner DRV, et al. Effect of Dexamethasone on Atrial Fibrillation After Cardiac Surgery: Prospective, Randomized, Double-Blind, Placebo-Controlled Trial. J Cardiothorac Vasc Anesth. 2007 Feb 1;21(1):68–75.

147. Abbaszadeh M, Khan ZH, Mehrani F, Jahanmehr H. Corticosteroides intravenosos no perioperatório reduzem a incidência de fibrilação atrial após cirurgia cardíaca: estudo randomizado. Rev Bras Cir Cardiovasc. 2012;27(1):18–23.

148. Prasongsukarn K, Abel JG, Jamieson WRE, Cheung A, Russell JA, Walley KR, et al. The effects of steroids on the occurrence of postoperative atrial fibrillation after

coronary artery bypass grafting surgery: A prospective randomized trial. J Thorac Cardiovasc Surg. 2005 Jul 1;130(1):93–8.

149. Yared JP, Starr NJ, Torres FK, Bashour CA, Bourdakos G, Piedmonte M, et al. Effects of single dose, postinduction dexamethasone on recovery after cardiac surgery. Ann Thorac Surg. 2000 May;69(5):1420–4.

150. Whitlock RP, Chan S, Devereaux PJ, Sun J, Rubens FD, Thorlund K, et al. Clinical benefit of steroid use in patients undergoing cardiopulmonary bypass: a meta-analysis of randomized trials. Eur Heart J. 2008 Nov 1;29(21):2592–600.

151. Agboola KM, Dietrich M, Karki R, Lodhi F, McGill T, Asirvatham SJ, et al. Single-Dose Intraprocedural Steroid Administration Does Not Impact Early Atrial Fibrillation Recurrence. Cardiovasc Drugs Ther. 2023 Feb 1;37(1):151–7.

152. Rozencwajg S, Desthieux C, Szymkiewicz O, Ynineb Y, Fulgencio JP, Bonnet F. The risk of atrial fibrillation after pneumonectomy is not impaired by preoperative administration of dexamethasone. A cohort study. Anaesth Crit Care Pain Med. 2017 Jun 1;36(3):185–9.

153. Mahrose R, Elsayed AM, Elshorbagy MS. Bisoprolol Versus Corticosteroid and Bisoprolol Combination for Prevention of Atrial Fibrillation After On-Pump Coronary Artery Bypass Graft Surgery. Open Anesth J. 2019 Apr 30;13(1):18–24.

154. Viviano A, Kanagasabay R, Zakkar M. Is perioperative corticosteroid administration associated with a reduced incidence of postoperative atrial fibrillation in adult cardiac surgery? Interact Cardiovasc Thorac Surg. 2014 Feb;18(2):225–9.

155. Alhadidy MAA. Combined Use of Dexmedetomidine and Hydrocortisone to Prevent New Onset Atrial Fibrillation After Coronary Artery Bypass Grafting Surgery [Internet]. clinicaltrials.gov; 2023 Jan [cited 2023 May 10]. Report No.: NCT05674253. Available from: https://clinicaltrials.gov/ct2/show/NCT05674253

156. Minneapolis Heart Institute Foundation. SAAB: Randomized, Double Blind STudy of Corticosteroid Pulse After Ablation [Internet]. clinicaltrials.gov; 2019 Jul [cited 2023 May 8]. Report No.: NCT00807586. Available from: https://clinicaltrials.gov/ct2/show/NCT00807586

157. Iskandar S, Reddy M, Afzal MR, Rajasingh J, Atoui M, Lavu M, et al. Use of Oral Steroid and its Effects on Atrial Fibrillation Recurrence and Inflammatory Cytokines Post Ablation – The Steroid AF Study. J Atr Fibrillation. 2017 Feb 28;9(5):1604.

158. Gong IY, Atzema CL, Lega IC, Austin PC, Na Y, Rochon PA, et al. Levothyroxine dose and risk of atrial fibrillation: A nested case-control study. Am Heart J. 2021 Feb 1;232:47–56.

159. Pantos CI, Trikas AG, Pissimisis EG, Grigoriou KP, Stougiannos PN, Dimopoulos AK, et al. Effects of Acute Triiodothyronine Treatment in Patients with Anterior Myocardial Infarction Undergoing Primary Angioplasty: Evidence from a Pilot Randomized Clinical Trial (ThyRepair Study). Thyroid. 2022 Jun;32(6):714–24.

160. Tharmapoopathy M, Thavarajah A, Kenny RPW, Pingitore A, Iervasi G, Dark J, et al. Efficacy and Safety of Triiodothyronine Treatment in Cardiac Surgery or Cardiovascular Diseases: A Systematic Review and Meta-Analysis of Randomized Controlled Trials. Thyroid Off J Am Thyroid Assoc. 2022 Aug;32(8):879–96.

161. Janssen J, Löwenberg B, Manz M, Bargetzi M, Biemond B, Borne P von dem, et al. Inferior Outcome of Addition of the Aminopeptidase Inhibitor Tosedostat to Standard Intensive Treatment for Elderly Patients with AML and High Risk MDS. Cancers. 2021 Feb 7;13(4):672.

162. Lai X, Wan Q, Jiao SF, Sun XC, Hu JF, Peng HW. Cardiovascular toxicities following the use of tyrosine kinase inhibitors in hepatocellular cancer patients: a retrospective, pharmacovigilance study. Expert Opin Drug Saf. 2023;0(0):1–10.

163. Mehdizadeh M, Naud P, Abu-Taha IH, Hiram R, Xiong F, Xiao J, et al. The role of cellular senescence in profibrillatory atrial remodelling associated with cardiac pathology. Cardiovasc Res. 2024 Jan 5;cvae003.

164. Wu WC, Huang CC, Tsai YF, Lin YS, Feng CJ, Chen YJ, et al. The association of trastuzumab with atrial fibrillation and heart failure in breast cancer patients in routine clinical practice: a population-based propensity score matching and competing risk model analysis. Breast Cancer Res Treat. 2023;198(1):113–22.

165. Abdel-Qadir H, Thavendiranathan P, Fung K, Amir E, Austin PC, Anderson GS, et al. Association of Early-Stage Breast Cancer and Subsequent Chemotherapy With Risk of Atrial Fibrillation. JAMA Netw Open. 2019 Sep 20;2(9):e1911838.

166. Al-Yafeai Z, Ghoweba M, Ananthaneni A, Abduljabar H, Aziz D. Cardiovascular complications of modern multiple myeloma therapy: A pharmacovigilance study. Br J Clin Pharmacol. 2023;89(2):641–8.

167. Ahmad J, Thurlapati A, Thotamgari S, Grewal US, Sheth AR, Gupta D, et al. Anti-cancer Drugs Associated Atrial Fibrillation—An Analysis of Real-World Pharmacovigilance Data. Front Cardiovasc Med. 2022 Apr 15;9:739044.

168. Alexandre J, Salem JE, Moslehi J, Sassier M, Ropert C, Cautela J, et al. Identification of anticancer drugs associated with atrial fibrillation: analysis of the WHO pharmacovigilance database. Eur Heart J - Cardiovasc Pharmacother. 2021 Jul 23;7(4):312–20.

169. Gridelli C, Cigolari S, Gallo C, Manzione L, Ianniello GP, Frontini L, et al. Activity and toxicity of gemcitabine and gemcitabine+vinorelbine in advanced non-small-cell lung cancer elderly patients: Phase II data from the Multicenter Italian Lung Cancer in the Elderly Study (MILES) randomized trial. Lung Cancer. 2001 Mar 1;31(2):277–84.

170. Bretagne M, Lebrun-Vignes B, Pariente A, Shaffer CM, Malouf GG, Dureau P, et al. Heart failure and atrial tachyarrhythmia on abiraterone: A pharmacovigilance study. Arch Cardiovasc Dis. 2020 Jan 1;113(1):9–21.

171. Huang WF, Tsai YW, Wen YW, Hsiao FY, Kuo KN, Tsai CR. Osteoporosis treatment and atrial fibrillation: alendronate versus raloxifene. Menopause. 2010 Jan;17(1):57.

172. Perez MV, Wang PJ, Larson JC, Virnig BA, Cochrane B, Curb JD, et al. Effects of postmenopausal hormone therapy on incident atrial fibrillation: the Women's Health Initiative randomized controlled trials. Circ Arrhythm Electrophysiol. 2012 Dec 1;5(6):1108–16.

173. Tsai WC, Haung YB, Kuo HF, Tang WH, Hsu PC, Su HM, et al. Hormone replacement therapy and risk of atrial fibrillation in Taiwanese menopause women: A nationwide cohort study. Sci Rep. 2016 Apr 7;6:24132.

174. Kommu S, Arepally S. The Effect of Colchicine on Atrial Fibrillation: A Systematic Review and Meta-Analysis. Cureus. 15(2):e35120.

175. Tsai YT, Lin FY, Lin CS, Loh SH, Li CY, Lin CY, et al. B-type natriuretic peptide enhances fibrotic effects via matrix metalloproteinase-2 expression in the mouse atrium in vivo and in human atrial myofibroblasts in vitro. Transl Res. 2019 Jun 1;208:30–46.

176. Gemel J, Simon AR, Patel D, Xu Q, Matiukas A, Veenstra RD, et al. Degradation of a Connexin40 mutant linked to atrial fibrillation is accelerated. J Mol Cell Cardiol. 2014 Sep;74:330–9.

177. Guan YZ, Liu H, Huang HJ, Liang DY, Wu SY, Zhang T. Identification of the Potential Molecular Mechanism of TGFBI Gene in Persistent Atrial Fibrillation. Comput Math Methods Med. 2022 Nov 8;2022:1643674.

178. Chiba S, Hashimoto K. Sustained Atrial Fibrillation Induced by Carbachol, Methacholine and Bethanechol. Jpn J Pharmacol. 1971;21(2):167–73.

179. Jones DL, Tuomi JM, Chidiac P. Role of Cholinergic Innervation and RGS2 in Atrial Arrhythmia. Front Physiol. 2012 Jun 29;3:239.

180. Raskin J, Pritchett YL, Wang F, D'Souza DN, Waninger AL, Iyengar S, et al. A Double-Blind, Randomized Multicenter Trial Comparing Duloxetine with Placebo in the Management of Diabetic Peripheral Neuropathic Pain. Pain Med. 2005 Sep 1;6(5):346–56.

181. Bethge KP, Godt U, Bianco-Cruz E, Lichtlen PR. Incidence of Cardiac Arrhythmias During Antidepressant Therapy with Maprotiline or Nomifensine. J Cardiovasc Pharmacol. 1982 Feb;4(1):142.

182. Barati S, Jahangirifard A, Ahmadi ZH, Tavakoli-Ardakani M, Dastan F. The Effects of Melatonin on the Oxidative Stress and Duration of Atrial Fibrillation after Coronary Artery Bypass Graft Surgery: A Randomized Controlled Trial. Endocr Metab Immune Disord - Drug Targets. 21(6):1142–9.

183. Open Targets Platform [Internet]. [cited 2024 Mar 13]. Available from: https://platform-docs.opentargets.org/target

184. Haddaway NR, Page MJ, Pritchard CC, McGuinness LA. PRISMA2020: An R package and Shiny app for producing PRISMA 2020-compliant flow diagrams, with interactivity for optimised digital transparency and Open Synthesis. Campbell Syst Rev. 2022;18(2):e1230.
